# Synthesis and Isolation of Bismuth Scorpionate Complexes: Toward Bond Activation via Bismuth‐Ligand Cooperation

**DOI:** 10.1002/chem.202502814

**Published:** 2025-11-26

**Authors:** Lennart Hensle, Viktoria H. Gessner

**Affiliations:** ^1^ Faculty of Chemistry and Biochemistry Ruhr‐University Bochum Bochum Germany

**Keywords:** bismuth, bond activation, ligand design, metal‐ligand cooperation, ylide

## Abstract

Over the past twenty years, main group chemistry has significantly expanded its scope to include reactivities once considered the exclusive domain of transition metals. Among the emerging concepts, element‐ligand cooperation has proven to be a powerful strategy for enabling bond activation reactions but remains relatively unexplored for heavier group 15 elements. In this study, we present a novel class of ylide ligands incorporating additional amino donor sites, specifically designed to promote bismuth‐ligand cooperativity. We successfully isolated a series of well‐defined bismuth scorpionate complexes supported by these dianionic *N,C_ylide_,N* ligands, each bearing different substituents at the ylide moiety. These variations modulate the degree of negative charge stabilization, thereby tuning the extent of electron donation to the bismuth center. Halide abstraction generated cationic bismuth complexes, which exhibited enhanced ligand‐to‐bismuth electron donation, resulting in low Lewis acidities and reduced reactivity. In contrast, deprotonation of the ylide ligand bearing an electron‐withdrawing Ar^F^ substituent enabled the synthesis and NMR characterization of a rare bismuth yldiide complex. This yldiide underwent a [2 + 2] addition with a carbodiimide across the formal Bi = C double bond, demonstrating that bismuth yldiides can engage in bond activation through bismuth‐ligand cooperativity.

## 1 Introduction

Historically, catalysis and bond activation have been dominated by transition metals, owing to their ability to efficiently mediate electron transfer between multiple oxidation states, facilitated by the spatial and energetic accessibility of their partially occupied d‐orbitals [1, 2, 3]. However, the high cost, limited availability and potential toxicity of many commonly used transition metals have motivated extensive efforts over the past few decades to develop reactive main group species capable of mimicking and enabling similar types of reactivity [4, 5, 6, 7]. Since main group complexes in their common bonding states generally exhibit large HOMO‐LUMO gaps, the deliberate design of ligands is essential to modulate their electronic, steric, and geometric properties and to enhance their reactivity.

**FIGURE 1:**
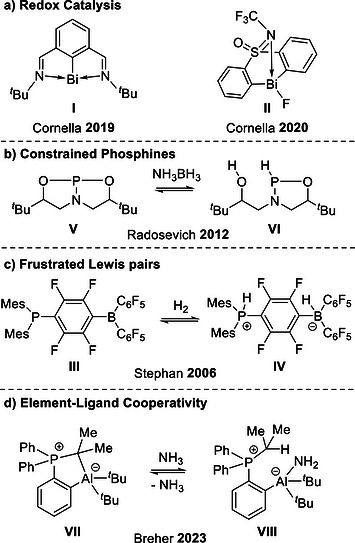
Pioneering catalytic applications of main group species.

Bond activation by group 15 compounds is dominated by three concepts: Transition‐metal like oxidative addition to the element center, bond activation by frustrated Lewis pairs (FLP) [8], and element‐ligand cooperativity [9]. The first activation mode requires a +2 oxidation state change, involving either a low‐valent (+I to +III) or a high‐valent (+III to +V) transition between oxidation states. Impressive advances in this area have been made in recent years [10, 11, 12, 13], such as by *Cornella* in bismuth chemistry, who, for example, employed *Dostal's* monomeric Bi(I) complex **I** [14] as a transfer hydrogenation catalyst [15] or his Bi(III) complex **II** for the fluorination of aryl boronic esters (Figure 1a) [16]. In addition to bismuth, phosphorus has also been shown to undergo oxidative bond activations when constrained into a distorted T‐shaped geometry. The deviation from its typical trigonal pyramidal geometry significantly lowers the LUMO energy, thereby increasing the Lewis acidity of the pnictogen center and resulting in a geometry‐induced change in reactivity [17, 18, 19, 20, 21, 22, 23, 24, 25]. This was demonstrated in pioneering studies by *Radosevich* in 2012 (Figure 1b) based on *Arduengo's* phosphine **V** [26, 27, 28], which functions as a transfer hydrogenation catalyst in the reduction of azobenzenes with ammonia borane as a dihydrogen source. A phosphenium complex recently reported by *Dobrovetsky* even allowed the direct activation of dihydrogen, enabling the reduction of unactivated alkenes [29].

The first application of an FLP system (**III**) in the heterolytic cleavage of dihydrogen was reported by *Stephan* in 2006 (Figure 1c) [30]. This concept is based on the combination of a bulky Lewis acid and a bulky Lewis base, whose tendency for adduct formation is impeded by steric restriction, leaving their intrinsic reactivity for substrate activation [31]. To date, various bulky amines and phosphines in combination with internal or external Lewis acids have been successfully employed to activate H_2_, CO_2_, and other small molecules [32]. In addition, the Lewis acidity of heavier group 15 compounds has likewise been investigated in this context [33, 34, 35, 36].

In the case of element‐ligand cooperation, a Lewis acidic element center and a Lewis basic ligand site typically act in concert to activate a substrate. This concept is well established for transition metals [37, 38, 39, 40], but far less for p‐block complexes [41]. Most reported examples of this reactivity involve Lewis acids of the group 13 elements boron [42, 43, 44, 45, 46] and aluminum [47, 48, 49, 50, 51, 52]. A notable recent example is the ylide‐stabilized aluminum complex **VII** by *Breher* (Figure 1d), which enabled the reversible N−H bond cleavage of ammonia [53], a transformation that is challenging even for transition metal complexes [54].

**SCHEME 1:**
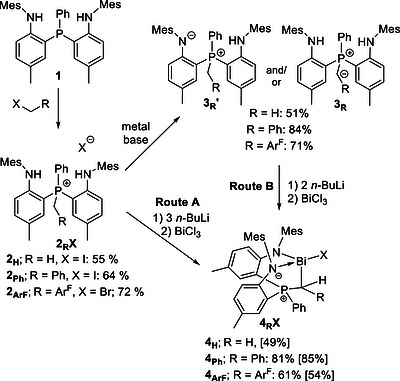
Synthesis of the bismuth complexes **4_R_X** with R = H, Ph, Ar^F^ (Ar^F^ = 3,5‐(CF_3_)_2_C_6_H_3_) and X = Cl, Br, I; Base = LDA for **2_ArF_** and KH for **2_Ph_** and **2_H_**. Yields refer to isolated yields. Yields in brackets refer to route B. For reaction conditions, see the Supporting Information.

In efforts to broaden the scope of group 15 element‐ligand cooperative chemistry, the heavier congeners antimony and bismuth represent promising targets due to their increased Lewis acidity compared to phosphorus [55]. Although bismuth possesses an occupied 6s orbital, the large energetic gap to the 6p orbitals and relativistic effects [56, 57] render this lone pair chemically inert unless treated with a strong oxidant. This inertness enables bismuth salts to function as catalysts in Lewis acid‐mediated processes [58], like Diels Alder cycloadditions [59] and Friedel‐Crafts acylations [60]. However, the application of bismuth in element‐ligand cooperativity is still rare. In 2016, *Suzuki* and *Yamashita* isolated a stable bismabenzene, which reacted in a [4 + 2] cycloaddition with dimethyl acetylenedicarboxylate to simultaneously form a Bi–C and a C–C bond within the ligand backbone [61]. A related hetero‐Diels‐Alder reaction has also been reported for Bi(I) pincer complexes by *Dostal* [62, 63]. More recently, Lichtenberg and coworkers reported on a series of addition reactions of C = X bonds across Bi‐N bonds, including carbon dioxide and carbon disulfide as well as ethylene [64, 65, 66].

In recent years, our group has focused on the isolation and utilization of metalated ylides for the stabilization of reactive main group species [67], including Lewis acidic boron [68] and phosphenium cations [69] or heavier carbene analogs [70, 71, 72, 73]. These studies have shown that ylide ligands can actively engage in bond activations by functioning as Brønsted base [74, 75]. We hypothesized that this reactivity could be extended to bismuth complexes, allowing for substrate activation through the combination of a Lewis acidic bismuth center and a basic ylide ligand.

## 2 Results and Discussion

### 2.1 Ligand Synthesis

To investigate the potential of bismuth ylide^1^ complexes to enable element‐ligand cooperation in a similar fashion to aluminum complex **VII**, we designed a novel ylide ligand featuring additional amido donor sites. Given that heteroleptic bismuth complexes are often susceptible to ligand scrambling and elimination due to the inherently low bismuth‐element bond strengths, we employed a chelating scorpionate ligand framework to promote the formation of well‐defined and robust complexes suitable for a systematic investigation of element–ligand cooperative effects. As a starting point for our synthesis, we selected the diaminophosphine **1** previously reported by Fryzuk [76]. We aimed for its conversion into the corresponding phosphonium salts **2_R_X** and their ylides **3_R_** to finally generate the *N,C_ylide_,N*‐scorpionate complexes **4_R_X** (Scheme 1). In this design, the nitrogen donors serve to stabilize the coordination sphere of bismuth, while the ylidic carbon atom acts as a Lewis basic site to facilitate the bifunctional substrate activation in cooperation with the Lewis acidic bismuth center. Furthermore, variation of the alkyl halide in the synthesis of **2_R_X** enables the incorporation of various R substituents that modulate the stability of the negative charge at the ylidic carbon center in **4_R_X**. To assess the impact of these electronic effects on reactivity, we chose hydrogen, phenyl, and 3,5‐trifluoromethylphenyl (Ar^F^) as representative substituents.

**FIGURE 2:**
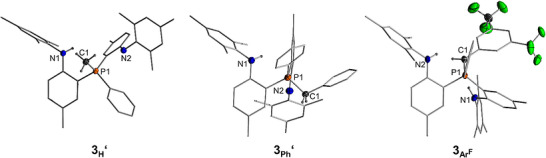
Molecular structures of the zwitterionic ligand precursors **3_H_**, **3_Ph_,** and **3_Ar_F**. All hydrogen atoms except for the C1, N1, and N2 protons and additional solvent molecules are omitted for clarity. Ellipsoids are drawn at the 50% probability level. Crystallographic details are given in the Supporting Information.

The reaction of various alkyl halides with phosphine **1** afforded the corresponding phosphonium salts **2_R_X**, which could be isolated as colorless solids in 55 to 72% yield. The salts are characterized by singlets between 17.0 and 12.4 ppm in the ^31^P{^1^H} NMR spectrum and by doublets for the CH_2/_CH_3_ moiety adjacent to phosphorus in the ^1^H NMR spectrum, with coupling constants of approx. 15 Hz. Subsequent deprotonation of the phosphonium salts with one equivalent of a metal amide or hydride led to the formation of the corresponding zwitterionic species. Depending on the stabilizing effect of the substituent R, proton abstraction occurred either at one of the nitrogen atoms yielding **3_R_’,** or in the α‐position to the phosphorus atom yielding ylide **3_R_**. ^1^H and ^31^P{^1^H} NMR spectroscopy, as well as X‐ray diffraction (XRD) analyses (Figure 2) allowed us to identify the preferred site of deprotonation in each compound: For **3_H_**, deprotonation occurs selectively at the nitrogen atom, as evidenced by a slight shift of the ^31^P{^1^H} NMR signal from 17.0 ppm in **2_H_I** to 11.8 ppm and a broad ^1^H NMR resonance at 2.84 ppm (integrating to three protons) corresponding to the methyl group attached at the phosphorus center. In contrast, compound **3_Ar_F** exhibits a pronounced high‐field shift in the ^31^P{^1^H} NMR spectrum from 15.9 to 2.5 ppm, clearly indicating ylide formation. No signals assignable to the protons on the amine nitrogen or the ylidic carbon center of **3_Ar_F** were observed in the ^1^H NMR spectrum at room temperature. However, variable‐temperature (VT) NMR studies at ‐40°C revealed a high‐field shifted resonance for the ylidic CH_2_ group appearing at 3.18 ppm (cf. 6.05 ppm for **2_ArF_Br**), providing further evidence for predominant ylide formation. Thus, the ^1^H NMR data indicate the presence of an amide‐ylide equilibrium in solution, which lies predominantly on the ylide side, resulting in the observed high‐field shift in the ^31^P NMR signal. A similar situation is observed for the phenyl derivative **3_Ph_**, which also exhibits an equilibrium at room temperature; however, this equilibrium appears to be shifted more toward the amide tautomer, as suggested by the absence of a pronounced high‐field shift in the ^31^P NMR spectrum.

In the solid‐state structures of the zwitterions **3**, only the solution‐predominant tautomer was detected. Accordingly, XRD analysis of **3_Ar_F** confirmed the formation of the ylide tautomer, whereas the amide form was observed in the solid‐state structure of **3_H_** and **3_Ph_**. The ylide character of **3_Ar_F** was, for example, evidenced by locating only a single H atom in the difference Fourier map at the C1 carbon atom of **3_Ar_F**, as well as by a shortened P1–C1 bond length of 1.710(2) Å compared to 1.801(1) Å in amide **3_H_** and 1.834(2) Å in **3_Ph_**. Due to the strong electron‐withdrawing nature of the Ar^F^ group, **3_Ar_F** is the only ligand precursor that preferentially adopts the ylide rather than the amide form.

### 2.2 Bismuth Halide Complexes

With the ligand precursors at hand, defined bismuth halide complexes **4_R_X** were synthesized using either the phosphonium salts **2_R_X** or the zwitterionic compounds **3_R_** (Scheme 1). The choice of ligand precursor was found to determine the nature of the halide X coordinated to bismuth in the final complex. When using the neutral zwitterionic precursors **3_R_**, the chloride from the used bismuth salt BiCl_3_ was incorporated due to the absence of any competing halide anion. Conversely, when the phosphonium salts were employed, coordination of the heavier halide from the salt precursor was preferred, consistent with the HSAB principle.

**FIGURE 3:**
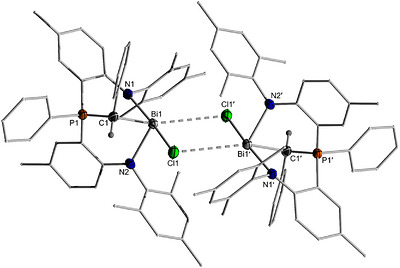
Molecular halide structure of **4_Ph_Cl**. All other bismuth halide complexes feature the same dimeric structures. Hydrogen atoms, except for the one at C1, and additional solvent molecules are omitted for clarity. Ellipsoids are drawn at the 50% probability level. Crystallographic details are given in Table 1 and the Supporting Information.

**FIGURE 4:**
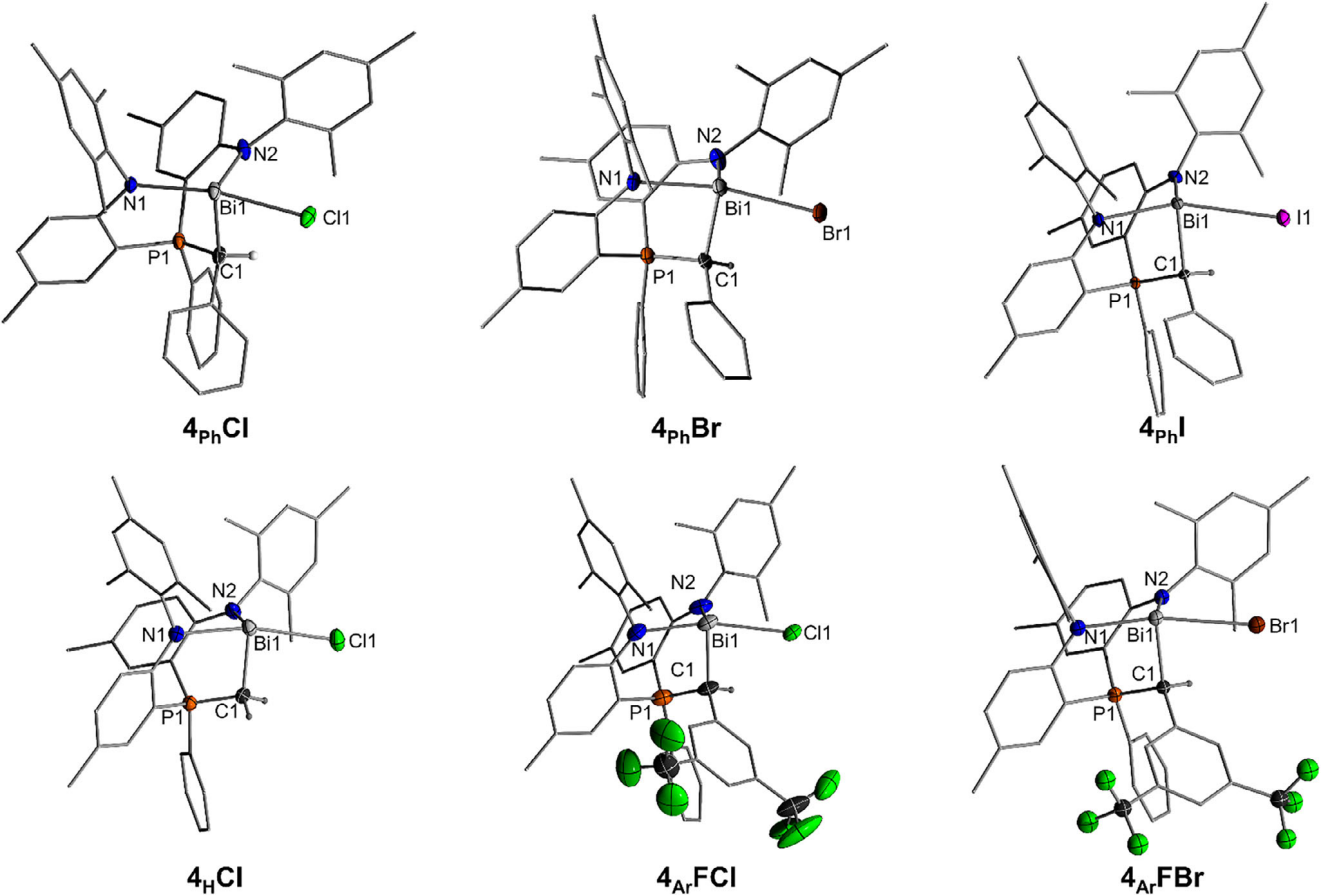
Molecular structures of the phenyl‐substituted bismuth complexes **4_Ph_Cl**, **4_Ph_Br,** and **4_Ph_I** (top) and the hydrogen‐substituted complex **4_H_Cl,** as well as the Ar^F^‐substituted complexes **4_ArF_Cl** and **4_ArF_Br** (bottom). All hydrogen atoms except for the C1 protons and additional solvent molecules are omitted for clarity. Ellipsoids are drawn at the 50% probability level. Crystallographic details are given in Table 1 and the Supporting Information.

At first, we focused on the synthesis of the complexes with the phenyl‐substituted ligand. Starting from the corresponding precursors **2_Ph_** and **3_Ph_**, respectively, the three bismuth(III) halide complexes **4_Ph_Cl**, **4_Ph_Br**, and **4_Ph_I** were successfully isolated and characterized, including by XRD analysis. In the solid state, all complexes form dimeric structures in which the halide ligands act as μ_2_‐bridges between bismuth centers (Figure 3). However, the interaction between the two monomeric units within the dimers is weak, as evidenced by the markedly different Bi‐X distances, which differ significantly but remain within the sum of the van der Waals radii (Table 1). In the monomeric subunits, the bismuth center adopts a bisphenoidal coordination geometry defined by the ylidic carbon atom, the two nitrogen donors, and the halide ligand (Figure 4). Comparison of the Bi−N bond lengths reveals that the Bi‐N1 bond, located trans to the more strongly bound halide X1, is consistently longer than the Bi1−N2 bond. This bond length difference (ΔBi–N) decreases across the series **4_Ph_Cl**, **4_Ph_Br,** and **4_Ph_I**, a trend that can be attributed to the diminishing *trans*‐influence of the halide ligand down the group. In contrast, the Bi–C bond length remains largely unaffected by the nature of the halide, with values ranging from 2.290(4) Å to 2.300(3) Å. The preference for halide coordination trans to N1 rather than to C1 can be rationalized by the stronger *trans*‐influence exerted by the ylide ligand.

**TABLE 1:** Comparison of selected bond lengths [Å] in the isolated bismuth scorpionate complexes.

	4_H_Cl	4_Ph_Cl	4_Ph_Br	4_Ar_FCl	4_Ar_FBr	5_Ph_OTf	5_Ph_BPh_4_
Bi1–X	2.854(1)	2.719(1)	2.873(1)	2.849(1)	3.001(1)	2.774(8)	3.125(1)
Bi1–X‘	3.078(1)	3.457(5)	3.606(4)	3.065(2)	3.246(5)	–	–
Bi1–N1	2.338(3)	2.395(3)	2.396(3)	2.348(4)	2.345(3)	2.242(3)	2.210(3)
Bi1–N2	2.252(3)	2.200(3)	2.214(3)	2.256(4)	2.284(3)	2.188(3)	2.193(3)
ΔBi–N	0.086(6)	0.195(6)	0.182(6)	0.092(8)	0.061(6)	0.054(6)	0.017(6)
Bi1–C1	2.231(4)	2.291(3)	2.290(4)	2.280(4)	2.290(4)	2.271(4)	2.282(3)

The asymmetrical coordination of the amine ligands in the bismuth complexes is maintained in solution as evidenced by ^1^H NMR spectroscopy (recorded in d_8_‐THF), which, for example, shows eight signals for the CH_3_ groups of the tolyl and mesityl groups in the ligand backbone. In contrast, only four signals were observed in the spectra of the symmetric phosphonium salts **2_R_X** and ylides **3_R_**. Furthermore, two sets of aromatic signals were detected for the mesityl and tolyl groups upon complexation. All **4_Ph_X** compounds demonstrate high stability in benzene or THF solution for weeks, even at elevated temperatures, and can be stored indefinitely in the solid state in an argon‐filled glovebox.

To investigate the impact of the substitution pattern and the resulting stabilization of negative charge at the ylidic carbon atom on the coordination geometry of the bismuth center, the H‐substituted complex **4_H_Cl** and the 3,5‐trifluoromethylphenyl‐substituted complexes **4_Ar_FCl** and **4_Ar_FBr** were synthesized and crystallographically characterized (Figure 3). Interestingly, incorporation of the electron‐withdrawing Ar^F^ substituent leads to a more symmetric coordination environment in the dimeric complexes. The Bi‐X distances in **4_Ar_FCl** and **4_Ar_FBr** increase by approx. 0.13 Å relative to the corresponding phenyl‐substituted analogues, leading to a smaller difference between the two Bi‐X bonds (0.217(1) Å and 0.245(3) Å). This change is accompanied by an equalization of the Bi−N distances, with ΔBi–N values of only 0.092(8) Å for **4_Ar_FCl** and 0.061(6) Å for **4_Ar_FBr**. These trends can be attributed to the reduced electron density at Bi induced by the Ar^F^ substituent, which strengthens the interaction in the dimer, resulting in shorter Bi–X1’ bonds and a contraction of the bismuth–nitrogen bonds. The Bi1–C1 bond lengths remain largely unaffected by the change of the ylide substituent. However, in **4_H_Cl**, a slight shortening of the Bi1–C1 bond to 2.231(4) Å is observed, compared to 2.291(3) Å in **4_Ph_Cl**. This is likely due to the absence of a stabilizing aryl substituent, which renders the ylidic carbon more electron‐donating, as well as the reduced steric demand of the hydrogen‐substituted ligand. The Bi–Cl1 and Bi–Cl1’ distances in **4_H_Cl** are similar to those in the Ar^F^ complex **4_Ar_FCl**, while the shortened Bi–Cl1’ contact relative to **4_Ph_Cl** can presumably be rationalized by the decreased steric bulk of the hydrogen substituent compared to phenyl, allowing for a contraction of the dimer. Notably, the ΔBi–N value of 0.086(6) Å is the smallest among all bismuth chloride complexes studied.

### 2.3 Activation of the Bismuth Complexes

**SCHEME 2:**
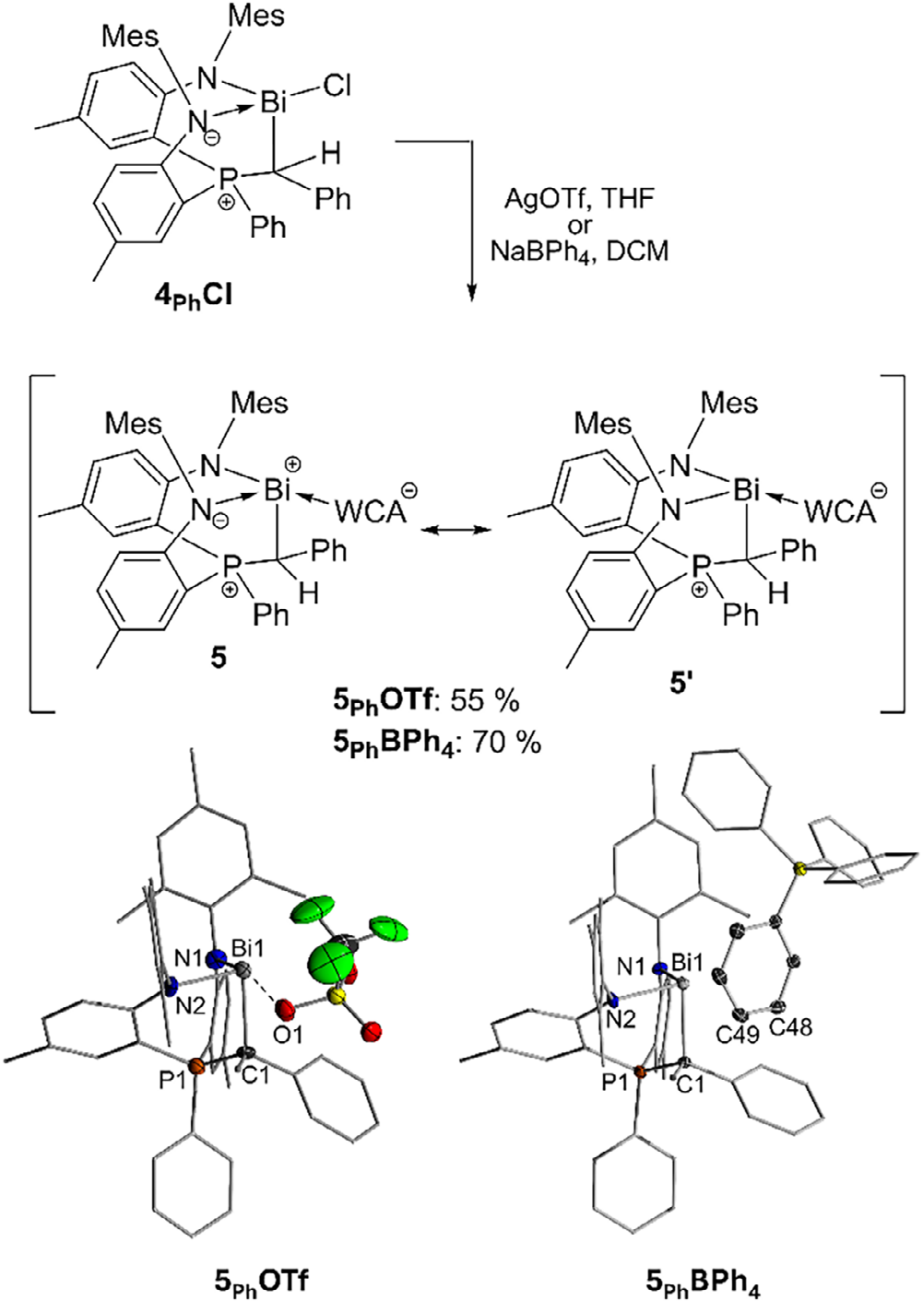
Synthesis of the cationic bismuth complexes **5_Ph_OTf** and **5_Ph_BPh_4_** (top) and their molecular structures (bottom). Yields refer to isolated yields. All hydrogen atoms except for the C1 protons and additional solvent molecules are omitted for clarity. Ellipsoids are drawn at the 50% probability level. Crystallographic details are given in Table 1 and the Supporting Information.

To obtain reactive bismuth complexes capable of activating bonds via metal‐ligand cooperation, we followed two strategies: i) the preparation of Lewis acidic cationic bismuth complexes through halide abstraction and ii) dehydrohalogenation to the corresponding yldiide complexes with a formal Bi = C double bond. At first, the abstraction of the halide ligands and their substitution with weakly coordinating anions (WCA) was investigated. The reaction of **4_Ph_Cl** with equimolar amounts of silver(I) triflate resulted in the selective formation of a new species, as indicated by a shift from 9.55 to 6.70 ppm in the ^31^P{^1^H} NMR spectrum. Crystals suitable for XRD analysis were obtained by the slow diffusion of *n*‐hexane into a concentrated THF solution and confirmed the successful replacement of the halide ligand with the triflate anion (Scheme 2). **5_Ph_OTf** features a symmetric coordination sphere around Bi1, as evidenced by the low ΔBi–N value of only 0.054(6) Å. Overall, the exchange to triflate results in a contraction of the coordination sphere (Table 1). The Bi1−C1 distance slightly decreases by 0.020(7) Å, while the Bi1−N1 bond significantly shortens from 2.395(3) Å to 2.242(3) Å. This contraction can be attributed to the reduced electron density at the Bi1 atom, which enhances the donation from the remaining donor atoms. Nonetheless, a weak interaction between Bi1 and the triflate oxygen atom O1 is observed in the solid state, with a distance of 2.774(8) Å, which is shorter than the sum of their van der Waals radii (3.22 Å). The O1 atom coordinates opposite to the N1 atom, explaining the slight elongation of the Bi1–N1 bond relative to Bi1–N2.

Interactions between bismuth and triflate oxygen atoms or the fluorine atoms of tetrafluoroborate are well‐documented for cationic complexes in literature [77]. Therefore, we selected tetraphenylborate, which lacks donor atoms for such interactions, as an alternative weakly coordinating anion to further investigate its impact on the structure of the complex. Salt metathesis between **4_Ph_Cl** and NaBPh_4_ yielded **5_Ph_BPh_4_**, from which single crystals were obtained through slow evaporation of a concentrated DCM solution. The molecular structure of **5_Ph_BPh_4_** showed the expected contraction in the coordination environment around Bi1, with bismuth ligand distances being slightly shorter than those observed in **5_Ph_OTf**. Notably, the borate complex features the smallest ΔBi–N value of all discussed complexes (ΔBi–N = 0.017(6) Å), indicating a minimal influence of the BPh_4_^−^ counterion. However, a weak interaction between Bi1 and one of the phenyl rings of the anion is still present in the complex, evidenced by short Bi1−C48 and Bi−C49 contacts of 3.220(2) Å and 3.125(1) Å, respectively, which lie well within the sum of their van der Waals radii (3.77 Å).

The Lewis acidities of **5_Ph_OTf** and **5_Ph_BPh_4_** were assessed using the modified Guttmann‐Beckett method for soft Lewis acids developed by Lichtenberg and coworkers [78]. The resulting acceptor numbers of 7.7 for **5_Ph_OTf** and 8.9 for **5_Ph_BPh_4_** indicate only low acidities, which can be attributed to the partial localization of the positive charge at the phosphorus atom (structure **5’**), rendering the bismuth center electronically saturated. This is also reflected in the reactivity of both compounds, **5_Ph_OTf** and **5_Ph_BPh_4_** toward, a series of compounds (e.g., ammonia, alcohols), which resulted in either decomposition, complex product mixtures, or no reaction at all.

Given the inability of the cationic scorpionate complexes to selectively activate substrates through enhanced Lewis acidity, we pursued the synthesis of a bismuth yldiide complex to create a Bi−C linkage with partial multiple bond character suitable for bond activations via element‐ligand cooperativity. Treatment of **4_Ph_I** or **4_Ph_OTf** with 1 equivalent of lithium diisopropylamide (LDA) resulted in the disappearance of the signal of the starting material in the ^31^P{^1^H} NMR spectrum at δ = 9.8 ppm and δ = 7.6 ppm, respectively, and in the selective formation of a new species at δ = 19.8 ppm. However, analysis of the ^1^H NMR spectrum indicated that amination of the bismuth center occurred instead of the targeted deprotonation to the yldiide. Stronger bases such as *n*‐butyllithium gave only uninterpretable mixtures of products, prompting us to investigate the deprotonation of **4_Ar_FBr** with a more acidic ylidic CH moiety. Treatment of **4_Ar_FBr** with 1 equivalent of LDA or potassium hexamethyldisilazide (KHMDS) resulted in the disappearance of the signal of the starting material at δ*_P_* = 9.0 ppm in the ^31^P{^1^H} NMR spectrum within one day and the appearance of a new upfield‐shifted resonance at −4.0 ppm. This upfield shift indicates increased electron density at phosphorus, consistent with the formation of the targeted yldiide **6**. Additionally, the ylidic CH proton disappeared in the ^1^H NMR spectrum, and the methyl groups of the tolyl and mesityl groups became equivalent due to the now symmetrical coordination of the nitrogen atoms in the absence of the halide ligand. Prior to compound **6**, only one bismuth yldiide had been reported by Wesemann in 2013 [79].

**SCHEME 3:**
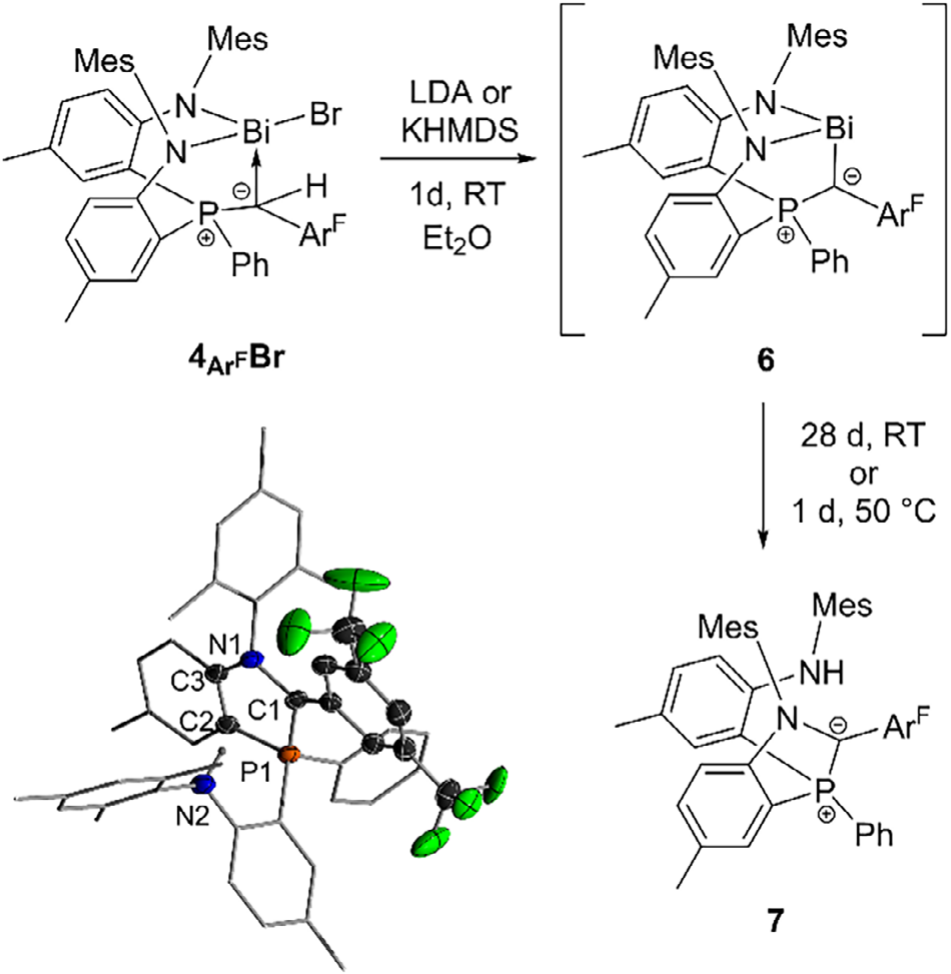
Synthesis of bismuth yldiide **6** and its decomposition via reductive C‐N bond formation to product **7** and crystal structure of **7**. All hydrogen atoms except for the N2 proton and additional solvent molecules are omitted for clarity. Ellipsoids are drawn at the 50% probability level. Crystallographic details are given in Table 1 and the Supporting Information.

Unfortunately, no single crystals suitable for XRD analysis could be obtained. The yldiide complex was found to be highly reactive and gradually decomposed in solution, preventing its isolation in a pure solid form. Prolonged storage of the solution for four weeks—in an attempt to induce crystallization—led to a single decomposition product (**7**), as indicated by a signal at –5.6 ppm in the ^31^P{^1^H} NMR spectrum (Scheme 3). Due to the high solubility of the yldiide in solvents with varying polarity, rapid crystallization to prevent this undesired reaction could not be achieved. When the yldiide solution was heated to 50°C overnight, the decomposition was significantly faster, resulting in complete conversion to **7** overnight. Complex **7** is presumably formed through reductive elimination of the ligand via C−N bond formation. Following the decomposition at different concentrations showed no concentration dependency, supporting an intramolecular decomposition pathway (Figures S3, S23).

**SCHEME 4:**
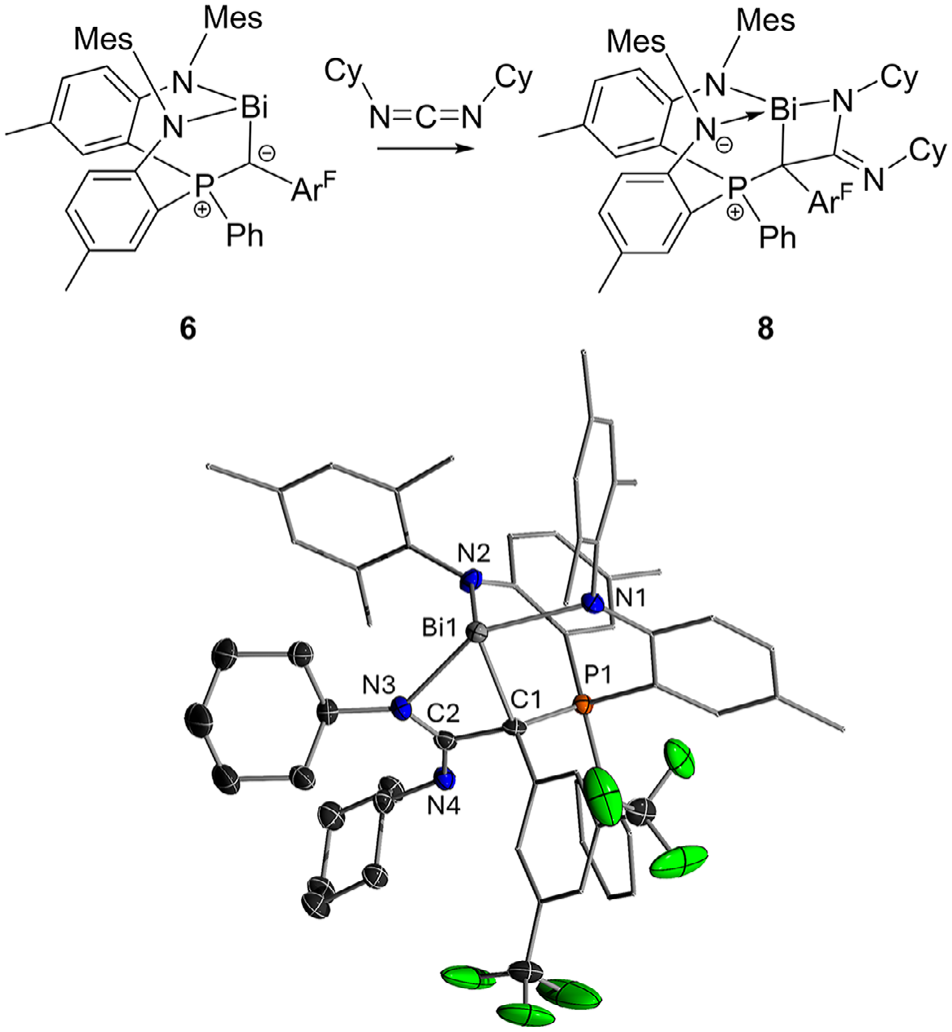
Synthesis and molecular structure of [2 + 2] cycloaddition product **8**. All hydrogen atoms are omitted for clarity. Ellipsoids are drawn at the 50% probability level. Crystallographic details are given in the Supporting Information.

To provide further evidence for the intermediate formation of bismuth yldiide **6**, we attempted it's in situ trapping by reaction with an unsaturated compound. *N*,*N*‐Dicyclohexylcarbodiimide was selected due to its high polarity and ease of activation. In the ^31^P{^1^H} NMR spectrum recorded 30 min after the addition of the carbodiimide to a solution of in situ generated yldiide **6**, the signal corresponding to complex **6** at −4.0 ppm disappeared, and a new product characterized by a signal at 7.2 ppm was formed. The product was identified as the targeted [2 + 2] addition product **8**, formed by the addition of one C = N bond of the carbodiimide across the Bi‐C bond. The complex could be isolated in a 41% yield. In the ^1^H NMR spectrum, compound **8** exhibits an asymmetric coordination environment, as evidenced by eight different new CH_3_ signals. Single crystals suitable for X‐ray diffraction analysis were obtained by slow evaporation of a concentrated toluene solution of the reaction mixture, allowing for unambiguous confirmation of the connectivity of **8** (Scheme 4). The newly formed C−C bond (1.550(4) Å) between the ylidic carbon atom C1 and the N = C = N carbon C2 falls within the range of a typical single bond [80]. The Bi−N3 bond (2.301(3) Å) is intermediate in length compared to the Bi−N1 (2.400(3) Å) and Bi−N2 (2.211(2) Å) bonds to the ligand. This variation reflects the asymmetric coordination environment observed in the ^1^H NMR spectrum and can be attributed to the destabilizing effect of the N3 donor trans to N1 in the distorted bisphenoidal coordination geometry.

The reaction of **6** to **8** confirms the potential of bismuth yldiide complexes to activate substrates through element‐ligand cooperativity. This reactivity is presumably primarily driven by the nucleophilicity of the ylidic carbon. Unfortunately, further attempts to utilize the in situ formed yldiide complex **6** in bond activations remained unsuccessful. Due to the instability of the complex, reactions with different substrates (H_2_, CO_2_, CS_2_ as well as a variety of ketones, aldehydes, and unsaturated hydrocarbons) either resulted in a complex product mixture or re‐protonation.

## 3 Conclusion

In conclusion, we have reported the preparation of a series of bismuth(III) complexes featuring diamino(ylide) scorpionate ligands with different substituents at the ylide moiety. The coordination of the ligands was found to be highly sensitive to changes in the electron density at the bismuth center, as evidenced by distinct bond length changes depending on the nature of the coligands. Substitution of the halide with a weakly coordinating anion generated cationic bismuth complexes that showed increased electron donation from the ligand to bismuth, resulting in a lower Lewis acidity than initially anticipated. Consistently, no clean bond activation reactions could be achieved with these complexes. In contrast, deprotonation of the complex with an electron‐withdrawing Ar^F^ substituent enabled the synthesis and NMR characterization of a rare bismuth yldiide. The yldiide could not be isolated due to its high reactivity and instability toward reductive elimination, but its formation was confirmed by its reaction with *N*,*N’*‐dicyclohexylcarbodiimide, which resulted in a [2 + 2] addition across the formal Bi = C double bond. This reaction highlights the ability of bismuth yldiides to facilitate bond activation through bismuth‐ligand cooperativity. However, further ligand design is needed to enhance the stability of these complexes, enabling a systematic investigation of their potential in bond activation reactions.

## Supporting Information

The authors have cited additional references within the Supporting Information [81, 82, 83, 84, 85, 86]. The Supporting Information includes experimental, spectroscopic and crystallographic details.

Deposition Numbers 2484196 (for **2_Ph_I**), 2484197 (for **3_Ar_F**), 2484198 (for **3_H_**), 2484199 (for **3_Ph_**), 2484200 (for **4_ArF_Br**), 2484201 (for **4_H_Cl**), 2484202 (for **4_Ph_Br**), 2484203 (for **4_Ph_Cl**), 2484204 (for **4_Ph_I**), 2484205 (for **5_Ph_BPh4**), 2484206 (for **5_Ph_OTf**), 2484207 (for **7**), 2484208 (for **8**), 2484209 (for **4_ArF_Cl**) contain the supplementary crystallographic data for this paper. These data are provided free of charge by the joint Cambridge Crystallographic Data Centre and Fachinformationszentrum Karlsruhe Access Structures service.

## Conflicts of Interest

The authors declare no conflicts of interest.

## Supporting information




**Supporting Information File 1**: chem70482‐sup‐0001‐SuppMat.pdf

## Data Availability

The data that support the findings of this study are available in the supplementary material of this article.

## Endnotes

1 We would like to point out that the term bismuth ylide complex in this work refers to a bismuth complex with a phosphorus ylide as ligand. This must not be confused with bismuth ylides of the general formula R_3_Bi = CR_2_.
