# Supplementary material for: Synthesis and Isolation of Bismuth Scorpionate Complexes: Toward Bond Activation via Bismuth‐Ligand Cooperation
Source: Chemistry. 2025 Nov 26;32(29):e02814. doi: 10.1002/chem.202502814 (PMC13451580; doi:10.1002/chem.202502814)
Supplement: Supplementary file 1 [file CHEM-32-e02814-s001.pdf]

Supporting Information

Synthesis and Isolation of Bismuth Scorpionate Complexes: Towards Bond Activation via Bismuth-Ligand Cooperation

Lennart Hensle,[a] Viktoria H. Gessner*[a]

[a] Faculty of Chemistry and Biochemistry, Ruhr-University Bochum, Universitätsstraße 150, 44801 Bochum, Germany, E-mail: viktoria.gessner@rub.de

Inhalt

1 General Experimental Details 2
2 Experimental Procedures 3
2.1 Synthesis of phosphonium salt $2_{H}I$ 3
2.2 Synthesis of phosphonium salt $2_{Ph}I$ 3
2.3 Synthesis of phosphonium salt $2_{Ar}FBr$ 4
2.4 Synthesis of zwitterionic compound 3_{H} 5
2.5 Synthesis of zwitterionic compound 3_{Ph} 6
2.6 Synthesis of ylide $3_{Ar}F$ 6
2.7 Synthesis of $4_{Ph}Cl$ 7
2.8 Synthesis of $4_{Ph}Br$ 8
2.9 Synthesis of $4_{Ph}I$ 9
2.10 Synthesis of $4_{H}Cl$ 10
2.11 Synthesis of $4_{Ar}FBr$ 11
2.12 Synthesis of $4_{Ar}FCl$ 12
2.13 Synthesis of $5_{Ph}OTf$ 13
2.14 Synthesis of $5_{Ph}BPh_4$ 14
2.15 Synthesis of bismuth yldiide 6 15
2.16 Synthesis of decomposition product 7 16
2.17 Synthesis of bismuth carbodiimide complex 8 17
2.18 General protocols for further reactivity studies of 6 18
3 Determination of the Lewis acidity via the Gutmann-Beckett method 19
4 NMR study on the decomposition of 6 to 7 21
5 NMR spectra of the isolated compounds 32
6 Single-crystal structure analyses 62
6.1 Data collection and structure refinement details 62
6.2 ORTEP plots of all crystal structures 66
7 References 80

1 General Experimental Details

Chemicals and conditions: If not stated otherwise, all experiments were carried out using standard Schlenk techniques under an argon atmosphere, which was dry and free of oxygen. Argon (99.999%) was a product of *Air Liquide* and was used without any further drying. An MBraun SPS 800 was used to dry solvents before their usage (THF, toluene, DCM, ACN, *n*-pentane, *n*-hexane). All solvents were stored over molecular sieves under an argon atmosphere. Commercial substrates were used as received unless stated otherwise. The phosphine **1** was prepared following the synthetic route published by Fryzuk.[1]

NMR spectroscopy: ^{1}H, $^{13}C\{^{1}H\}$ and $^{31}P\{^{1}H\}$ NMR spectra were recorded on an Avance-spectrometer at 25 °C if not stated otherwise. All values of the chemical shift are in ppm regarding the δ-scale. All spin-spin coupling constants (*J*) are printed in Hertz (Hz). To display multiplicities and signal forms correctly the following abbreviations were used: s = singlet, d = doublet, m = multiplet, br = broad signal. Signal assignment was supported by APT, HSQC, HMBC and COSY experiments.

X-Ray data collection of the compounds was conducted with an Oxford Synergy. The structures were solved using dual space FT and direct methods, refined with the Shelx software package and expanded using Fourier techniques.[2] The crystals of all compounds were mounted in an inert oil (perfluoropolyalkylether). Crystal structure determination was affected at 100 K. Crystallographic data (including structure factors) have been deposited with the Cambridge Crystallographic Data Centre as supplementary publication no. CCDC 2484196-2484209. For explicit assignment, see chapter 5.1. Copies of the data can be obtained free of charge on application to Cambridge Crystallographic Data Centre, 12 Union Road, Cambridge CB2 1EZ, UK; [fax: (+44) 1223-336-033; email: deposit@ccdc.cam.ac.uk].

Melting points were measured with the SMP30 melting point apparatus from Stuart.

Elemental analyses were performed in the in-house facility on an Elementar vario MICRO cube elemental analyzer.

HRMS-ESI: An LTQ Orbitrap Velos (Thermo Fisher Scientific, Bremen, Germany) was used for direct infusion via a syringe pump. The heated desolvation capillary was set to 200°C and a spray voltage of 1.8 kV was supplied. In the tune file the LTQ Orbitrap was set to the following parameters (R = 30,000; IT = 500 ms; AGC Target = 1,000,000).

HRMS-LIFDI: A JEOL AccuTof GCv (JMS-T100GCV) (JEOL, Tokyo, Japan) was equipped with a LIFDI source from Linden (CMS, Weyhe, Germany). The emitter heating current was set to 20 mA min^{-1} at a constant rate.

2 Experimental Procedures

2.1 Synthesis of phosphonium salt 2$_{H}$I

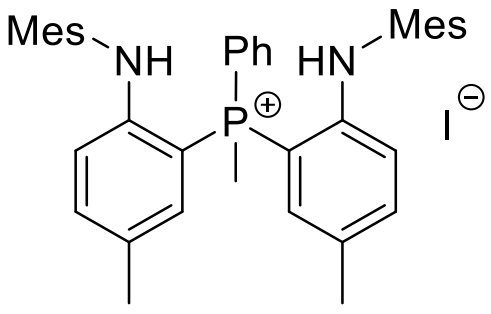

Phosphine **1** (707 mg, 1.27 mmol, 1.0 eq) and methyl iodide (182 mg, 1.27 mmol, 1.0 eq) were dissolved in dry toluene (6 mL) and the orange solution was stirred for 5 d at room temperature. The solvent was evaporated under reduced pressure and the remaining orange oil was washed three times with Et_2O (3x 5 mL). The resulting yellow solid was characterized as the targeted product.

Yield: 55 % (459 mg, 699 µmol).

^{1}H NMR (400 MHz, CD_2Cl_2) δ = δ 8.01 (dd, $^3J_{PH}$ = 13.7, $^3J_{HH}$ = 7.7 Hz, 2H, H13), 7.94 (t, $^3J_{HH}$ = 7.2 Hz, 1H, H15), 7.83 (ddd, $^3J_{HH}$ = 7.7, $^3J_{HH}$ = 7.7, $^4J_{PH}$ = 3.3 Hz, 2H, H14), 7.38 (d, $^3J_{HH}$ = 8.6 Hz, 2H, H5), 7.22 (dd, $^3J_{PH}$ = 16.1, $^4J_{HH}$ = 2.1 Hz, 2H, H7), 6.91 (s, 4H, H10), 6.45 (dd, $^3J_{HH}$ = 8.6, $^4J_{PH}$ = 6.5 Hz, 2H, H4), 5.11 (s, 2H, NH), 3.17 (d, $^2J_{PH}$ = 13.2 Hz, 2H, H1), 2.28 (s, 6H, H16), 2.26 (s, 6H, H18), 1.87 (s, 6H, H17), 1.84 (s, 6H, H17) ppm.

^{13}C{^{1}H} NMR (101 MHz, CD_2Cl_2) δ = 148.6 (d, $^3J_{PC}$ = 4.9 Hz, C3) 139.0 (d, $^4J_{PC}$ = 2.6 Hz, C8, 137.2 (s, C11), 136.3 (d, $^4J_{PC}$ = 3.0 Hz, C15), 135.0 (s, C9), 134.9 (s, C9), 134.3 (d, $^2J_{PC}$ = 10.4 Hz, C13), 134.1 (d, $^2J_{PC}$ = 11.0 Hz, C7), 134.1 (s, C5), 131.6 (d, $^3J_{PC}$ = 12.7 Hz, C14), 131.3 (d, $^3J_{PC}$ = 12.7 Hz, C6) 130.3 (s, C10) 130.2 (s, C10), 117.9 (d, $^1J_{PC}$ = 87.3 Hz, C12) 117.1 (d, $^3J_{PC}$ = 8.4 Hz, C4), 100.4 (d, $^1J_{PC}$ = 88.0 Hz, C2) 21.1 (s, C18), 20.7 (s, C16), 18.6 (C17), 18.5 (C17), 8.8 (d, $^1J_{PC}$ = 59.4 Hz, C1) ppm.

^{31}P{^{1}H} NMR (162 MHz, CD_2Cl_2) δ = 17.0 ppm.

ESI-HRMS: 698.2287 [M^+] calculated, 571.3237 [M^+ - I^-] measured.

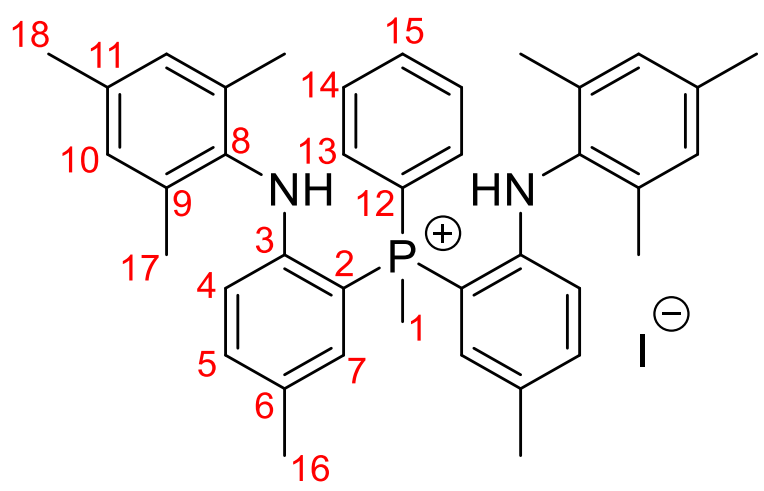

2.2 Synthesis of phosphonium salt 2$_{Ph}$I

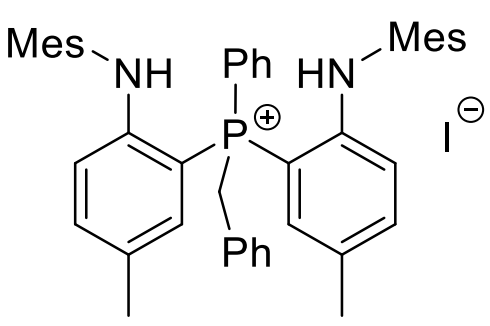

Phosphine **1** (1.13 g, 2.03 mmol, 1.0 eq) was dissolved in dry toluene (8 mL) and iodobenzene (444 mg, 2.03 mmol, 1.0 eq) was added. The orange solution was stirred over night at 80 °C. The next day the solvent was evaporated under reduced pressure and the remaining yellow solid was washed two times with Et_2O (2x 6 mL) and recrystallized in hot toluene (6 mL). The resulting colorless solid was characterized as the targeted product. Single crystals suitable for XRD analysis were obtained by slow diffusion of *n*-hexane into a concentrated THF solution of **2$_{Ph}$I**.

Yield: 64 % (997 mg, 1.31 mmol).

^{1}H NMR (400 MHz, CD_2Cl_2) δ = 7.80 (t, $^{3}J_{HH}$ = 7.6 Hz, 1H, H15), 7.66 (d, $^{3}J_{PH}$ = 14.2 Hz, 2H, H7), 7.53 (td, $^{3}J_{HH}$ = 7.8, 3.5 Hz, 2H, H14), 7.42 (d, $^{3}J_{HH}$ = 8.9 Hz, 2H, H5), 7.37 (dd, $^{3}J_{HH}$ = 6.9, $^{4}J_{HH}$ = 1.8 Hz, 1H, H19), 7.25 (m, 2H, H18), 7.20 (m, 2H, H13), 6.93 (dd, $^{3}J_{HH}$ = 7.8, $^{4}J_{HH}$ = 2.4 Hz, 2H, H17), 6.87 (s, 4H, H10), 6.46 (dd, $^{3}J_{HH}$ = 8.6, $^{4}J_{PH}$ = 6.4 Hz, 2H, H4), 5.30 (s, 2H, NH), 5.14 (d, $^{3}J_{HH}$ = 14.7 Hz, 2H, H1), 2.30 (s, 6H, C20-H), 2.24 (s, 6H, C22-H), 1.75 (s, 6H, C21-H), 1.69 (s, 6H, C21-H) ppm.

^{13}C{^{1}H} NMR (101 MHz, CD_2Cl_2) δ = 148.7 (d, $^{3}J_{PC}$ = 4.6 Hz, C3), 139.2 (d, $^{3}J_{PC}$ = 2.4 Hz, C15), 137.3 (s, C11), 136.2 (d, $^{4}J_{PC}$ = 3.1 Hz, C5), 135.2 (d, $^{3}J_{PC}$ = 9.6 Hz, C13), 134.9 (s, C9), 134.8 (s, C9), 134.3 (d, $^{2}J_{PC}$ = 8.5 Hz, C7), 133.9 (s, C8), 131.8 (d, $^{3}J_{PC}$ = 6.0 Hz, C17), 131.5 (d, $^{3}J_{PC}$ = 11.8 Hz, C6), 130.5 (d, $^{3}J_{PC}$ = 12.4 Hz, C14), 130.3 (s, C10), 130.2 (s, C10), 129.8 (d, $^{4}J_{PC}$ = 3.1 Hz, C18), 129.5 (d, $^{5}J_{PC}$ = 3.8 Hz, C19), 127.7 (d, $^{3}J_{PC}$ = 7.9 Hz, C16), 117.0 (d, $^{3}J_{PC}$ = 8.3 Hz, C4), 113.7 (d, $^{1}J_{PC}$ = 86.8 Hz, C12), 98.3 (d, $^{1}J_{PC}$ = 82.6 Hz, C2), 27.9 (d, $^{1}J_{PC}$ = 49.1 Hz, C1), 21.1 (s, C22), 20.7 (s, C20), 18.3 (s, C21), 18.1 (s, C21) ppm.

^{31}P{^{1}H} NMR (162 MHz, CD_2Cl_2) δ = 12.4 ppm.

Melting Point: 190 °C.

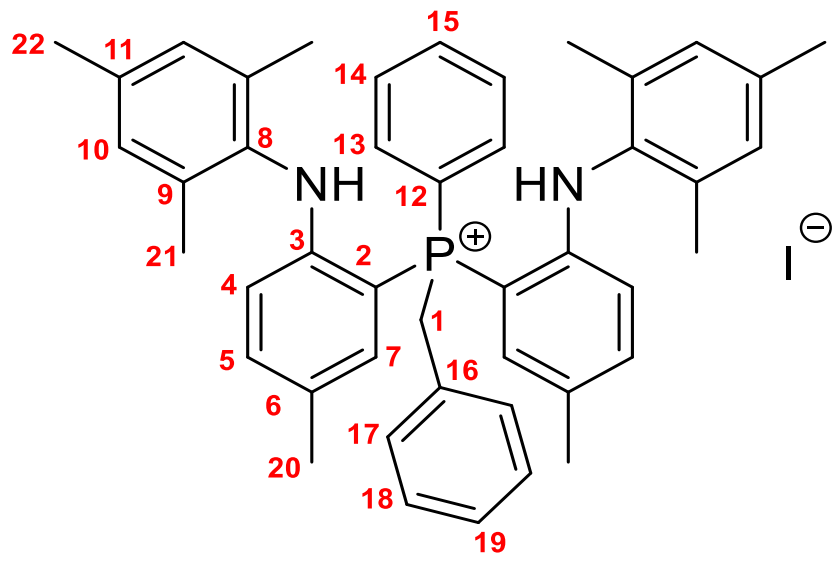

2.3 Synthesis of phosphonium salt 2_{ArF}Br

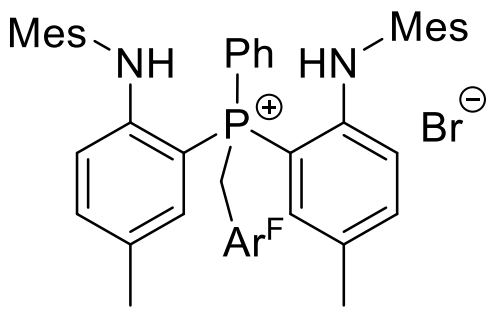

Phosphine **1** (2.88 g, 5.17 mmol, 1.00 eq) and 3,5-bis(trifluoromethyl)benzyl bromide (1.00 mL, 5.17 mmol, 1.00 eq) were dissolved in toluene (10 mL) and the yellow solution was stirred at 80 °C for 2 d. After that time the solution had become a yellow suspension. The precipitate was isolated from an orange solution by filtration. The beige solid was washed once with diethyl ether (4 mL) and the remaining colorless solid was characterized as the targeted product.

Yield: 72 % (3.22 g, 3.73 mmol)

^{1}H-NMR (400 MHz, CD_2Cl_2) δ = 7.86 (s, 1H, H19) 7.83 (d, $^{3}J_{PH}$ = 13.1 Hz, 2H, H7), 7.81 (m, 1H, H15), 7.72 (s, 2H, H17) 7.63 (m, 4H, H13 & H14), 7.39 (d, $^{3}J_{HH}$ = 8.6 Hz, 2H, H5), 6.89 (s, 2H, H10), 6.86 (s, 2H, H10), 6.43 (dd, $^{3}J_{HH}$ = 8.6, $^{4}J_{PH}$ = 6.6 Hz, 2H, H4), 6.05 (d, $^{2}J_{PH}$ = 15.0 Hz, 2H, H1), 5.55 (s, 2H, NH), 2.29 (s, 6H, H20), 2.25 (s, 6H, H22), 1.86 (s, 6H, H21), 1.65 (s, 6H, H21) ppm.

^{13}C{^{1}H}-NMR (101 MHz, CD_2Cl_2) δ = 148.5 (d, $^{2}J_{PC}$ = 4.8 Hz, C3), 139.0 (d, $^{4}J_{PC}$ = 2.6 Hz, C5), 137.1 (C8), 135.9 (d, $^{2}J_{PC}$ = 3.3 Hz, C15), 135.0 (d, $^{2}J_{PC}$ = 9.4 Hz, C7), 135.0 (C11), 134.8 (d, $^{2}J_{PC}$ = 10.0 Hz, C13), 133.9 (C9), 132.3 (d, $^{3}J_{PC}$ = 3.3 Hz, C17), 132.2 (C16), 131.9 (d, $^{4}J_{PC}$ = 3.4 Hz, C18) 131.2 (d, $^{1}J_{PC}$ = 12.1 Hz, C6), 130.6 (d, $^{3}J_{PC}$ = 12.5 Hz, C14), 130.1 (C10), 130.0 (C10), 125.6, 124.7 (C23), 122.5 (s, C19), 122.0 (C23), 116.5 (d, $^{3}J_{PC}$ = 8.4 Hz, C4), 115.2 (d, $^{1}J_{PC}$ = 85.4 Hz, C12), 97.6 (d, $^{1}J_{PC}$ = 84.0 Hz, C2), 26.9 (d, $^{1}J_{PC}$ = 50.0 Hz, C1), 20.9 (C22), 20.2 (C20), 18.4 (C21), 18.0 (C21) ppm.

^{19}F NMR (377 MHz, CD_2Cl_2) δ = –63.4 ppm.

^{31}P{^{1}H}-NMR (162 MHz, CD_2Cl_2) δ = 15.9 ppm.

Elemental Analysis: Calculated: C 65.36, H 5.37, N 3.24; Found: C 65.50, H 5.28, N 2.98

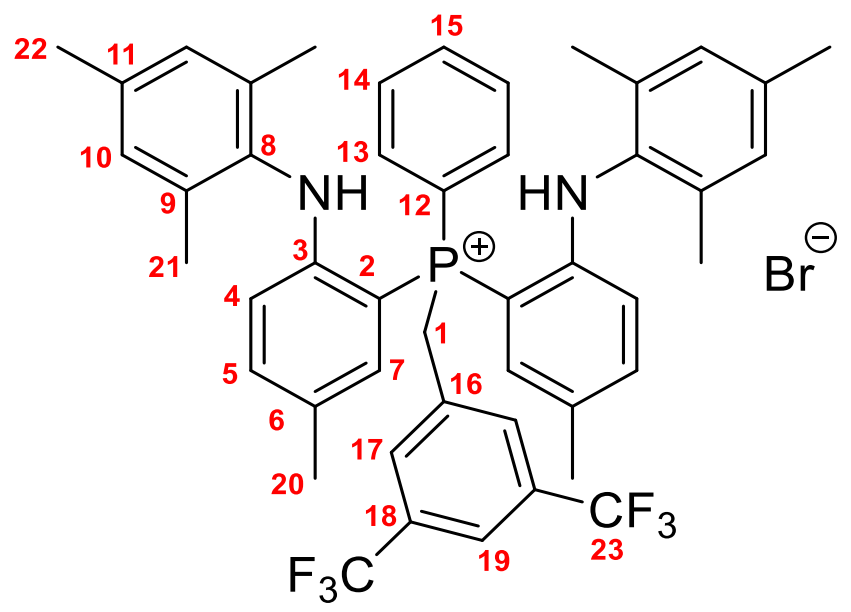

2.4 Synthesis of zwitterionic compound 3_H

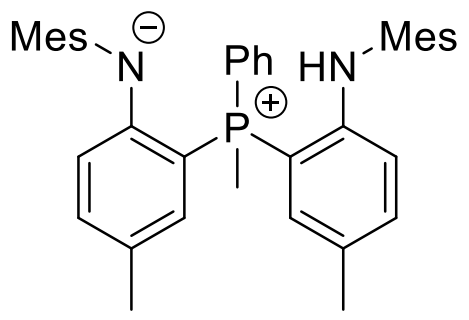

Methyl phosphonium salt 2_HI (400 mg, 573 µmol, 1.0 eq) and KH (23.0 mg, 573 µmol, 1.0 eq) were dissolved in dry THF (8 mL) and the orange solution was stirred over night at RT. The solution was separated from the precipitate that had formed and the solvent was evaporated. The remaining solid was washed one time with a small amount of hexane (1 mL) and the remaining orange powder was characterized as the product. Single crystals suitable for XRD analysis were obtained by slow evaporation of a saturated *n*-hexane solution of **3_H**.

Yield: 51 % (167 mg, 293 µmol).

^{1}H NMR (400 MHz, CD_2Cl_2) δ = 7.81 (dd, $^{3}J_{PH}$ = 12.9, $^{3}J_{HH}$ = 7.6 Hz, 2H, H13), 7.65 (m, 1H, H15), 7.57 (ddd, $^{3}J_{HH}$ = 7.8, $^{3}J_{HH}$ = 7.8, $^{4}J_{PH}$ = 3.9 Hz, 2H, H14), 6.90 (d, $^{3}J_{HH}$ = 8.8 Hz, 2H, H5), 6.78 (s, 4H, H10), 6.76 (d, $^{3}J_{PH}$ = 14.4 Hz, 2H, H7), 5.97 (dd, $^{3}J_{HH}$ = 8.8, $^{4}J_{PH}$ = Hz, 2H, H4), 5.76 (bs, 1H, N-H), 2.84 (bs, 3H, H1), 2.21 (s, 6H, H18), 2.06 (s, 6H, H16), 1.87 (s, 6H, H17), 1.82 (s, 6H, H17) ppm.

^{13}C{^{1}H} NMR (101 MHz, CD_2Cl_2) δ = 153.1 (d, $^{2}J_{PC}$ = 4.9 Hz, C3), 143.1 (s, C8), 136.3 (C5), 134.0 (d, $^{3}J_{PC}$ = 10.9 Hz, C14), 133.52 (d, $^{3}J_{PH}$ = 10.0 Hz, C13), 133.2 (s, C11), 133.1 (d, $^{3}J_{PC}$ = 3.9 Hz), 132.1 (C9), 129.7 (d, $^{4}J_{PC}$ = 12.3 Hz, C4), 129.3 (s, C10), 124.2 (d, $^{1}J_{PC}$ = 65.9 Hz, C12) 121.5 (d, $^{1}J_{PC}$ = 13.3 Hz, C2), 96.0 (s, C6), 21.0(s, C18), 20.5 (s, C16), 18.9 (s, C17), 18.8 (s, C17), 9.8 (d, $^{1}J_{PC}$ = 65.1 Hz, C15) ppm.

^{31}P{^{1}H} NMR (162 MHz, CD_2Cl_2) δ = 11.8 ppm.

ESI-HRMS: 570.3164 [M^+] calculated, 571.3236 [$M^+ + H^+$] measured.

Melting Point: 180 °C.

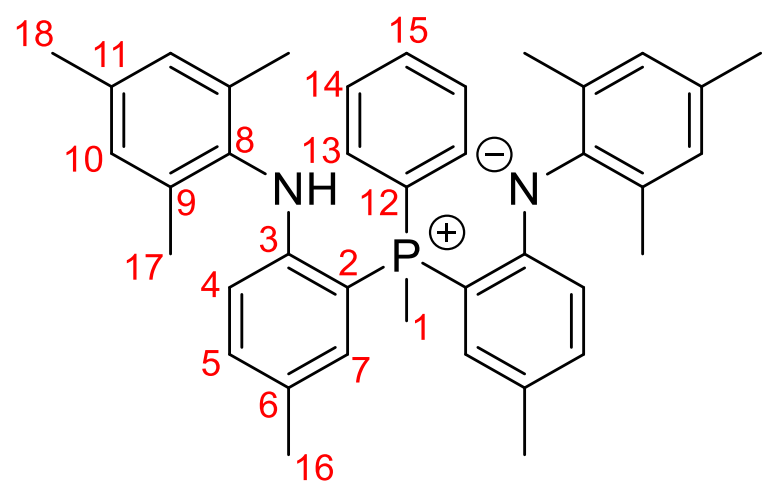

2.5 Synthesis of zwitterionic compound **3_{Ph}**

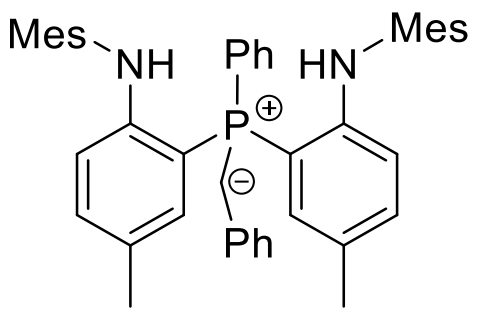

Phosphonium salt **2_{Ph}I** (700 mg, 903 µmol, 1.0 eq) and KH (36.2 mg, 903 µmol, 1.0 eq) were dissolved in dry THF (20 mL). The resulting orange solution was stirred overnight at RT, resulting in a change from a clear solution to a suspension. The liquid phase was separated from the precipitate and the solvent of the solution was evaporated under reduced pressure. The obtained orange solid was washed one time with *n*-hexane (4 mL), giving the product as a yellow powder. Single crystals suitable for XRD analysis were obtained by slow evaporation of a saturated *n*-hexane solution of **3_{Ph}**.

Yield: 84 % (490 mg, 758 µmol).

^{1}H NMR (400 MHz, CD_2Cl_2) δ = 7.53 (dt, $^3J_{HH}$ = 7.2, $^4J_{HH}$ = 1.7 Hz, 1H, H15), 7.30 (dt, $^3J_{HH}$ = 7.7, $^3J_{HH}$ = 4.0 Hz, 2H, H14), 7.24 (dd, $^3J_{PH}$ = 11.3, $^3J_{HH}$ = 7.8 Hz, 2H, H13), 7.15 (m, 3H, H18 & H19), 7.04 (m, 4H, H7 & H17), 6.95 (dd, $^3J_{HH}$ = 8.7, $^4J_{PH}$ = 2.1 Hz, 2H, H5), 6.78 (s, 2H, H10), 6.76 (s, 2H, H10), 6.02 (dd, $^3J_{HH}$ = 8.7, $^4J_{PH}$ = 6.2 Hz, 2H, H4), 5.40 (m, 3H, H1 & NH), 2.20 (s, 6H, H22), 2.09 (s, 6H, H20), 1.85 (s, 6H, H21), 1.73 (s, 6H, H21) ppm.

^{13}C{^{1}H} NMR (101 MHz, CD_2Cl_2) δ = 152.8 (d, $^3J_{PC}$ = 7.1 Hz, C3), 142.13 (s, C8), 135.94 (d, $^4J_{PC}$ = 2.1 Hz, C5), 134.67 (d, $^3J_{PC}$ = 8.7 Hz, C13), 134.3 (d, $^3J_{PC}$ = 9.4 Hz, C7), 133.8 (C16), 133.4 (C11), 132.90 (d, $^4J_{PC}$ = 2.9 Hz, C15), 132.57 (s, C9), 130.3 (d, $^3J_{PC}$ = 7.0 Hz, C17), 129.3 (s, C10), 129.28 (s, C10), 128.9 (d, $^4J_{PC}$ = 2.3 Hz, C18), 128.8 (d, $^3J_{PC}$ = 11.6 Hz, C14), 126.1 (s, C19), 121.8 (m, C12 & C2), 114.8 (d, $^3J_{PC}$ = 8.5 Hz, C4), 27.7 (d, $^3J_{PC}$ = 899.4 Hz, C1) 21.0 (s, C22), 20.62 (C20), 18.67 (C21), 18.62 (C21) ppm.

^{31}P{^{1}H} NMR (162 MHz, CD_2Cl_2) δ = 10.1 ppm.

ESI-HRMS: 646.3477 [M^+] calculated, 647.3552 [$M^+ + H^+$] measured.

Melting Point: 186 °C.

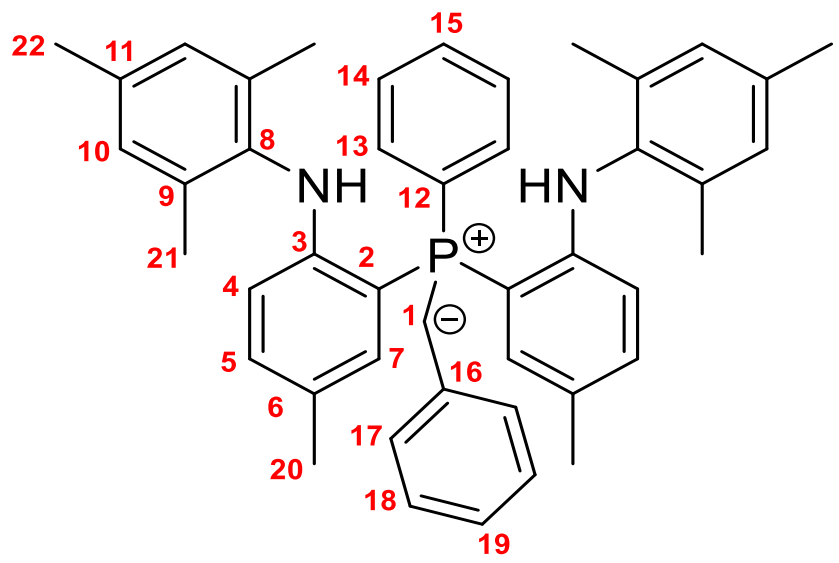

2.6 Synthesis of ylide **3_{ArF}**

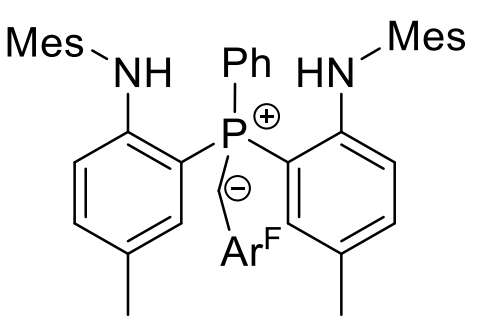

Phosphonium salt **2_{ArF}Br** (46.9 mg, 54.3 µmol, 1.00 eq) and LDA (6.0 mg, 54.3 µmol, 1.00 eq) were weight into a J-Young NMR tube and Et_2O (0.5 mL) was added. Within seconds the colorless suspension turned into a clear yellow solution. The tube was shaken for 1 d at RT, then the solvent was evaporated. The residual yellow solid was taken up in hexane (2 mL) and insoluble fractions were removed via filtration. The solvent of the yellow solution was evaporated, and the product was isolated as a yellow solid. Single crystals suitable for XRD analysis were obtained by slow evaporation of a saturated *n*-pentane solution of **3_{ArF}**.

Yield: 71 % (30.8 mg, 38.4 µmol)

^{1}H NMR (400 MHz, CD_2Cl_2) δ = 7.79 (dd, $^3J_{PH}$ = 12.7, $^3J_{HH}$ = 7.4 Hz, 2H, H13), 7.60 (td, $^3J_{HH}$ = 7.4, $^4J_{HH}$ = 1.7 Hz, 1H, H15), 7.50 (td, $^3J_{HH}$ = 7.7, $^4J_{PH}$ = 2.8 Hz, 2H, H14), 7.29 (d, $^3J_{PH}$ = 15.7 Hz, 2H, H7), 7.12 (d, $^3J_{HH}$ = 8.5 Hz, 2H, H5), 6.97 (s, 2H, H17), 6.86 (d, J = 2.0 Hz, 2H, H10), 6.80 (d, J = 2.1 Hz, 2H, H10), 6.72 (s, 1H, H19), 6.12 (dd, $^3J_{HH}$ = 8.4, $^4J_{PH}$ = 5.8 Hz, 2H, H5), 2.23 (s, 6H, H22), 2.19 (s, 6H, H20), 1.94 (s, 6H, H21), 1.57 (s, 6H, H21) ppm.

^{13}C NMR (101 MHz, CD_2Cl_2) δ 149.2 (d, $^2J_{PC}$ = 7.7 Hz, C3), 148.6 (C8), 136.4 (C9), 136.2 (C11), 136.1 (C9), 135.4 (d, $^5J_{PC}$ = 2.4 Hz, C5), 135.2 (d, $^3J_{PC}$ = 10.6 Hz, C7), 133.2 (d, $^3J_{PC}$ = 10.2 Hz, C13), 132.5 (d, $^5J_{PC}$ = 2.8 Hz, C15), 131.3 (d, $^4J_{PC}$ = 2.1 Hz, C18), 131.0 (d, $^4J_{PC}$ = 2.2 Hz, C18), 129.6 (C14), 129.5 (C10), 129.4 (C10), 128.2 ($^1J_{PC}$ = 114.7 Hz, C12), 127.1 (d, $^3J_{PC}$ = 12.1 Hz, C6), 124.8 (d, 272.3 Hz, C16), 119.5 (C17),* 113.3 (d, $^4J_{PC}$ = 7.8 Hz, C4), 106.8 (C19), 105.2 ($^1J_{PC}$ = 94.4 Hz, C2),* 21.0 (C22), 20.5 (C20), 18.4 (C21), 17.7 (C21) ppm.

*Determined per ^{1}H-^{13}C-HMBC-experiment

C1 and C23 could not be found

^{19}F NMR (377 MHz, CD_2Cl_2) δ = –64.0 ppm.

^{31}P NMR (162 MHz, CD_2Cl_2) δ = 2.5 ppm.

LIFDI-HRMS: 782.3225 [M^+] calculated, 782.3228 [M^+] measured.

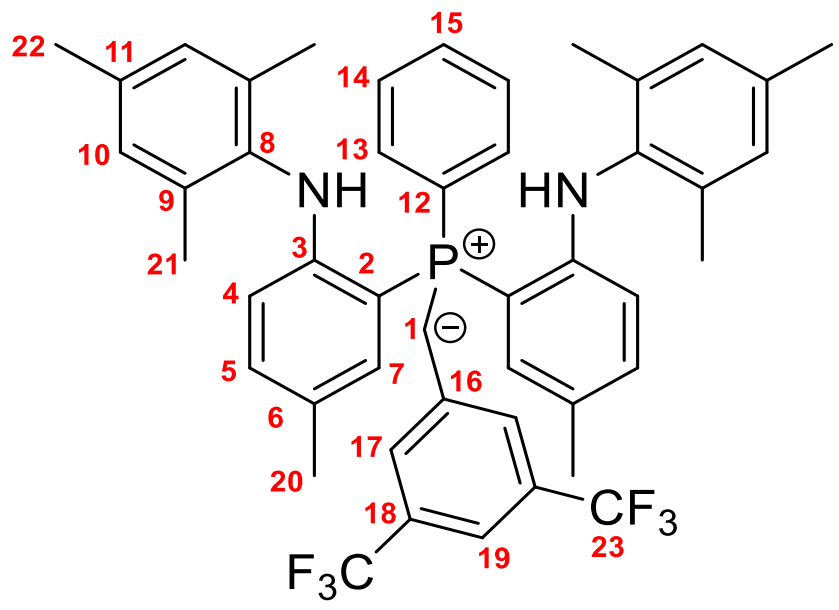

2.7 Synthesis of 4_{Ph}Cl

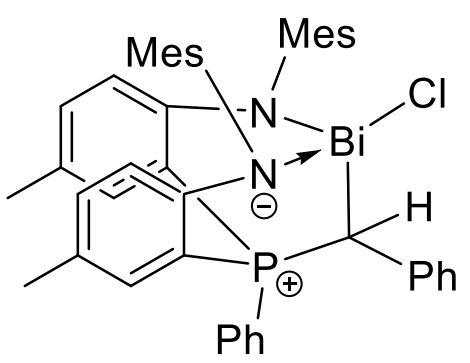

Zwitterionic compound **3_{Ph}** (250 mg, 386 µmol, 1 eq) was dissolved in dry THF (4 mL) in a Schlenk tube and *n*-BuLi (1.52 M, 508 µL, 772 µmol, 2 eq) was added at -78 °C. The dark red solution was allowed to warm up to RT and the mixture was stirred for 5 h. Next the solution of deprotonated **4** was added to a solution of $BiCl_3$ (122 mg, 386 µmol, 1 eq), which was purified by sublimation, in THF (3 mL) at -78 °C. This led to a color change of the solution to brown. The solution was stirred for 1 d, then the solution was filtered, which led to the separation of an orange solution from a small amount of black solid. The solvent of the solution was evaporated and the remaining orange solid was dissolved in toluene (10 mL) to separate the product from LiCl residues after filtration. The solvent of the filtrate was evaporated and the remaining orange solid was characterized as the targeted product.

Yield: 85 % (291 mg, 327 µmol)

^{1}H NMR (400 MHz, THF-d_8) δ = 7.59 (td, $^3J_{HH}$ = 7.3, $^4J_{HH}$ = 1.6 Hz, 1H, H-15), 7.47 (ddd, $^3J_{HH}$ = 7.8, $^3J_{HH}$ = 7.8, $^4J_{HP}$ = 3.2 Hz, 2H, H14), 7.24 (t, $^3J_{HH}$ = 7.6 Hz, 2H, H18), 7.17 (dd, $^3J_{HH}$ = 7.3, $^4J_{HH}$ = 1.3 Hz, 2H, H13), 7.15 (m, 1H, H-1), 7.11 (m, 1H, H5), 7.09 (m, 1H, H-19), 7.07 (m, 2H, H-3) 7.01 (dd, $^3J_{HH}$ = 6.0, $^4J_{HH}$ = 2.0 Hz, 1H, H5), 6.96 (m, 2H, H-17), 6.95 (s, H-10), 6.92 (s, 1H, H-10), 6.78 (s, 1H, H-10), 6.76 (s, 1H, H-10), 6.24 (dd, $^4J_{HP}$ = 8.8, $^3J_{HH}$ = 7.0 Hz, 1H, H4),

5.99 (dd, $^4J_{HP}$ = 8.7, $^3J_{HH}$ = 6.7 Hz, 1H, H4), 3.64 (d, $^2J_{PH}$ = 9.5 Hz, 1H, H1), 2.30 (s, 3H, H21), 2.29 (s, 3H, H21), 2.25 (s, 3H, H22), 2.24 (s, 3H, H22), 2.17 (s, 3H, H20), 2.14 (s, 3H, H20), 1.67 (s, 3H, H21), 1.28 (s, 3H, H21) ppm.

$^{13}C\{^1H\}$ NMR (101 MHz, THF-d$_8$) δ = 159.0 (C8), 157.8 (d, $^2J_{CP}$ = 5.5 Hz, C7), 148.3 (C11), 142.8 (C11),* 137.2 (C9), 136.9 (d, $^4J_{CP}$ = 2.2 Hz, C5), 136.0 (d, $^4J_{CP}$ = 2.9 Hz, C15), 135.8 (C9), 135.2 (C9), 135.0 (d, $^2J_{CP}$ = 8.1 Hz, C13), 134.4 (d, $^2J_{CP}$ = 9.1 Hz, C3), 134.3 (C9), 134.2 (d, $^4J_{CP}$ = 2.9 Hz, C5), 133.0 (d, $^2J_{CP}$ = 8.8 Hz, C3), 132.76 (d, $^3J_{CP}$ = 6.2 Hz, C17), 131.2 (C10), 130.9 (C10), 130.7 (C10), 130.0 (C10), 129.9 (d, $^3J_{CP}$ = 11.4 Hz, C14), 129.6 ppm (d, $^3J_{CP}$ = 54.9 ppm, C1),* 129.1 (m, C18), 128.4 (C19), 126.2 (d, $^3J_{CP}$ = 13.2 ppm, C4), 123.1 (d, $^4J_{CP}$ = 16.4 Hz, C4),* 122.9 (m, C12) 118.76 (d, $^4J_{CP}$ = 9.9 Hz, C6), 116.43 (d, $^4J_{CP}$ = 8.8 Hz, C6), 110.45 (d, $^1J_{CP}$ = 91.3 Hz, C2), 100.6 (d, $^1J_{CP}$ = 101.6 Hz, C2),* 21.2 (C22), 21.1 (C22), 20.70 (C21), 20.4 (C20), 19.9 (C20), 19.8 (C21), 17.9 (C21), 17.7 (C21) ppm.

*Found via ^{1}H-^{13}C-HSQC or ^{1}H-^{13}C HMBC NMR experiment

Remark: C16 could not be assigned

$^{31}P\{^1H\}$ NMR (162 MHz, THF-d$_8$) δ = 9.6 ppm.

Elemental Analysis: Calculated: C 60.78, H 5.10, N 3.15; Found: C 60.63, H 5.26, N 2.71

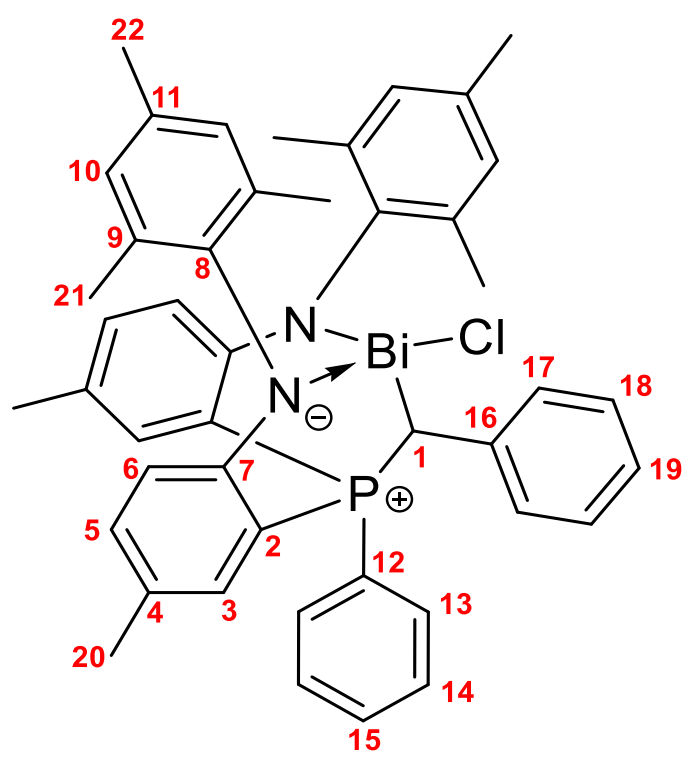

2.8 Synthesis of 4$_{Ph}$Br

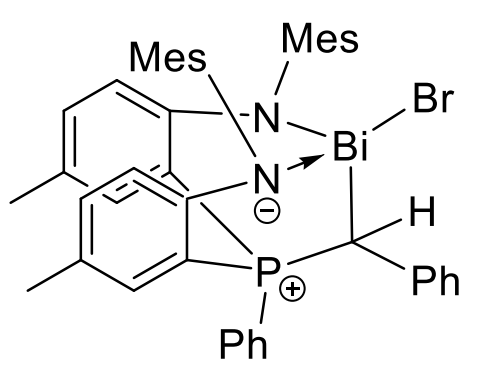

Phosphonium salt **2$_{Ph}$I** (372 mg, 480 µmol, 1.00 eq) and KH (19.3 mg, 480 µmol, 1.00 eq) were dissolved in THF (4 mL) inside a Schlenk tube and the yellow suspension was stirred for 1 d at RT. The next day the orange solution was separated by filtration from a grey precipitate and transferred into a new Schlenk tube where a solution of *n*-BuLi in *n*-hexane (1.6 M, 0.6 mL, 960 µmol, 2.00 eq) was added at -78 °C. The dark red solution was allowed to warm up to RT and the mixture was stirred for 2.5 h. Next the solution of deprotonated **2$_{Ph}$I** was added to a solution of sublimed $BiBr_3$ (218 mg, 480 µmol, 1.00 eq) in THF (3 mL) at -78 °C, which led to a color change of the solution to black. The solution was stirred for 4.5 h at RT. Then the solvent was evaporated, and the dark brown residue was dissolved in toluene (5 mL). The solution was isolated by filtration and the solvent was evaporated. The orange residue was washed with cold pentane (4 mL). The isolated orange solid was characterized as the targeted product. Single crystals of **4$_{Ph}$Br** suitable for XRD analysis were obtained by slow diffusion of *n*-hexane into a concentrated toluene solution.

Yield: 49 % (220 mg, 236 µmol)

^{1}H NMR (400 MHz, THF-d$_8$) δ = 7.60 (dt, $^{3}J_{HH}$ = 7.5, $^{4}J_{HH}$ = 2.5 Hz, 1H, H15), 7.48 (td, $^{3}J_{HH}$ = 7.8, $^{4}J_{PH}$ = 3.3 Hz, 2H, H14), 7.24 (t, $^{3}J_{HH}$ = 7.7 Hz, 2H, H18), 7.16 (m, 2H, H13), 7.12 (m, 1H, H5), 7.10 (m, 1H, H7), 7.09 (m, 1H, H19), 7.08 (m, 1H, H5), 7.04 (s, 1H, H7), 6.96 (m, 3H, H10 & H17), 6.90 (s, 1H, H10), 6.78 (s, 1H, H10), 6.75 (s, 1H, H10), 6.23 (m, 1H, H4), 6.01 (dd, $^{4}J_{PH}$ = 8.7, $^{3}J_{HH}$ = 6.4 Hz, 1H, H4), 3.74 (d, $^{2}J_{PH}$ = 9.6 Hz, 1H, H1), 2.30 (s, 3H, H21), 2.29 (s, 3H, H21), 2.25 (s, 3H, H22), 2.24 (s, 3H, H22), 2.17 (s, 3H, H20), 2.14 (s, 3H, H20), 1.67 (s, 3H, H21), 1.28 (s, 3H, H21) ppm.

^{13}C NMR (101 MHz, THF-d$_8$) δ = 158.7 (d, $^{2}J_{PC}$ = 6.2 Hz, C3), 157.7 (d, $^{2}J_{PC}$ = 5.5 Hz, C3), 148.1 (C8), 143.0 (C8), 139.2 (C11), 137.2 (C11), 136.9 (C5), 136.2 (C5), 136.0 (d, 2.4 Hz, C19), 135.7 (C9), 135.0 (C9), 134.9 (d, $^{2}J_{PC}$ = 8.1 Hz, C13), 134.5 (C9), 134.4 (C15), 134.3 (d, $^{3}J_{PC}$ = 2.7 Hz, C7), 132.9 (d, $^{3}J_{PC}$ = 8.8 Hz, C7), 132.8 (d, J = 6.2 Hz, C17), 131.2 (C10), 130.9 (C10), 130.7 (C10), 130.0 (C10), 129.9 (d, $^{3}J_{PC}$ = 11.4 Hz, C14), 129.0 (d, $^{4}J_{PC}$ = 2.6 Hz, C18), 126.3 (d, $^{3}J_{PC}$ = 12.9 Hz, C6), 123.4 (d, , $^{3}J_{PC}$ = 12.9 Hz, C6), 122.7 (d, , $^{1}J_{PC}$ = 82.9 Hz, C12), 118.8 (d, $^{3}J_{PC}$ = 9.9 Hz, C4), 116.4 (d, $^{3}J_{PC}$ = 8.8 Hz, C4), 110.3 (d, $^{1}J_{PC}$ = 91.0 Hz, C2), 101.0 (d, $^{1}J_{PC}$ = 93.2 Hz, C2), 68.5 (d, $^{1}J_{PC}$ = 47.5 Hz, C1), 21.1 (C22), 21.0 (C22), 20.8 (C21), 20.3 (C20), 19.9 (C20), 19.8 (C21), 17.7 (C21), 17.6 (C21).

Remark: C16 could not be assigned

^{31}P NMR (162 MHz, THF-d$_8$) δ = 9.5 ppm.

Elemental Analysis: Calculated: C 57.89, H 4.86, N 3.00; Found: C 57.28, H 4.43, N 3.01

LIFDI-HRMS: 932.2308 [M^{+}] calculated, 932.2326 [M^{+}] measured.

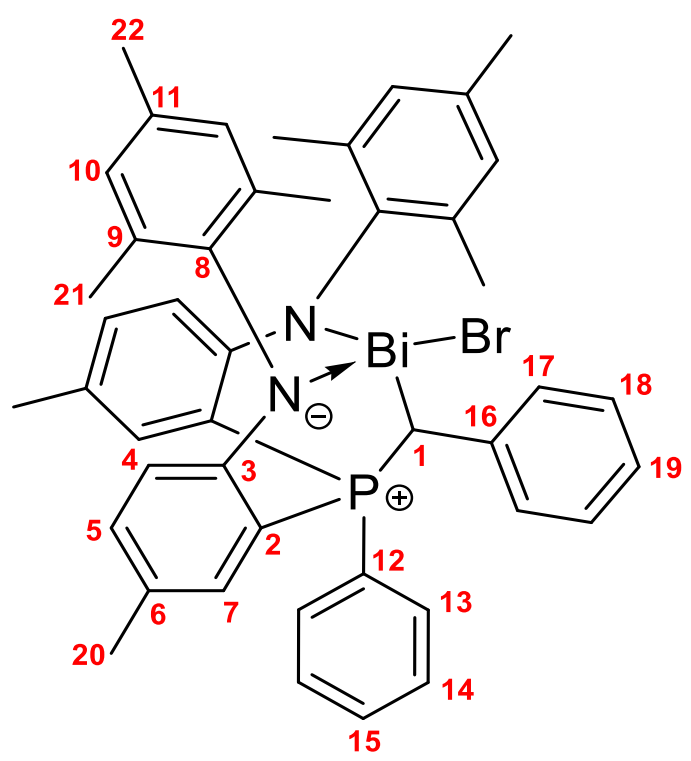

2.9 Synthesis of 4$_{Ph}$I

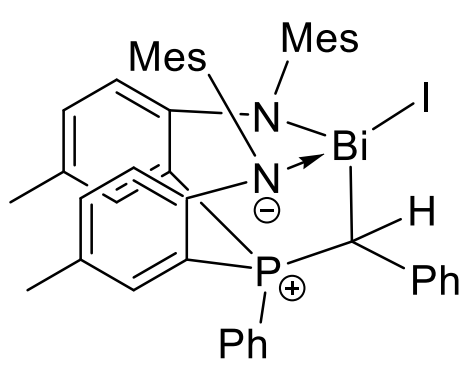

2$_{Ph}$I (1.24 g, 1.60 mmol, 1.00 eq) was dissolved in THF (10 mL) in a Schlenk tube and a solution of *n*-BuLi in *n*-hexane (1.6 M, 3 mL, 4.80 mmol, 3.00 eq) was added at -78 °C. The dark red solution was allowed to warm to RT and the mixture was stirred for 2 h. Next, that solution was added to a solution of $BiCl_3$ (505 mg, 1.60 mmol, 1.00 eq) in THF (5 mL) at -78 °C, which led to a color change to black. The solution was stirred for 1 d at RT. The solution was filtered to separate the dark brown solution from the black solid. The solvent of the solution was evaporated and the remaining solid was washed twice with *n*-hexane (5 ml), giving the product as orange solid. Single crystals suitable for XRD analysis were obtained by slow evaporation of a concentrated benzene solution of **4$_{Ph}$I**.

Yield: 81 % (1.27 g, 1.30 mmol)

^{1}H NMR (400 MHz, THF) δ = 7.64 (m, 1H, H15), 7.48 (td, $^{3}J_{HH}$ = 7.8, $^{4}J_{HH}$ = 3.2 Hz, 2H, H14), 7.25 (t, $^{3}J_{HH}$ = 7.6 Hz, 2H, H18), 7.15 (m, 2H, H13), 7.05 (m, 5H, H3 & H5 & H19), 6.98 (s, 1H, H10), 6.93 (s, 1H, H10), 6.91 (m, 2H, H17), 6.79 (s, 1H, H10), 6.77 (s, 1H, H10), 6.24 (dd, $^{3}J_{HH}$ = 8.7, $^{4}J_{PH}$ = 7.0 Hz, 1H, H4), 6.03 (dd, $^{3}J_{HH}$ = 8.7, $^{4}J_{PH}$ = 6.7 Hz, 1H, H4), 3.87 (d, $^{2}J_{PH}$ = 9.8 Hz, 1H, H1), 2.29 (s, 3H, H21), 2.28 (s, 3H, H21), 2.26 (s, 3H, H22), 2.25 (s, 3H, H22), 2.18 (s, 3H, H20), 2.15 (s, 3H, H20), 1.68 (s, 3H, H21), 1.29 (s, 3H, H21) ppm.

^{13}C{^{1}H} NMR (101 MHz, THF-d$_8$) δ = 158.3 (C7)*, 148.1 (C11), 143.9 (C11), 137.3 (C9), 136.7 (d, 1.8 Hz, C3), 136.0 (d, 2.5 Hz, C3), 135.9 (C9), 135.1 (C9), 135.0 (d, 8.0 Hz, C13), 134.5 (C9), 134.4 (C15), 134.3 (d, 2.8 Hz, C5), 133.0 (d, 4.6 Hz, C5), 132.9 (C17), 131.6 (C10), 131.0 (C10), 130.8 (C10), 130.1 (C10), 129.9 (d, 11.1 Hz, C14), 129.0 (d, 2.6 Hz, C18), 128.7 (d, 2.3 Hz, C15), 126.5 (d, 12.9 Hz, C4), 123.9 (C4), 122.6 (C12)*, 118.9 (d, 10.6 Hz, C6), 116.4 (d, 8.2 Hz, C6), 110.2 (C2)*,101.7 (C2)*, 63.9 (d, 34.5 Hz, C1)*, 21.2 (C22), 21.1 (C22), 21.0 (C21), 20.3 (C20), 19.9 (C20), 19.7 (C21), 17.6 (C21), 17.5 (C21) ppm.

*Found via ^{1}H-^{13}C-HSQC or ^{1}H-^{13}C HMBC NMR experiment

Remark: C8 and C16 could not be assigned

^{31}P{^{1}H} NMR (162 MHz, THF-d$_8$) δ = 9.6 ppm.

LIFDI-HRMS: 980.2169 [M$^+$] calculated, 980.2147 [M$^+$] measured.

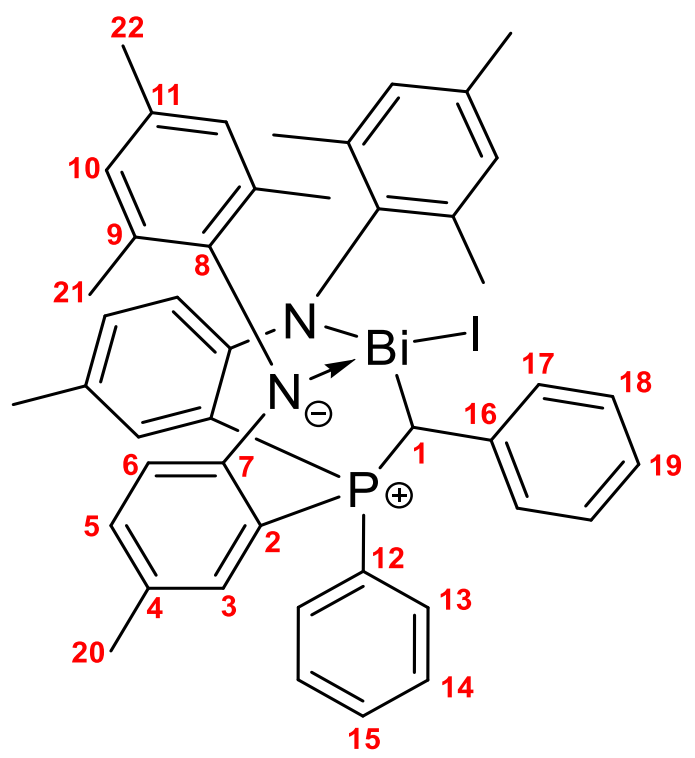

2.10 Synthesis of 4$_H$Cl

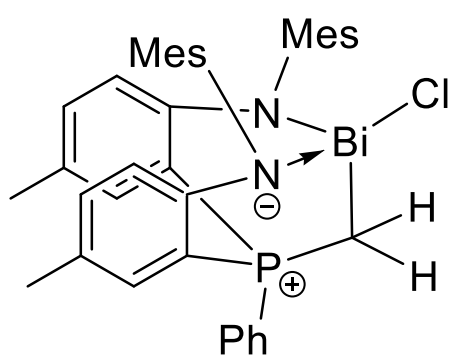

Phosphonium salt **2$_H$I** (168 mg, 240 µmol, 1 eq) and KH (10.1 mg, 252 µmol, 1.05 eq) were dissolved in dry THF (2.5 mL) in a Schlenk tube and the orange suspension was stirred for 1 d at RT. Next, an orange solution was separated via filtration from a yellow precipitate. To that solution *n*-BuLi (1.3 M, 370 µL, 480 µmol, 2 eq) was added at -78 °C. The dark red solution was allowed to warm up to RT and the mixture was stirred for 3 h. Then the solution was added to a solution of $BiCl_3$ (75.8 mg, 240 µmol, 1 eq) in THF (2 mL) at -78 °C. This led to a color change of the solution to brown. The solution was stirred for 1 d, then the solvent was evaporated, and the residual brown solid was extracted with toluene (3 mL). The solvent of the isolated toluene solution was evaporated, and the residual orange solid was washed three times with hexane (3x 1.5 mL). The remaining yellow solid was characterized as the targeted product. Single crystals suitable for XRD analysis were obtained by slow diffusion of *n*-hexane into a concentrated toluene solution of **4$_{Ph}$Br**.

Yield: 49 % (95.6 mg, 118 µmol)

^{1}H NMR (400 MHz, CD_2Cl_2) δ = 7.86 (ddd, $^{3}J_{PH}$ = 12.1, $^{3}J_{HH}$ = 7.5, $^{4}J_{HH}$ = 1.9 Hz, 2H, H13), 7.69 (m, 3H, H14 & H15), 7.08 (m, 4H, H5 & H7), 6.94 (d, $^{4}J_{HH}$ = 2.1 Hz, 2H, H10), 6.76 (d, $^{4}J_{HH}$ = 2.2 Hz, 2H), 6.08 (dd, $^{4}J_{PH}$ = 9.0, $^{3}J_{HH}$ =7.0 Hz, 2H, H4), 2.51 (d, $^{2}J_{PH}$ = 11.1 Hz, 2H, H1), 2.26 (s, 6H, H18), 2.24 (s, 6H, H21), 2.23 (s, 6H, H16), 1.31 (s, 6H, H17) ppm.

^{13}C NMR (101 MHz, CD_2Cl_2) δ = 157.7 (C3), 144.3 (C8), 136.8 (C11), 135.8 (C5), 135.7 (C9), 135.1 (C9), 134.3 (d, $^{1}J_{PC}$ = 136.9 Hz, C12), 132.5 (d, $^{2}J_{PC}$ = 9.3 Hz, C7), 130.1 (C10), 130.0 (C10), 125.0 (d, $^{3}J_{PC}$ = 13.2 Hz, C6), 117.8 (d, $^{3}J_{PC}$ = 9.8 Hz, C4), 105.6 (d, $^{1}J_{PC}$ = 93.5 Hz, C2), 54.1 (C1)*, 20.9 (C18), 20.0 (C16), 19.7 (C17), 17.1 (C17) ppm.

*Found via ^{1}H-^{13}C HSQC or ^{1}H-^{13}C HMBC NMR experiment

^{31}P NMR (162 MHz, CD_2Cl_2) δ = 3.0 ppm.

LIFDI-HRMS: 812.2500 [M^+] calculated, 812.2518 [M^+] measured.

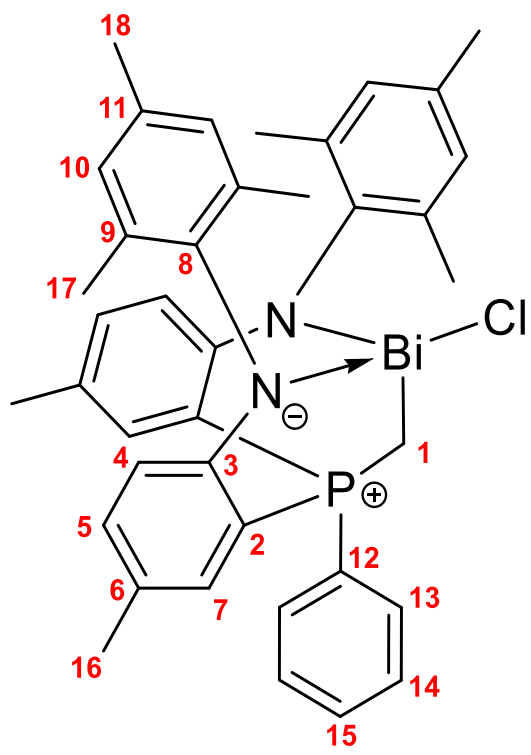

2.11 Synthesis of 4_{ArF}Br

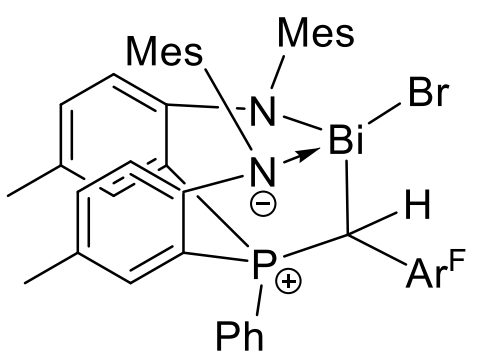

Phosphonium salt **2_{ArF}Br** (921 mg, 1.07 mmol, 1.00 eq) was dissolved in THF (10 mL) in a Schlenk tube and a solution of *n*-BuLi in *n*-hexane (1.6 M, 2.00 mL, 3.20 mmol, 3 eq) was added at -78 °C. The dark red solution was allowed to warm up to RT and the mixture was stirred for 2.5 h at that temperature. Next this solution was added to a solution of $BiCl_3$ (336 mg, 1.07 mmol, 1.00 eq) in THF (5 mL) at -78 °C. The resulting black solution was stirred for 4.5 h at RT. Then the solvent was evaporated and the dark brown residue was dissolved in toluene (8 mL). The solution was isolated by filtration and the solvent was evaporated. The orange residue was washed with cold pentane (15 mL) and the remaining orange powder was characterized as the targeted product. Single crystals suitable for XRD analysis were obtained by slow diffusion of *n*-hexane into a concentrated toluene solution of **4_{ArF}Br**.

Yield: 61 % (693 mg, 648 μmol)

^{1}H-NMR (400 MHz, CD_2Cl_2) δ = 7.65 (s, 1H, H19), 7.64 (d, J = 8.5 Hz, 1H, H15), 7.49 (td, J = 7.7, 3.3 Hz, 2H, H14), 7.38 (s, 2H, H17), 7.19 (d, 8.5 Hz, 1H, H5), 7.17 (d, 8.1 Hz, 1H, H5), 7.12 (dd, J = 13.8, 2.1 Hz, 1H, H7), 7.07 (m, 2H, H13), 7.03 (m, 1H, H7), 7.02 (s, 1H, H10), 6.98 (s, 1H, H10), 6.85 (s, 1H, H10), 6.83 (s, 1H, H10), 6.27 (dd, J = 8.8, 7.2 Hz, 1H, H4), 6.12 (dd, J = 8.7, 6.8 Hz, 1H, H4), 3.85 (d, J = 9.7 Hz, 1H, H1), 2.31 (s, 3H, H21), 2.30 (s, 3H, H22), 2.28 (s, 3H, H22), 2.26 (s, 3H, H21), 2.23 (H20) 2.22 (s, 3H, H20), 1.66 (s, 3H, H21), 1.26 (s, 3H, H21) ppm.

^{13}C{^{1}H} NMR (101 MHz, CD_2Cl_2) δ = 157.9 (d, $^{2}J_{PC}$ = 5.9 Hz, C3), 156.4 (d, $^{2}J_{PC}$ = 5.2 Hz, C3), 146.0 (C8), 141.5 (C8), 138.7 (C11), 137.0 (C11), 136.73 (d, $^{4}J_{PC}$ = 2.6 Hz, C5), 136.15 (m,

C5), 135.2 (C9), 134.6 (C9), 134.2 (d, $^4J_{PC}$ = 3.4 Hz, C15), 134.1 (d, $^2J_{PC}$ = 8.6 Hz, C13), 133.5 (C9), 133.4 (d, $^3J_{PC}$ = 9.8 Hz, C7), 132.2 (C17), 131.9 (d, $^3J_{PC}$ = 8.8 Hz, C7), 131.7 (d, $^4J_{PC}$ = 3.0 Hz, C18), 131.3 (d, $^4J_{PC}$ = 2.9 Hz, C18), 131.0 (C9), 130.7 (C10), 130.7 (C10), 130.2 (C10), 129.8 (C10), 129.6 (d, $^3J_{PC}$ = 11.7 Hz, C14), 127.0 (d, $^3J_{PC}$ = 13.2 Hz, C6), 125.4 (d, $^3J_{PC}$ = 13.0 Hz, C6), 125.1 (C16), 122.3, 121.7 (C19), 120.6 (d, $^1J_{PC}$ = 84.2 Hz, C12) 118.3 (d, $^3J_{PC}$ = 9.9 Hz, C4), 117.1 (d, $^3J_{PC}$ = 8.8 Hz, C4), 109.2 (d, $^1J_{PC}$ = 92.4 Hz, C2), 99.4 (d, $^1J_{PC}$ = 93.2 Hz, C2), 66.4 (d, $^1J_{PC}$ = 39.6 Hz, C1), 21.0 (C22), 21.0 (C22), 20.3 (C21), 20.1 (C20), 19.9 (C20), 19.4 (C21), 17.3 (C21), 17.0 (C21) ppm.

^{19}F NMR (377 MHz, CD_2Cl_2) δ = –63.4 ppm.

^{31}P{^{1}H} NMR (162 MHz, CD_2Cl_2) δ = 9.0 ppm.

Elemental Analysis: Calculated: C 52.77, H 4.05, N 2.62; Found: C 52.89, H 4.24, N 2.46

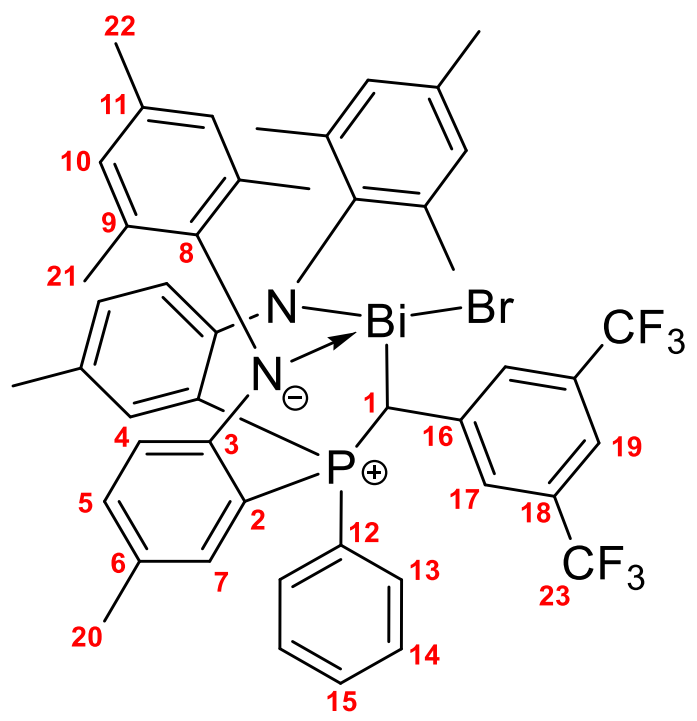

2.12 Synthesis of $4_{ArF}Cl$

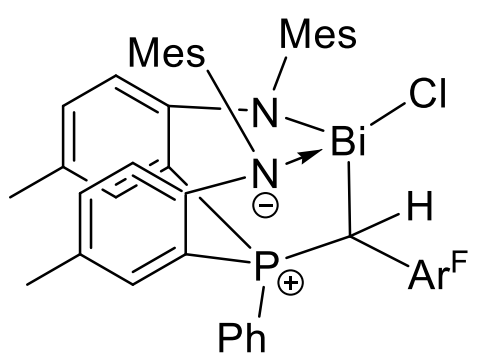

Phosphonium salt **$2_{ArF}Br$** (408 mg, 472 µmol, 1 eq) and KH (22.7 mg, 567 µmol, 1.2 eq) were dissolved in THF (10 mL) inside a Schlenk flask and the yellow suspension was stirred at RT for 1 d. Next the resulting orange suspension was filtered to isolate the orange solution from a disposable yellow precipitate and a solution of *n*-BuLi in *n*-hexane (1.6M, 0.59 mL, 945 µmol, 2 eq) was added at -78 °C. The dark red solution was stirred for 3 h at RT and then dropped to a solution of $BiCl_3$ (149 mg, 472 µmol, 1 eq) in THF (5 mL) at -78 °C. The dark brown solution was stirred for 1 d at RT. The next day the solvent was evaporated and the remaining dark brown solid was dissolved in toluene (8 mL). The dark solution was isolated by filtration and the solvent was evaporated. The remaining orange solid was washed two times with hexane (2x 5 mL) and the remaining yellow solid was characterized as the targeted product. Single crystals suitable for XRD analysis were obtained by slow diffusion of *n*-hexane into a concentrated benzene solution of **$4_{ArF}Cl$**.

Yield: 54 % (263 mg, 257 µmol)

^{1}H NMR (400 MHz, CD_2Cl_2) δ = 7.66 (s, 1H, H19), 7.63 (m, 1H, H15), 7.49 (td, $^3J_{HH}$ = 7.8, $^4J_{PH}$ = 3.2 Hz, 2H, H14), 7.39 (s, 2H, H17), 7.19 (d, 8.7 Hz, 1H, H5), 7.16 (d, 8.8 Hz, 1H, H5), 7.10 (m, 4H, H7 & H13), 7.03 (s, 1H, H10), 6.97 (d, $^4J_{HH}$ = 2.2 Hz, 1H, H10), 6.85 (d, $^4J_{HH}$ = 2.2 Hz, 1H, H10), 6.83 (d, $^4J_{HH}$ = 2.1 Hz, 1H, H10), 6.27 (dd, $^4J_{PH}$ = 8.8, $^3J_{HH}$ = 7.2 Hz, 1H, H4), 6.10 (dd, $^4J_{PH}$ = 8.7, $^3J_{HH}$ = 6.8 Hz, 1H, H4), 3.76 (d, $^2J_{PH}$ = 9.5 Hz, 1H, H1), 2.32 (s, 3H, H21), 2.30 (s, 3H, H22), 2.28 (s, 3H, H22), 2.27 (s, 3H, H21), 2.23 (s, 3H, H20), 2.21 (s, 3H, H20), 1.66 (s, 3H, H21), 1.26 (s, 3H, H21) ppm.

$^{13}C\{^1H\}$ NMR (101 MHz, CD_2Cl_2) δ = 158.2 (d, $^2J_{PC}$ = 5.9 Hz, C3), 156.5 (d, $^2J_{PC}$ = 5.2 Hz, C3), 146.2 (C8), 141.2 (C8), 139.1 (C11), 136.9 (C11), 136.8 (d, $^4J_{PC}$ = 2.3 Hz, C5), 136.3 (d, $^4J_{PC}$ = 2.2 Hz, C5), 135.0 (C9), 134.7 (C9), 134.4 (d, $^1J_{PC}$ = 7.2 Hz, C12), 134.2 (C9), 134.1 (d, $^3J_{PC}$ = 4.6 Hz, C13), 133.5 (C9), 133.5 (d, $^2J_{PC}$ = 10.3 Hz, C7), 132.14, 132.0 (d, $^2J_{PC}$ = 8.6 Hz, C7), 131.8 (d, $^4J_{PC}$ = 2.9 Hz, C15), 130.7 (C10), 130.6 (C10), 130.2 (C10), 129.8 (C10), 129.6 (d, $^2J_{PC}$ = 11.7 Hz, C14), 126.9 (d, J = 13.2 Hz, C6), 125.0 (d, J = 6.6 Hz, C6), 124.9 (C16), 121.6 (C19), 118.3 (d, $^3J_{PC}$ = 9.9 Hz, C4), 117.1 (d, $^3J_{PC}$ = 8.8 Hz, C4), 109.3 (d, $^1J_{PC}$ = 92.8 Hz, C2), 98.8 (d, $^1J_{PC}$ = 92.2 Hz, C2), 68.5 (d, $^1J_{PC}$ = 43.2 Hz, C1), 21.0 (C22), 20.9 (C22), 20.2 (C20), 20.1 (C20), 19.9 (C21), 19.4 (C21), 17.3 (C21), 17.1 (C21) ppm.

^{19}F NMR (377 MHz, CD_2Cl_2) δ = –63.4 ppm.

$^{31}P\{^1H\}$ NMR (162 MHz, C_2Cl_2) δ = 8.9 ppm.

Elemental Analysis: Calculated: C 55.06, H 4.23, N 2.73; Found: C 54.89, H 4.25, N 2.48

LIFDI-HRMS: 1024.2560 [M^+] calculated, 1024.2616 [M^+] measured.

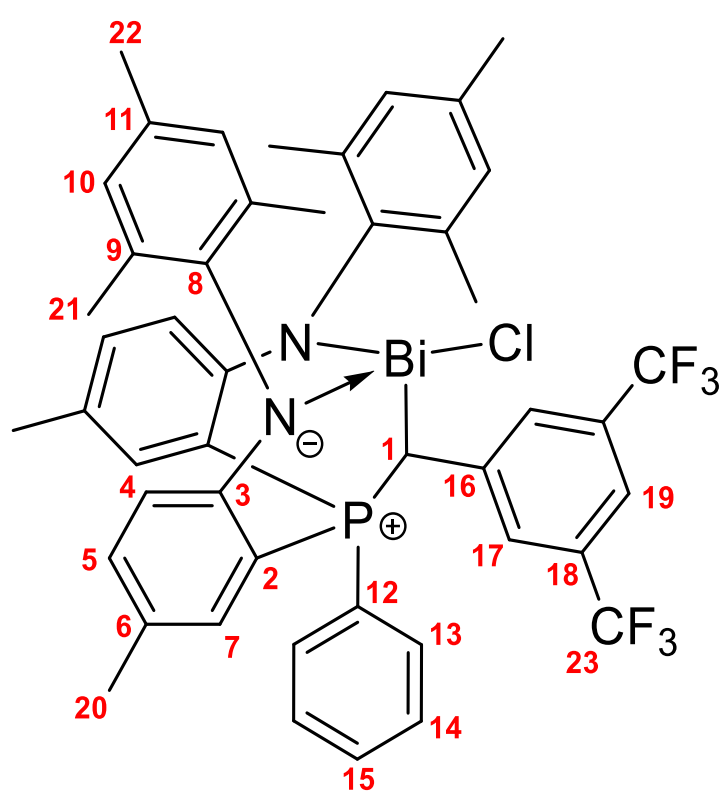

2.13 Synthesis of 5_{Ph}OTf

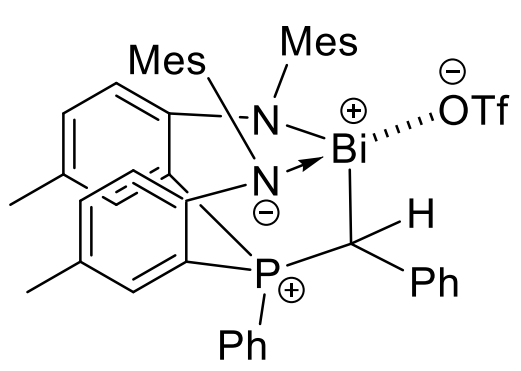

4_{Ph}Cl (69.2 mg, 77.8 µmol, 1.00 eq) and AgOTf (20.0 mg, 77.8 µmol, 1.02 eq) were dissolved in THF (2 mL) and the mixture was stirred for 1 d at RT. After this time the solvent was evaporated and the remaining dark green precipitate was suspensed in toluene (2 mL). An orange solution was separated from a black precipitate and the solvent was evaporated. The remaining solid was washed once with pentane (2 mL) and the product was isolated as a yellow solid. Single crystals suitable for XRD analysis were obtained by slow diffusion of *n*-hexane into a concentrated THF solution of **5_{Ph}OTf**.

Yield: 55 % (42.6 mg, 42.5 µmol)

^1H NMR (400 MHz, CD_2Cl_2) δ = 7.64 (m, 1H, H15), 7.48 (m, 2H, H14), 7.28 (m, 4H, H5, H7 & H18), 7.20 (m, 2H, H5 & H19), 7.12 (ddd, $^3J_{PH}$ = 11.3, $^3J_{HH}$ = 8.4, $^4J_{HH}$ = 1.4 Hz, 2H, H13), 7.04 (s, 1H, H10), 7.02 (m, 1H, H7), 7.01 (s, 1H, H10), 6.86 (s, H10), 6.84 (s, H10), 6.72 (m, 2H, H17), 6.31 (dd, $^4J_{PH}$ = 8.8, $^3J_{HH}$ = 7.1 Hz, 1H, H4), 6.23 (dd, $^4J_{PH}$ = 9.0, $^3J_{HH}$ = 7.0 Hz, 1H, H4), 3.55 (d, $^2J_{PH}$ = 11.3 Hz, 1H, H1), 3.38 (m, 2H, THF), 2.39 (s, 3H, H21), 2.30 (m, 6H, H20 & H22), 2.27 (s, 3H, H22), 2.26 (s, 3H, H21), 2.23 (s, 3H, H20), 1.58 (m, 5H, THF & H21), 1.20 (s, 3H, H21) ppm.

$^{13}C\{^1H\}$ NMR (101 MHz, CD_2Cl_2) δ = 156.9 (d, $^2J_{PC}$ = 5.9 Hz, C3), 155.8 (d, $^2J_{PC}$ = 5.5 Hz C3), 144.6 (C8), 140.5 (C8), 138.4 (C11), 137.4 (C11), 136.5 (d, $^4J_{PC}$ = 9.0 Hz, C18), 136.5 (d, $^4J_{PC}$

= 2.9 Hz, C5), 134.8 (d, $^2J_{PC}$ = 8.4 Hz, C13), 134.6 (C9), 134.4 (d, $^4J_{PC}$ = 2.7 Hz, C15), 134.2 (C9), 133.7 (d, $^2J_{PC}$ = 9.5 Hz, C7), 133.6 (C10), 132.2 (d, $^2J_{PC}$ = 8.1 Hz, C7), 132.0 (d, $^3J_{PC}$ = 5.9 Hz, C17), 131.0 (C10), 130.8 (C10), 130.7 (C10), 129.6 (C16), 129.4 (d, $^3J_{PC}$ = 12.5 Hz, C14), 129.02 (d, J = 2.9 Hz), 128.7 (C19), 128.5 (d, $^4J_{PC}$ = 4.9 Hz, C5), 127.6 (d, 2.2 Hz, C6), 127.5 (d, 2.5 Hz, C6), 119.56 (d, $^1J_{PC}$ = 85.8 Hz, C12), 118.10 (d, $^3J_{PC}$ = 10.3 Hz, C4), 108.49 (d, $^1J_{PC}$ = 89.5 Hz, C2), 103.3 (d, $^1J_{PC}$ = 91.7 Hz, C2), 71.4 (d, $^1J_{PC}$ = 38.5 Hz, C1), 70.9 (THF), 27.0 (THF), 21.0 (C22), 20.2 (C20), 19.9 (C20), 19.6 (C21), 17.4 (C21), 17.0 (C21) ppm.

^{19}F NMR (377 MHz, CD_2Cl_2) δ = –79.2 ppm.

^{31}P{^{1}H} NMR (162 MHz, CD_2Cl_2) δ = 7.5 ppm.

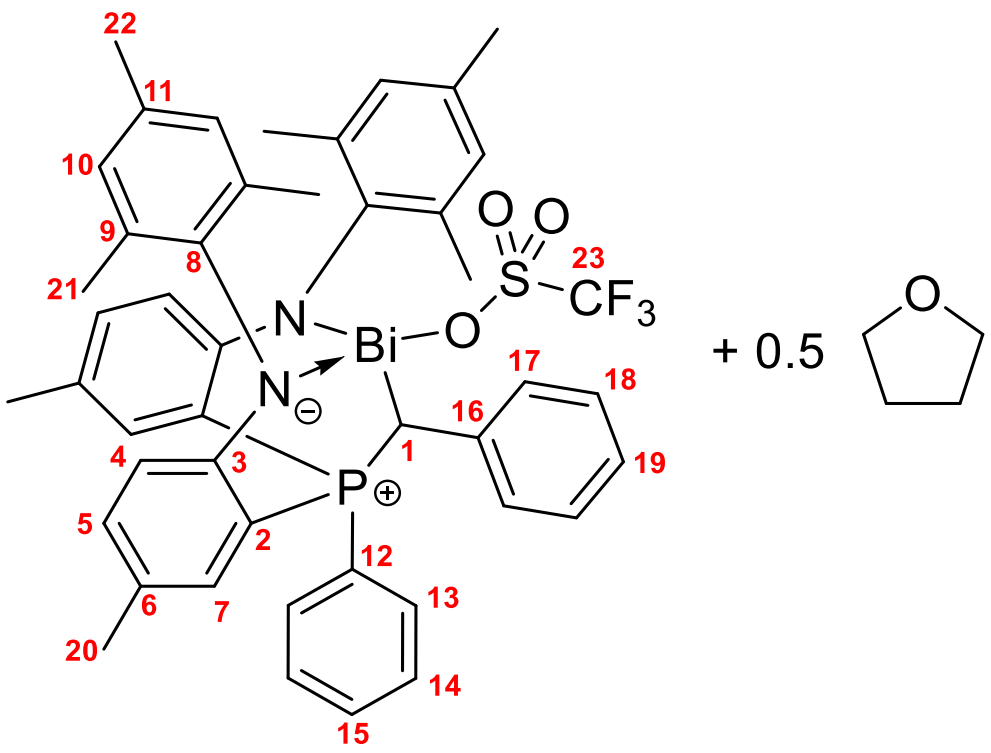

2.14 Synthesis of $5_{Ph}BPh_4$

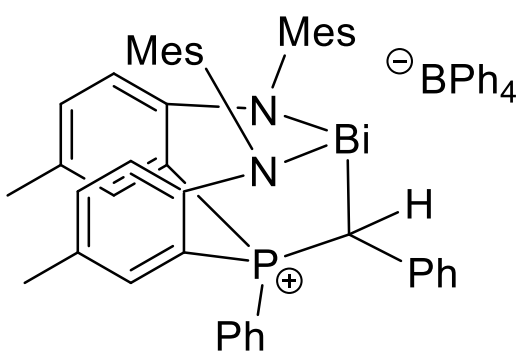

Bismuth complex **4_{Ph}Cl** (129 mg, 145 µmol, 1.00 eq) and $NaBPh_4$ (50.0 mg, 145 µmol, 1.00 eq) were dissolved in DCM and the solution was shaken for 1 d at RT. The solution was isolated from a precipitate via filtration and the solvent was evaporated. The remaining orange solid was washed three times with *n*-hexane (3x 2 mL), yielding **$5_{Ph}BPh_4$** as an orange solid. Single crystals suitable for XRD analysis were obtained by slow evaporation of a concentrated DCM solution of **$5_{Ph}BPh_4$**.

Yield: 70 % (120 mg, 102 µmol)

^{1}H NMR (400 MHz, CD_2Cl_2) δ = 7.67 (t, $^3J_{HH}$ = 7.5 Hz, 1H, H15), 7.52 (td, $^3J_{HH}$ = 7.8, $^4J_{HP}$ = 3.5 Hz, 2H, H14), 7.34 (t, J = 7.5 Hz, 2H, H18), 7.24 (m, 3H, H5 & H19), 7.10 (m, 12H, H13, H25 & H26), 7.00 (m, 2H, H7), 6.88 (s, H10), 6.81 (s, H10), 6.78 (m, 12H, H10, H24 & H26), 6.56 (d, $^2J_{HP}$ = 7.7 Hz, 2H, H17), 6.30 (t, $^4J_{HP}$ = 9.2, $^3J_{HH}$ = 7.9 Hz, 1H, H4), 6.16 (dd, $^4J_{HP}$ = 9.3, $^3J_{HH}$ = 7.8 Hz, 1H, H4), 3.31 (d, $^2J_{HP}$ = 12.3 Hz, 1H, H1), 2.35 (s, 3H, H22), 2.30 (s, 3H, H20), 2.26 (m, 6H, H21 & H22), 2.24 (s, 3H, H20), 2.11 (s, 3H, H21), 1.56 (s, 3H, H21), 1.12 (s, 3H, H21) ppm.

^{13}C NMR (101 MHz, CD_2Cl_2) δ = 165.1 (d, $^3J_{CP}$ = 49.2 Hz, C8), 164.1 (d, $^3J_{CP}$ = 49.5 Hz, C8), 156.2 (d, $^2J_{CP}$ = 5.5 Hz, C3), 155.4 (d, $^2J_{CP}$ = 5.1 Hz, C3), 143.9 (C11), 140.8 (C11), 137.0 (C9), 136.9 (C9), 136.7 (d, $^4J_{CP}$ = 2.9 Hz, C5), 136.5 (s, C25), 135.1 (C9), 134.8 (d, $^3J_{CP}$ = 8.5 Hz, C13), 134.7 (d, $^4J_{CP}$ = 3.0 Hz, C15), 134.3 (C9), 133.6 (d, J = 9.5 Hz, C7), 132.05 (d, $^4J_{CP}$ = 8.0 Hz, C5), 131.8 (d, $^3J_{CP}$ = 5.5 Hz, C17), 131.3 (C26), 131.1 (C10), 131.0 (C10), 130.6 (C10), 129.6 (d, $^2J_{CP}$ = 10.0 Hz, C14), 129.5 (d, $^4J_{CP}$ = 4.9 Hz, C18), 129.2 (d, $^5J_{CP}$ = 3.6 Hz, C19), 128.4 (m, C6 & C16), 126.2 (C26) 126.1 (C26), 122.0 (C24), 118.9 (d, $^1J_{CP}$ = 86.5 Hz, C12), 118.5 (d, $^4J_{CP}$ = 10.1 Hz, C4), 118.1 (d, $^4J_{CP}$ = 8.3 Hz, C4), 108.4 (d, $^3J_{CP}$ = 89.1 Hz, C2), 102.82

(d, $^{1}J_{CP}$ = 91.3 Hz, C2), 73.2 (d, $^{1}J_{CP}$ = 35.7 Hz, C1), 21.1 (C22), 21.0 (C22), 20.2 (C20), 20.0 (C21), 19.9 (C20), 19.7 (C21), 17.5 (C21), 16.7 (C21) ppm.

^{31}P NMR (162 MHz, CD_2Cl_2) δ = 6.9 ppm.

Elemental Analysis: Calculated: C 70.65, H 5.58, N 2.39; Found: C 70.90, H 5.66, N 2.11

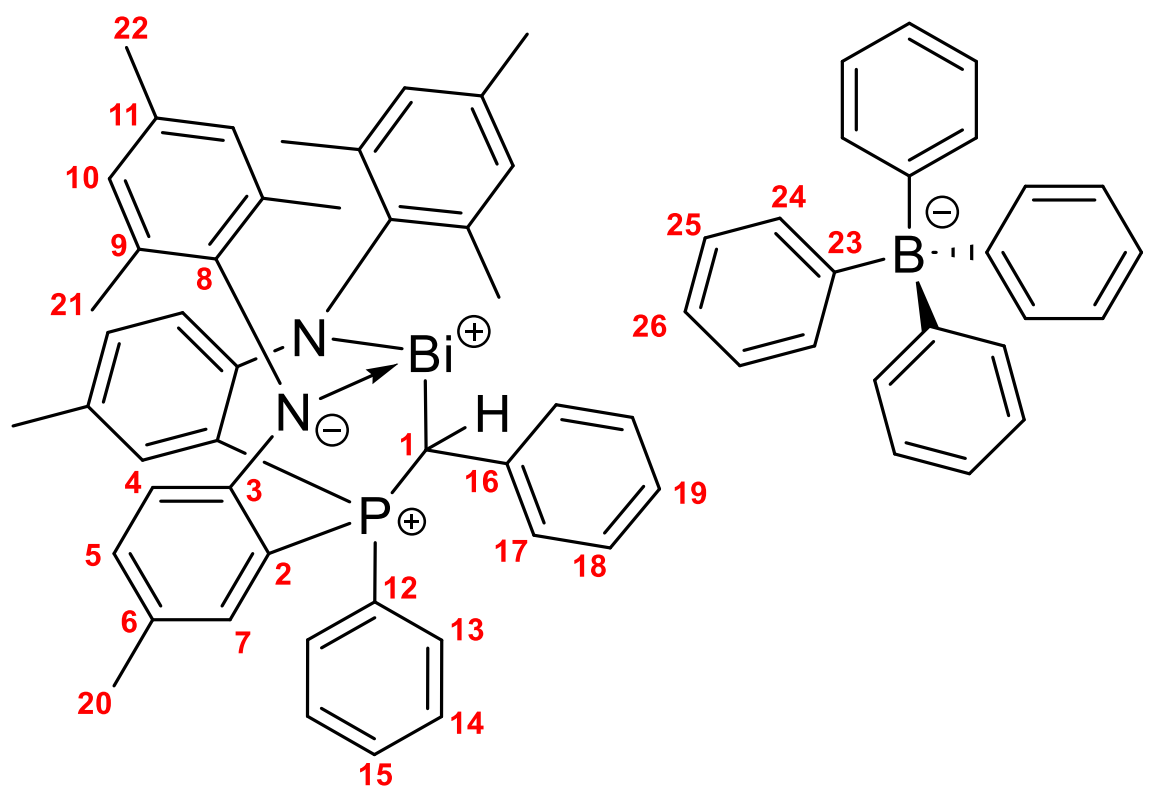

2.15 Synthesis of bismuth yldiide 6

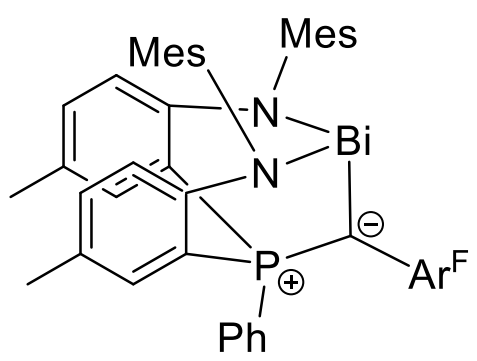

Bismuth complex **4_{Ar^F}Br** (24.4 mg, 22.8 µmol, 1.00 eq) and KHMDS (5.00 mg, 25.1 µmol, 1.10 eq) were dissolved in THF in a Schlenk tube. The dark brown solution was stirred at RT for 1 d. The next day the solvent was evaporated and the remaining dark brown solid was immediately taken up in d_8-THF for characterization by NMR spectroscopy on the same day. Isolation in the solid state could not be achieved, also storage in solution for prolonged time resulted in selective decomposition to **7**.

Yield: -

^{1}H NMR (400 MHz, THF-d_8) δ = 8.01 (ddd, $^{3}J_{PH}$ = 10.2, $^{3}J_{HH}$ = 6.1, $^{4}J_{HH}$ = 3.3 Hz, 2H, H13), 7.62 (m, 3H, H14 & H15), 7.28 (dd, $^{3}J_{PH}$ = 14.3, $^{4}J_{HH}$ = 2.2 Hz, 2H, H7), 6.94 (d, $^{3}J_{HH}$ = 9.2 Hz, 2H, H5), 6.89 (s, 2H, H10), 6.81 (s, 1H, H19), 6.75 (s, 2H, H10), 6.68 (s, 2H, H17), 6.13 (dd, $^{3}J_{HH}$ = 8.5, $^{4}J_{PH}$ = 6.5 Hz, 2H, H4), 2.23 (s, 6H, H22), 2.20 (s, 12H, H20 & H21), 1.39 (s, 6H, H21) ppm.

^{13}C NMR (101 MHz, THF-d_8) δ = 155.6 (d, $^{2}J_{PC}$ = 4.3 Hz, C3), 147.4 (d, $^{2}J_{PC}$ = 1.6 Hz, C16), 145.9 (C8), 137.3 (C9), 136.8 (C9), 136.7 (d, $^{2}J_{PC}$ = 9.2 Hz, C13), 135.3 (C11), 133.6 (d, $^{4}J_{PC}$ = 2.6 Hz, C5), 133.4 (d, $^{4}J_{PC}$ = 2.9 Hz, C15), 131.6 (d, $^{2}J_{PC}$ = 8.8 Hz, C7), 130.5 (C10), 130.4 (C10), 130.2 (d, $^{3}J_{PC}$ = 11.4 Hz, C14), 128.9 (d, $^{4}J_{PC}$ = 11.4 Hz, C17), 127.0 (d, $^{1}J_{PC}$ = 82.1 Hz, C12), 126.7 (C18), 125.35 (d, $^{3}J_{PC}$ = 12.1 Hz, C6), 124.0 (C18), 118.3 (d, $^{3}J_{PC}$ = 7.7 Hz, C4), 115.0 (d, $^{1}J_{PC}$ = 95.0 Hz, C2), 110.9 (C19), 95.1 (d, $^{1}J_{PC}$ = 66.6 Hz, C1), 21.1 (C22), 20.4 (C20), 19.4 (C21), 17.9 (C21).

^{19}F NMR (377 MHz, CD_2Cl_2) δ = –63.6 ppm.

^{31}P NMR (162 MHz, THF-d_8) δ = −4.0 ppm.

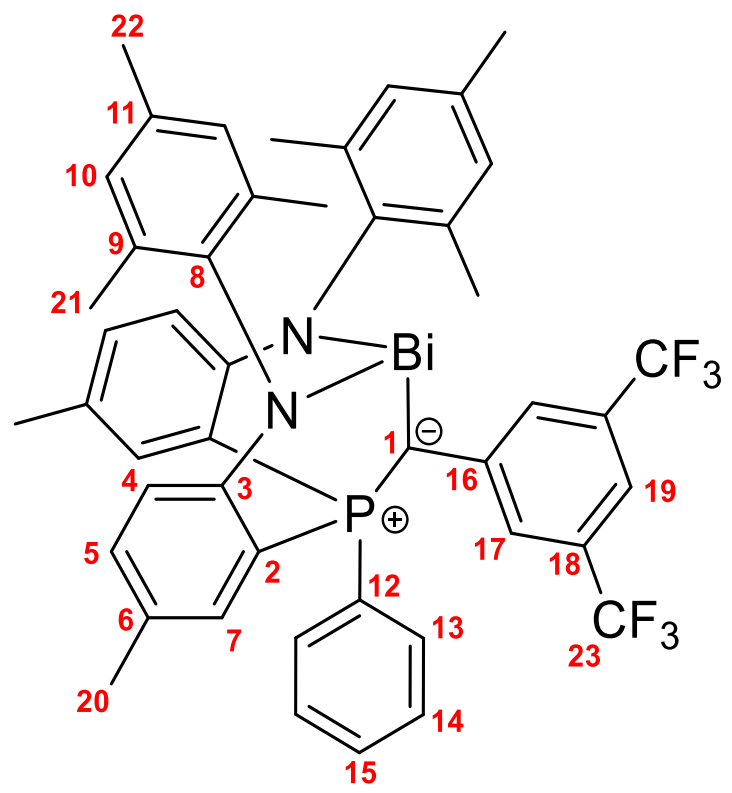

2.16 Synthesis of decomposition product 7

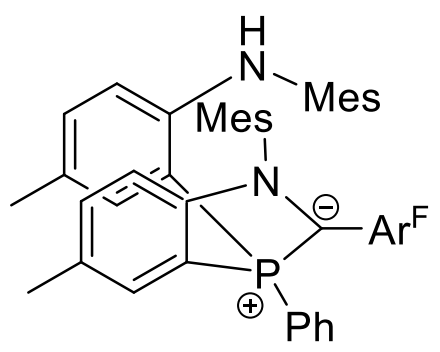

In a Young NMR tube, bismuth complex **4$_{ArF}$Br** (88.1 mg, 82.3 µmol, 1.00 eq) and LDA (10.0 mg, 90.5 µmol, 1.10 eq) were dissolved in THF. The resulting dark brown solution was shaken for 1 week at RT and the progress of the conversion of **6** to **7** monitored by ^{31}P NMR spectroscopy. For reaction completion, the solution was heated to 50 °C for 1 d. The solution was transferred to a Schlenk flask and the solvent evaporated. The dark red residue was taken up in toluene (1 mL) and the solution was filtered. The solvent of the filtrate was evaporated, and the residue was washed with *n*-pentane (2 mL) at –60 °C. The remaining dark red solid was characterized as 7 with impurities of unidentified compounds, which could not be removed by further washing steps. Crystals suitable for XRD analysis were obtained from a THF solution of **6** after storage for 4 weeks at –30 °C. During that time **6** decomposed to **7** to yield the crystals, despite the presence of DMAP as a potentially stabilizing additive.

Yield: 45 % (28.8 mg, 36.9 µmol)

^{1}H NMR (400 MHz, CD_2Cl_2) δ = 7.85 (m, 2H, H13), 7.60 (m, 3H, H14 & H15), 7.50 (d, $^{3}J_{PH}$ = 9.4 Hz, 1H, H7), 7.22 (d, $^{3}J_{PH}$ = 16.3 Hz, 1H), 7.02 (m, 3H, H5 & H10), 6.91 (s, 1H, H10), 6.79 (m, 4H, H10 & H17), 6.55 (s, 1H, H19), 6.22 (dd, $^{3}J_{HH}$ = 8.7, $^{4}J_{PH}$ = 4.3 Hz, 1H, H4), 5.98 (dd, $^{3}J_{HH}$ = 8.4, $^{4}J_{PH}$ = 6.2 Hz, 1H, H4), 5.71 (s, 1H, NH), 2.31 (s, 3H, H22), 2.25 (s, 3H, H20), 2.21 (s, 3H, H22), 2.16 (s, 3H, H20), 1.97 (s, 3H, H21), 1.97 (s, 3H, H21), 1.57 (s, 3H, H21), 1.50 (s, 3H, H21) ppm.

^{13}C{^{1}H} NMR (101 MHz, CD_2Cl_2) δ = 149.5 (C3), 147.2 (C3), 140.8 (d, $^{2}J_{PC}$ = 18.7 Hz, C16), 137.9 (C9), 137.4 (C8), 137.1 (C9), 136.6 (C8), 136.6 (C9), 136.3 (C9) 136.1 (C11), 135.8 (C11), 135.6 (C5), 133.3 (C5), 132.0 (d, $^{4}J_{PC}$ = 2.9 Hz, C15), 131.2 (d, $^{2}J_{PC}$ = 10.6 Hz, C13), 130.80 (d, $^{1}J_{PC}$ = 31.9 Hz, C12), 130.3 (C10), 129.7 (d, $^{3}J_{PC}$ = 12.5 Hz, C14), 129.6 (C10), 129.4 (C10), 129.2 (C10), 128.5 (d, $^{2}J_{PC}$ = 15.4 Hz, C7), 128.3 (d, $^{2}J_{PC}$ = 11.4 Hz, C7), 125.73 (d, $^{3}J_{PC}$ = 19.1 Hz, C6), 123.1 (C18), 115.6 (C17), 113.6 (d, $^{3}J_{PC}$ = 8.1 Hz, C4), 111.7 (d, $^{3}J_{PC}$ = 7.7 Hz, C4), 106.2 (C19), 102.1 (C2),* 99.24 (d, $^{1}J_{PC}$ = 96.8 Hz, C2), 64.8 (d, $^{1}J_{PC}$ = 137.9 Hz, C1), 21.0 (C22), 21.0 (C22), 20.6 (C20), 20.4 (C20), 18.5 (C21), 18.4 (C21), 17.6 (C21), 17.2 (C21) ppm.

*Found via ^{1}H-^{13}C HSQC or ^{1}H-^{13}C HMBC NMR experiment

^{19}F{^{1}H} NMR (377 MHz, CD_2Cl_2) δ = –64.2 ppm.

^{31}P{^{1}H} NMR (162 MHz, CD_2Cl_2) δ = –4.9 ppm.

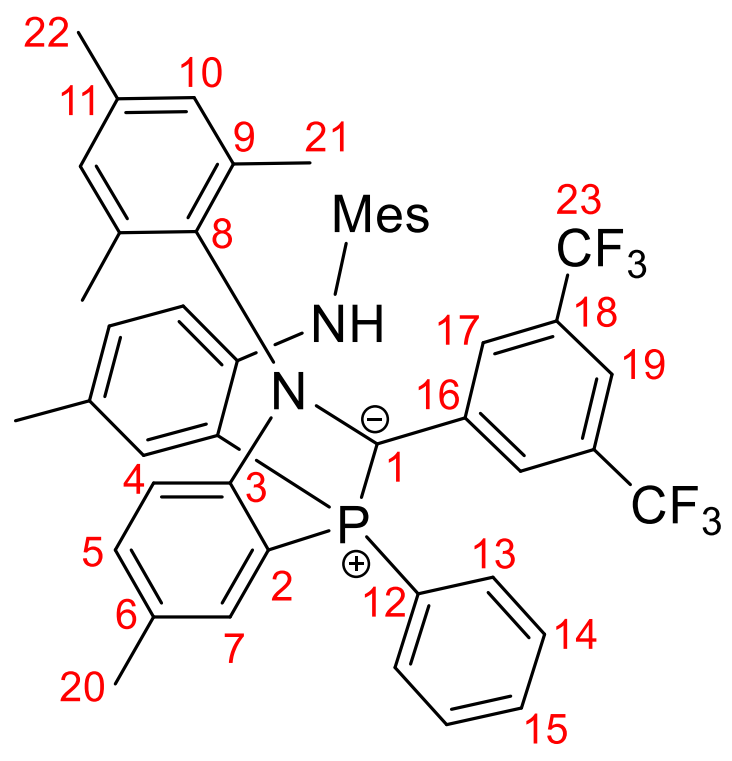

2.17 Synthesis of bismuth carbodiimide complex 8

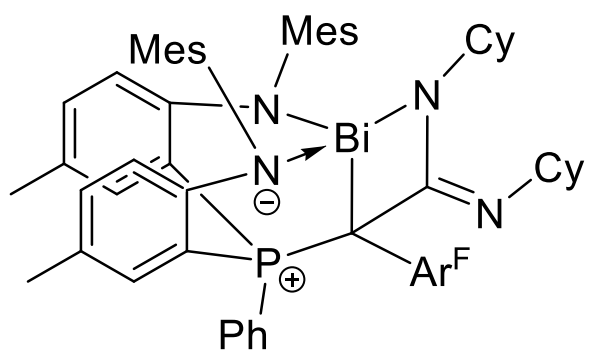

$\mathbf{4_{Ar^F}Br}$ (121 mg, 113 µmol, 1.00 eq) and KHMDS (25.0 mg, 119 µmol, 1.05 eq) were dissolved in THF (3 mL) in a Schlenk flask. The dark red solution was stirred for 1 d at RT. Subsequently, *N,N'*-dicyclohexylcarbodiimide (35.1 mg, 170 µmol, 1.50 eq) was added and the mixture stirred at RT for 1 d. The resulting dark brown solution was filtered to obtain a dark red solution. After evaporation of the solvent the dark brown residue was washed twice with pentane at 0 °C, giving the product as beige solid. Single crystals of **8** suitable for XRD analysis were obtained via slow evaporation of a concentrated toluene solution.

Yield: 41 % (55.3 mg, 46.3 µmol)

^{1}H NMR (400 MHz, CD_2Cl_2) δ = 8.37 (s, 2H, H17), 7.65 (s, 1H, H19), 7.54 (td, $^{3}J_{HH}$ = 7.5, $^{4}J_{HH}$ = 1.7 Hz, 1H, H15), 7.35 (m, 2H, H14), 7.05 (m, 2H, H15), 7.03 (m, 1H, H7), 7.02 (m, 1H, H5), 6.93 (s, 1H, H10), 6.92 (s, 1H, H10), 6.89 (m, 1H, H5), 6.80 (m, 1H, H7), 6.75 (s, 1H, H10), 6.74 (s, 1H, H10), 6.27 (dd, $^{4}J_{PH}$ = 8.7, $^{3}J_{HH}$ = 7.2 Hz, 1H, H4), 5.98 (dd, *J* = 8.6, 6.3 Hz, 1H, H4), 3.89 (t, $^{3}J_{HH}$ = 9.8 Hz, 1H, H25), 3.48 (t, $^{3}J_{HH}$ = 10.4 Hz, 1H, H25), 2.28 (s, 3H, H21), 2.26 (m, 6H, H21 & H22), 2.19 (s, 3H, H22), 2.18 (s, 3H, H20), 2.04 (s, 3H, H20), 1.59 (m, 4H, H26), 1.46 (s, 3H, H21), 1.29 (m, 6H, H26 & H27), 1.19 (s, 3H, H21), 1.07 (m, 4H, H27), 0.89 (m, 3H, H27 & H28), 0.61 (m, 3H, H27 & H28) ppm.

^{13}C{^{1}H} NMR (101 MHz, CD_2Cl_2) δ = 158.5 (d, 6.9 Hz, C3), 157.2 (d, 4.8 Hz, C3), 151.8 (C24), 148.3 (C8), 143.1 (C8), 139.5 (C11), 137.6 (C11), 136.6 (C9), 135.6 (d, $^{3}J_{PC}$ = 10.3 Hz, C13), 135.0 (C9), 134.8 (d, $^{4}J_{PC}$ = 2.9 Hz, C5), 134.6 (C9), 134.4 (d, $^{4}J_{PC}$ = 2.6 Hz, C5), 134.13 (d, $^{2}J_{PC}$ = 8.4 Hz, C7), 132.6 (C17), 132.8 (C9), 132.25 (d, $^{2}J_{PC}$ = 8.4 Hz, C7), 130.7 (C10) 130.4 (C10), 130.1 (C10), 129.8 (C10), 129.5 (d, $^{4}J_{PC}$ = 4.0 Hz, C15), 127.68 (d, $^{3}J_{PC}$ = 12.1 Hz, C14), 124.7 (d, $^{2}J_{PC}$ = 13.2 Hz, C6), 121.7 (d, 83.4 Hz, C12) 121.6 (d, $^{2}J_{PC}$ = 12.6 Hz, C6), 120.5 (C19), 117.9 (d, $^{3}J_{PC}$ = 9.5 Hz, C4), 116.8 (d, $^{3}J_{PC}$ = 9.5 Hz, C4), 109.28 (d, $^{1}J_{PC}$ = 91.3 Hz, C2), 104.66 (d, $^{1}J_{PC}$ = 89.5 Hz, C2), 56.6 (C25), 56.1 (C25), 35.4 (C26), 25.9 (C27), 25.1 (C28), 21.0 (C22), 20.8 (C22), 20.8 (C21), 20.5 (C21), 20.1 (C20), 20.0 (C20), 17.6 (C21), 17.1 (C21) ppm.

^{19}F NMR (377 MHz, CD_2Cl_2) δ = –62.9 ppm.

^{31}P NMR (162 MHz, CD_2Cl_2) δ = 7.2 ppm.

LIFDI-HRMS: 1194.4577 [M^+] calculated, 1194.4510 [M^+] measured.

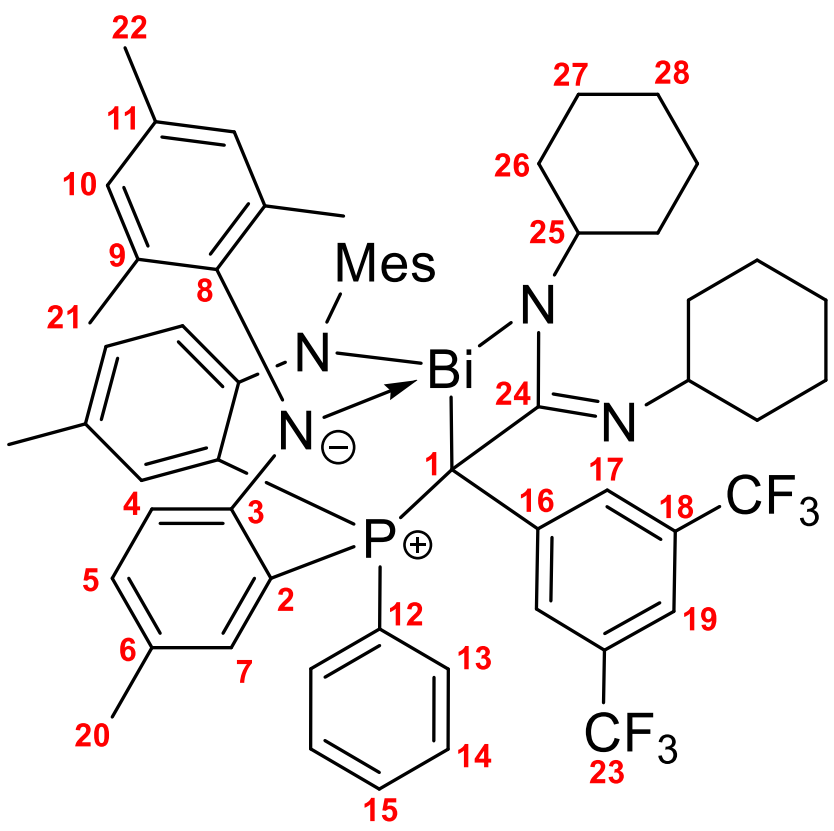

2.18 General protocols for further reactivity studies of 6

General protocol for gaseous substrates

4ArFBr (30.0 mg, 28.0 µmol, 1.00 eq) and LDA (3.41 mg, 30.8 µmol, 1.10 eq) were dissolved in Et_2O inside a J-Young NMR tube. The tube was shaken for 1 d at RT. Upon completion of the deprotonation, the atmosphere in the tube was exchanged via a freeze pump process to the respective gas with a pressure of 2 bar. The tube was shaken at RT and the reaction progress following the gas exposure was monitored using $^{31}P\{^1H\}$ NMR spectroscopy.

General protocol for solid substrates

4ArFBr (30.0 mg, 28.0 µmol, 1.00 eq) and LDA (3.41 mg, 30.8 µmol, 1.10 eq) were dissolved in Et_2O inside a J-Young NMR tube. The tube was shaken for 1 d at RT. Upon completion of the deprotonation, the solid substrate (28.0 µmol, 1.00 eq) was added to the tube inside an argon-filled glove box. The tube was shaken at RT and the reaction progress following the gas exposure was monitored using $^{31}P\{^1H\}$ NMR spectroscopy.

3 Determination of the Lewis acidity via the Gutmann-Beckett method

The protocol was inspired by a study on the Lewis acidity of cationic bismuth complexes published by Lichtenberg.[3] In a J-Young NMR tube an equimolar mixture of $SePMe_3$ and **5_{Ph}OTf** or **5_{Ph}OTf** were dissolved in CD_2Cl_2 and the tube was shaken for 3 h. After that time the $^{31}P\{^1H\}$ NMR spectrum was measured and the acceptor number AN was determined using formula (1). The value of δ_{free} equals a shift of 7.80 ppm.

$$\text{AN} = 5.71 \cdot (\delta_{\text{accepting}} - \delta_{\text{free}}) \quad (1)$$

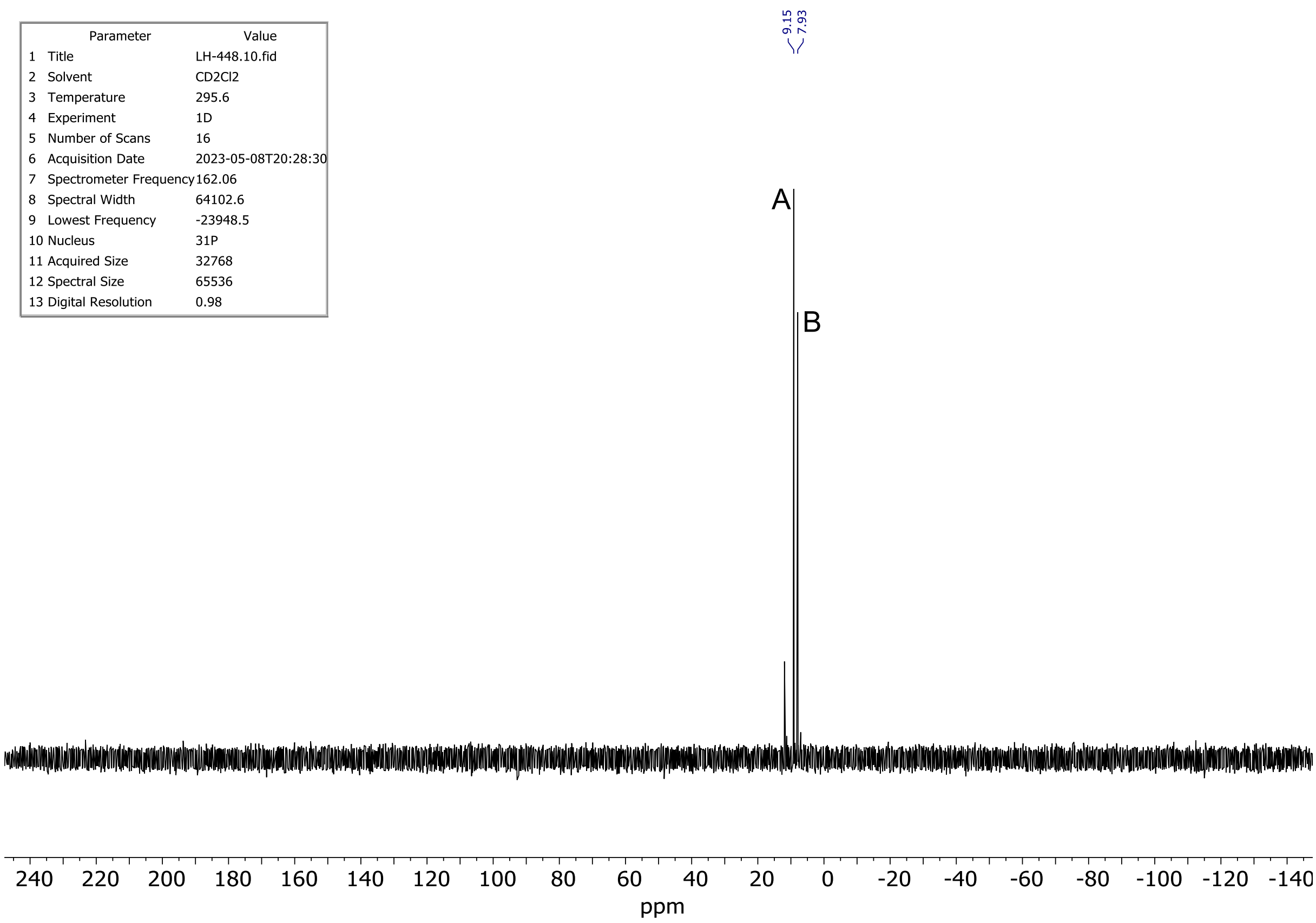

Figure S1: $^{31}P\{^1H\}$ NMR spectrum of a 1:1 mixture of $SePMe_3$ (A) and **5_{Ph}OTf** (B) in CD_2Cl_2.

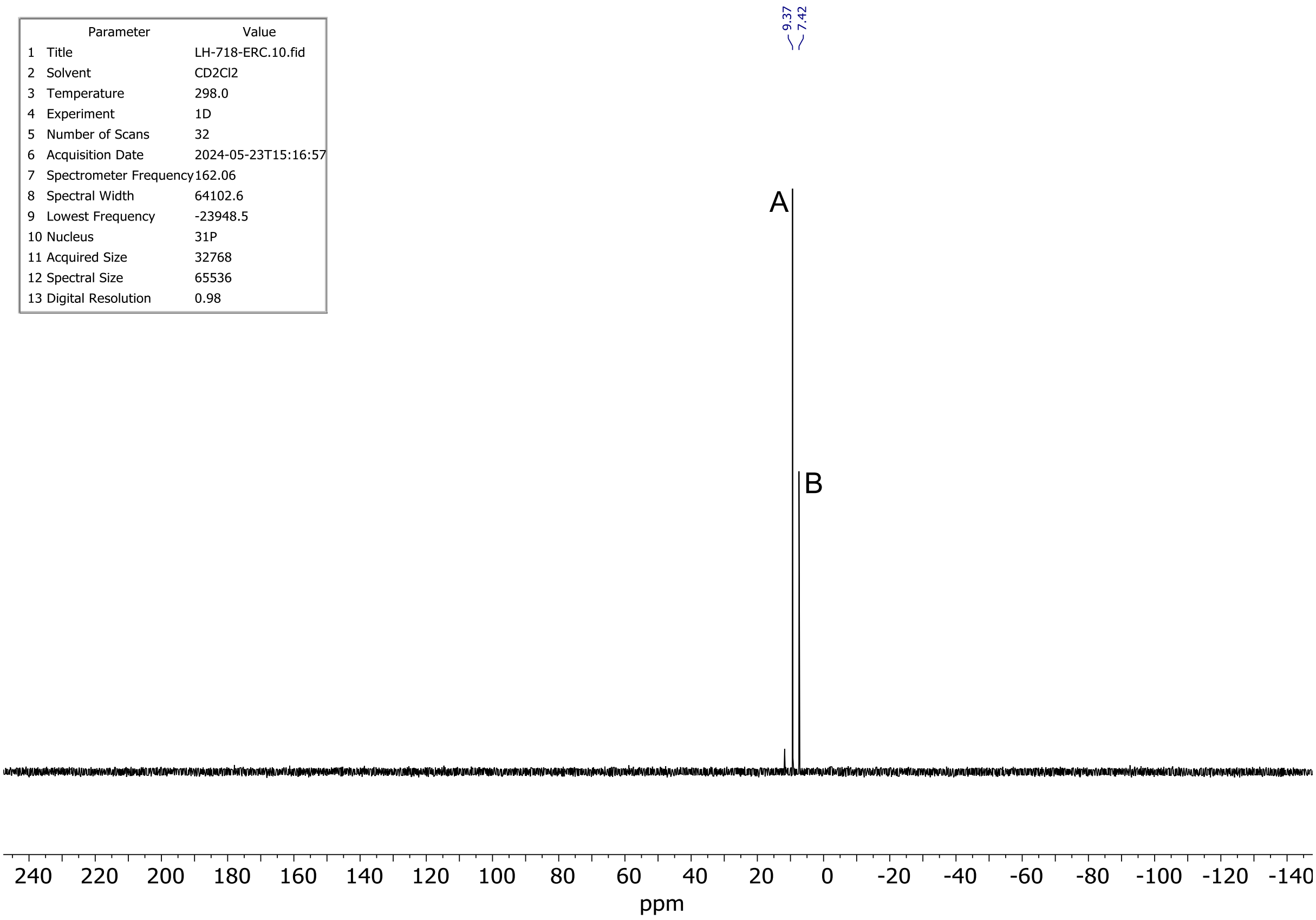

Figure S2: $^{31}P\{^{1}H\}$ NMR spectrum of a 1:1 mixture of $SePMe_3$ (A) and $\mathbf{5_{Ph}BPh_4}$ (B) in CD_2Cl_2.

4 NMR study on the decomposition of 6 to 7

In three Young NMR tubes, a mixture of $\mathbf{4_{ArF}Br}$ (1.00 eq) and LDA (1.10 eq) was dissolved in THF-d_8 (0.6 mL). To investigate a possible concentration dependency of the decomposition of **6** to **7**, three concentrations of $\mathbf{4_{ArF}Br}$ were chosen: 34.33 µmol/mL, 68.67 µmol/mL and 137.33 µmol/mL. The signal of **6** can be found at –4.0 ppm, while **7** has a chemical shift of –5.20 ppm. The progress of the decomposition was monitored by collecting ^{31}P NMR spectra in an interval of 1 d and comparing the integrals of the signals of the two species.

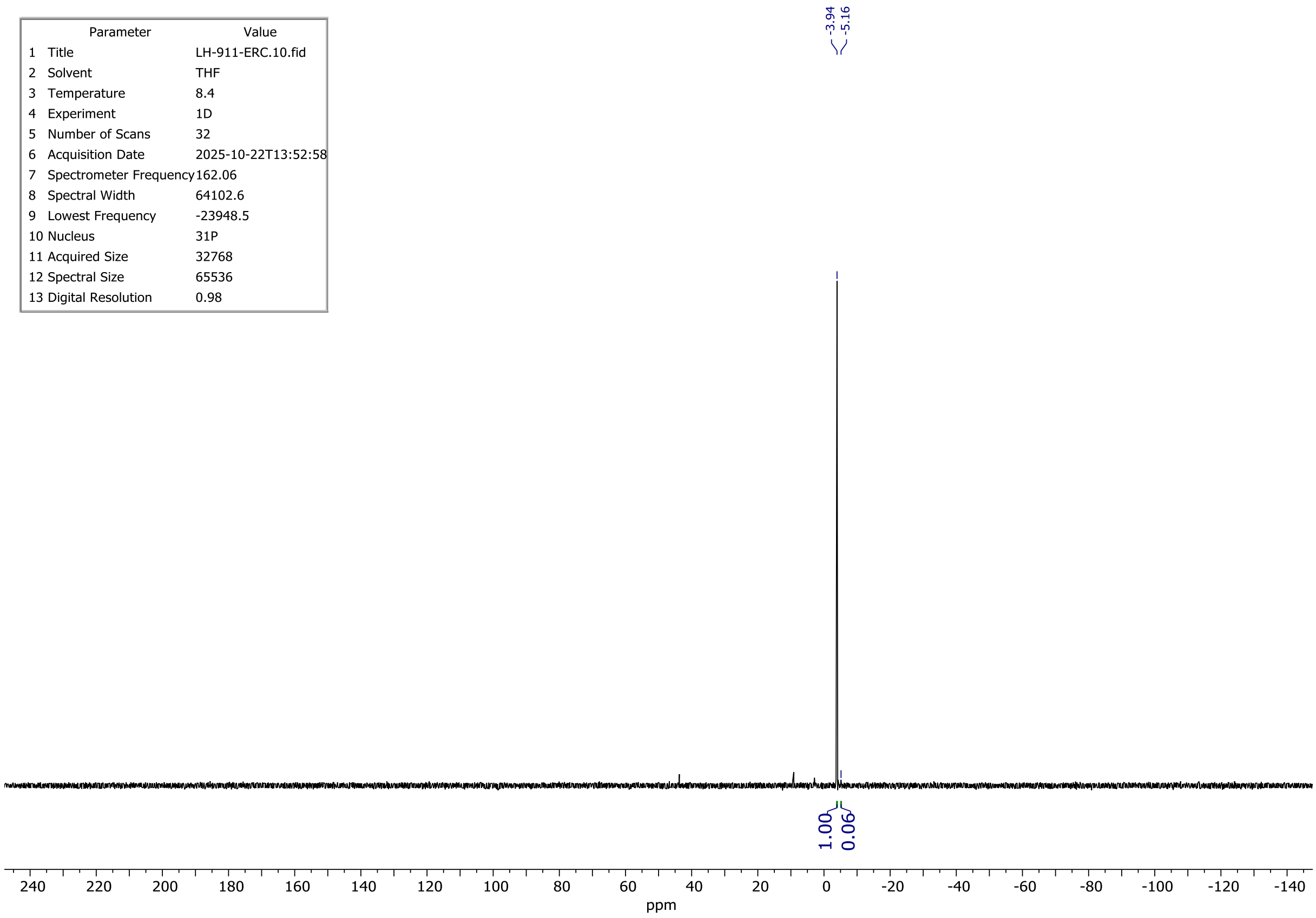

Figure S3: ^{31}P{^{1}H} NMR spectrum of a THF-d_8 solution of **6** with a concentration of 34.33 µmol/mL, 1 d after $\mathbf{4_{ArF}Br}$ and LDA have been dissolved in the solvent.

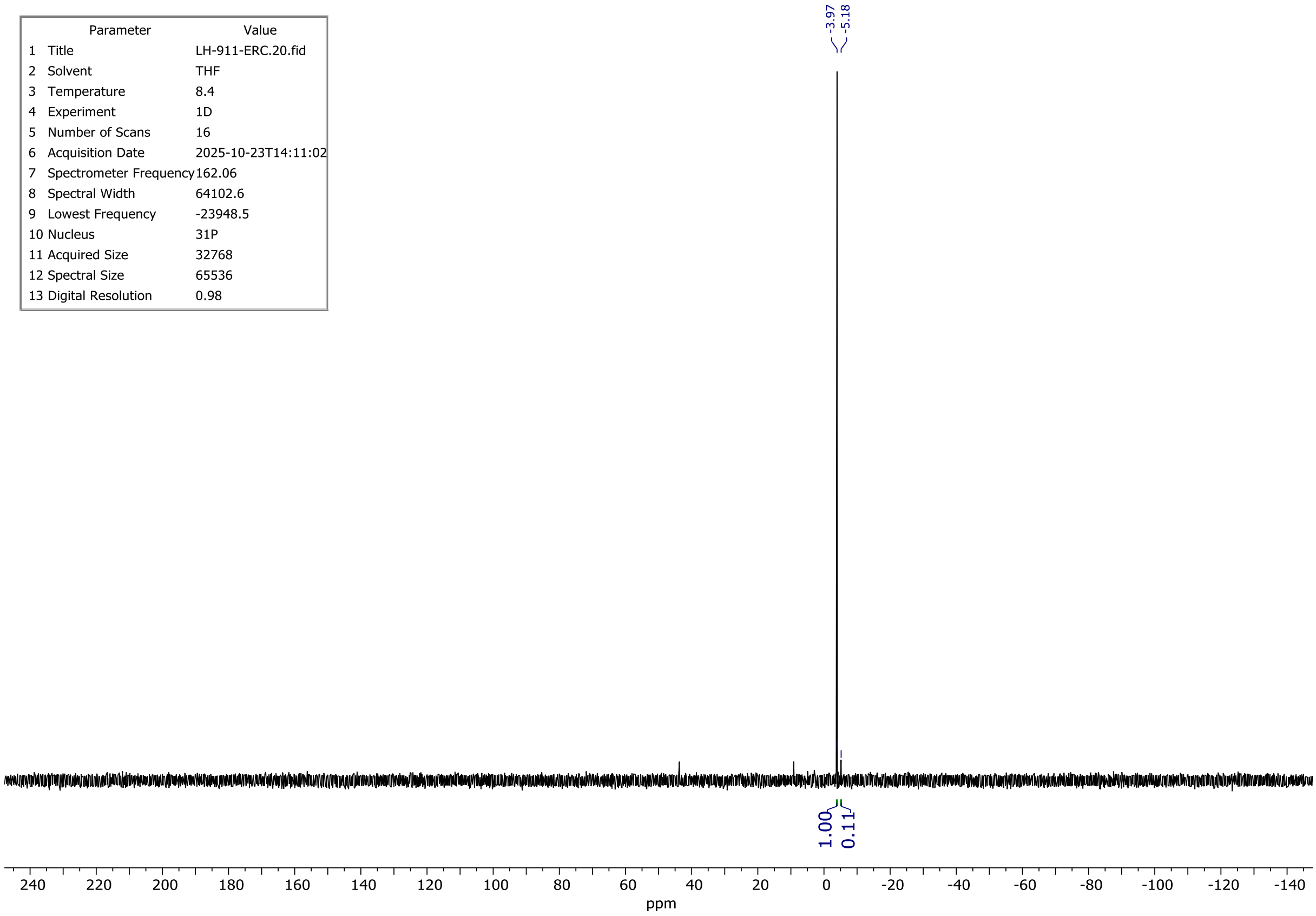

Figure S4: $^{31}P\{^{1}H\}$ NMR spectrum of a THF-d$_8$ solution of **6** with a concentration of 34.33 µmol/mL, 2 d after **4$_{Ar}$FBr** and LDA have been dissolved in the solvent.

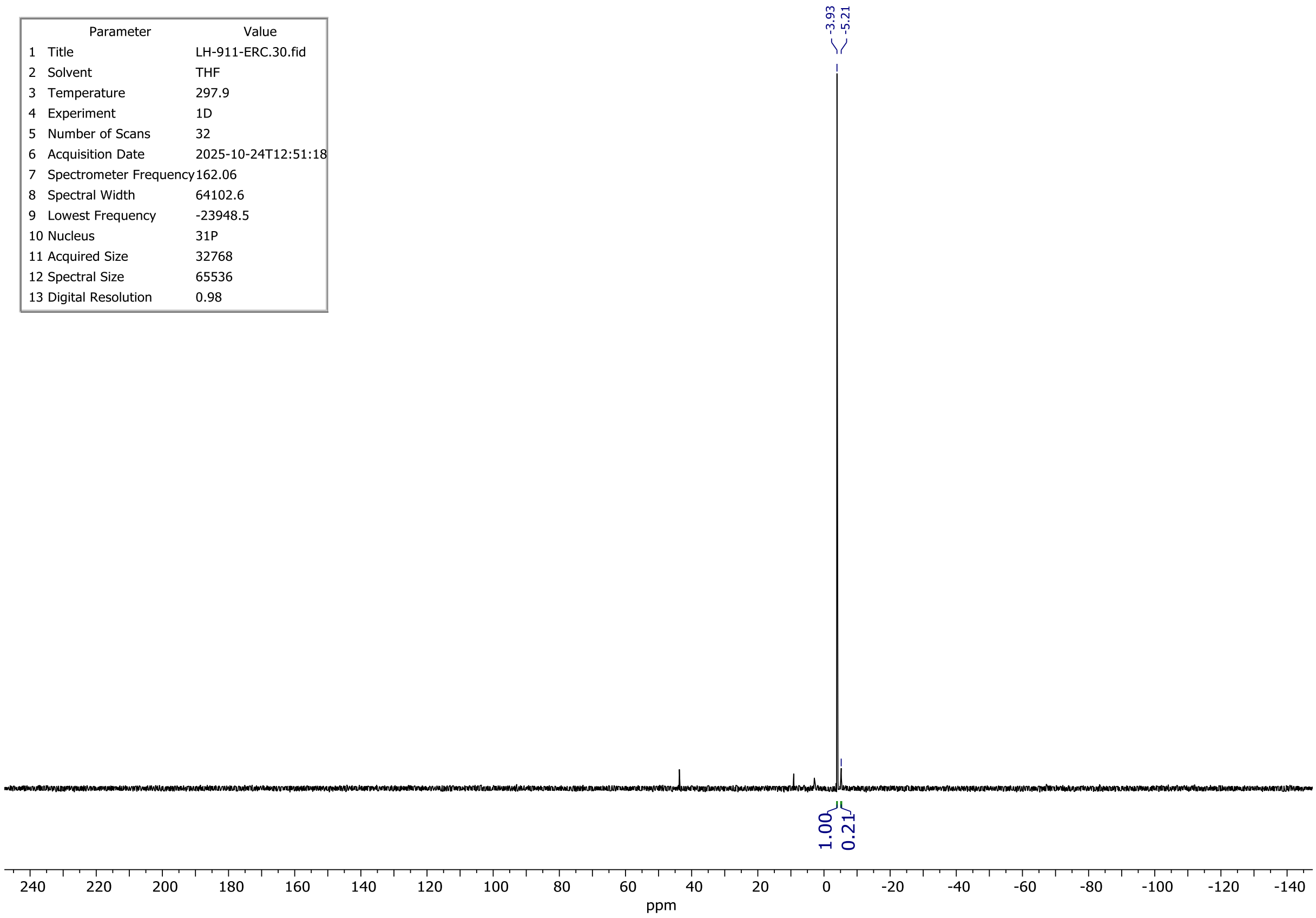

Figure S5: $^{31}P\{^{1}H\}$ NMR spectrum of a THF-d$_8$ solution of **6** with a concentration of 34.33 µmol/mL, 3 d after **4$_{Ar}$FBr** and LDA have been dissolved in the solvent.

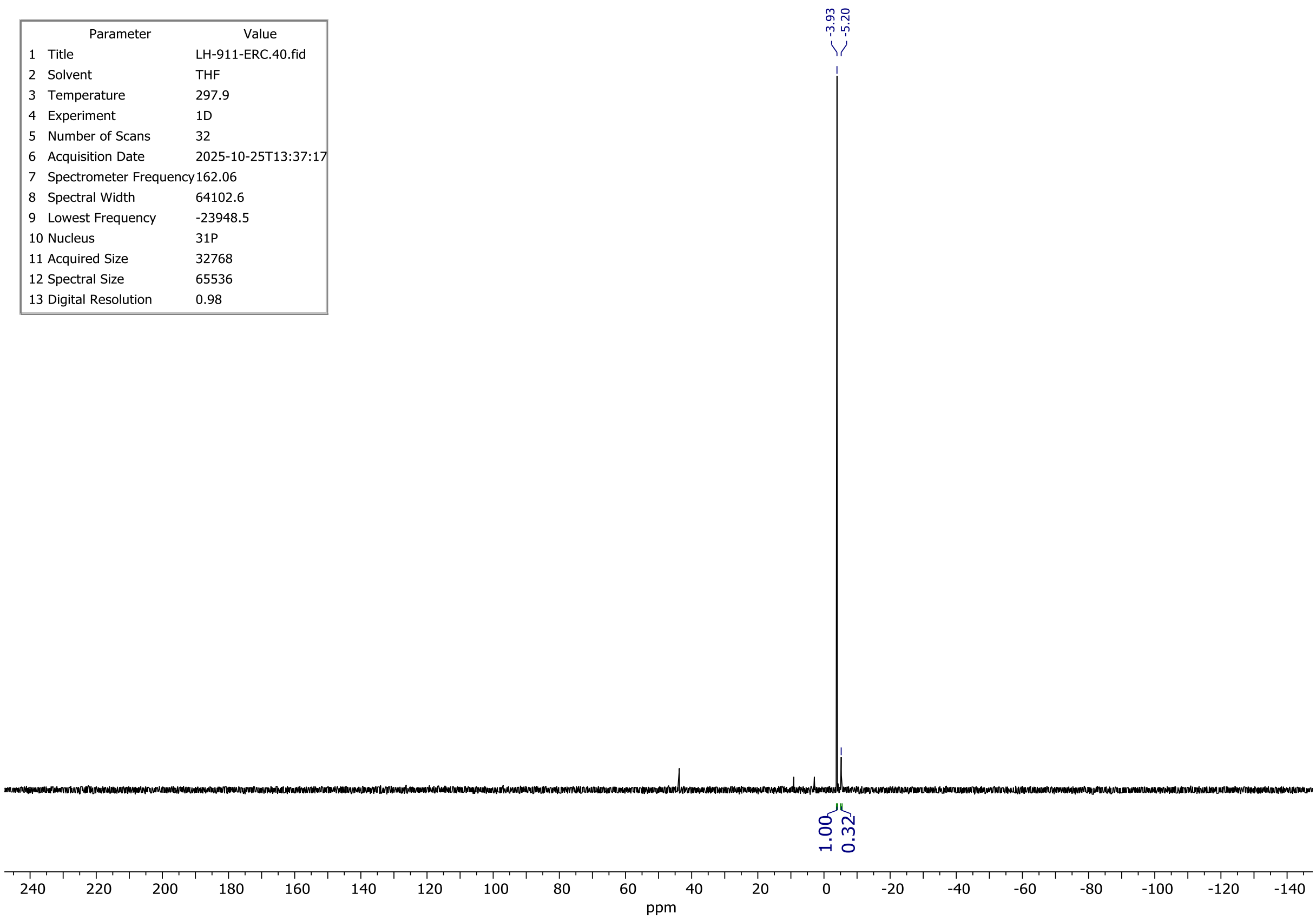

Figure S6: $^{31}P\{^{1}H\}$ NMR spectrum of a THF-d_8 solution of **6** with a concentration of 34.33 µmol/mL, 4 d after **4_{Ar}FBr** and LDA have been dissolved in the solvent.

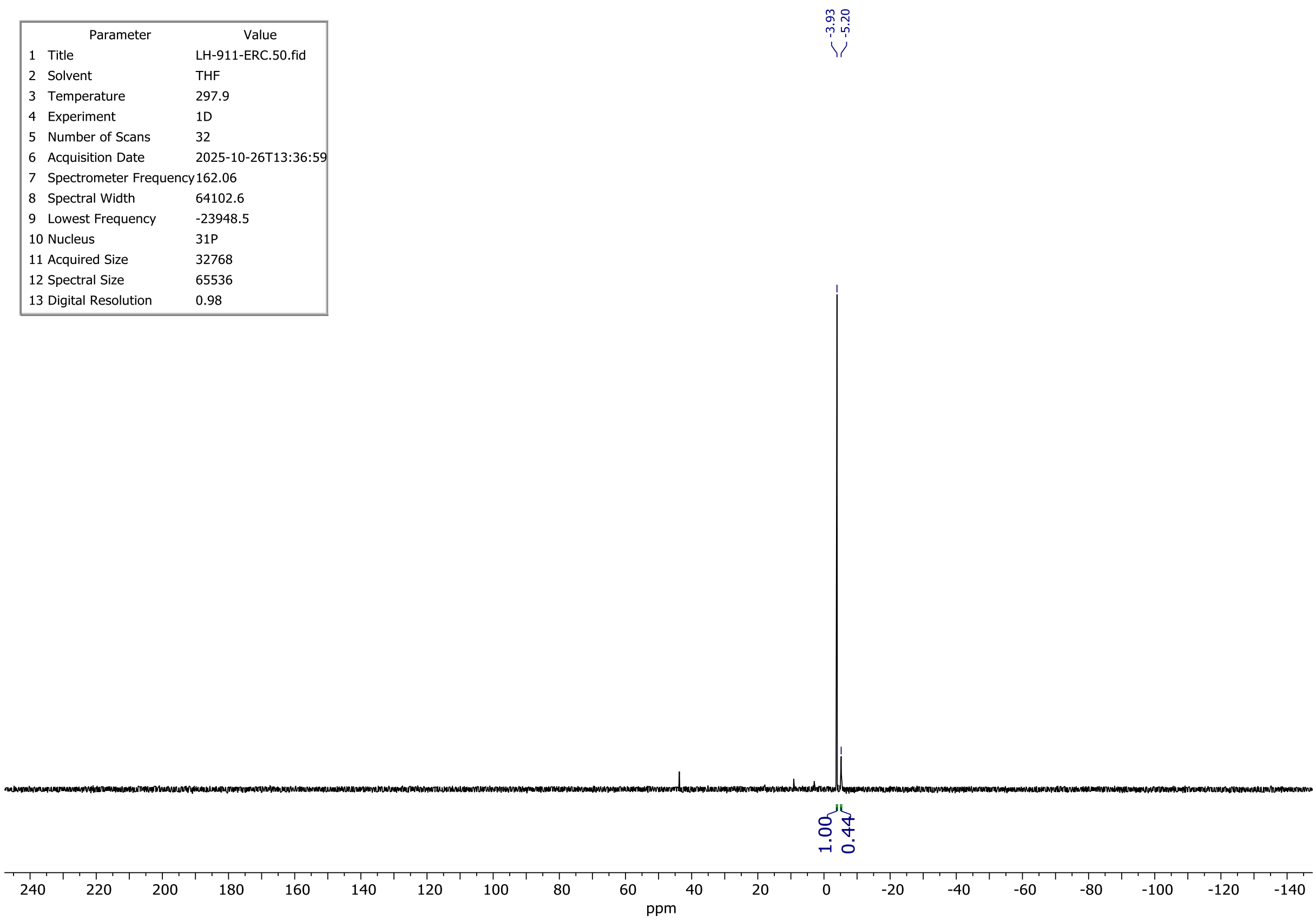

Figure S7: $^{31}P\{^{1}H\}$ NMR spectrum of a THF-d_8 solution of **6** with a concentration of 34.33 µmol/mL, 5 d after **4_{Ar}FBr** and LDA have been dissolved in the solvent.

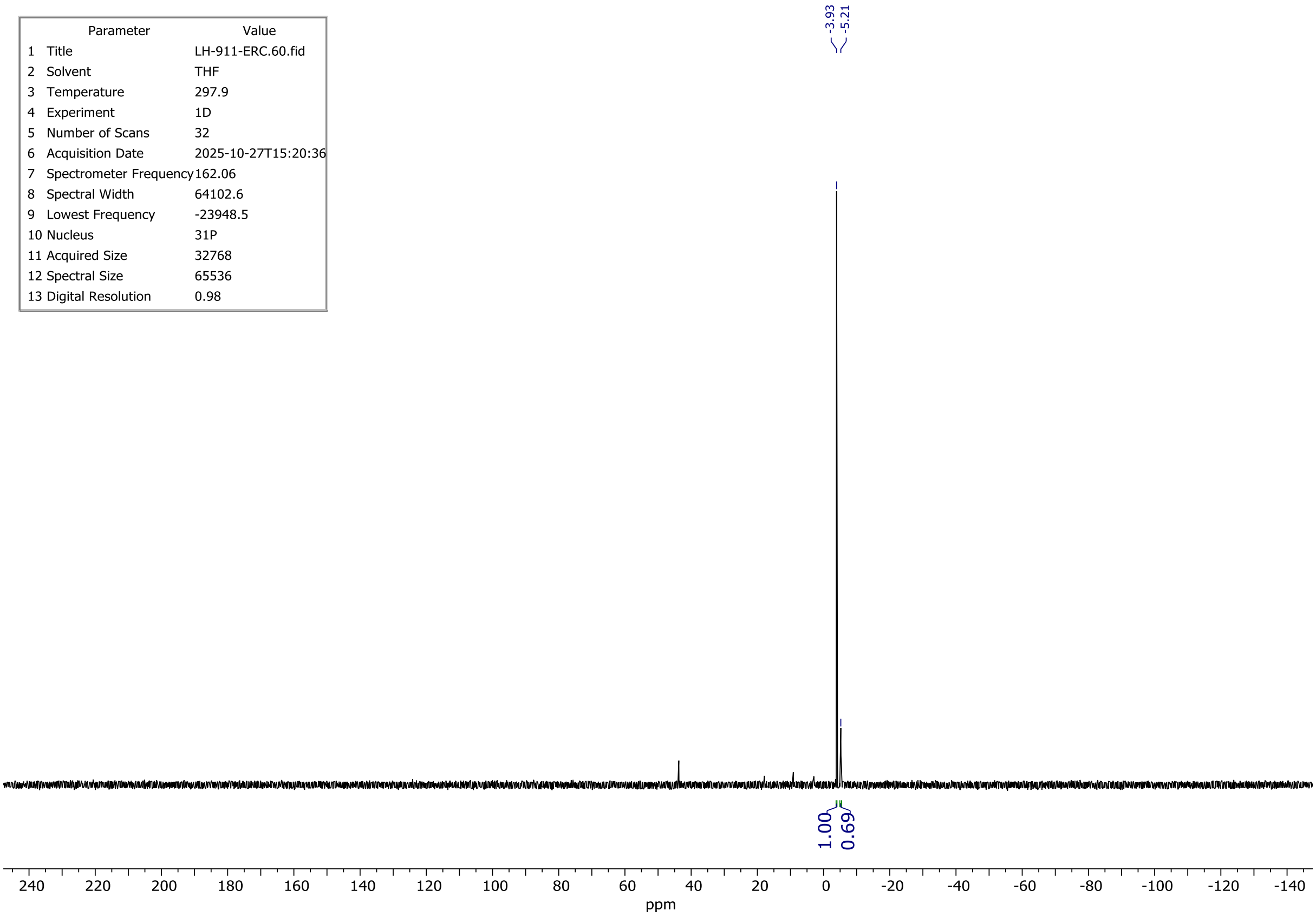

Figure S8: $^{31}P\{^{1}H\}$ NMR spectrum of a THF-d_8 solution of **6** with a concentration of 34.33 µmol/mL, 6 d after **4_{Ar}FBr** and LDA have been dissolved in the solvent.

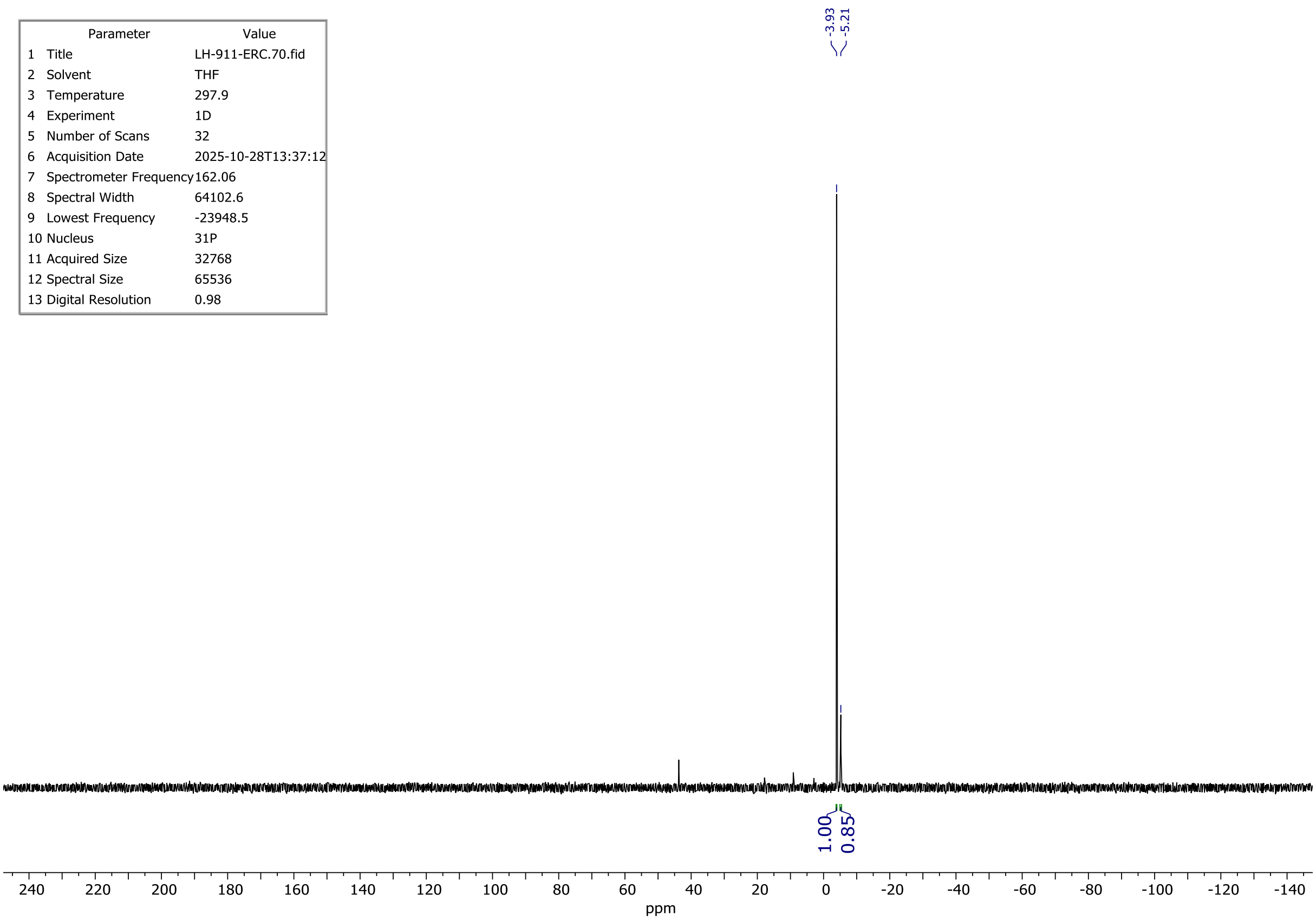

Figure S9: $^{31}P\{^{1}H\}$ NMR spectrum of a THF-d_8 solution of **6** with a concentration of 34.33 µmol/mL, 7 d after **4_{Ar}FBr** and LDA have been dissolved in the solvent.

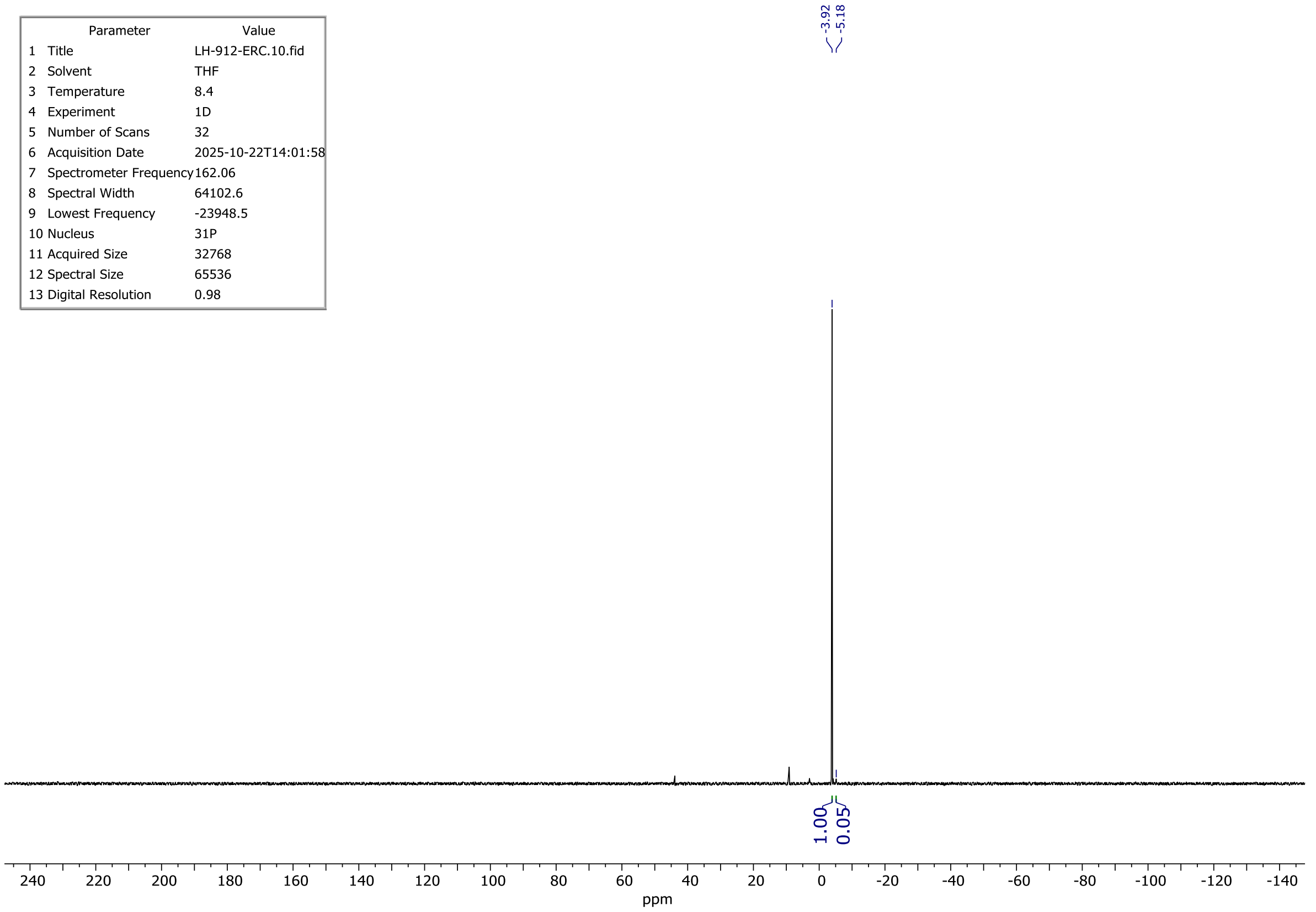

Figure S10: $^{31}P\{^1H\}$ NMR spectrum of a THF-d_8 solution of **6** with a concentration of 68.67 µmol/mL, 1 d after **4_{ArF}Br** and LDA have been dissolved in the solvent.

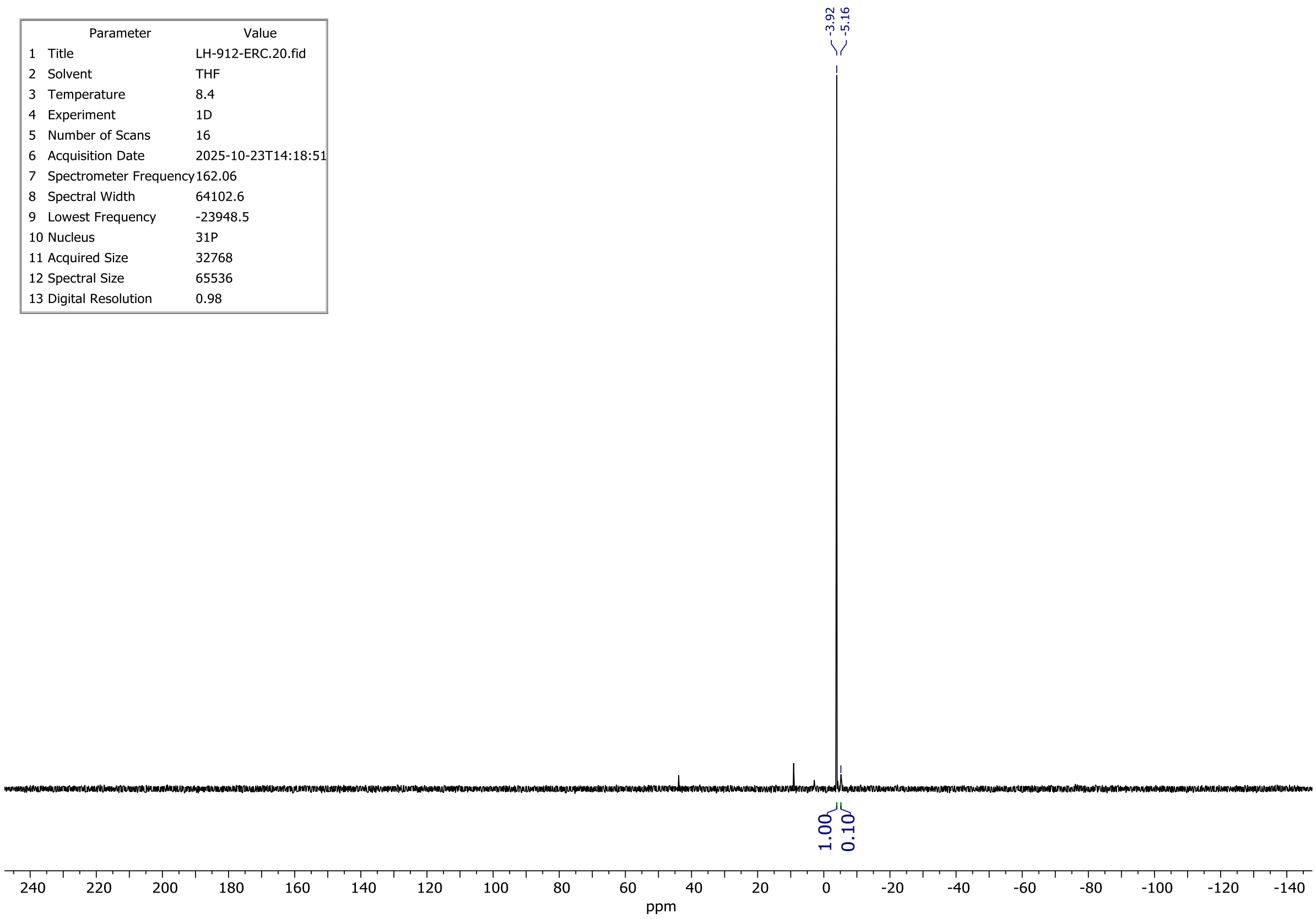

Figure S11: $^{31}P\{^1H\}$ NMR spectrum of a THF-d_8 solution of **6** with a concentration of 68.67 µmol/mL, 2 d after **4_{ArF}Br** and LDA have been dissolved in the solvent.

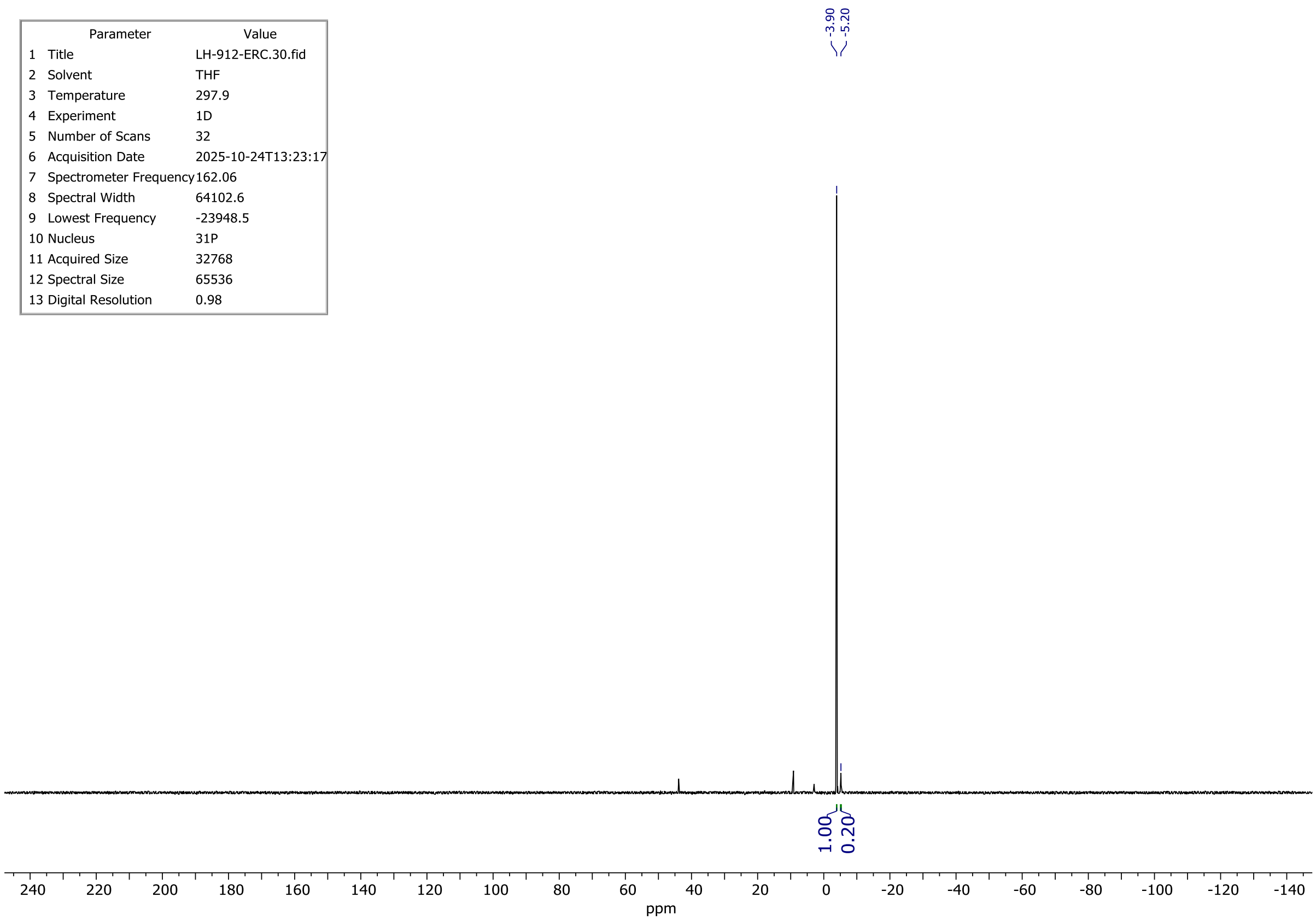

Figure S12: $^{31}P\{^1H\}$ NMR spectrum of a THF-d$_8$ solution of **6** with a concentration of 68.67 µmol/mL, 3 d after **4$_{\mathbf{ArF}}$Br** and LDA have been dissolved in the solvent.

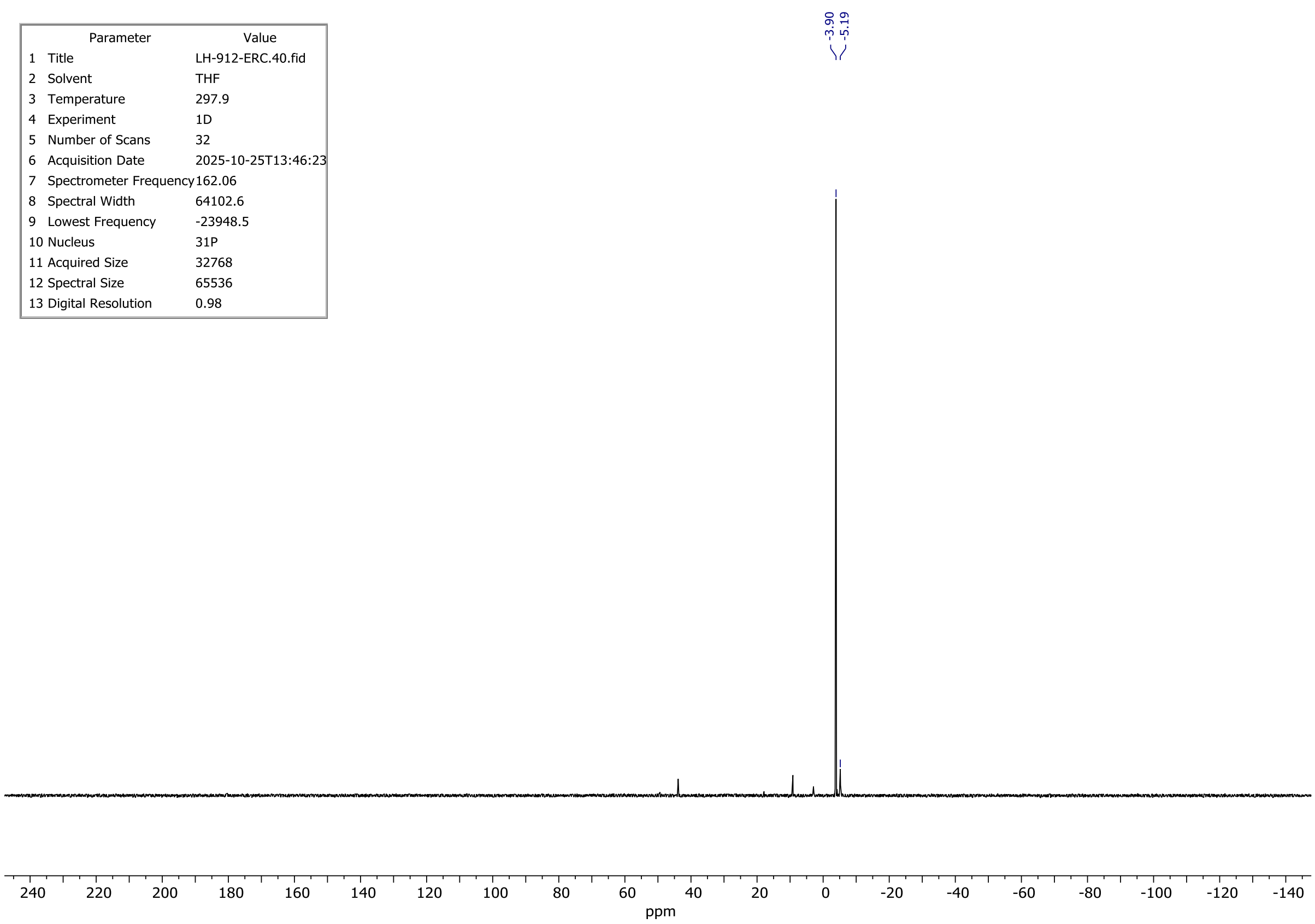

Figure S13: $^{31}P\{^1H\}$ NMR spectrum of a THF-d$_8$ solution of **6** with a concentration of 68.67 µmol/mL, 4 d after **4$_{\mathbf{ArF}}$Br** and LDA have been dissolved in the solvent.

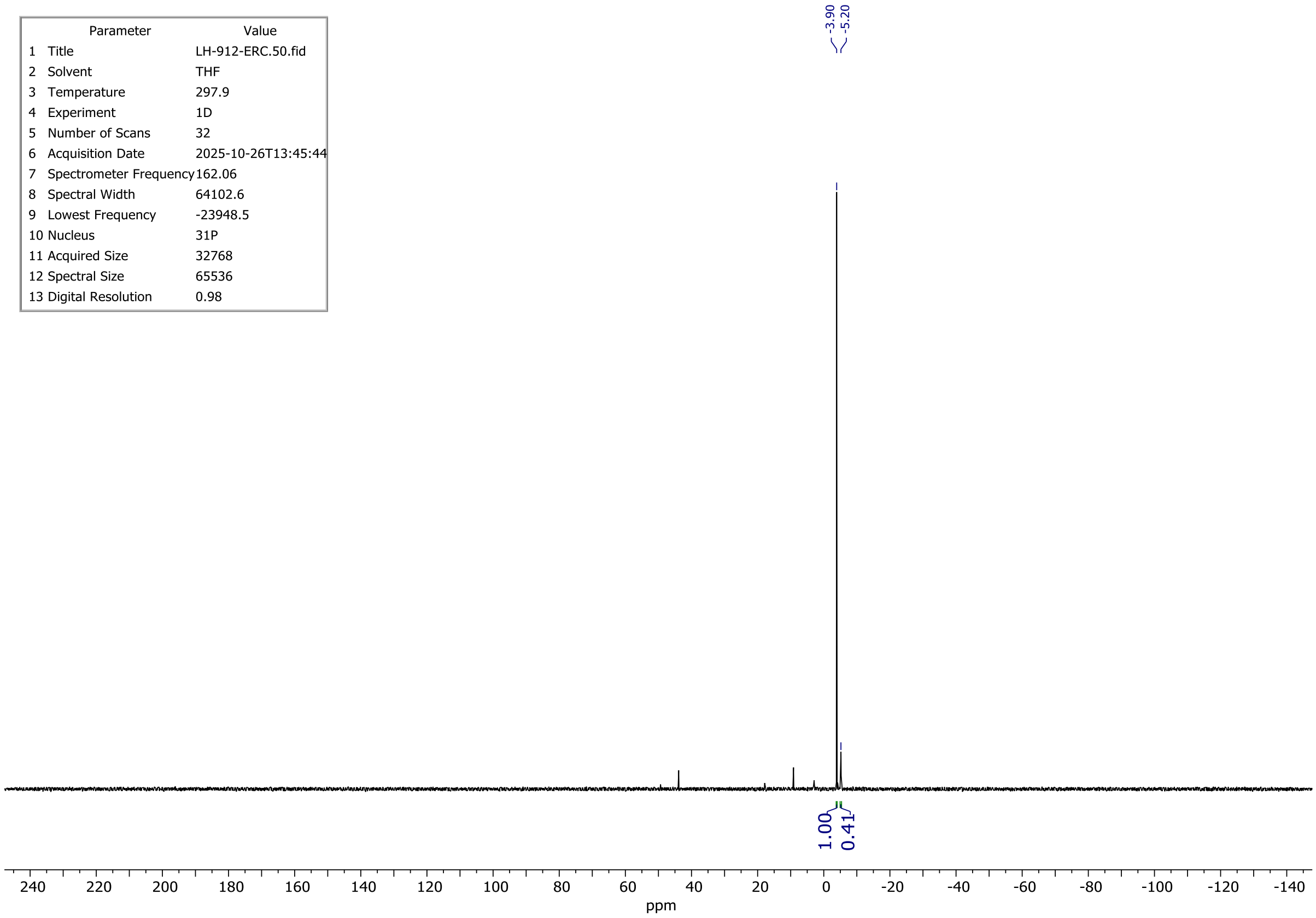

Figure S14: $^{31}P\{^1H\}$ NMR spectrum of a THF-d_8 solution of **6** with a concentration of 68.67 µmol/mL, 5 d after **$4_{Ar}FBr$** and LDA have been dissolved in the solvent.

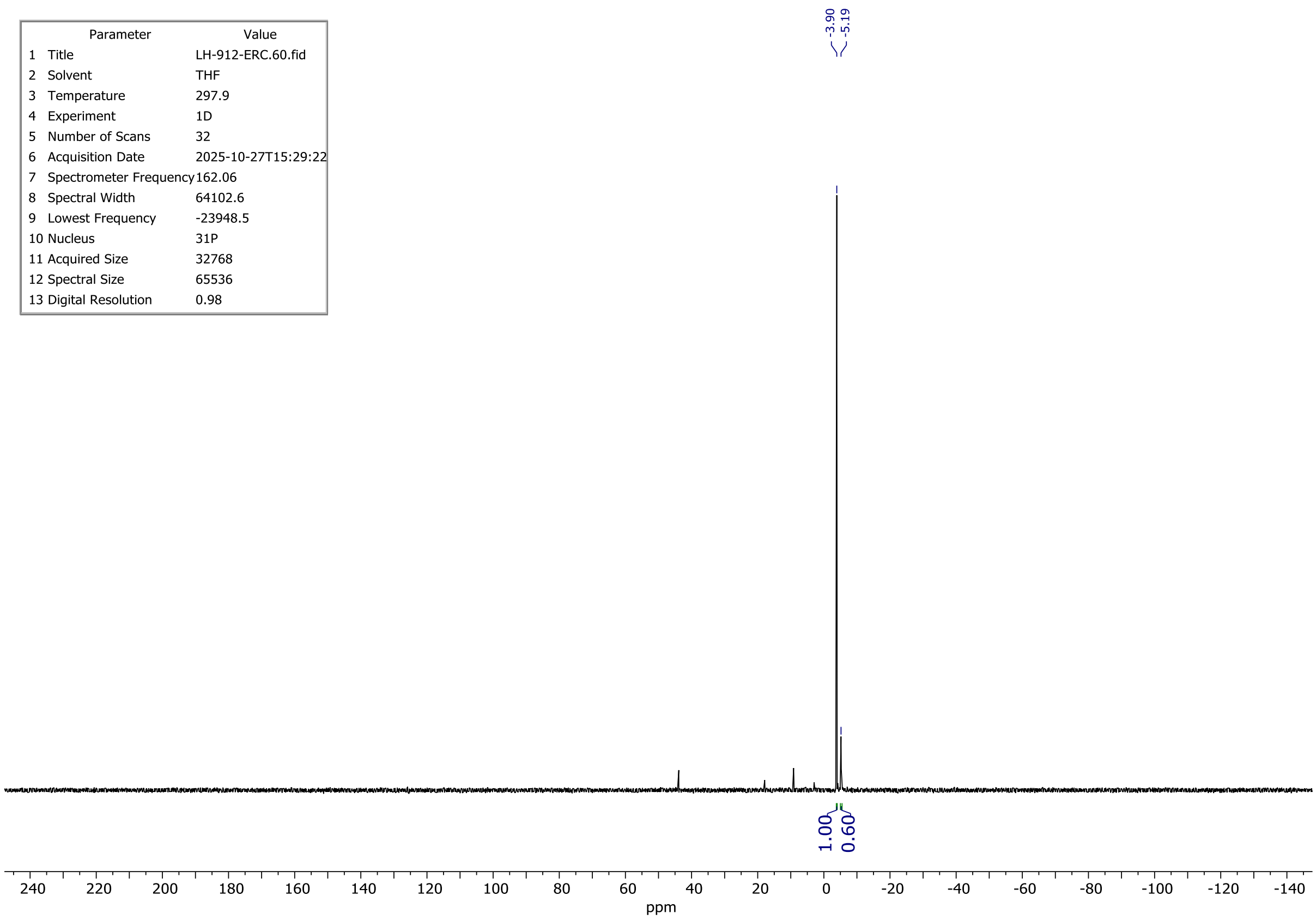

Figure S15: $^{31}P\{^1H\}$ NMR spectrum of a THF-d_8 solution of **6** with a concentration of 68.67 µmol/mL, 6 d after **$4_{Ar}FBr$** and LDA have been dissolved in the solvent.

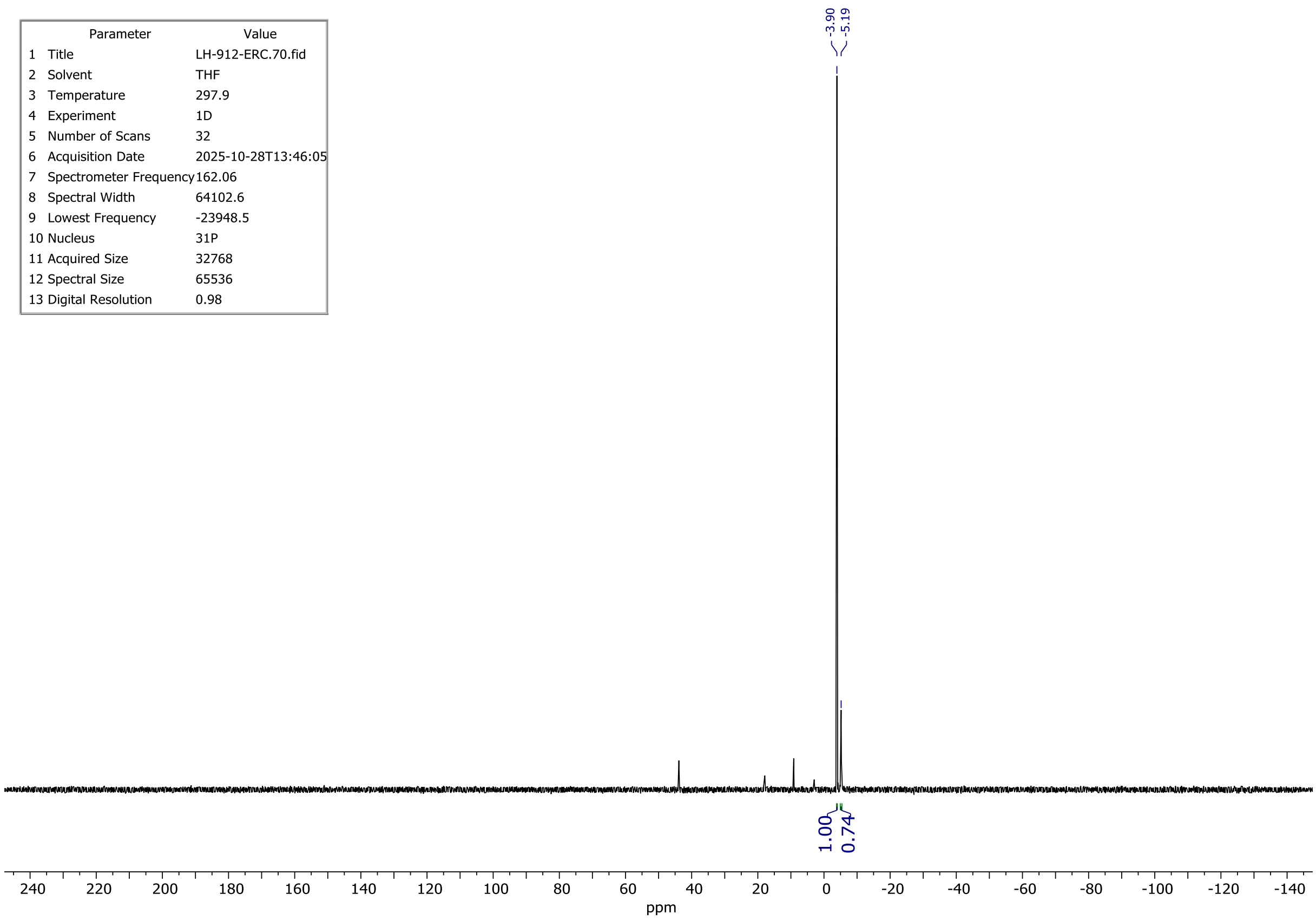

Figure S16: $^{31}P\{^1H\}$ NMR spectrum of a THF-d_8 solution of **6** with a concentration of 68.67 µmol/mL, 7 d after **4_{ArF}Br** and LDA have been dissolved in the solvent.

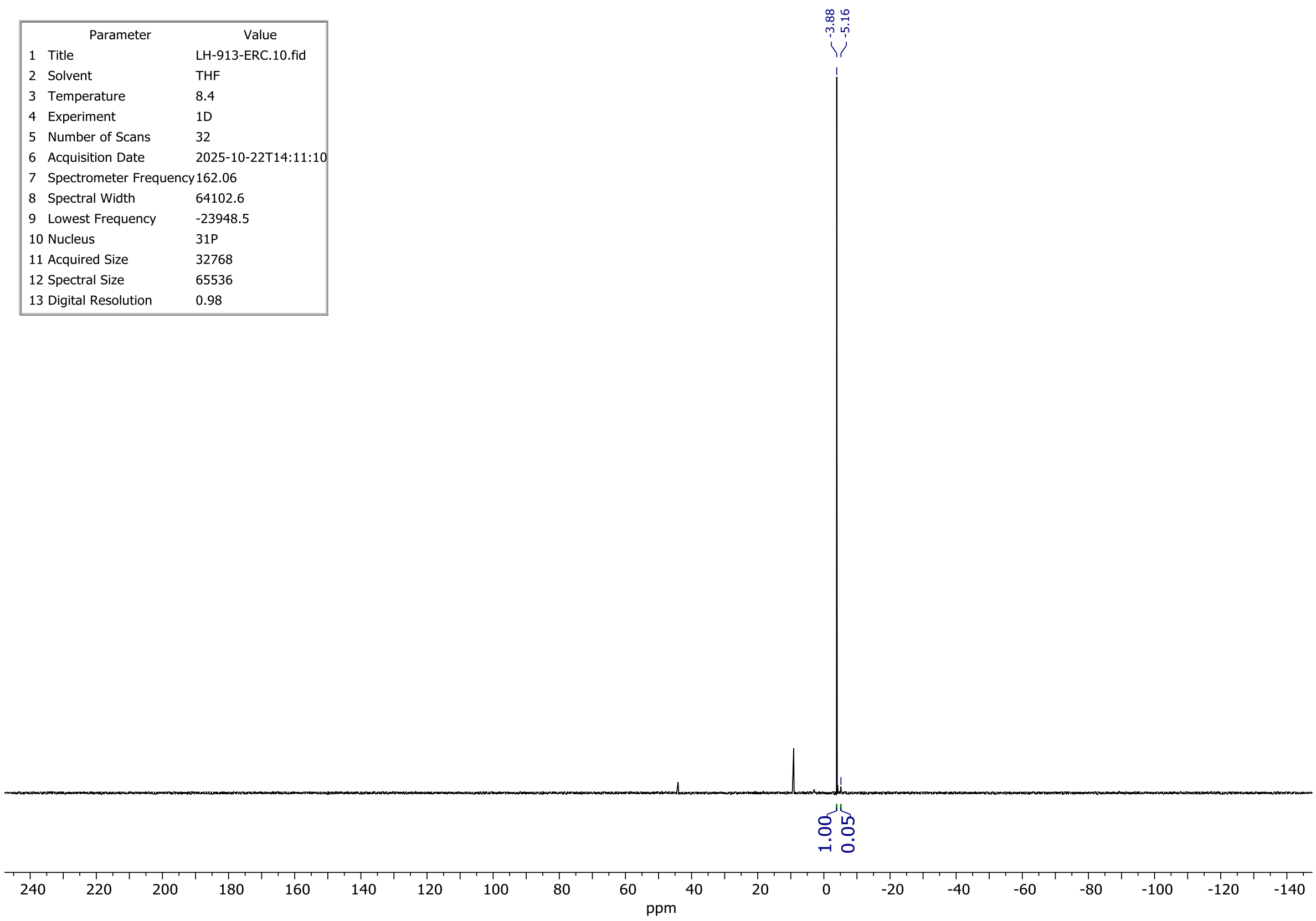

Figure S17: $^{31}P\{^1H\}$ NMR spectrum of a THF-d_8 solution of **6** with a concentration of 137.33 µmol/mL, 1 d after **4_{ArF}Br** and LDA have been dissolved in the solvent.

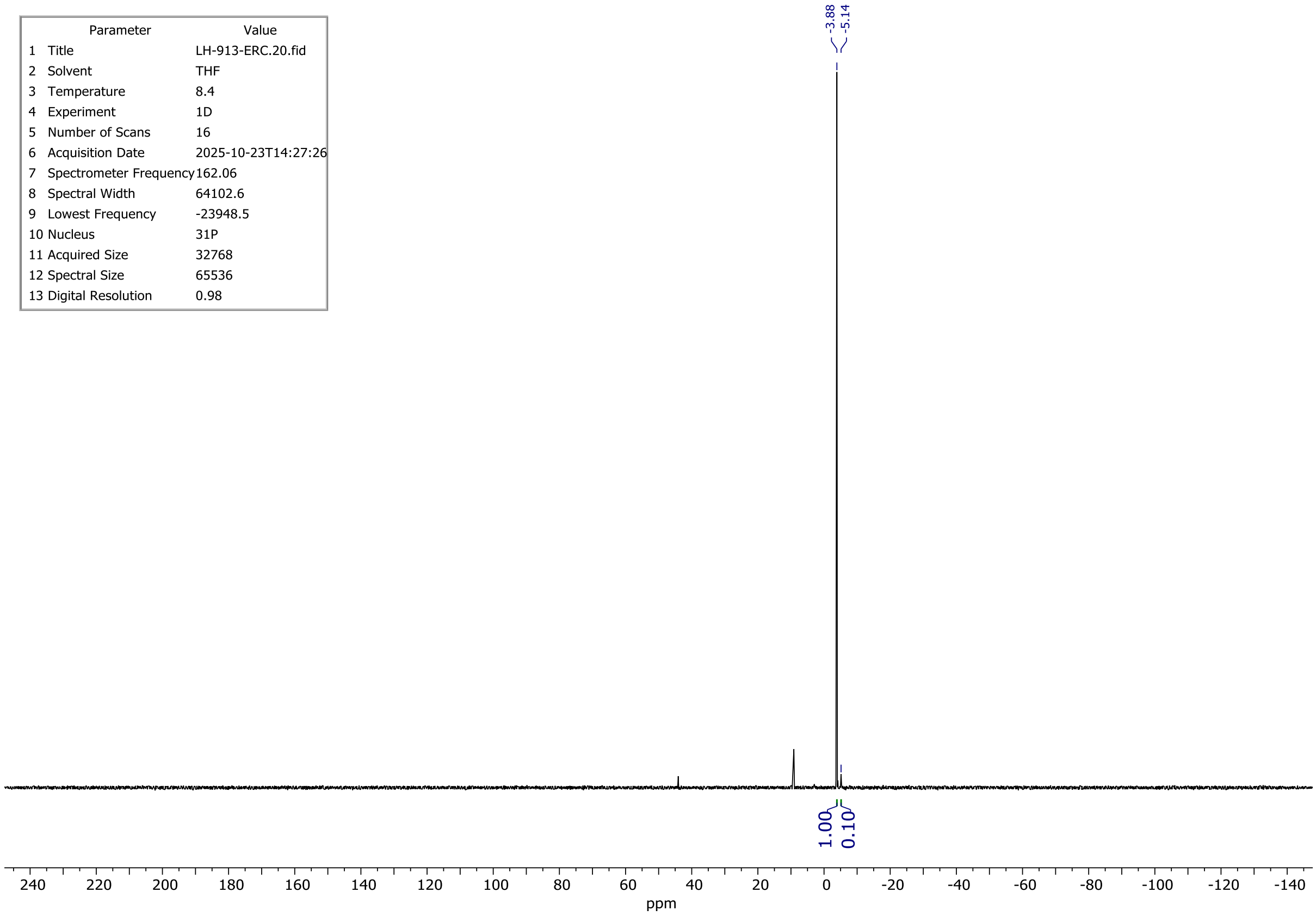

Figure S18: $^{31}P\{^{1}H\}$ NMR spectrum of a THF-d_8 solution of **6** with a concentration of 137.33 µmol/mL, 2 d after **4**$_{Ar}$**FBr** and LDA have been dissolved in the solvent.

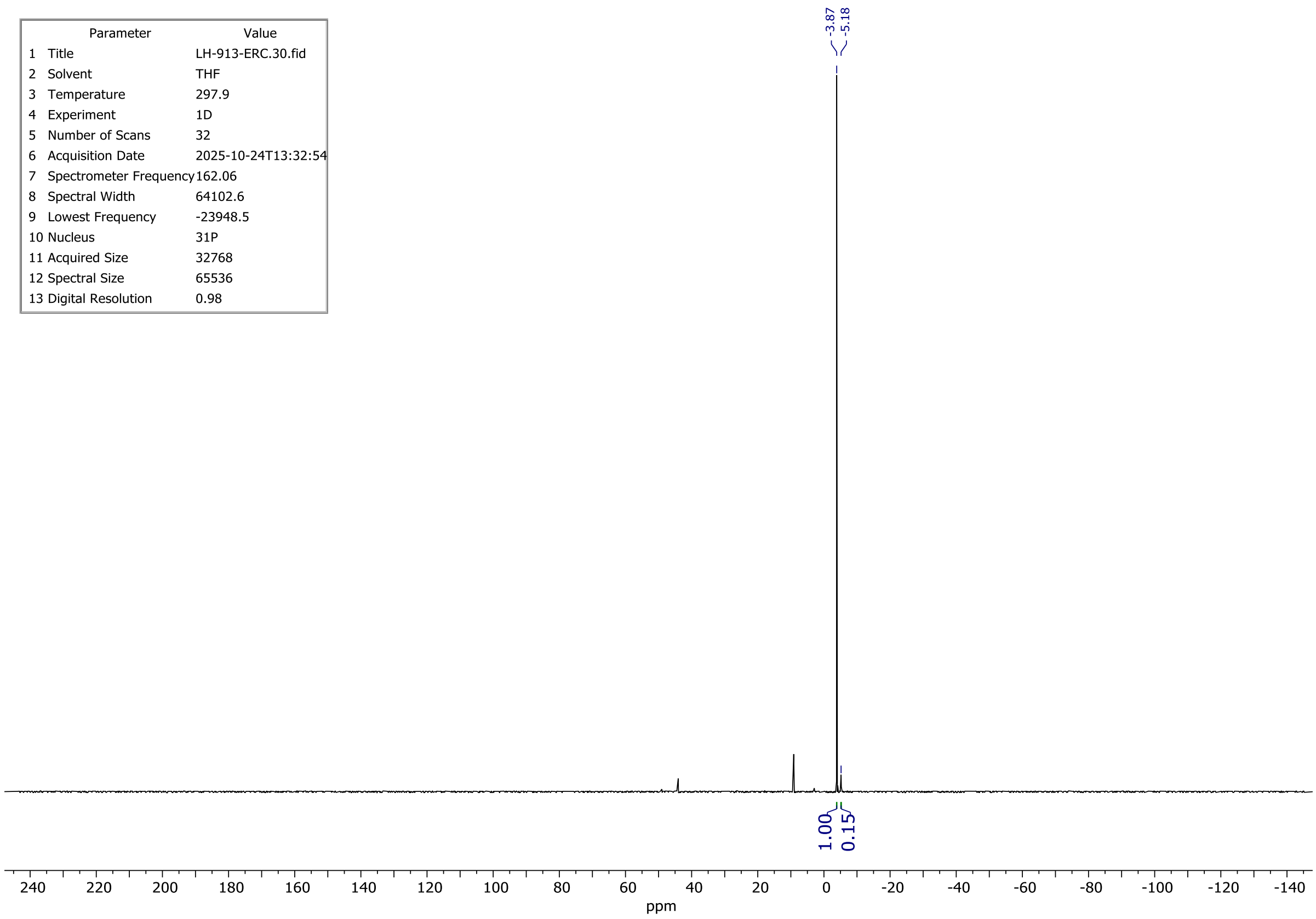

Figure S19: $^{31}P\{^{1}H\}$ NMR spectrum of a THF-d_8 solution of **6** with a concentration of 137.33 µmol/mL, 3 d after **4**$_{Ar}$**FBr** and LDA have been dissolved in the solvent.

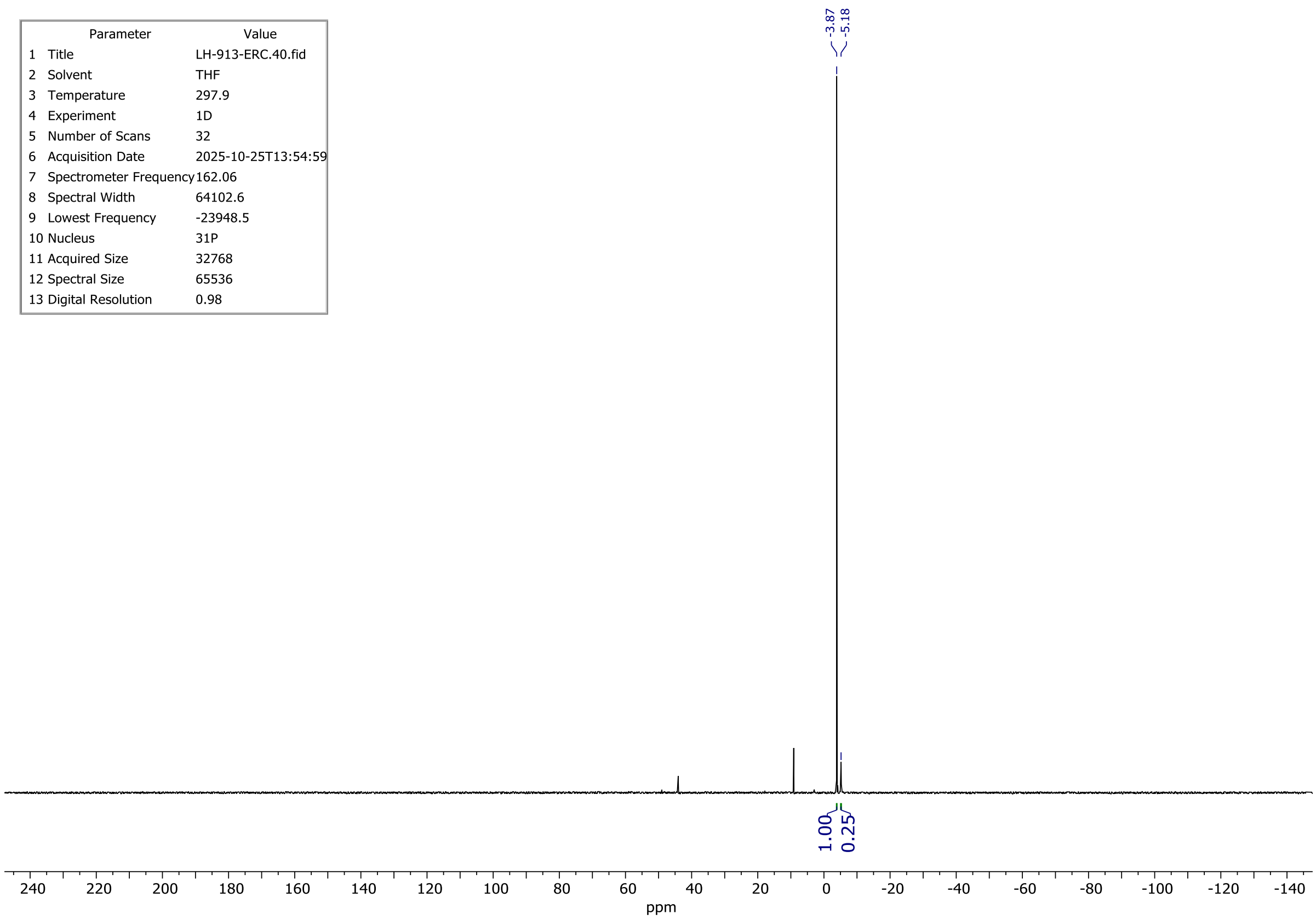

Figure S20: ^{31}P{^{1}H} NMR spectrum of a THF-d_8 solution of **6** with a concentration of 137.33 µmol/mL, 4 d after **4**$_{ArF}$**Br** and LDA have been dissolved in the solvent.

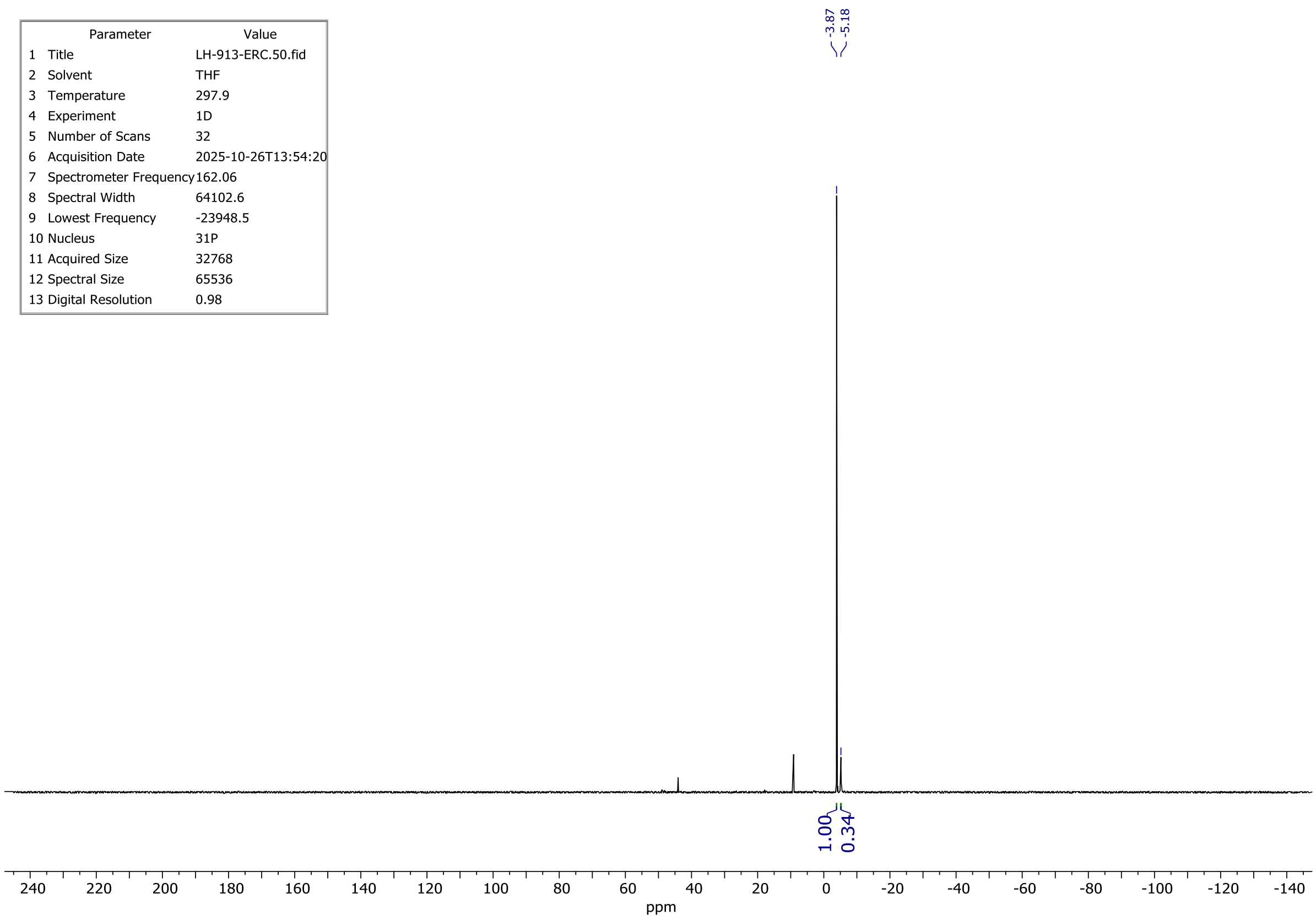

Figure S21: ^{31}P{^{1}H} NMR spectrum of a THF-d_8 solution of **6** with a concentration of 137.33 µmol/mL, 5 d after **4**$_{ArF}$**Br** and LDA have been dissolved in the solvent.

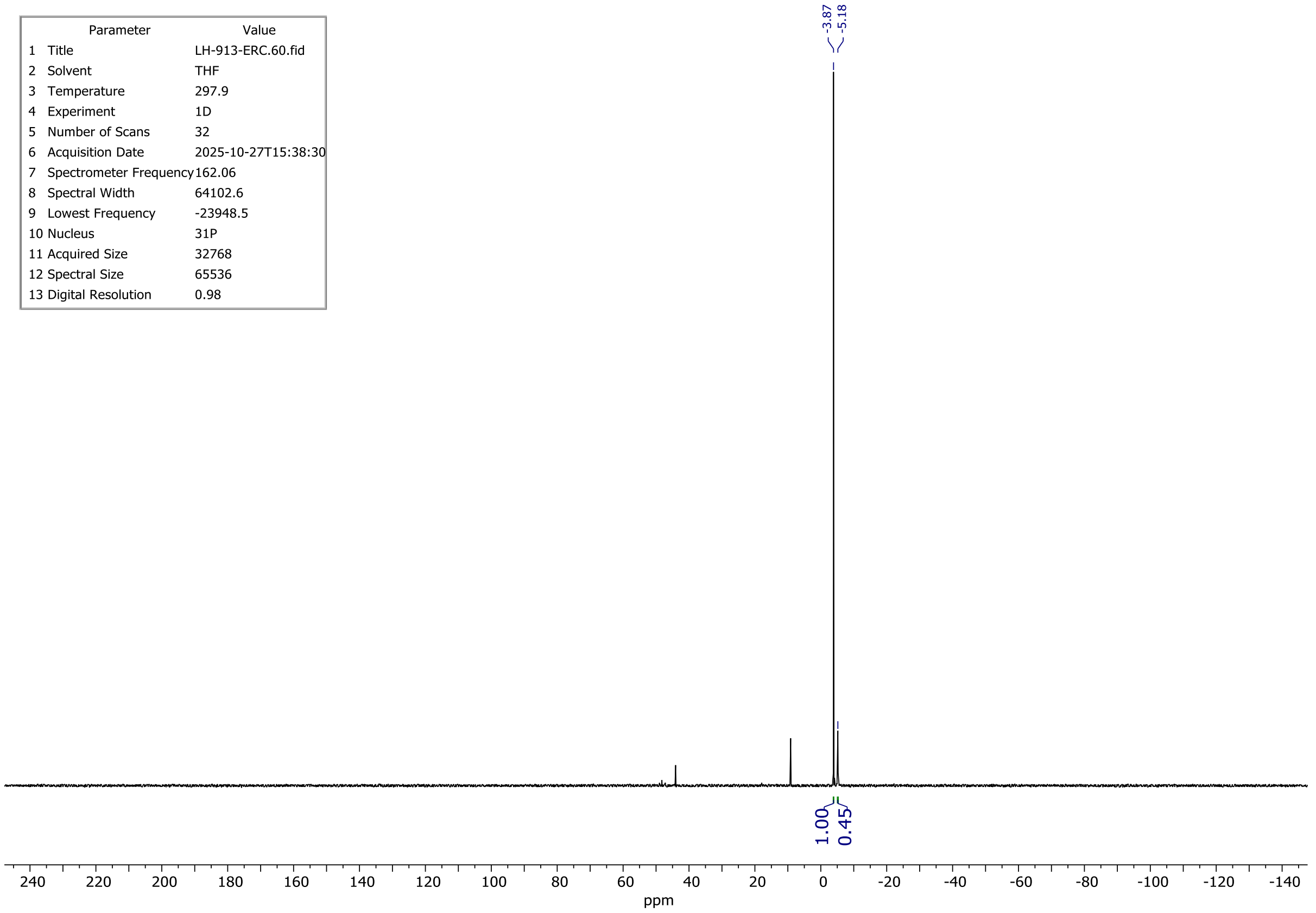

Figure S22: $^{31}P\{^1H\}$ NMR spectrum of a THF-d_8 solution of **6** with a concentration of 137.33 µmol/mL, 6 d after **4_{ArF}Br** and LDA have been dissolved in the solvent.

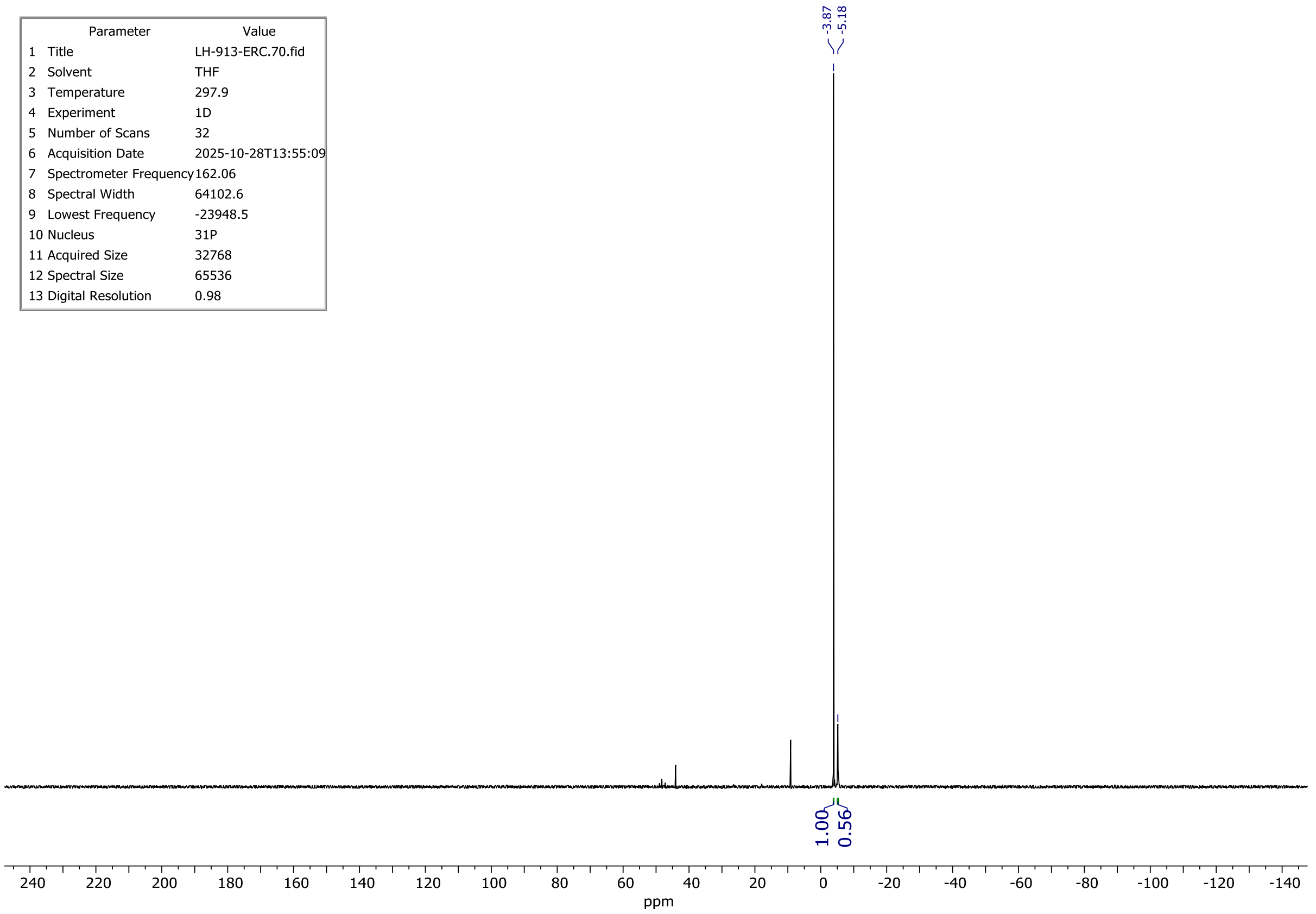

Figure S23: $^{31}P\{^1H\}$ NMR spectrum of a THF-d_8 solution of **6** with a concentration of 137.33 µmol/mL, 7 d after **4_{ArF}Br** and LDA have been dissolved in the solvent.

5 NMR spectra of the isolated compounds

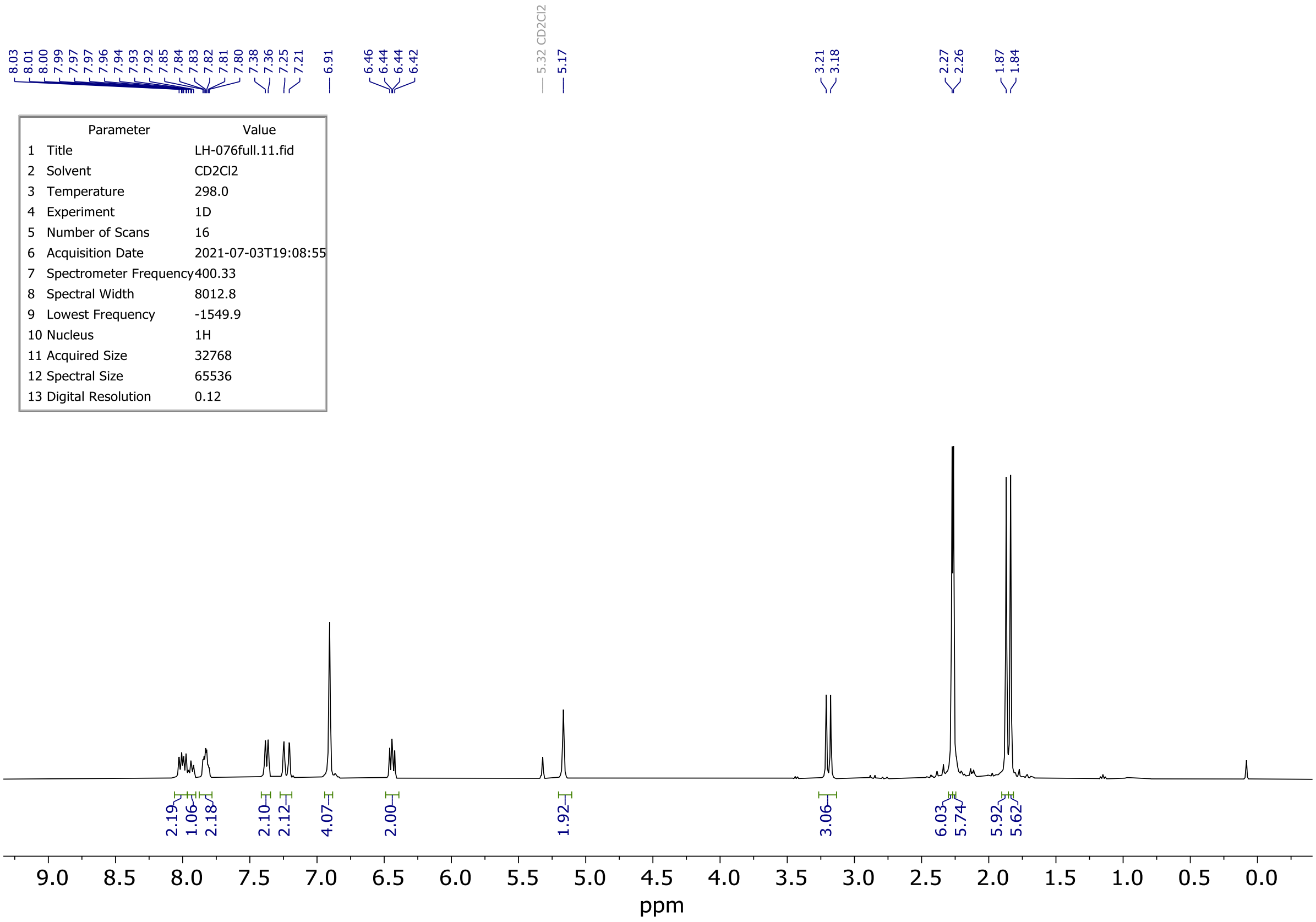

Figure S24: ^{1}H NMR spectrum of $\mathbf{2_{H}I}$ in CD_2Cl_2.

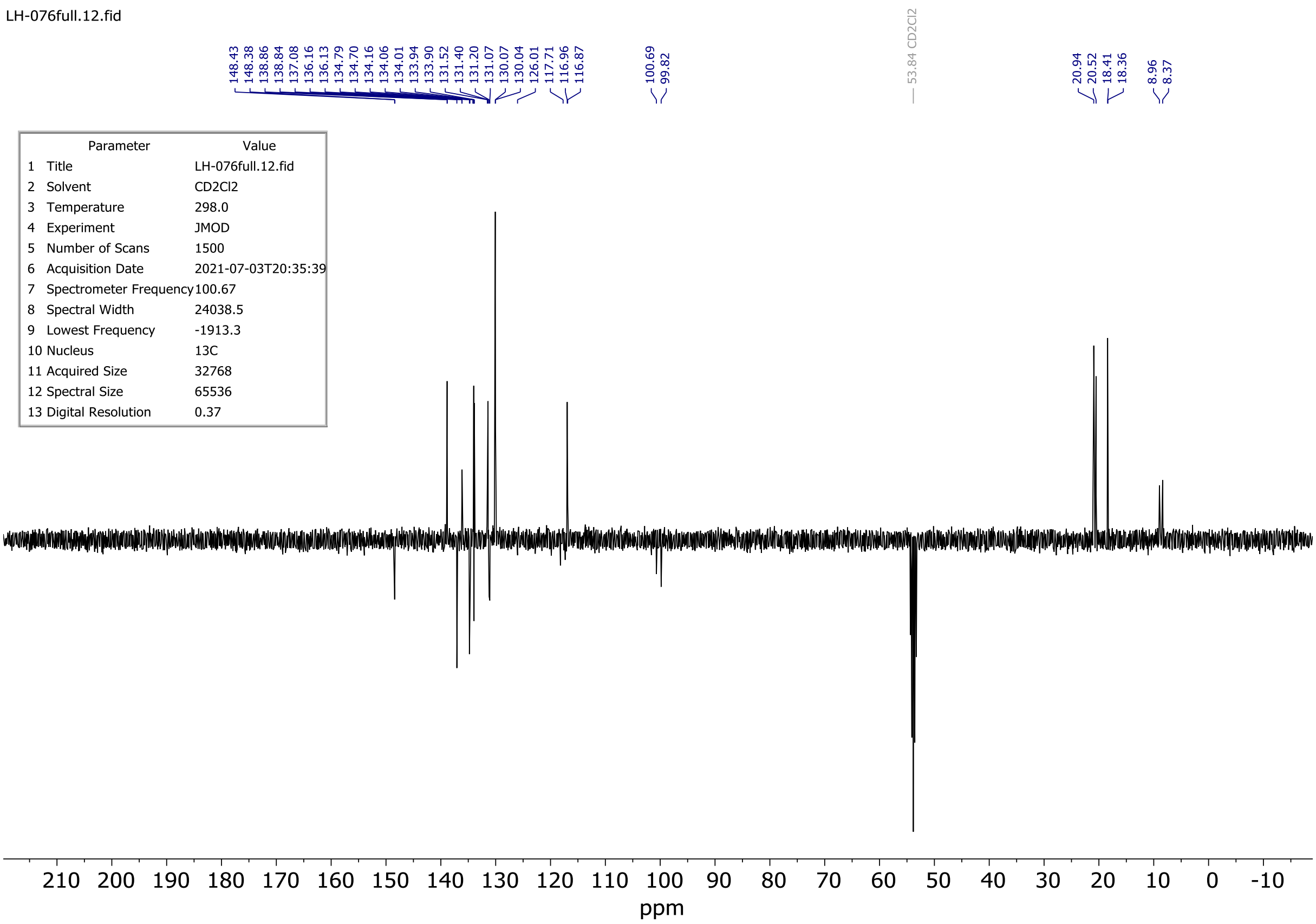

Figure S25: $^{13}C\{^{1}H\}$-APT NMR spectrum of $\mathbf{2_{H}I}$ in CD_2Cl_2.

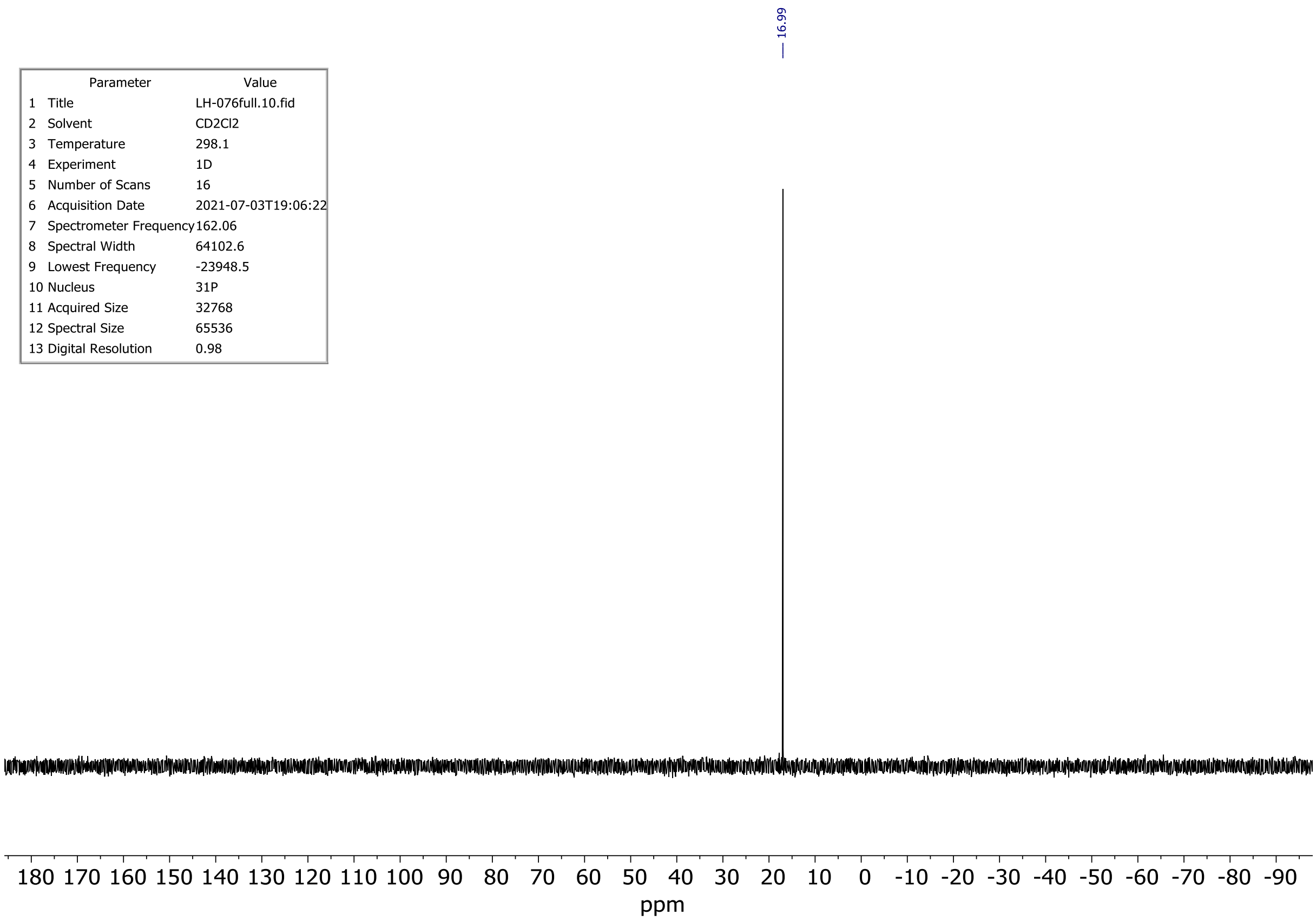

Figure S26: $^{31}P\{^{1}H\}$ NMR spectrum of $\mathbf{2_{H}I}$ in CD_2Cl_2.

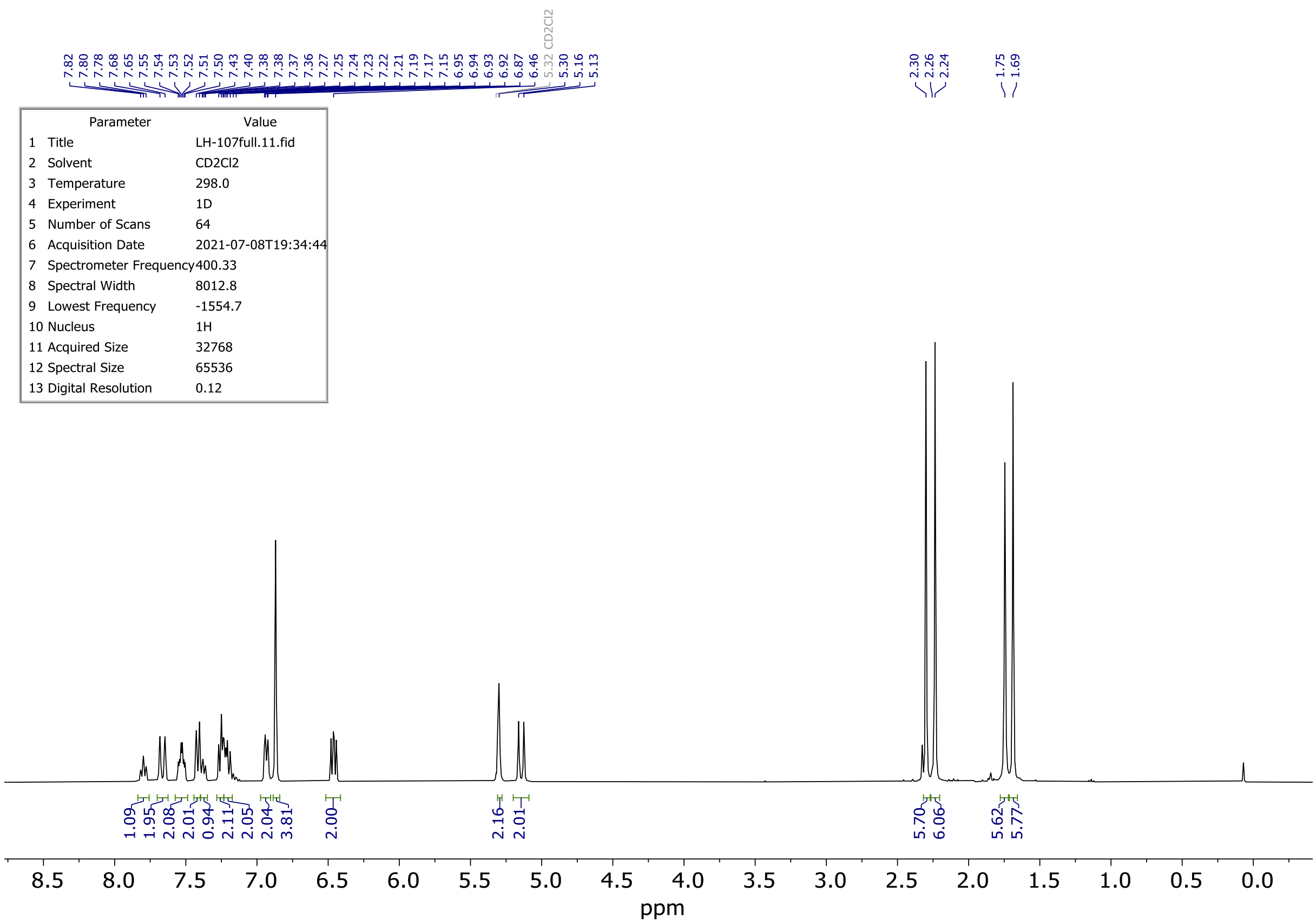

Figure S27: ^{1}H NMR spectrum of $\mathbf{2_{Ph}I}$ in CD_2Cl_2.

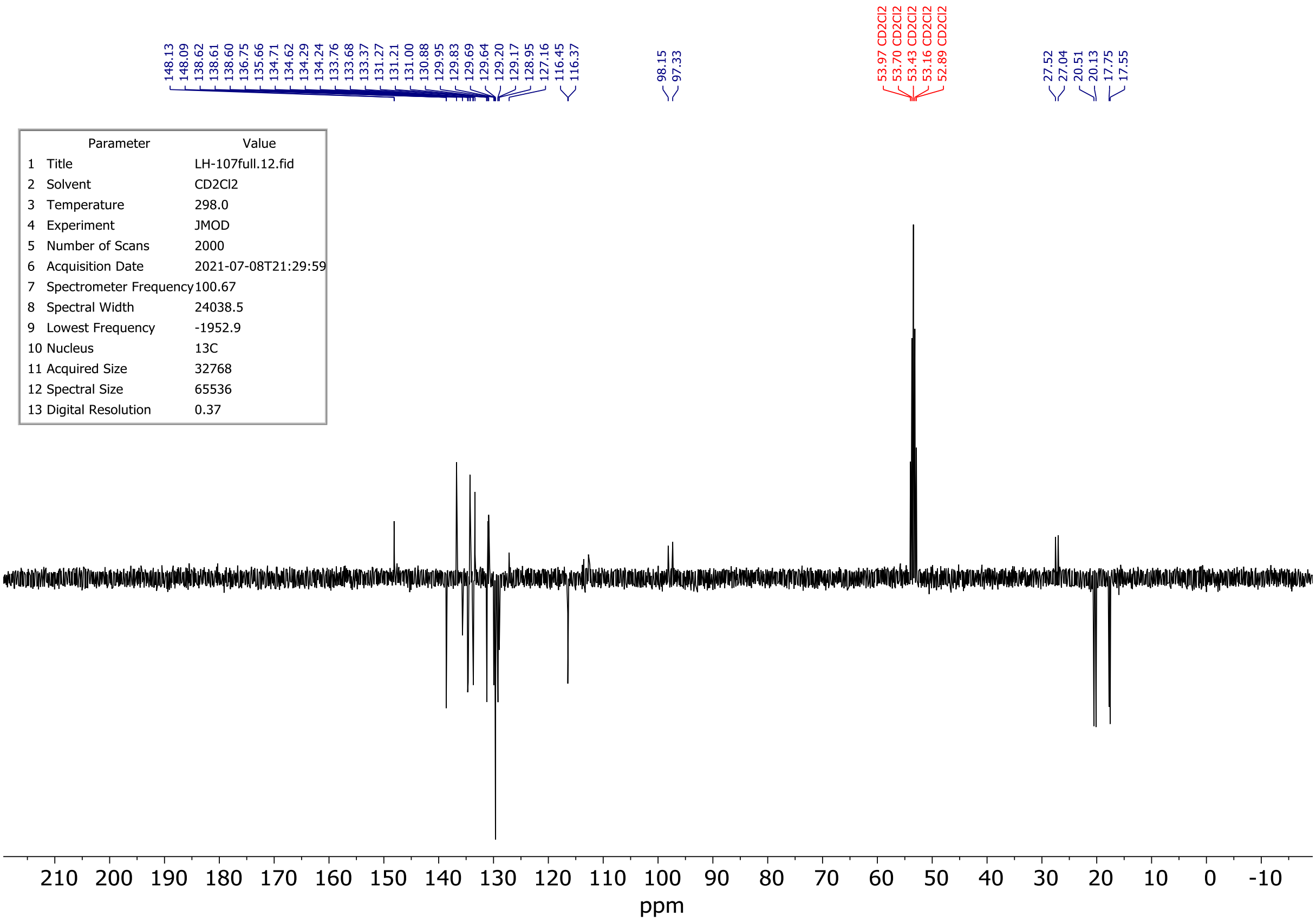

Figure S28: $^{13}C\{^{1}H\}$-APT NMR spectrum of $\mathbf{2_{Ph}I}$ in CD_2Cl_2.

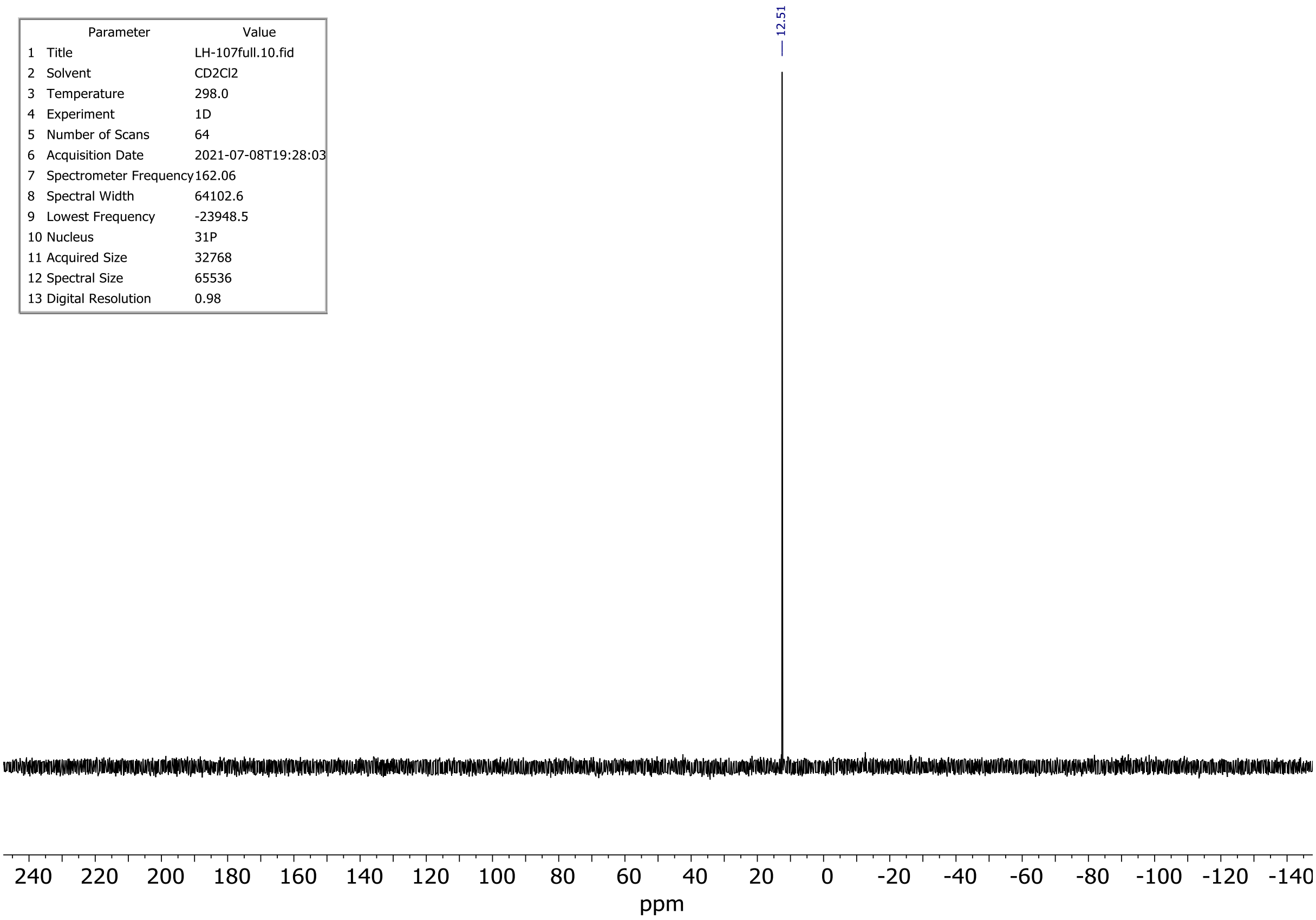

Figure S29: $^{31}P\{^{1}H\}$ NMR spectrum of $\mathbf{2_{Ph}I}$ in CD_2Cl_2.

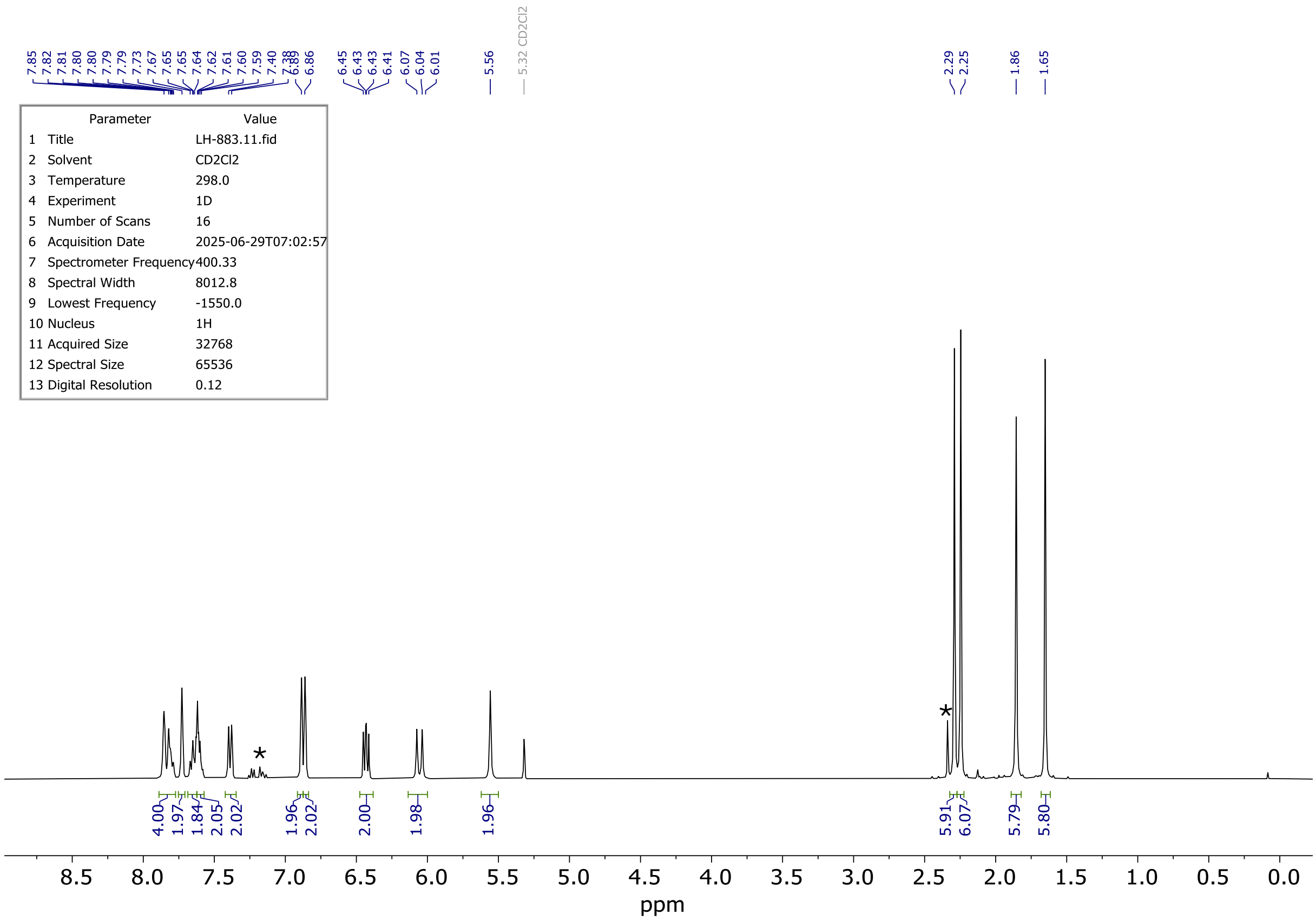

Figure S30: ^{1}H NMR spectrum of **2_{ArF}Br** in CD_2Cl_2. The * highlights the signals of residual toluene.

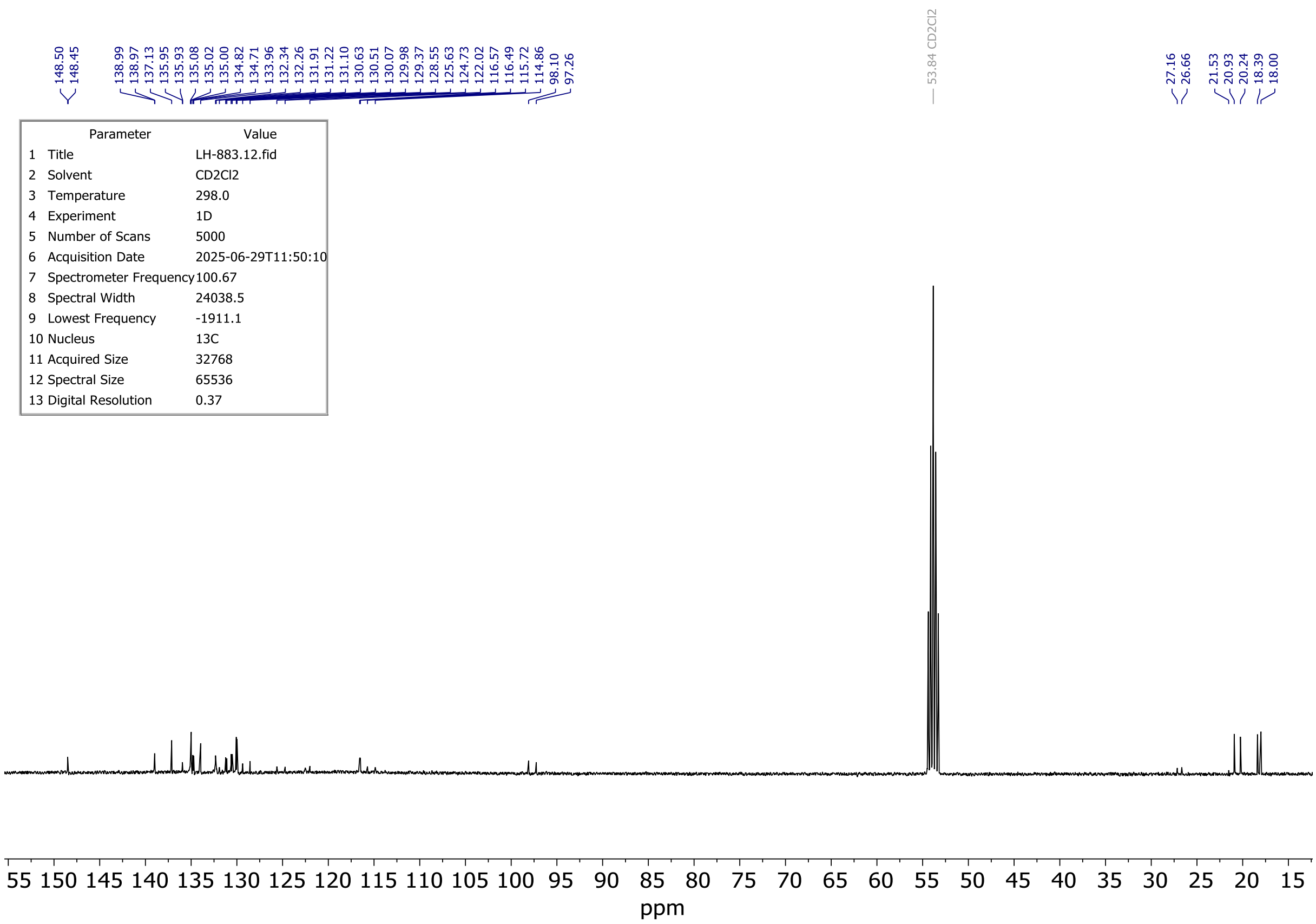

Figure S31: $^{13}C\{^{1}H\}$ NMR spectrum of **2_{ArF}Br** in CD_2Cl_2.

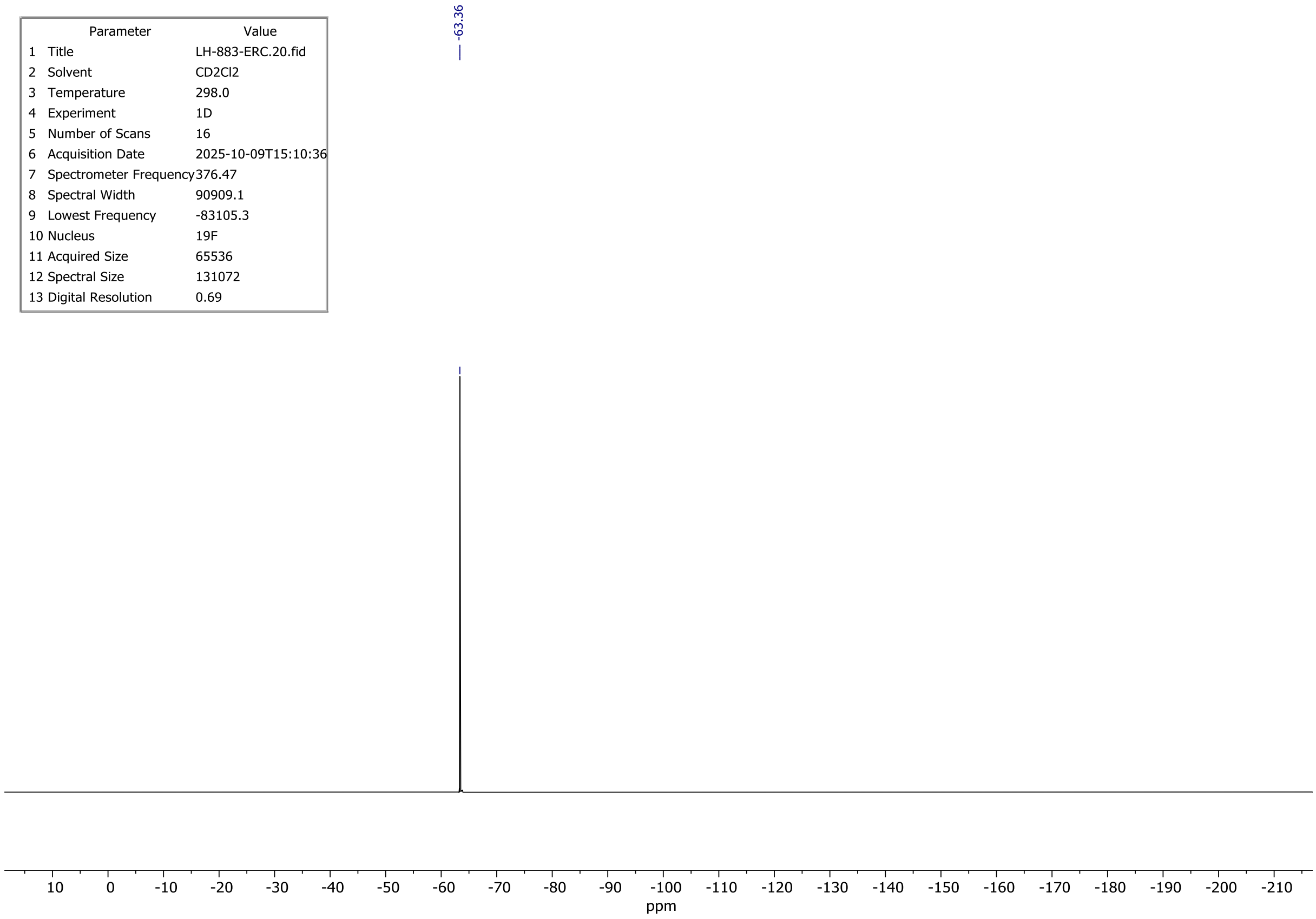

Figure S32: $^{19}F\{^{1}H\}$ NMR spectrum of **2_{ArF}Br** in CD_2Cl_2.

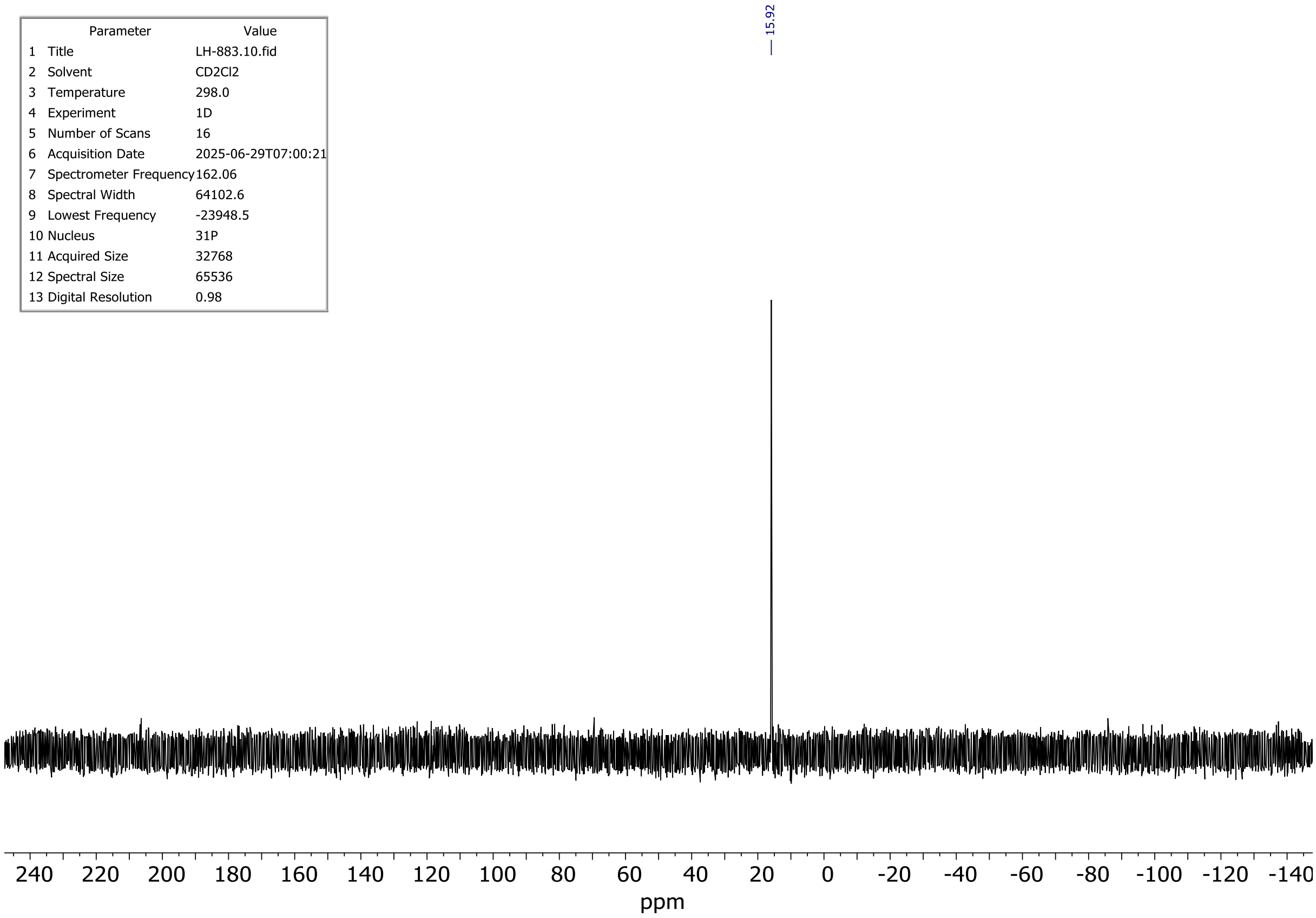

Figure S33: $^{31}P\{^{1}H\}$ NMR spectrum of **2_{ArF}Br** in CD_2Cl_2.

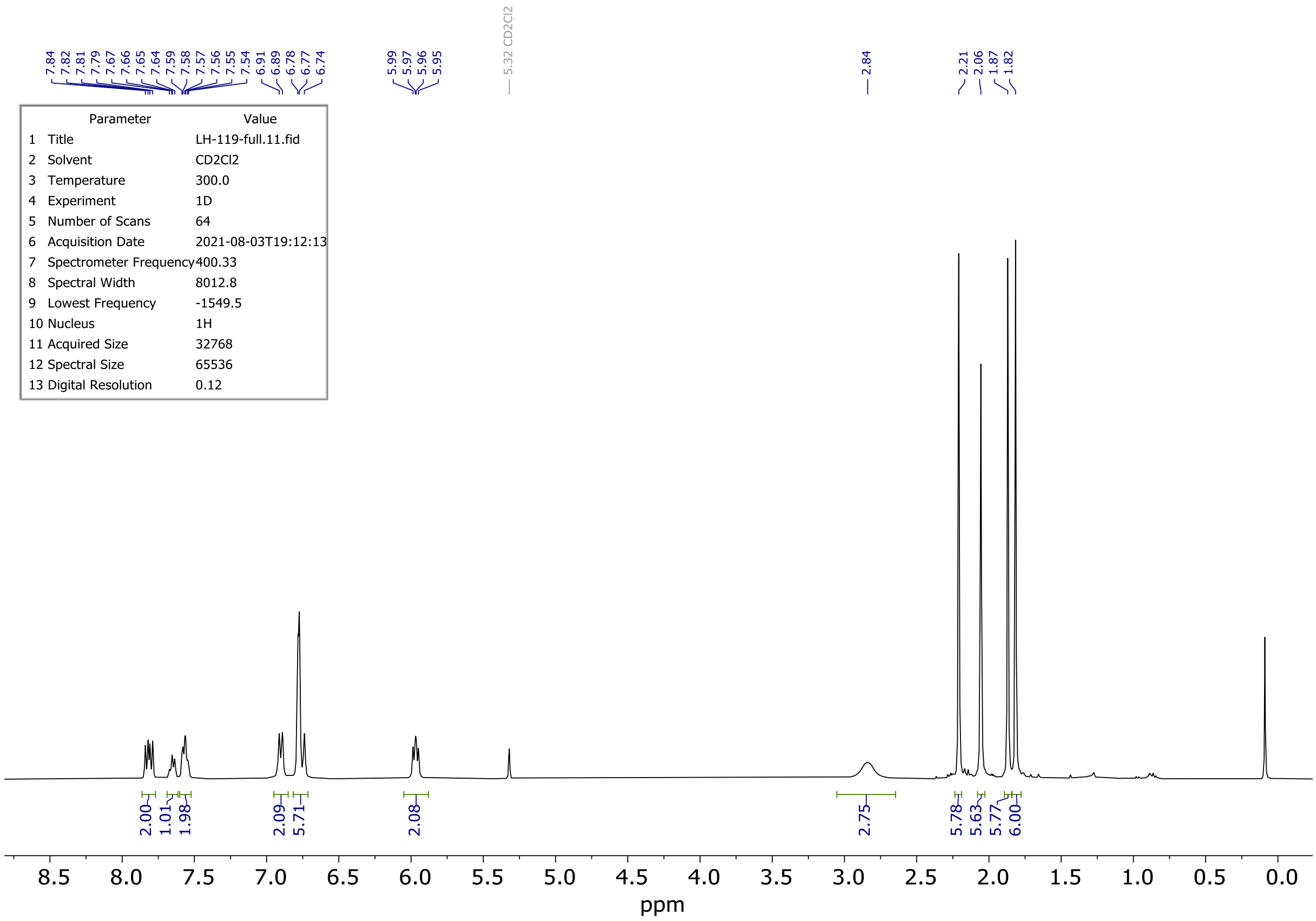

Figure S34: ^{1}H NMR spectrum of **3_H** in CD_2Cl_2.

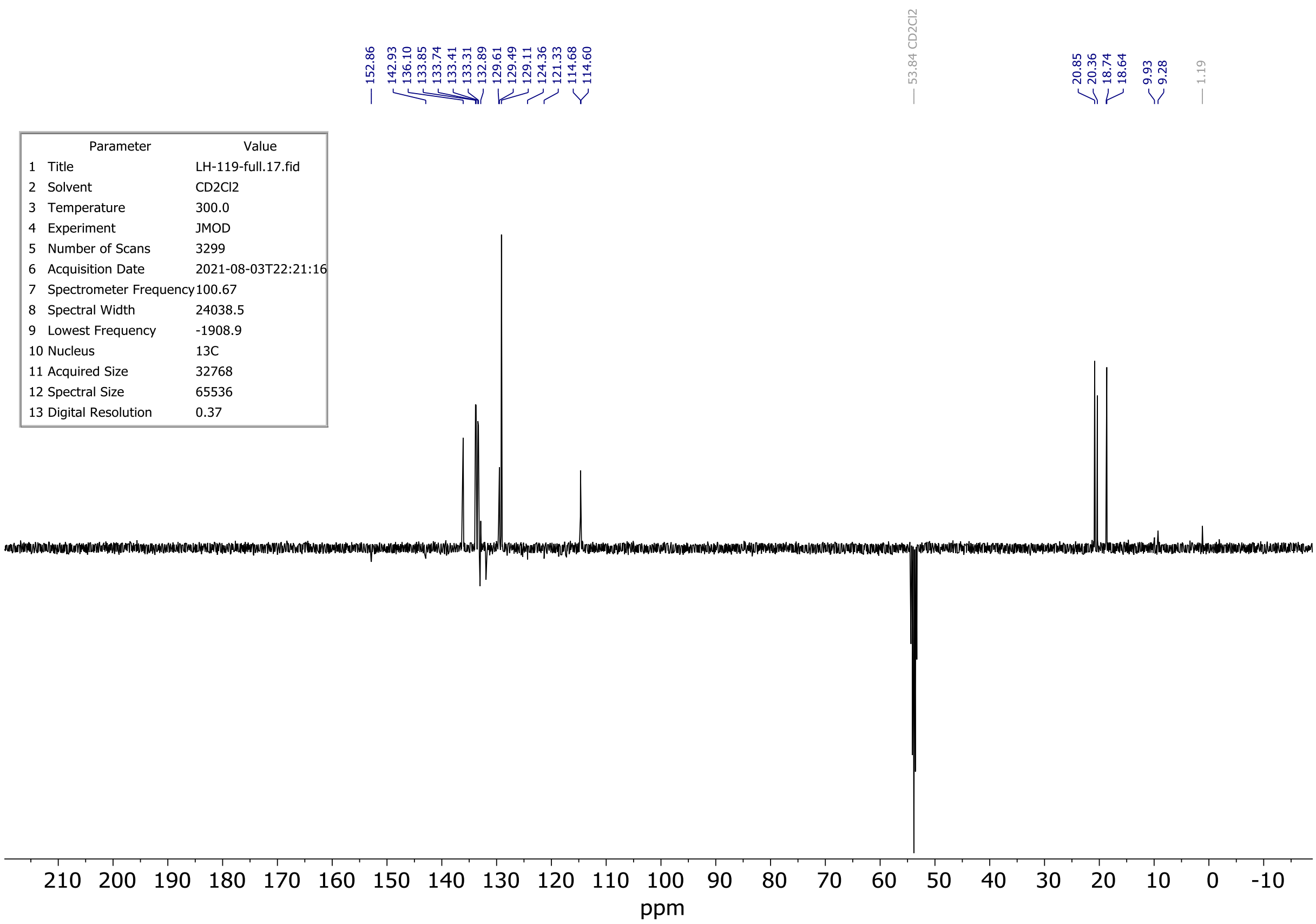

Figure S35: $^{13}C\{^1H\}$-APT NMR spectrum of **3_H** in CD_2Cl_2.

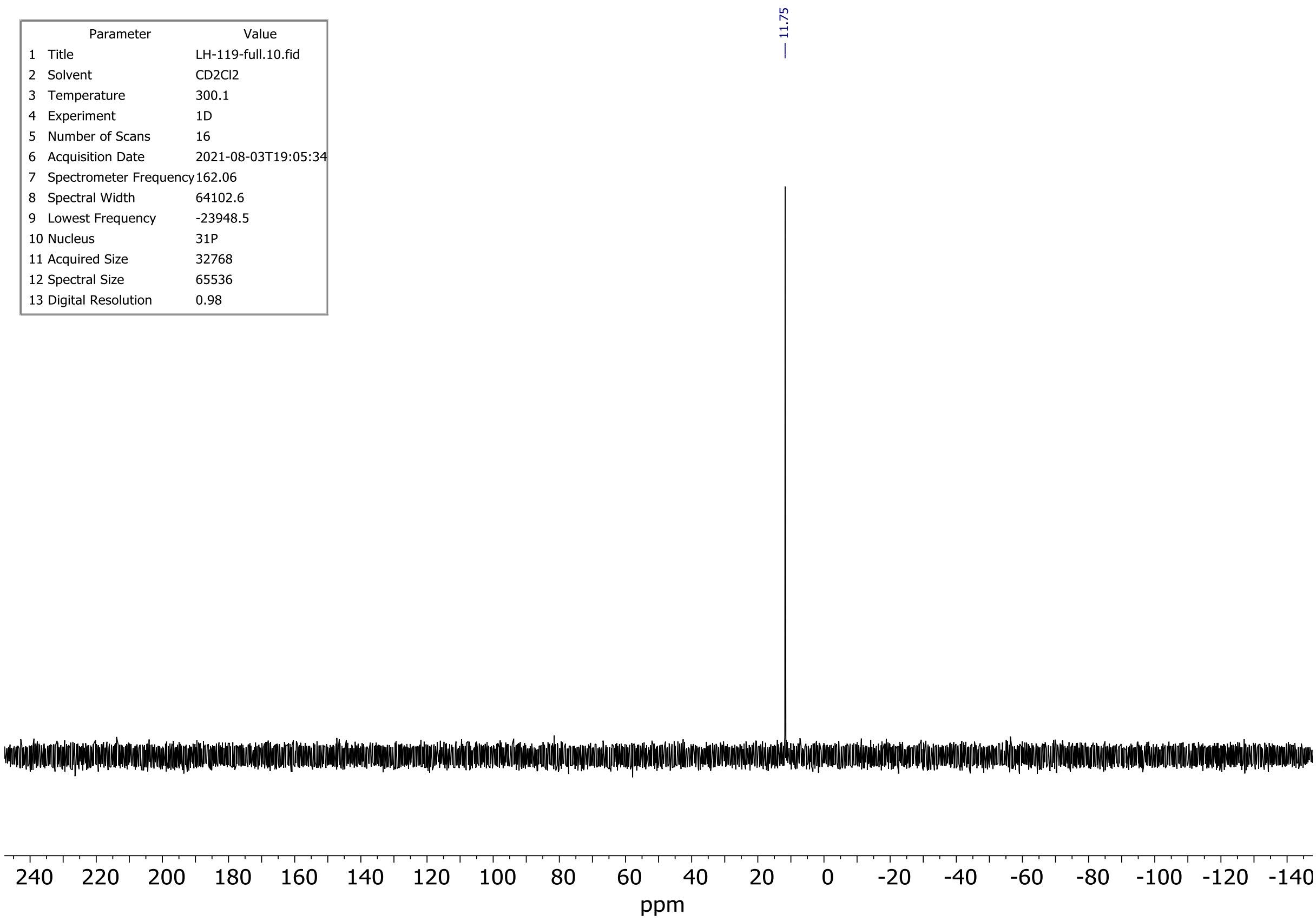

Figure S36: $^{31}P\{^{1}H\}$ NMR spectrum of $\mathbf{3_H}$ in CD_2Cl_2.

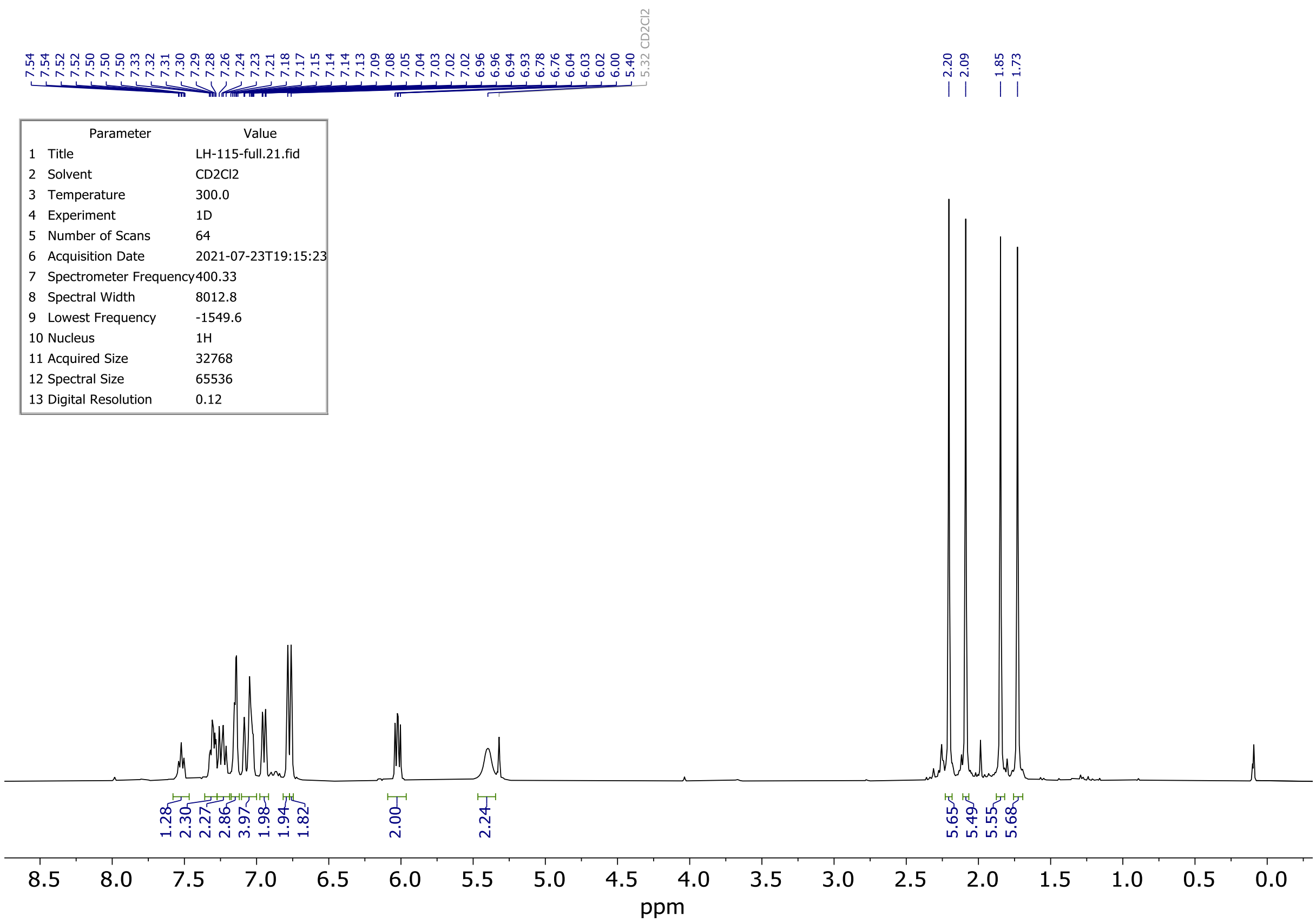

Figure S37: ^{1}H NMR spectrum of $\mathbf{3_{Ph}}$ in CD_2Cl_2.

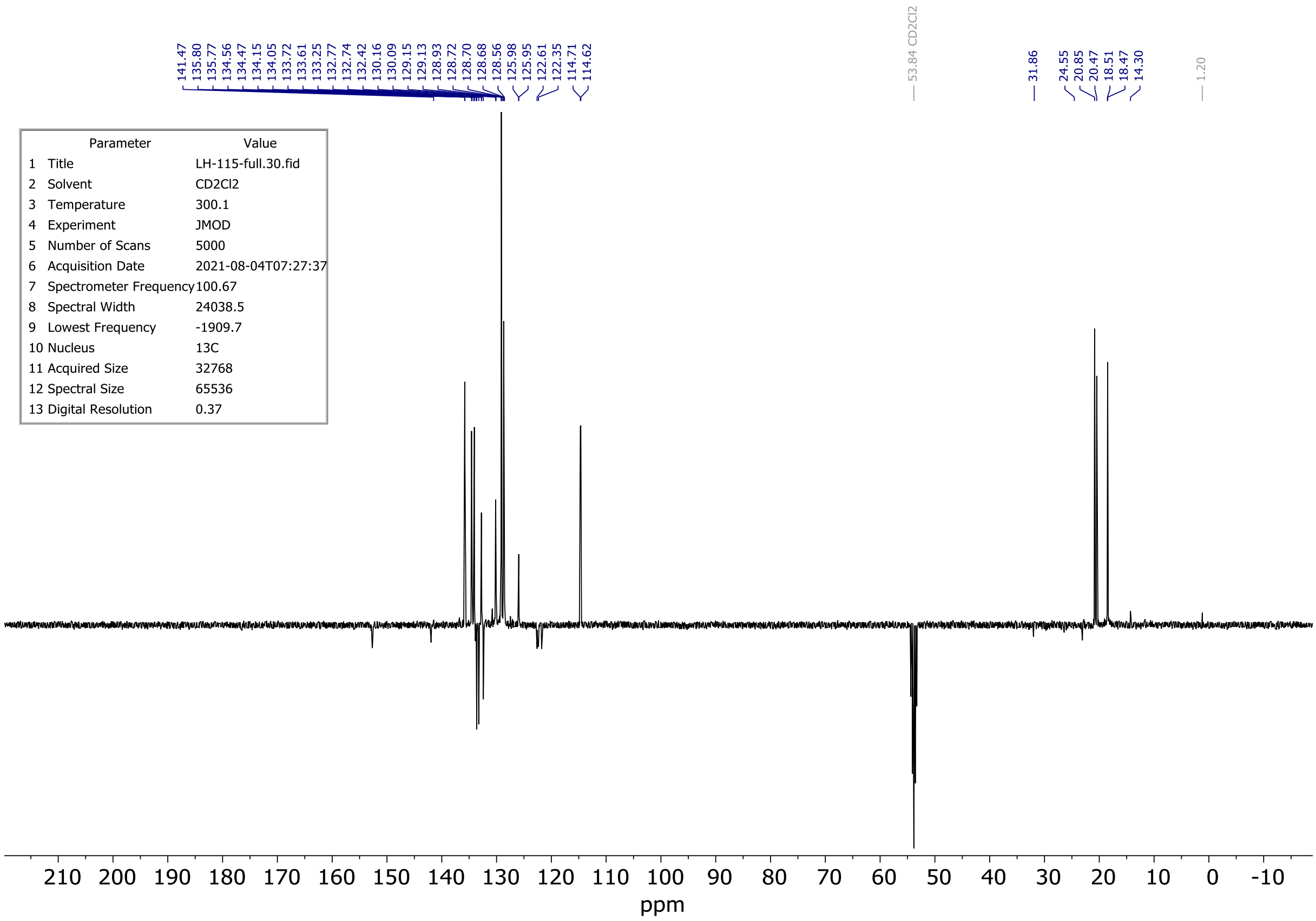

Figure S38: $^{13}C\{^{1}H\}$-APT NMR spectrum of $\mathbf{3_{Ph}}$ in CD_2Cl_2.

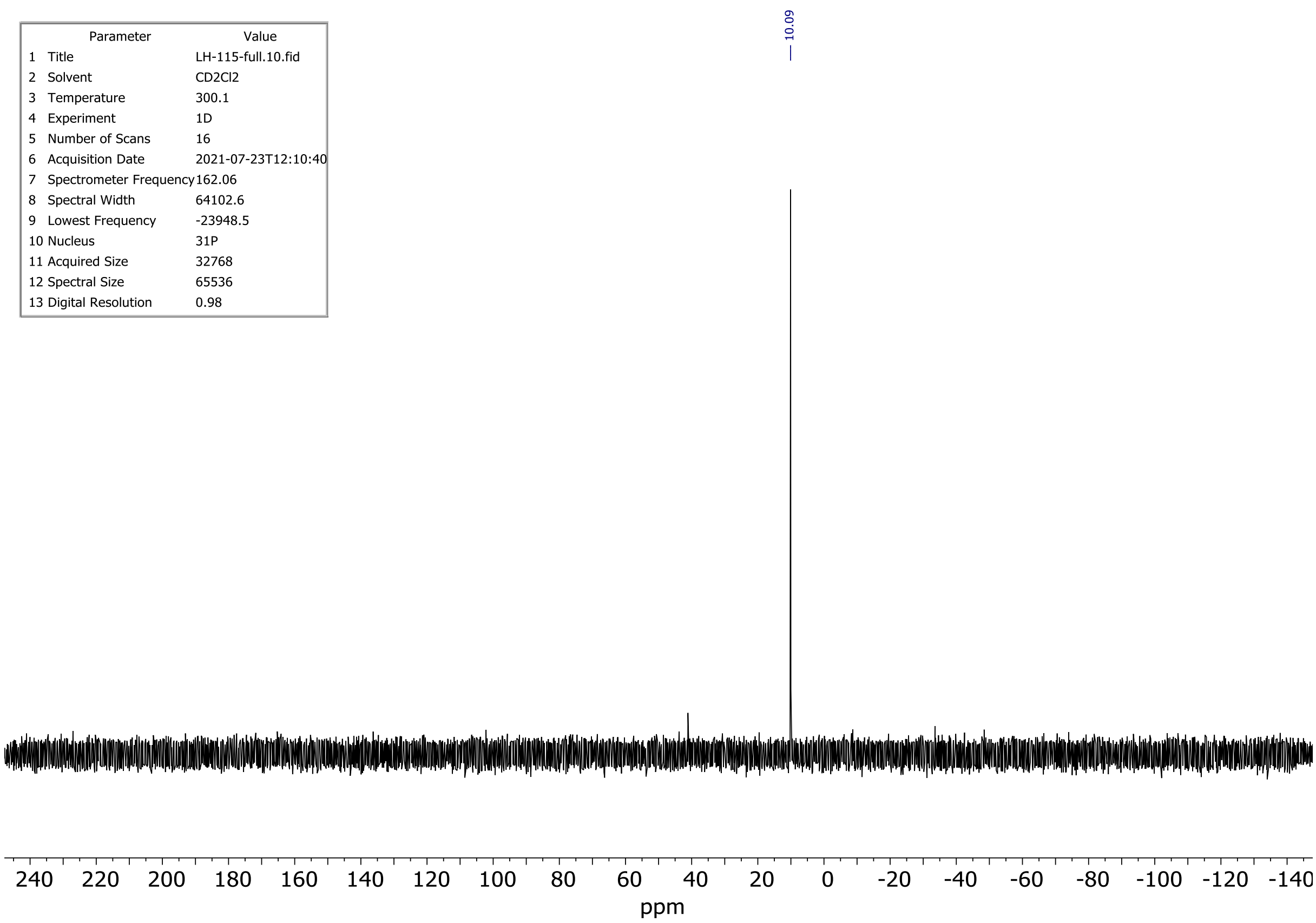

Figure S39: $^{31}P\{^{1}H\}$ NMR spectrum of $\mathbf{3_{Ph}}$ in CD_2Cl_2.

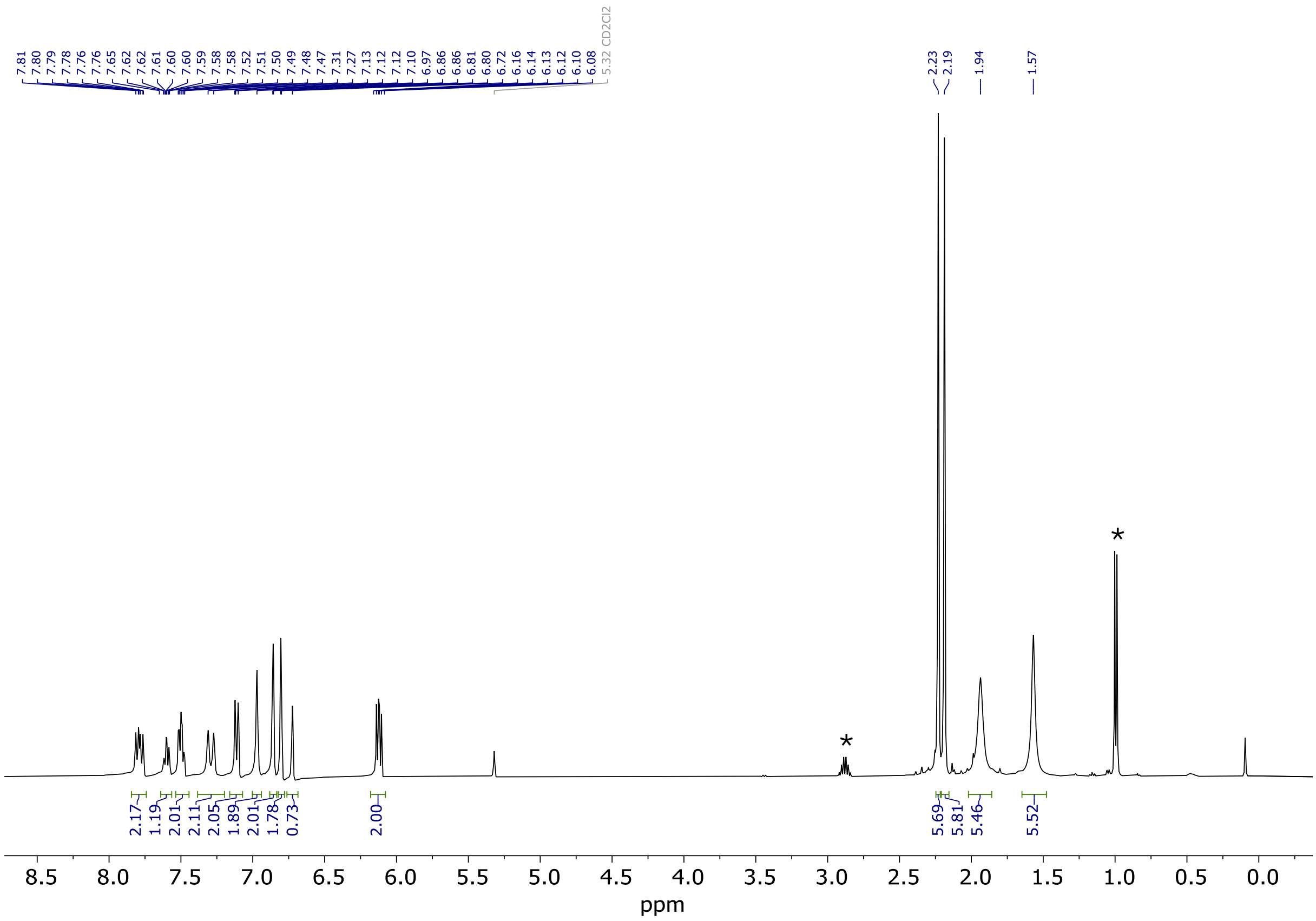

Figure S40: ^{1}H NMR spectrum of **3**$_{\mathbf{ArF}}$ in CD_2Cl_2 at RT. The * highlights the signals of residual LDA.

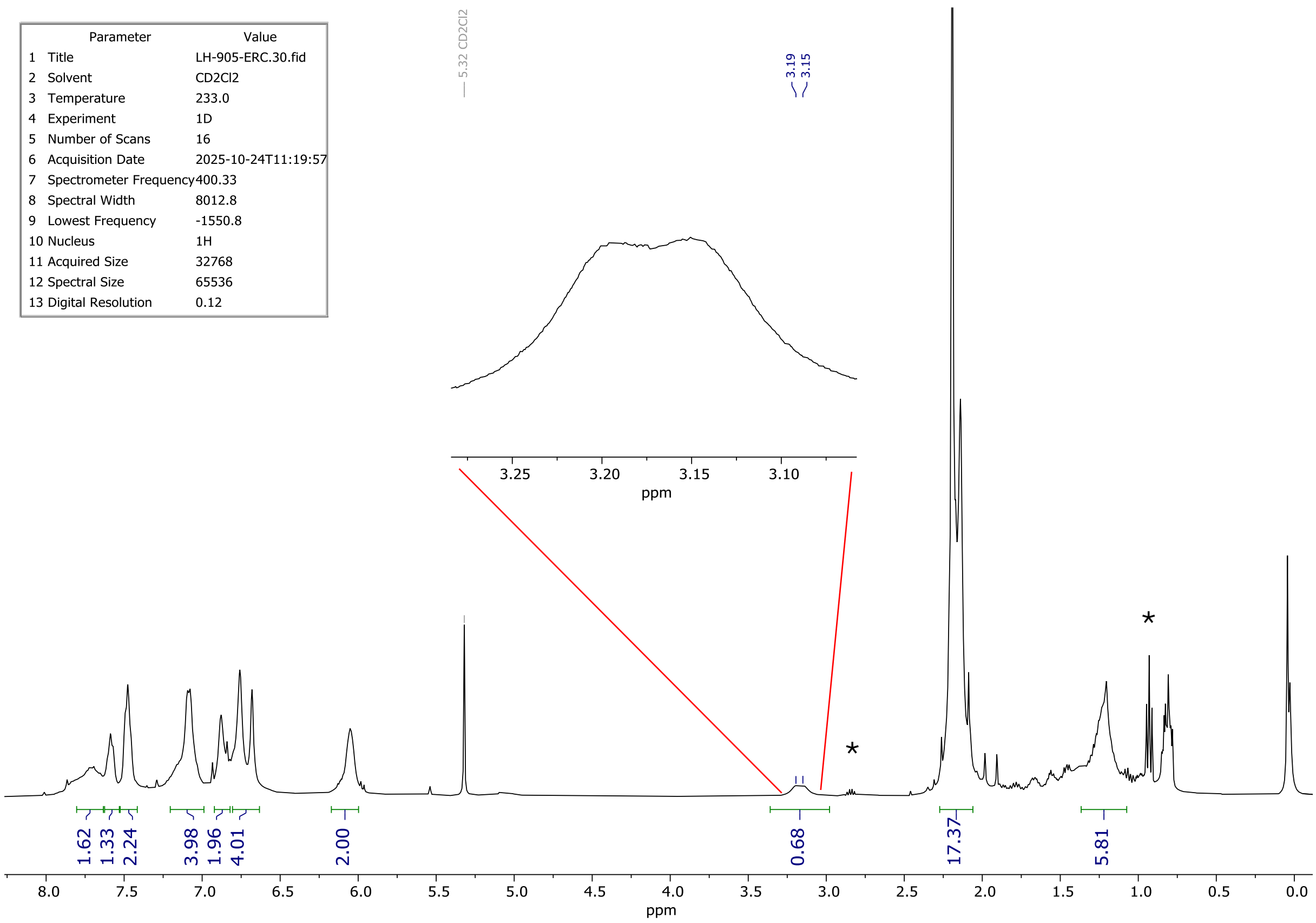

Figure S41: ^{1}H NMR spectrum of **3**$_{\mathbf{ArF}}$ in CD_2Cl_2 at –40 °C. The * highlights the signals of residual LDA.

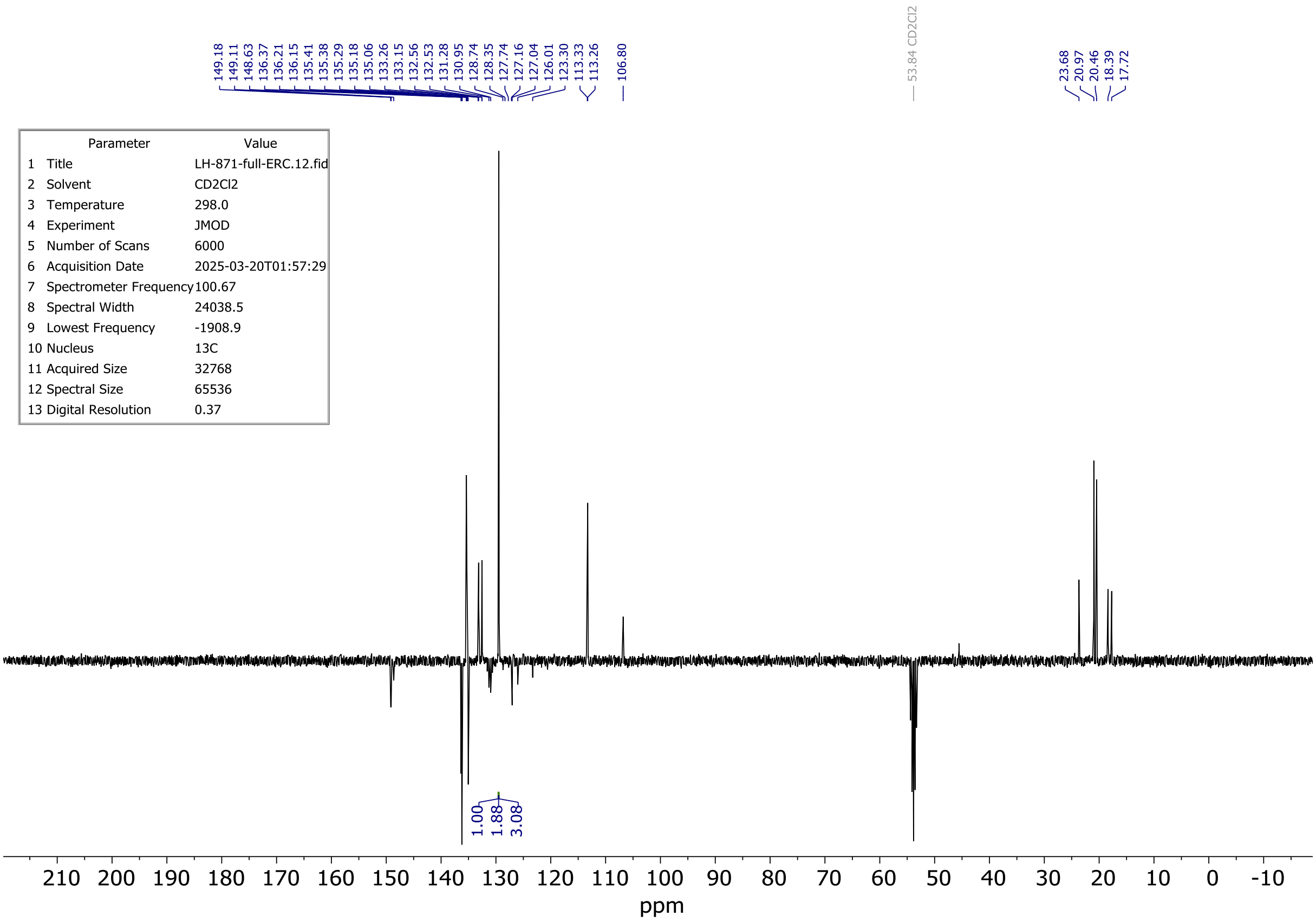

Figure S42: $^{13}C\{^{1}H\}$-APT NMR spectrum of $\mathbf{3_{ArF}}$ in CD_2Cl_2.

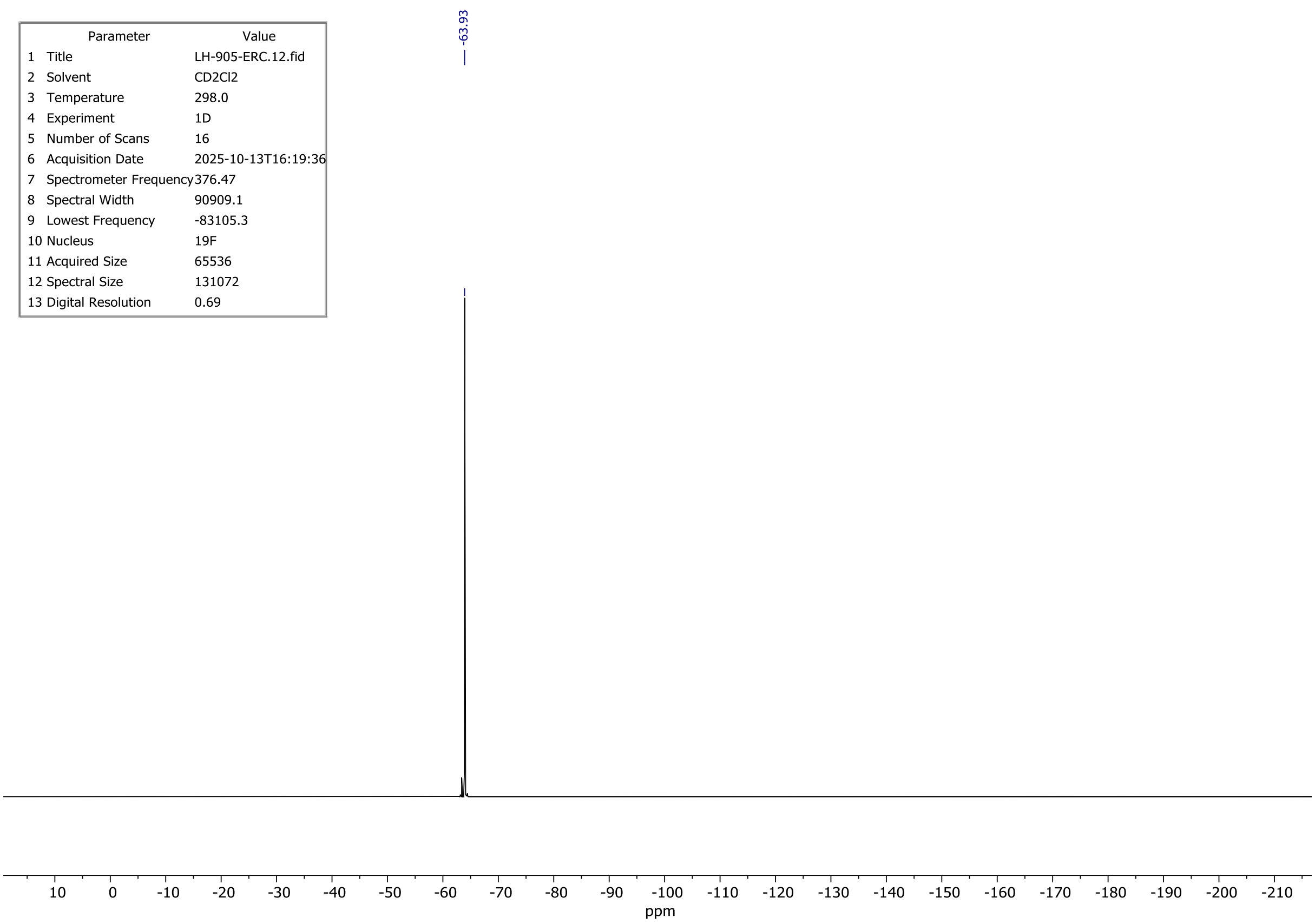

Figure S43: $^{19}F\{^{1}H\}$ NMR spectrum of $\mathbf{3_{ArF}}$ in CD_2Cl_2.

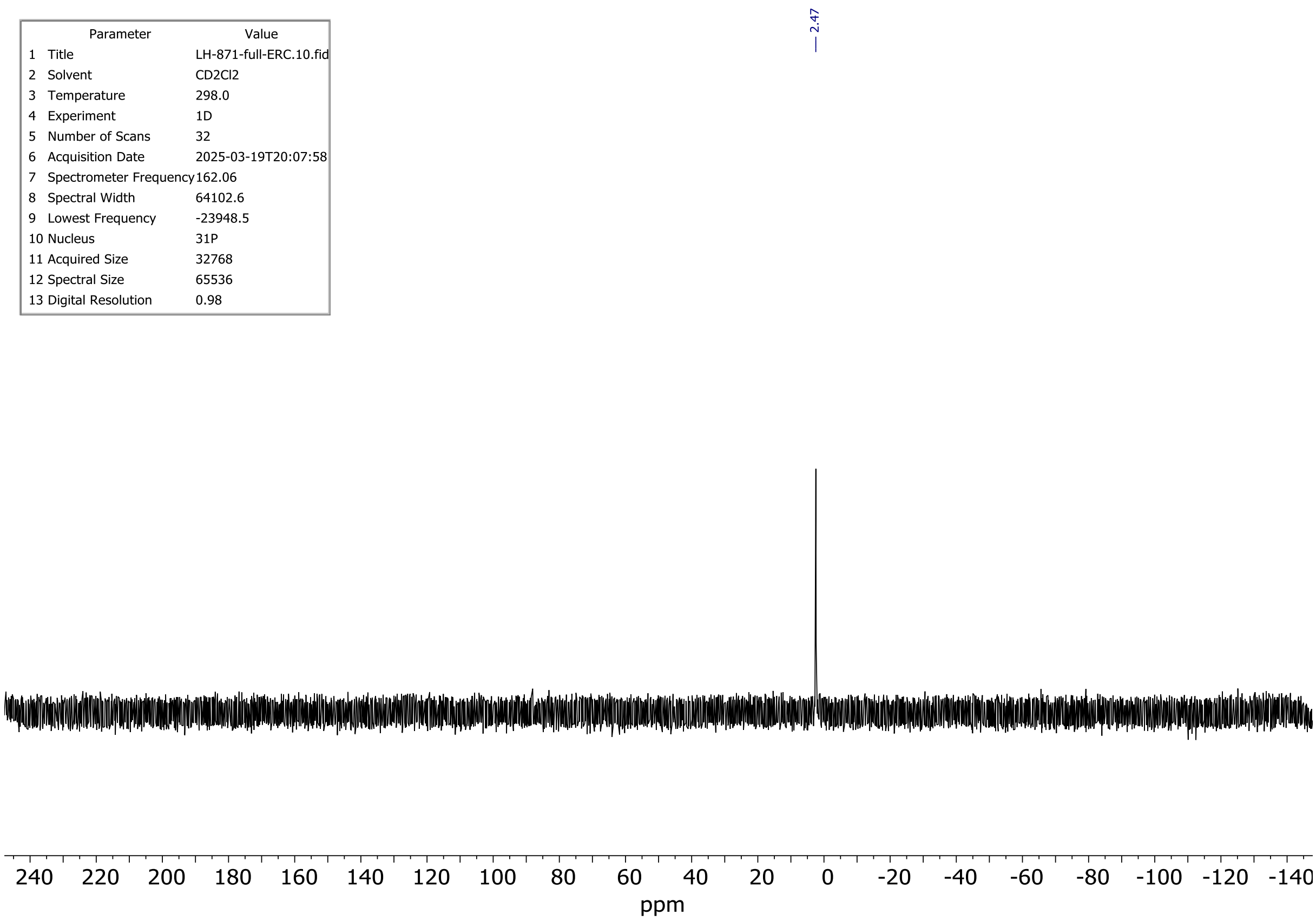

Figure S44: $^{31}P\{^1H\}$ NMR spectrum of $\mathbf{3_{ArF}}$ in CD_2Cl_2.

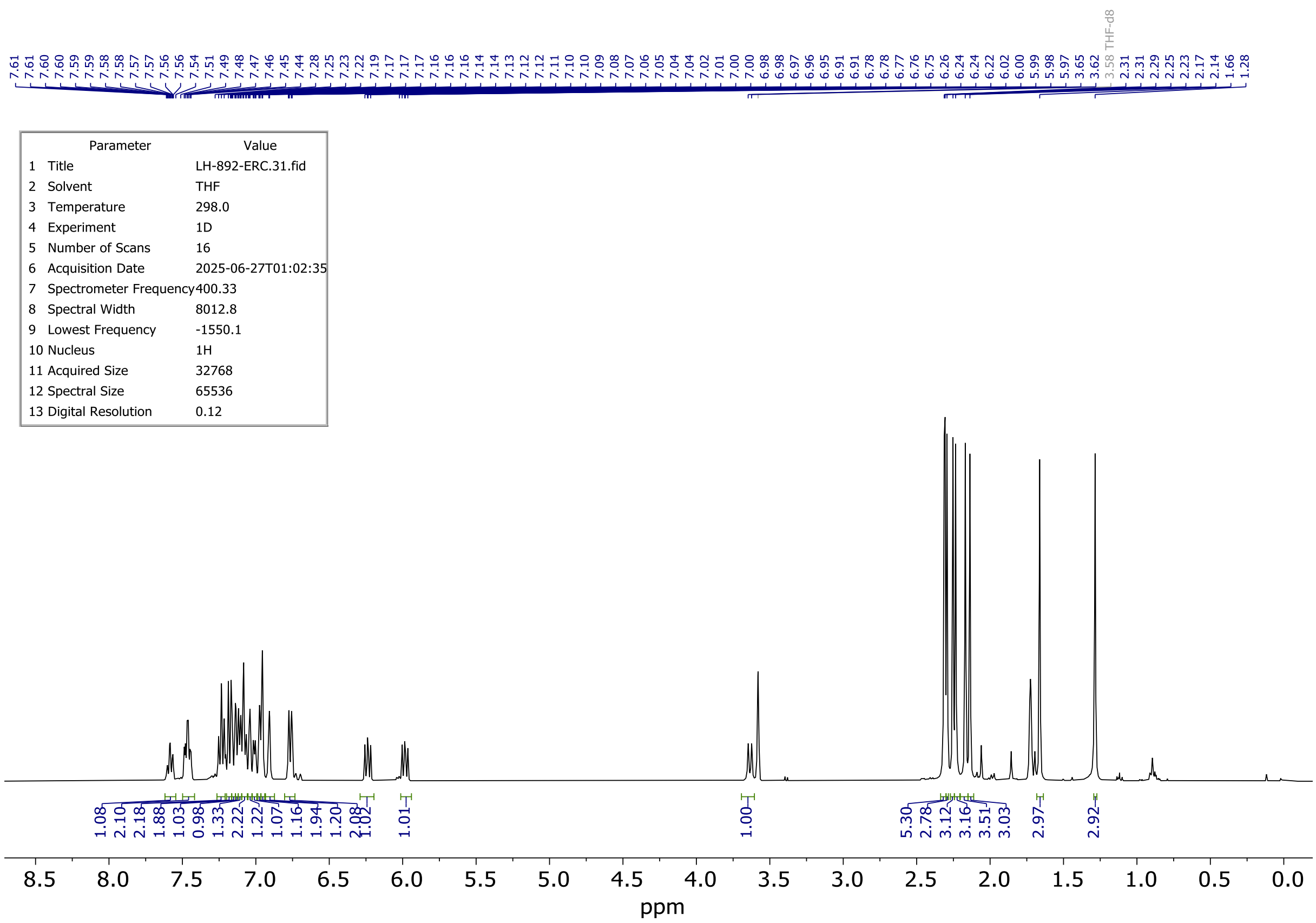

Figure S45: ^1H NMR spectrum of $\mathbf{4_{Ph}Cl}$ in THF-d_8.

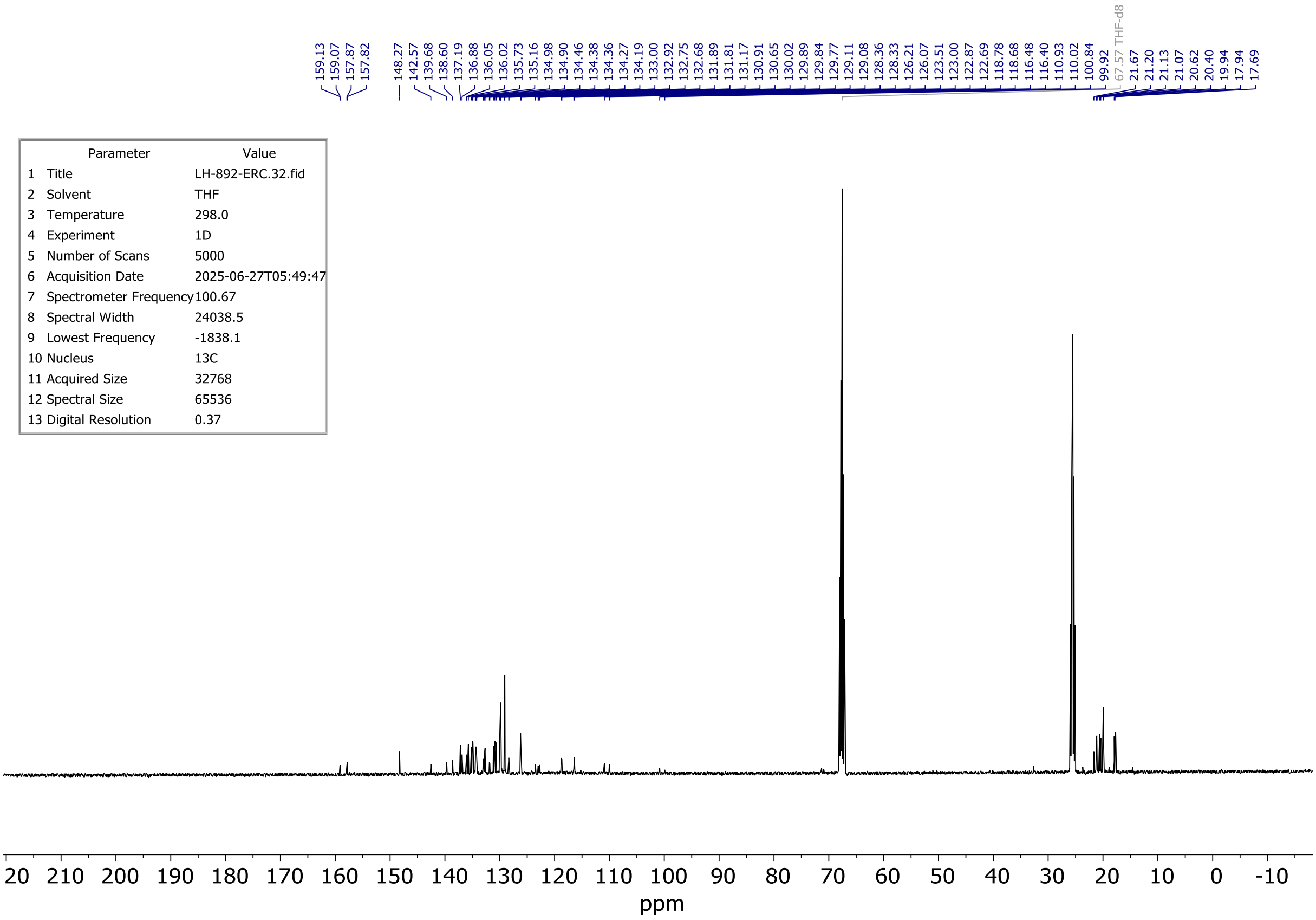

Figure S46: $^{13}C\{^1H\}$ NMR spectrum of $\mathbf{4_{Ph}Cl}$ in THF-d_8.

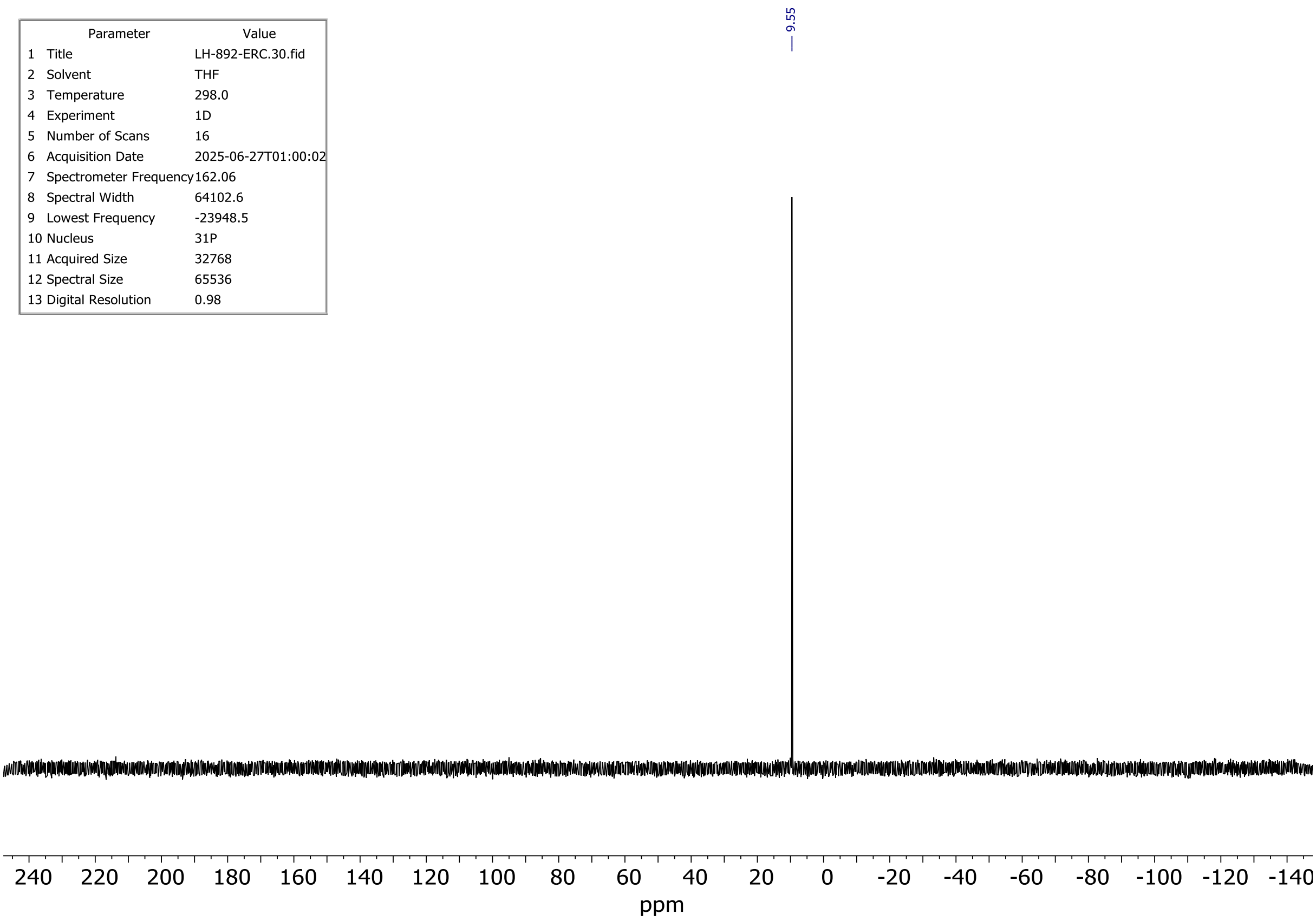

Figure S47: $^{31}P\{^1H\}$ NMR spectrum of $\mathbf{4_{Ph}Cl}$ in THF-d_8.

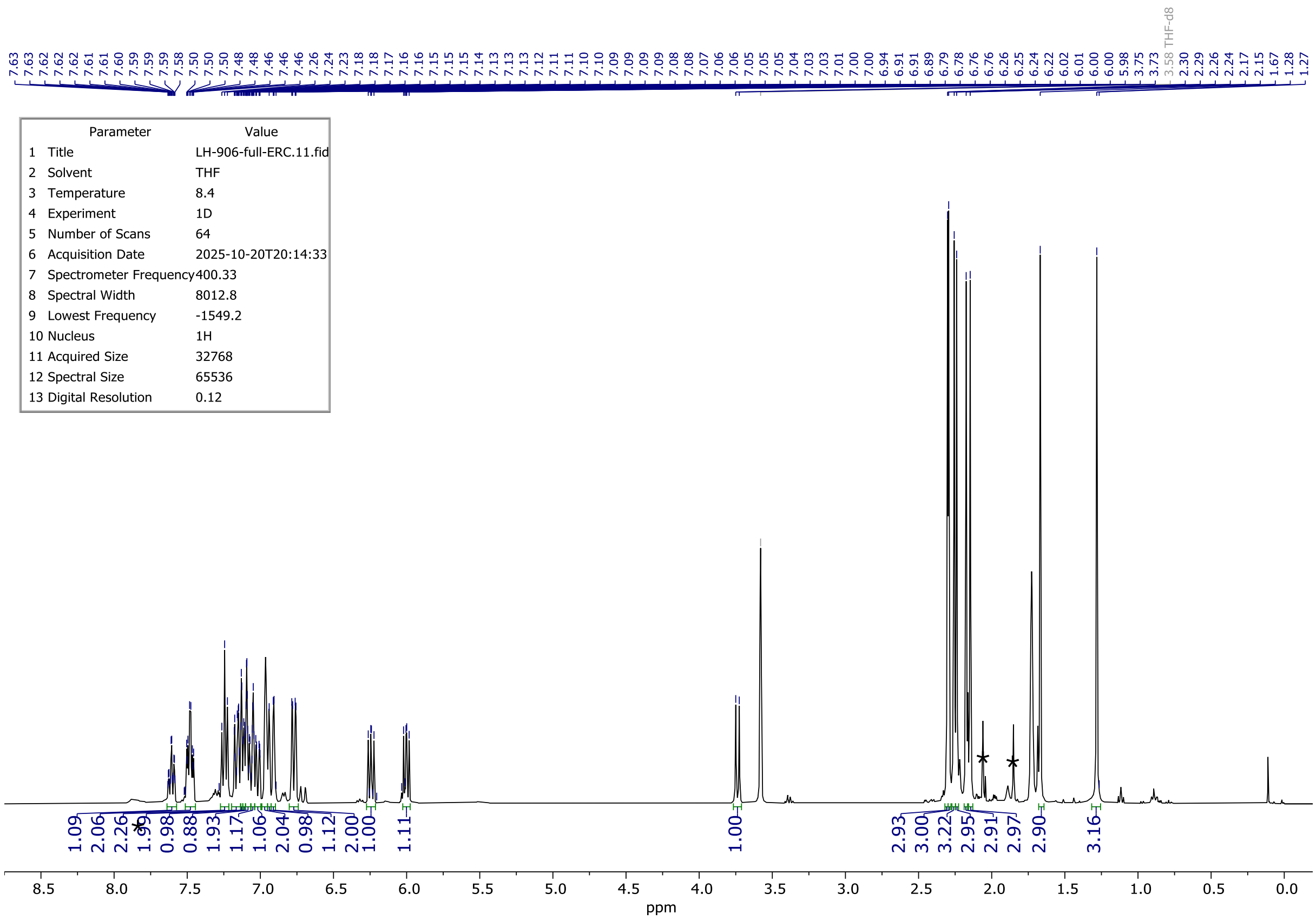

Figure S48: ^{1}H NMR spectrum of **4$_{Ph}$Br** in THF-d$_8$. The * highlights the signals of residual **2$_{Ph}$I**.

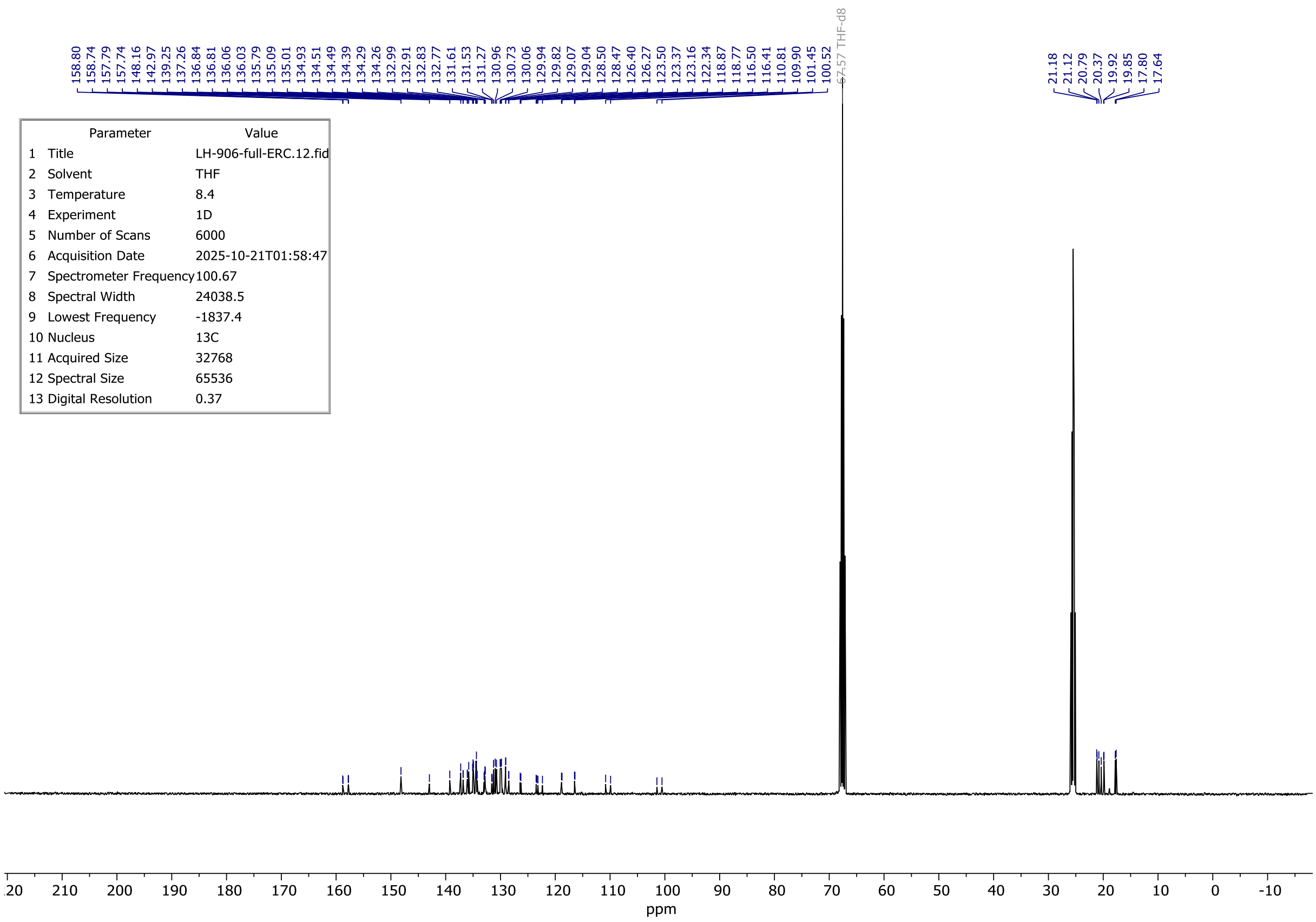

Figure S49: ^{13}C{^{1}H} NMR spectrum of **4$_{Ph}$Br** in THF-d$_8$.

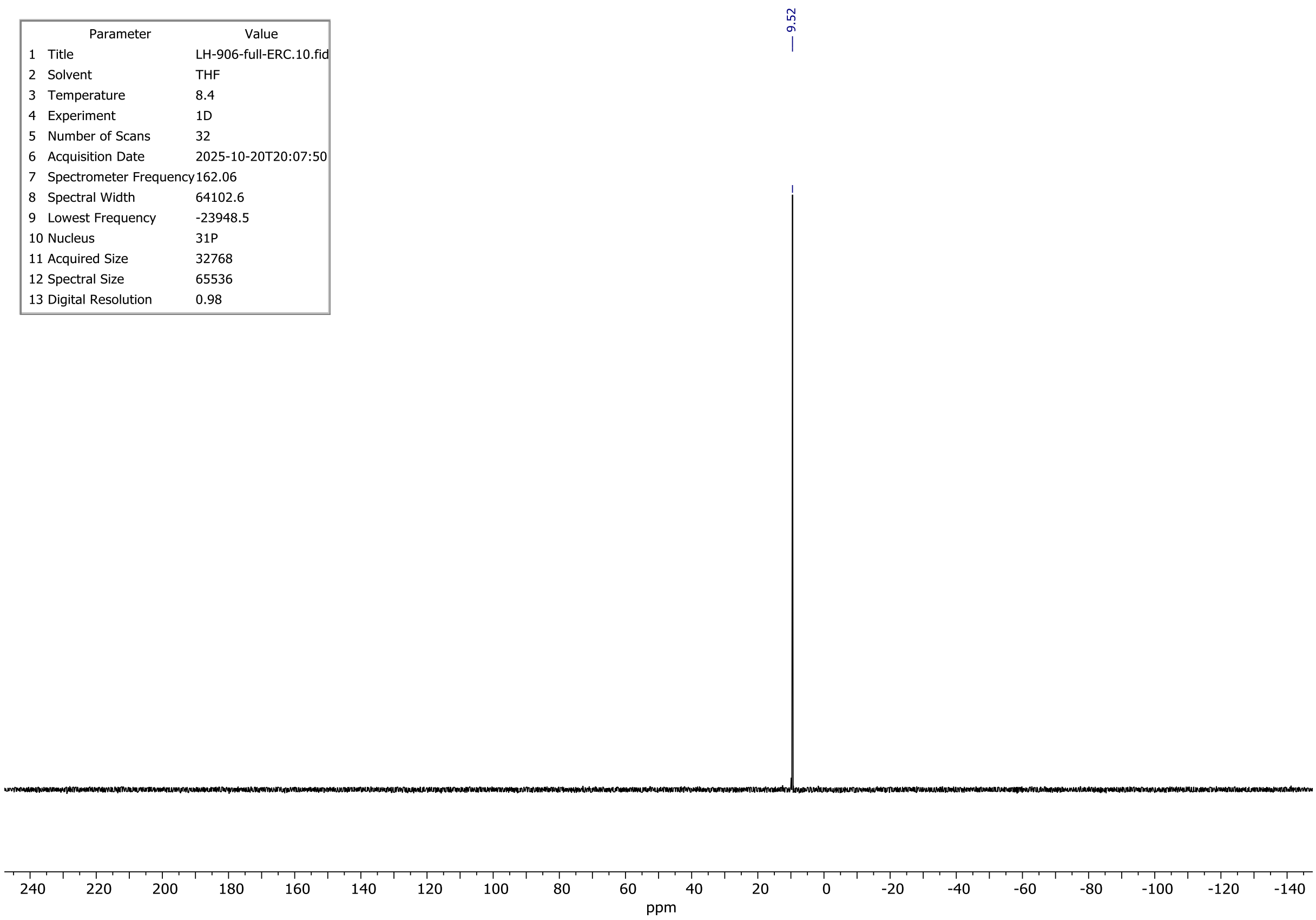

Figure S50: $^{31}P\{^{1}H\}$ NMR spectrum of $\mathbf{4_{Ph}Br}$ in THF-d8.

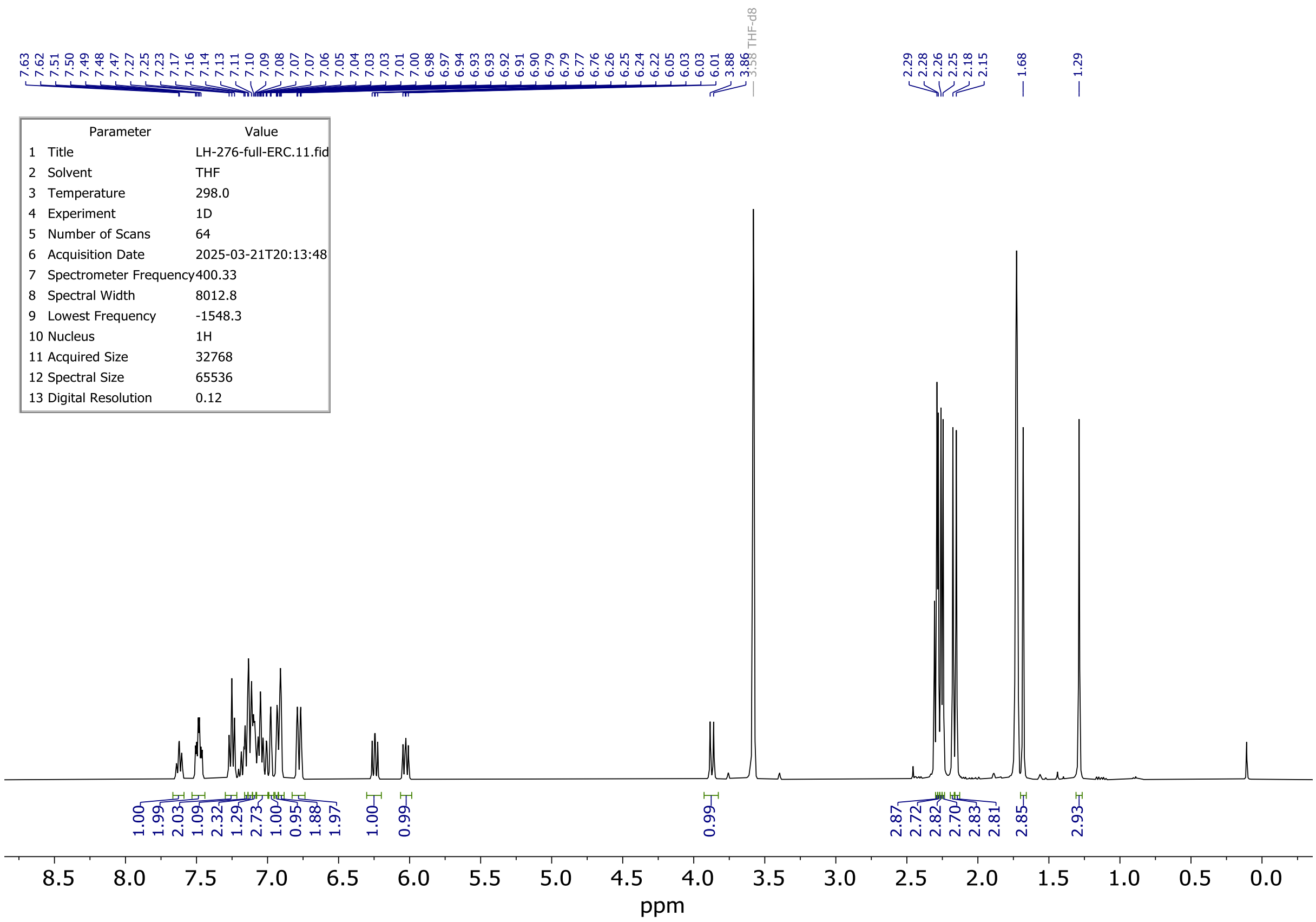

Figure S51: ^{1}H NMR spectrum of $\mathbf{4_{Ph}I}$ in THF-d8.

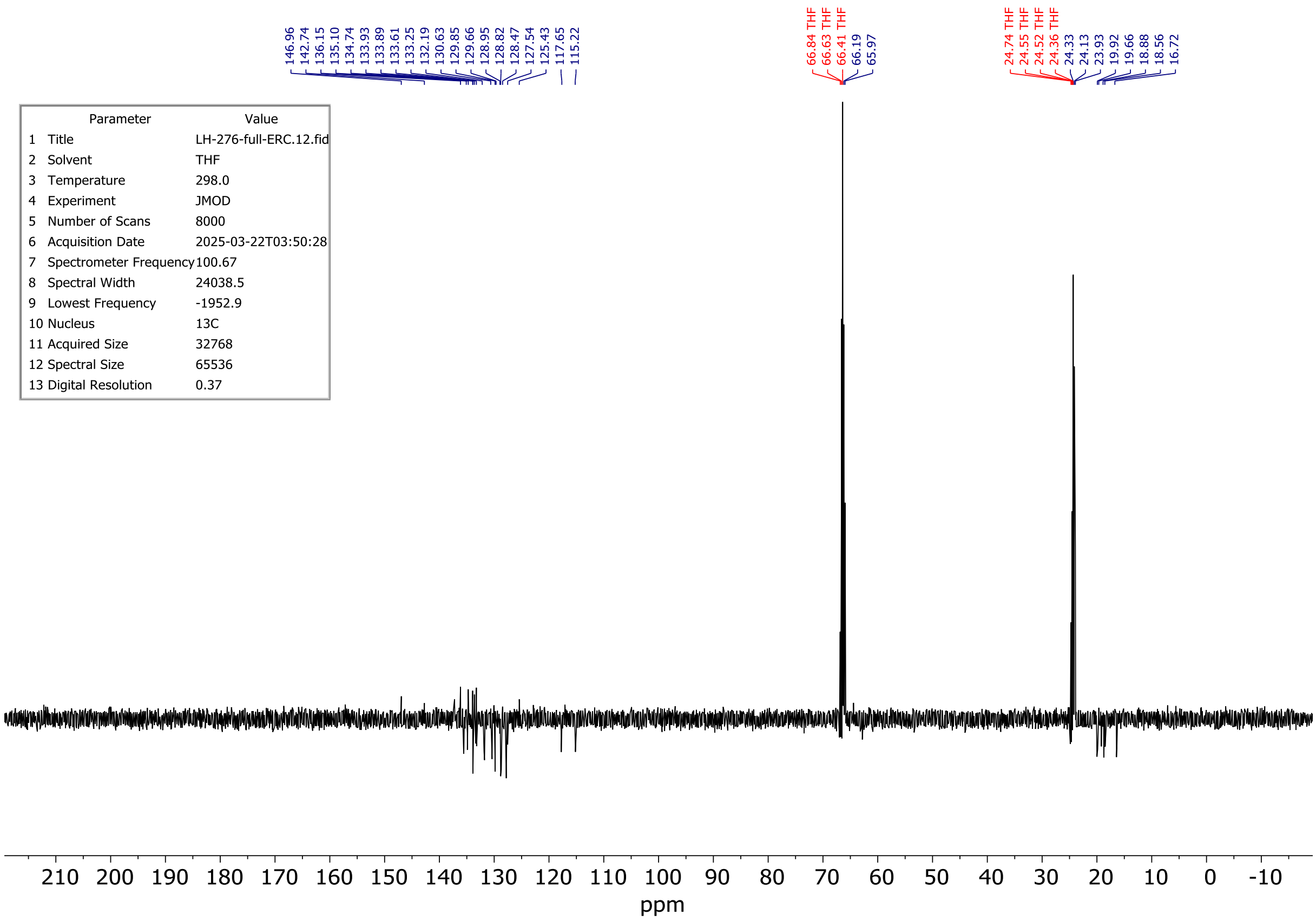

Figure S52: $^{13}C\{^{1}H\}$ NMR spectrum of $\mathbf{4_{Ph}I}$ in THF-d_8.

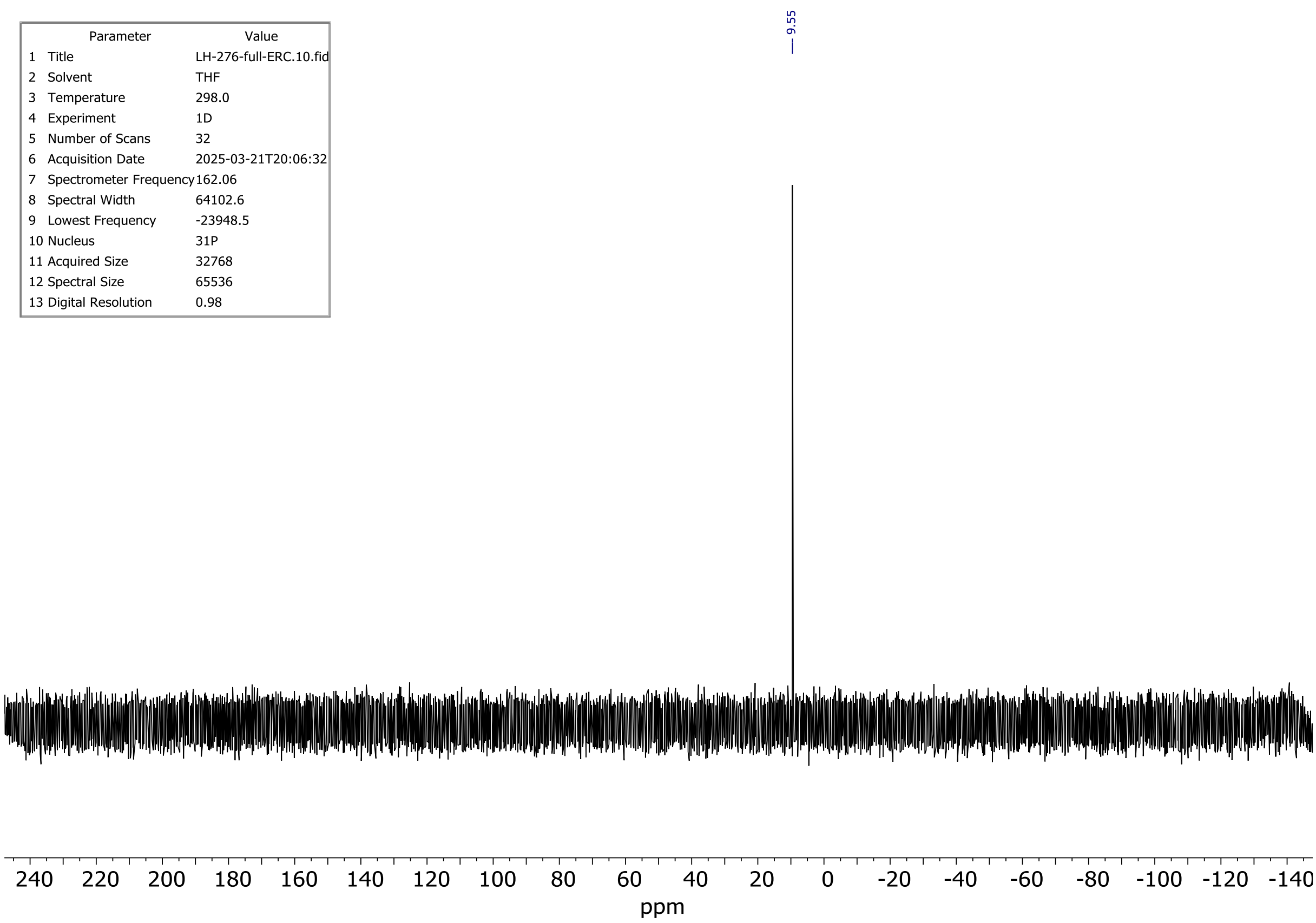

Figure S53: $^{31}P\{^{1}H\}$ NMR spectrum of $\mathbf{4_{Ph}I}$ in THF-d_8.

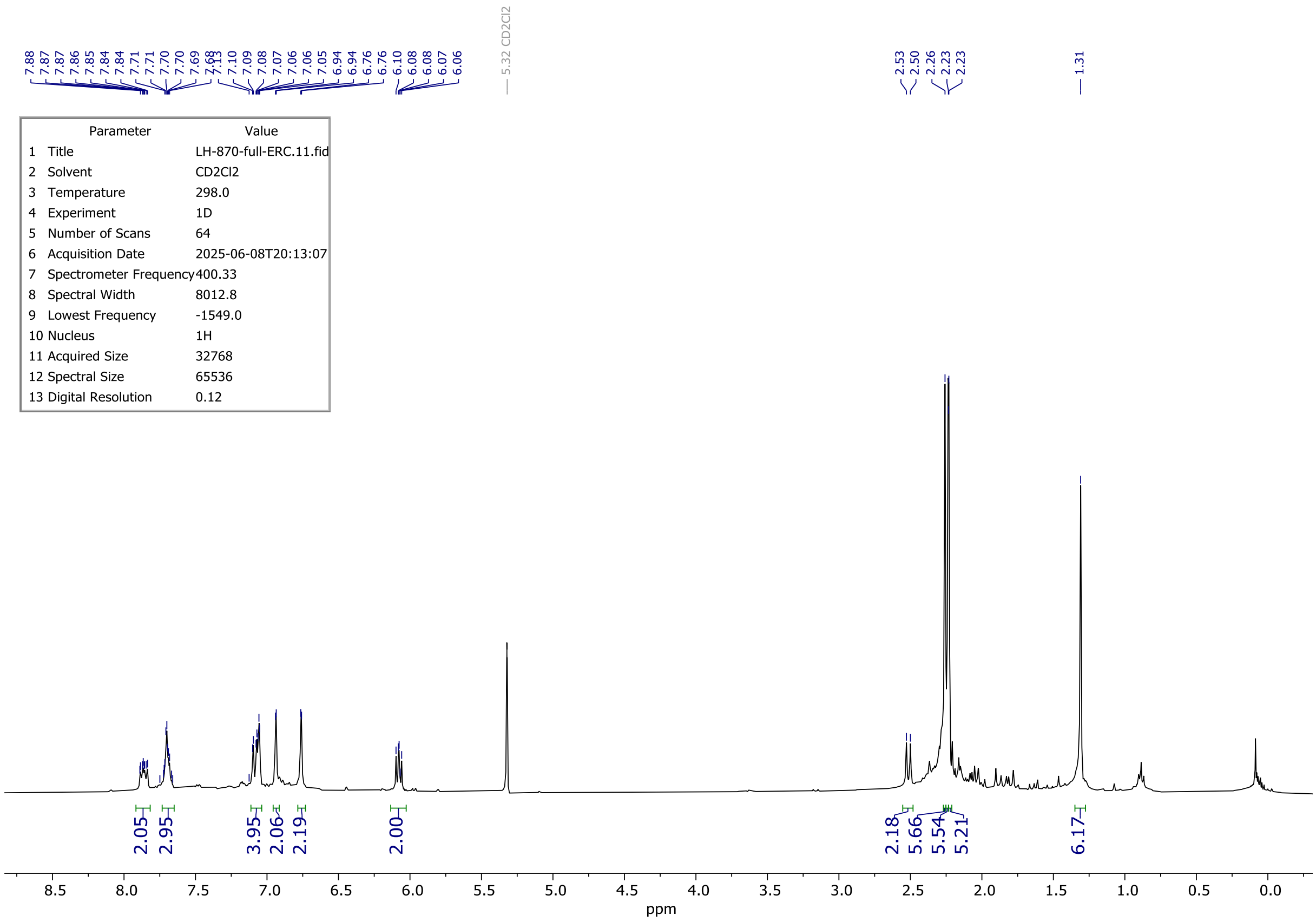

Figure S54: ^{1}H NMR spectrum of **$4_{H}Cl$** in THF-d_8.

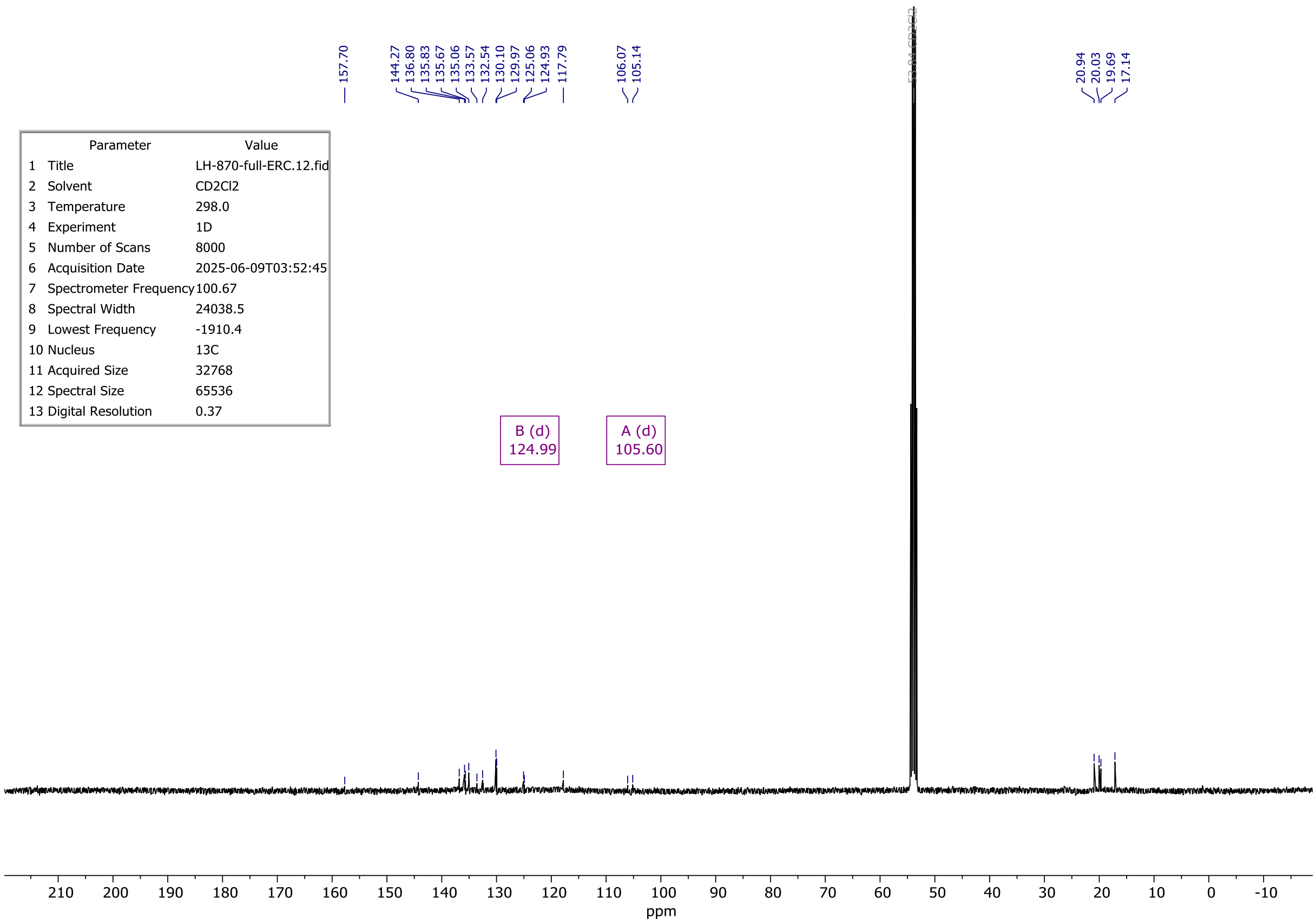

Figure S55: $^{13}C\{^{1}H\}$ NMR spectrum of **$4_{H}Cl$** in THF-d_8.

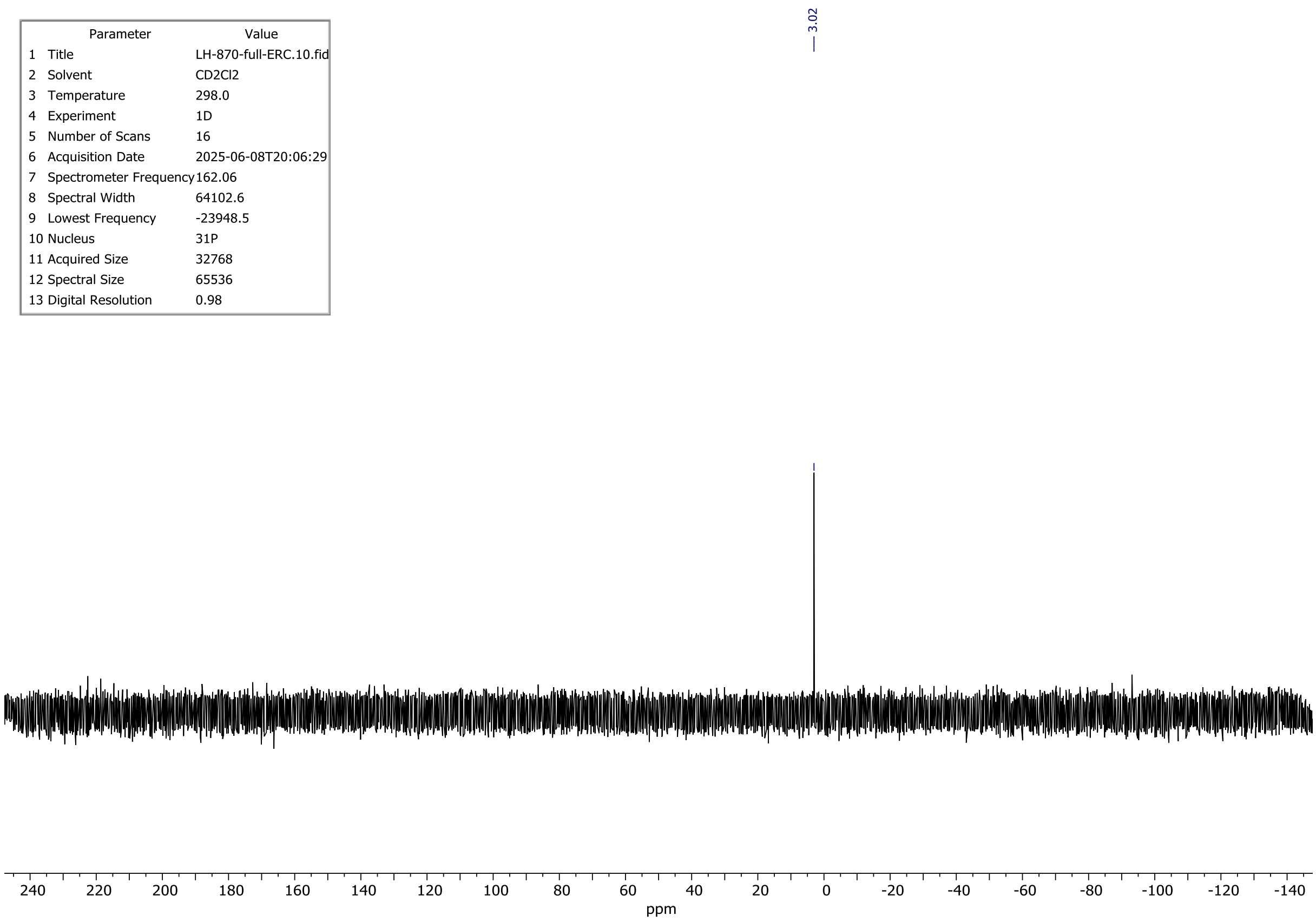

Figure S56: $^{31}P\{^{1}H\}$ NMR spectrum of **4_{H}Cl** in THF-d_8.

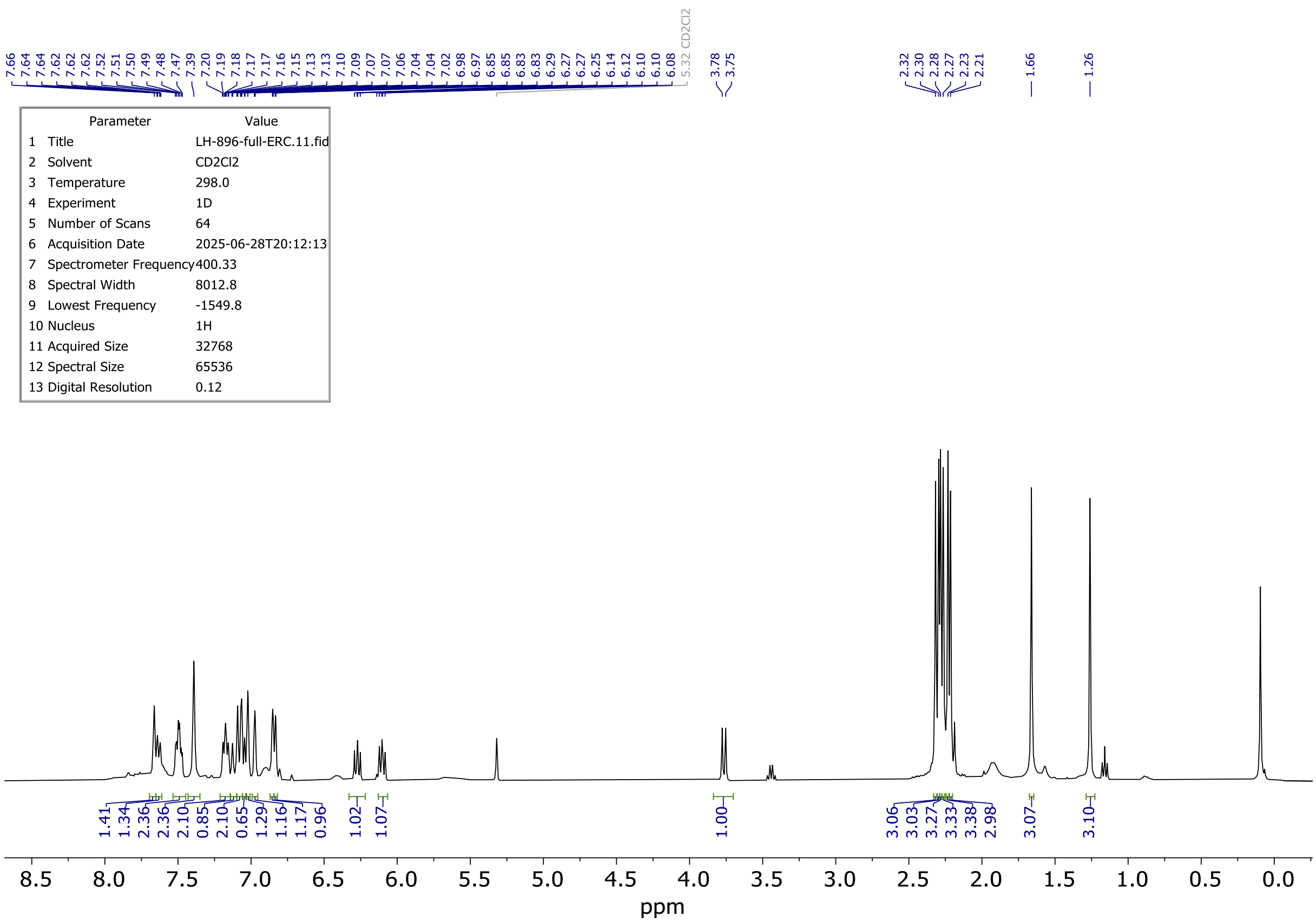

Figure S57: ^{1}H NMR spectrum of **4_{ArF}Cl** in CD_2Cl_2.

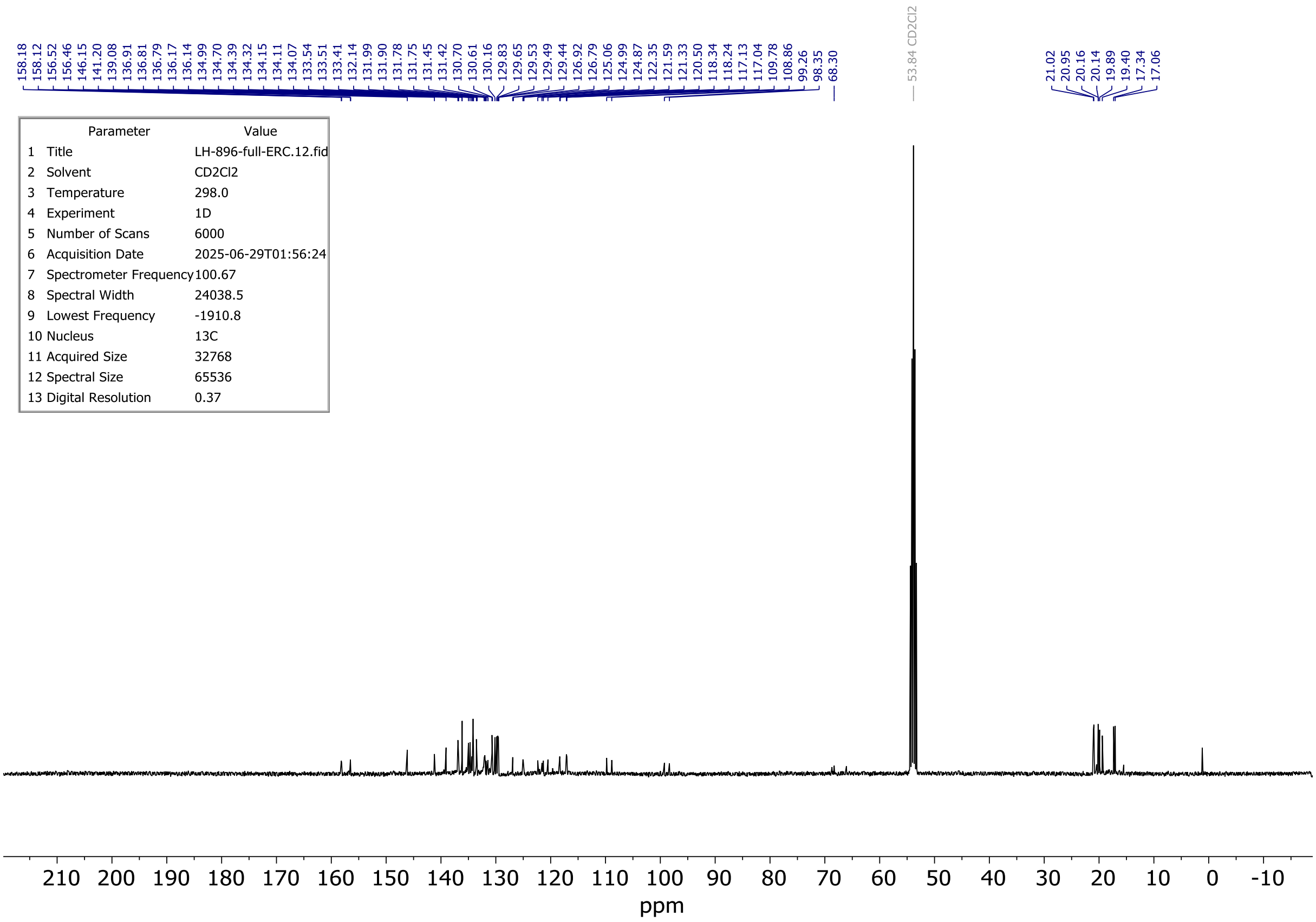

Figure S58: $^{13}C\{^{1}H\}$ NMR spectrum of $\mathbf{4_{ArF}Cl}$ in CD_2Cl_2.

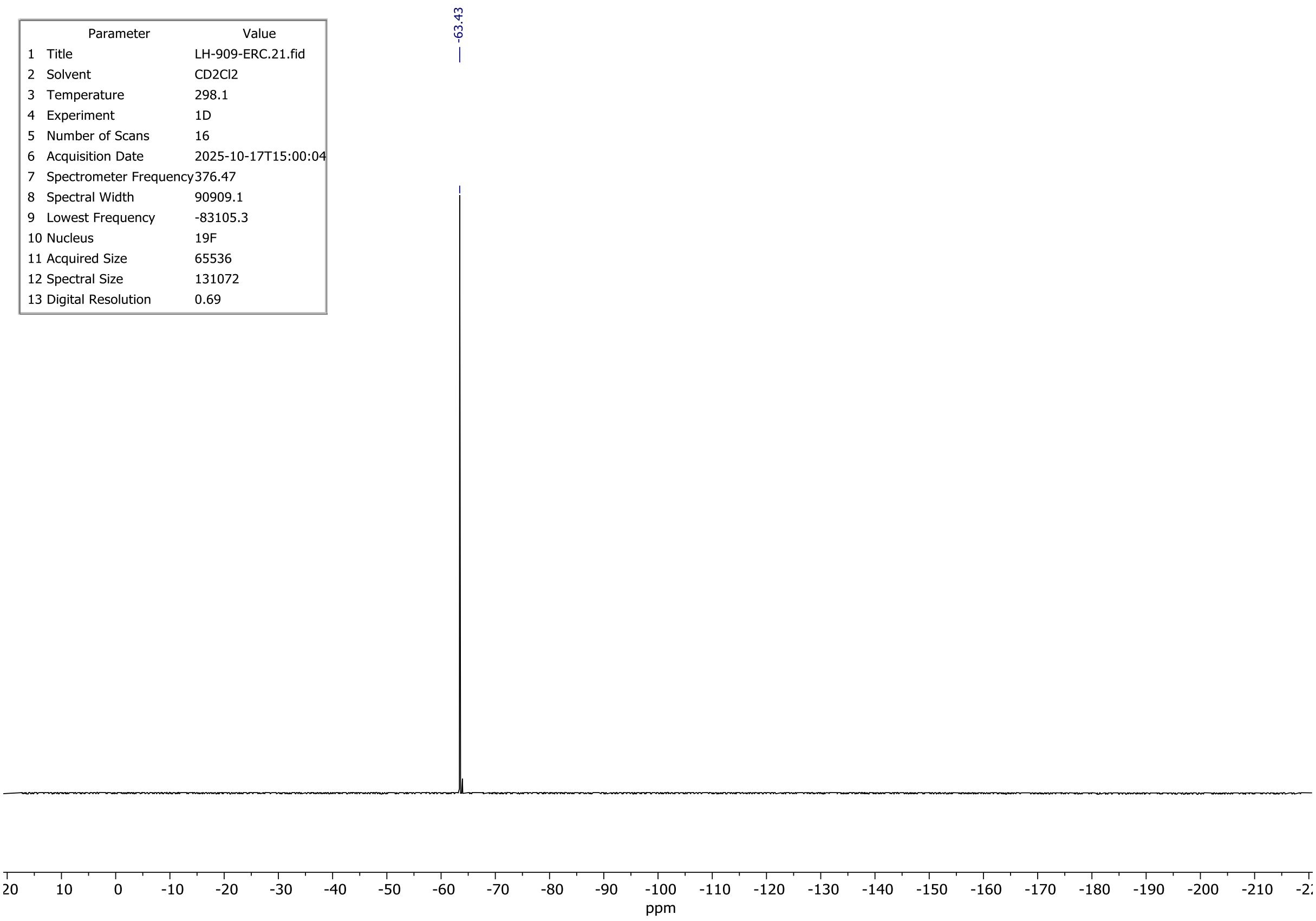

Figure S59: $^{19}F\{^{1}H\}$ NMR spectrum of $\mathbf{4_{ArF}Cl}$ in CD_2Cl_2.

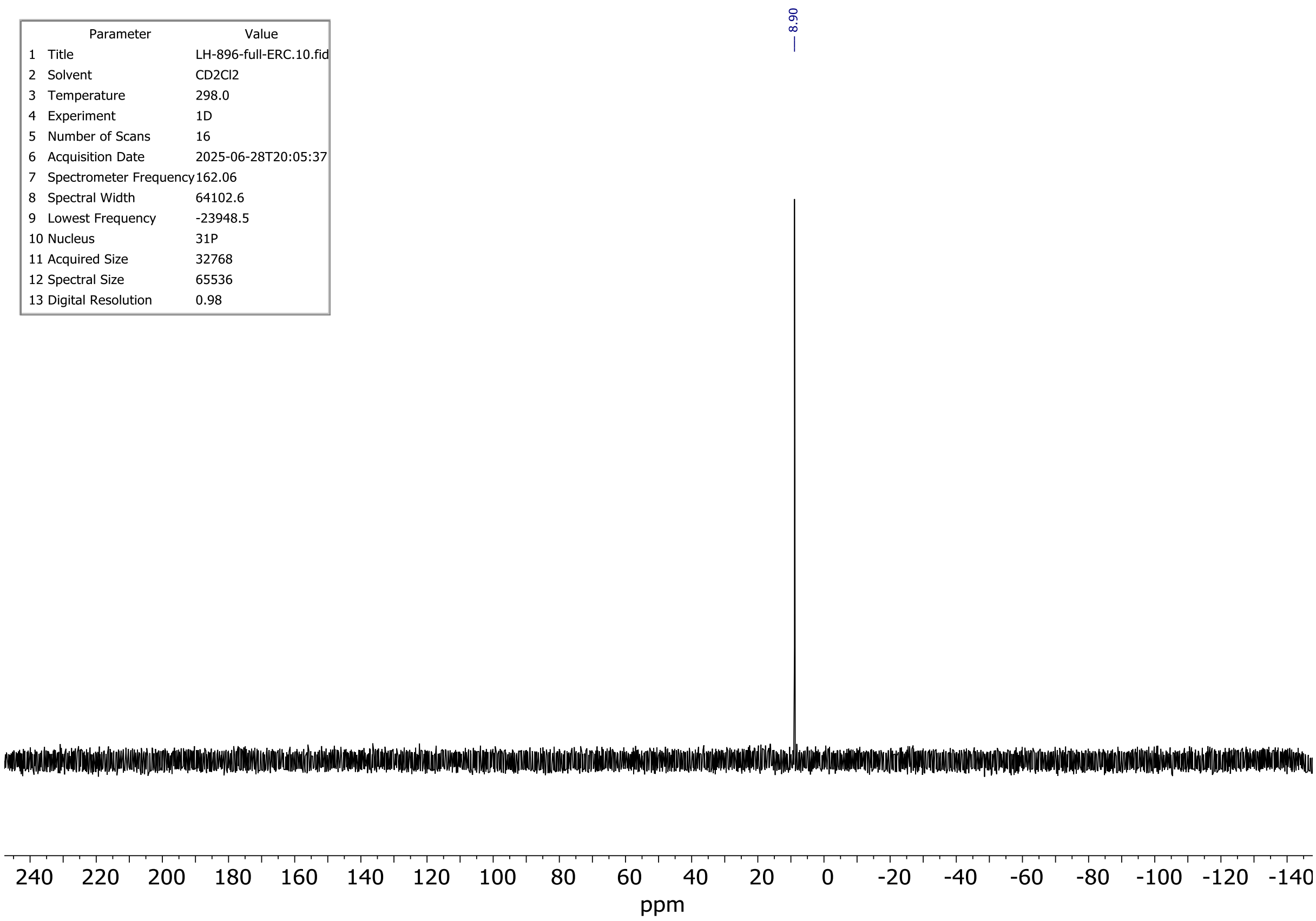

Figure S60: $^{31}P\{^1H\}$ NMR spectrum of $\mathbf{4_{ArF}Cl}$ in CD_2Cl_2.

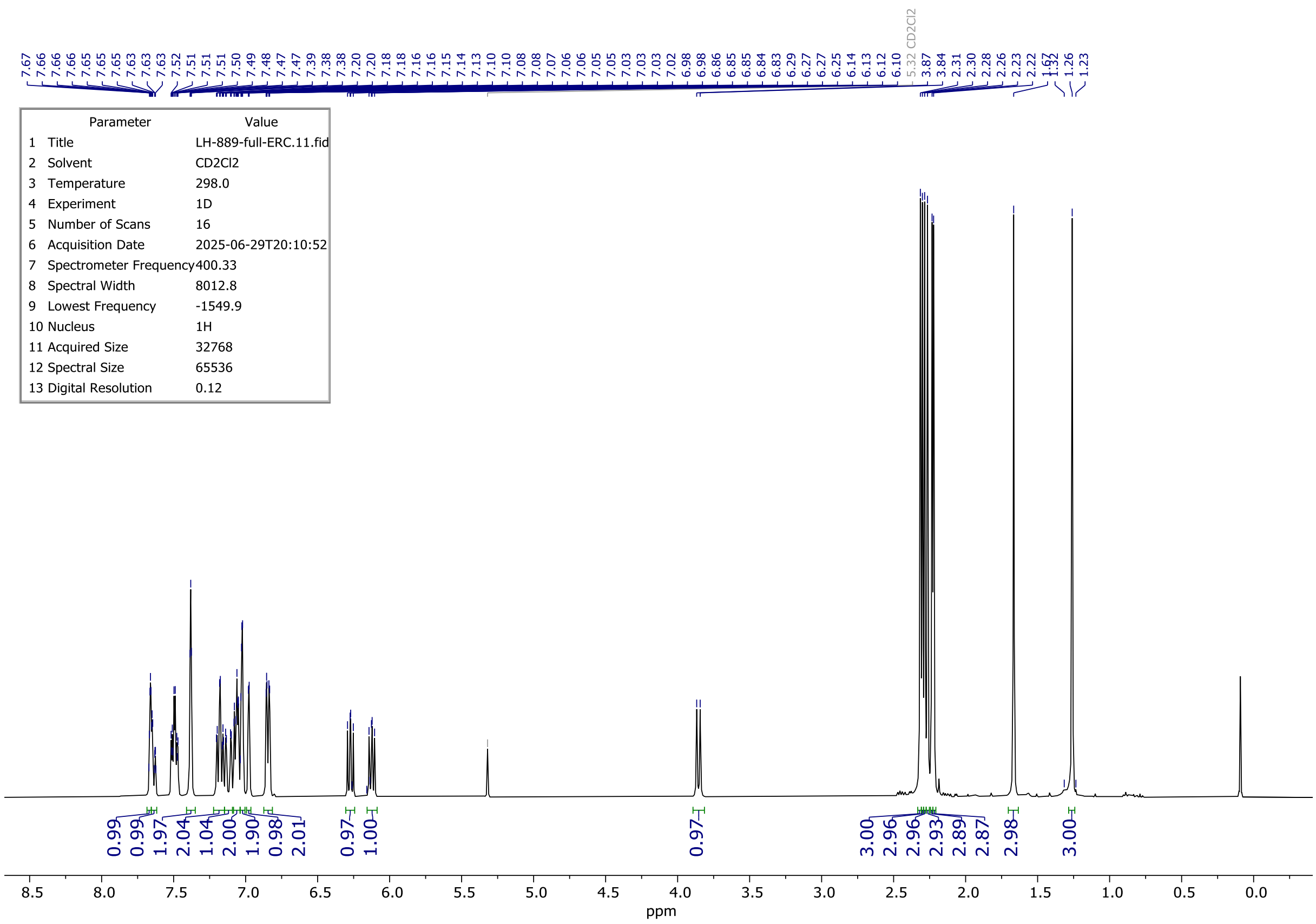

Figure S61: ^1H NMR spectrum of $\mathbf{4_{ArF}Br}$ in CD_2Cl_2.

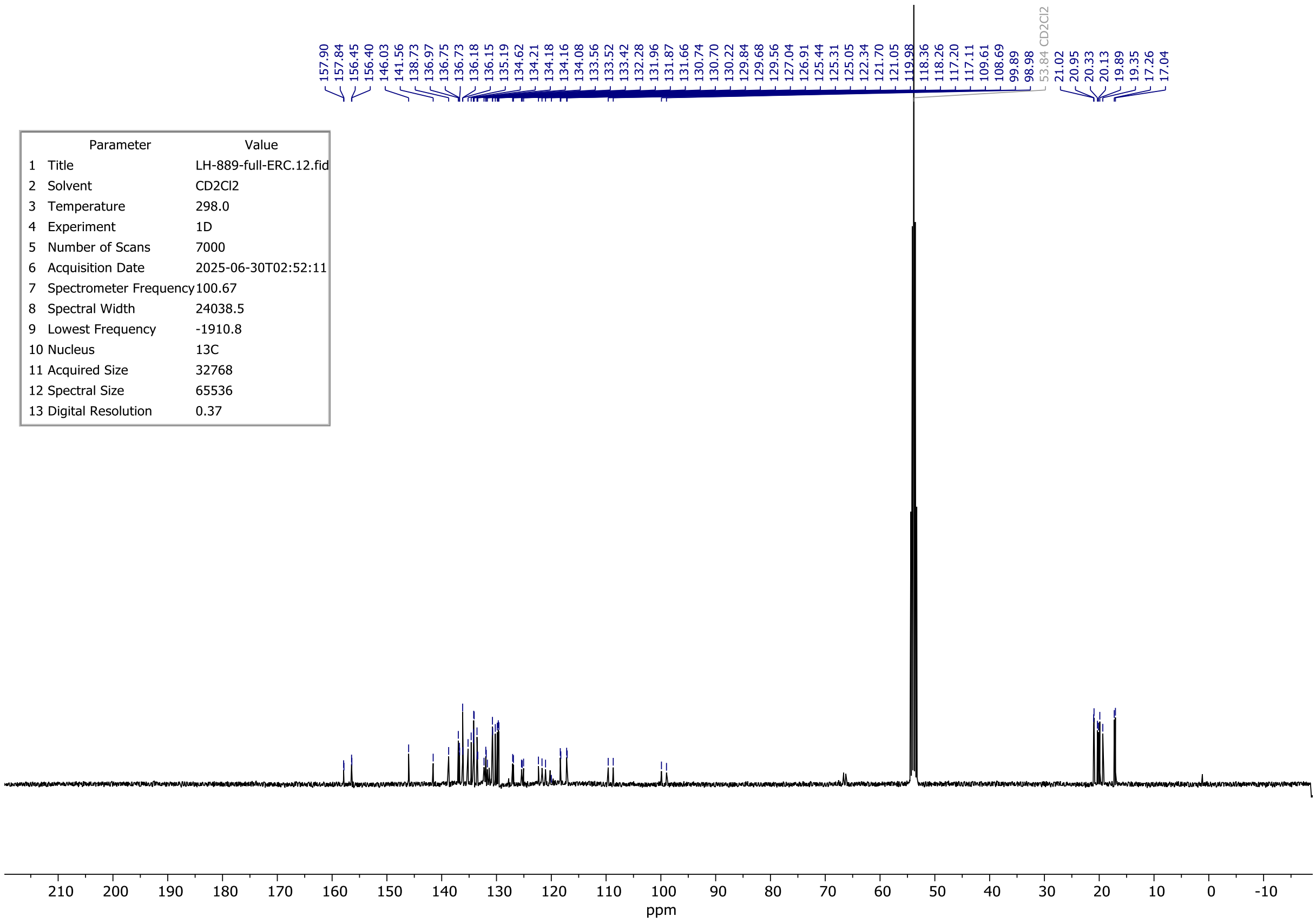

Figure S62: $^{13}C\{^1H\}$ NMR spectrum of $\mathbf{4_{ArF}Br}$ in CD_2Cl_2.

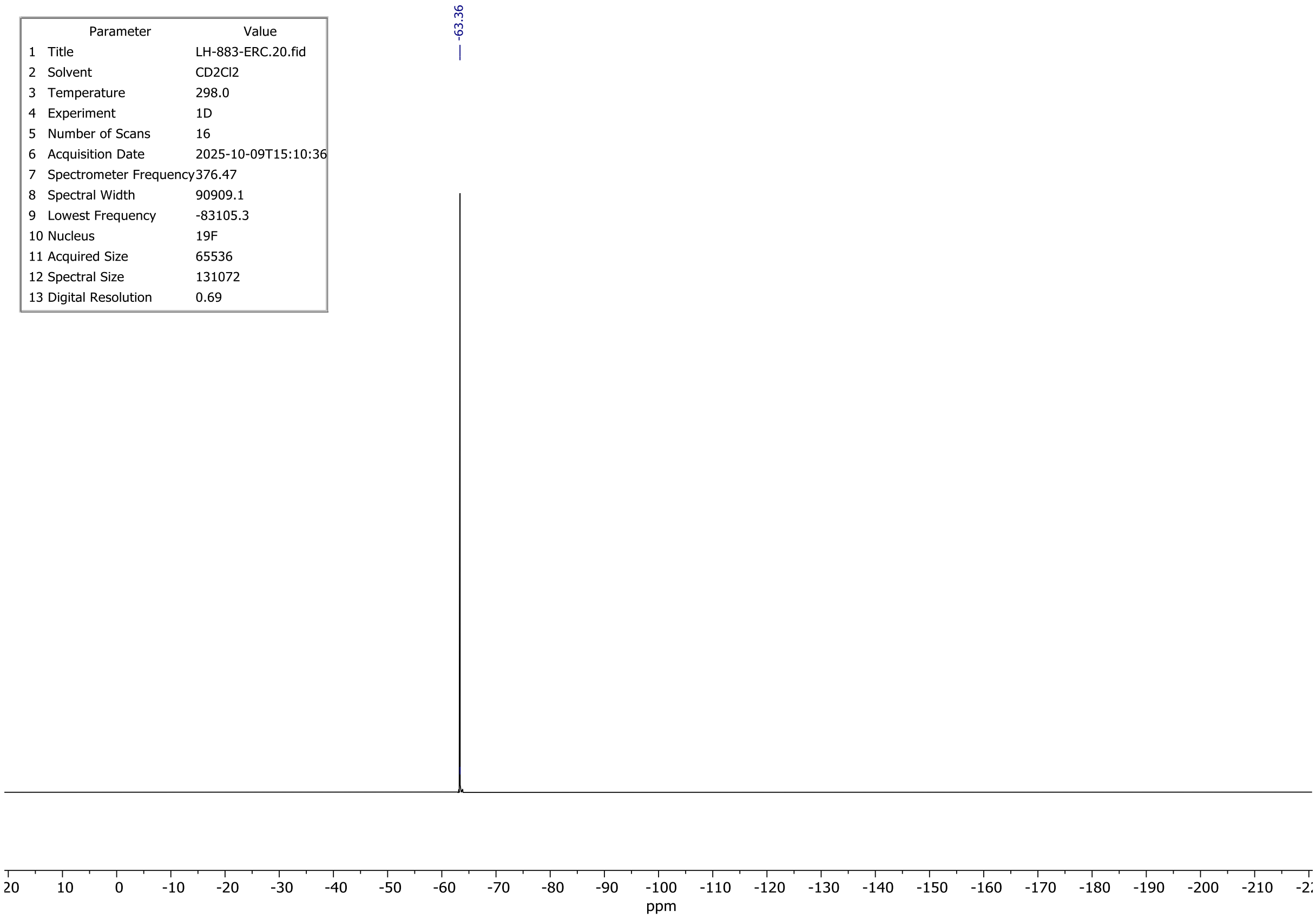

Figure S63: $^{19}F\{^1H\}$ NMR spectrum of $\mathbf{4_{ArF}Br}$ in CD_2Cl_2.

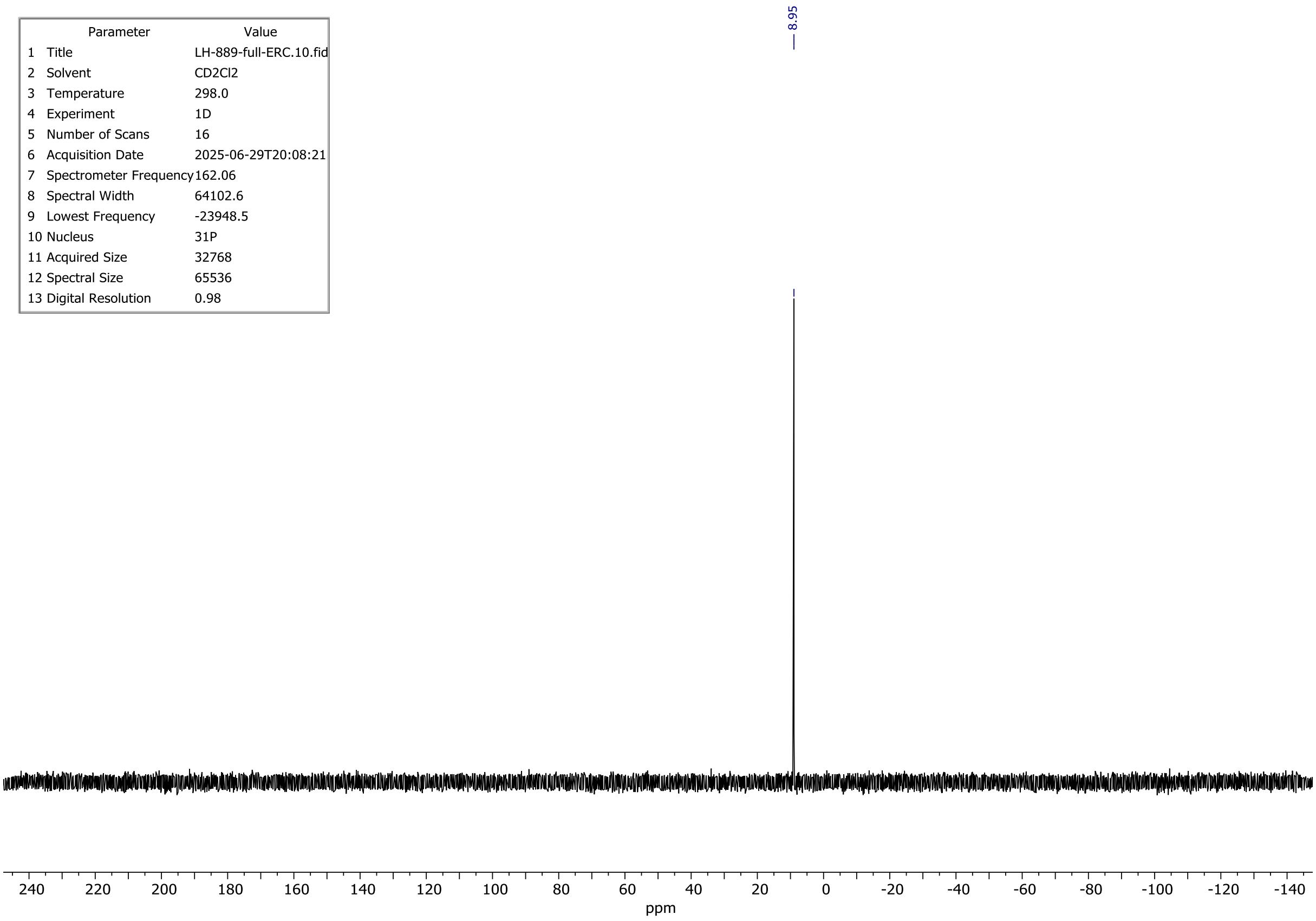

Figure S64: $^{31}P\{^{1}H\}$ NMR spectrum of $\mathbf{4_{ArF}Br}$ in CD_2Cl_2.

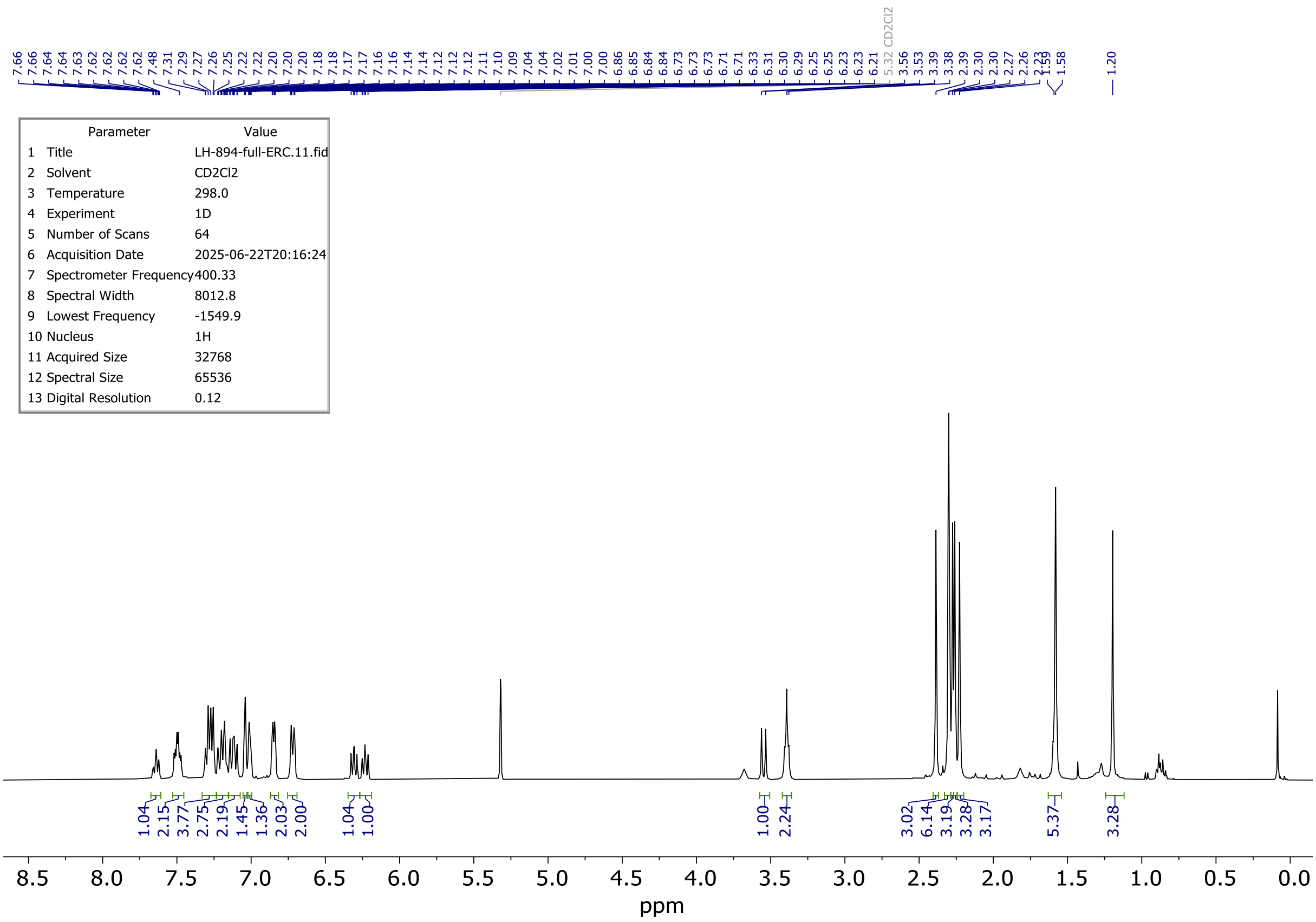

Figure S65: ^{1}H NMR spectrum of $\mathbf{5_{Ph}OTf}$ in CD_2Cl_2.

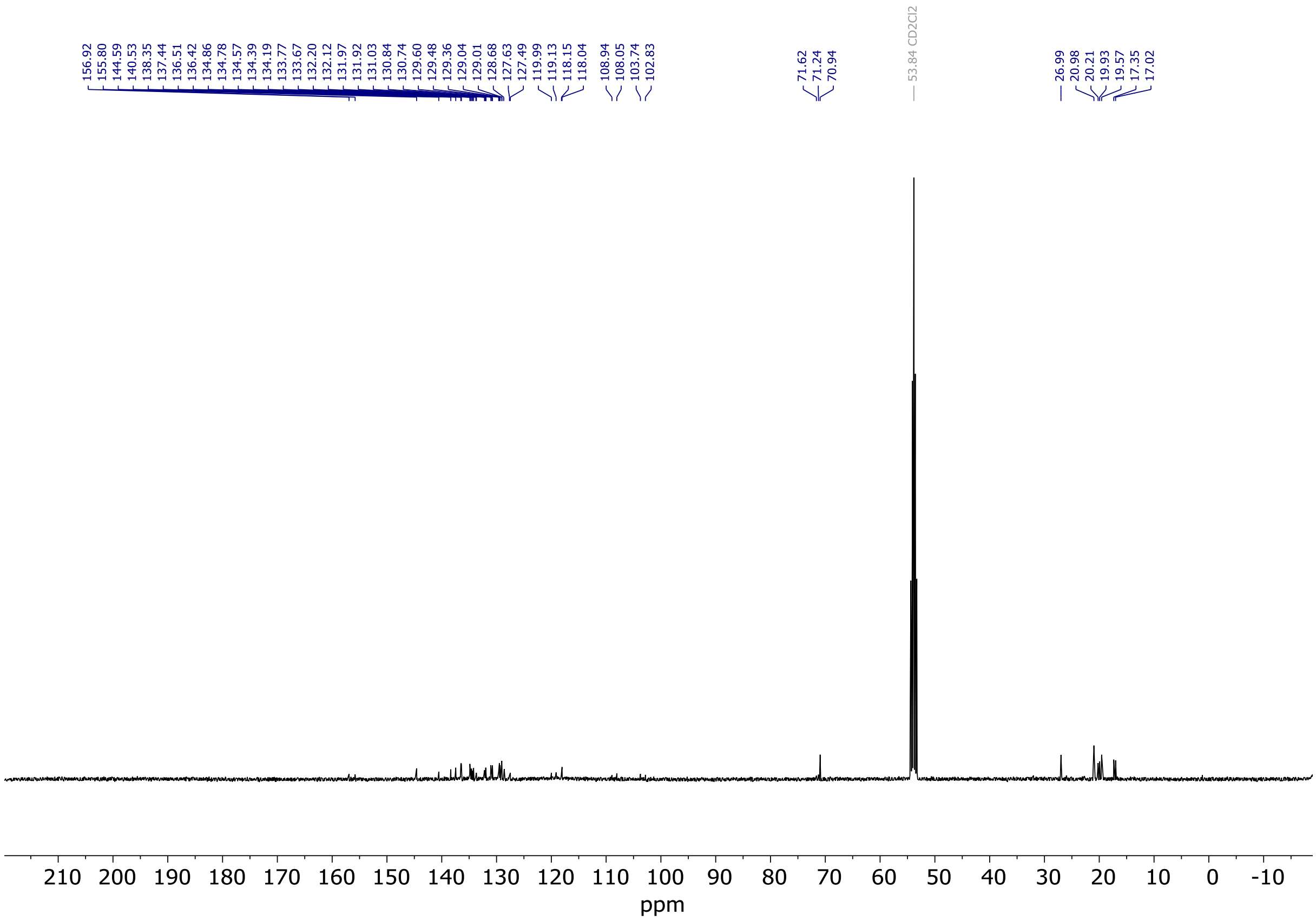

Figure S66: $^{13}C\{^1H\}$ NMR spectrum of $\mathbf{5_{Ph}OTf}$ in CD_2Cl_2.

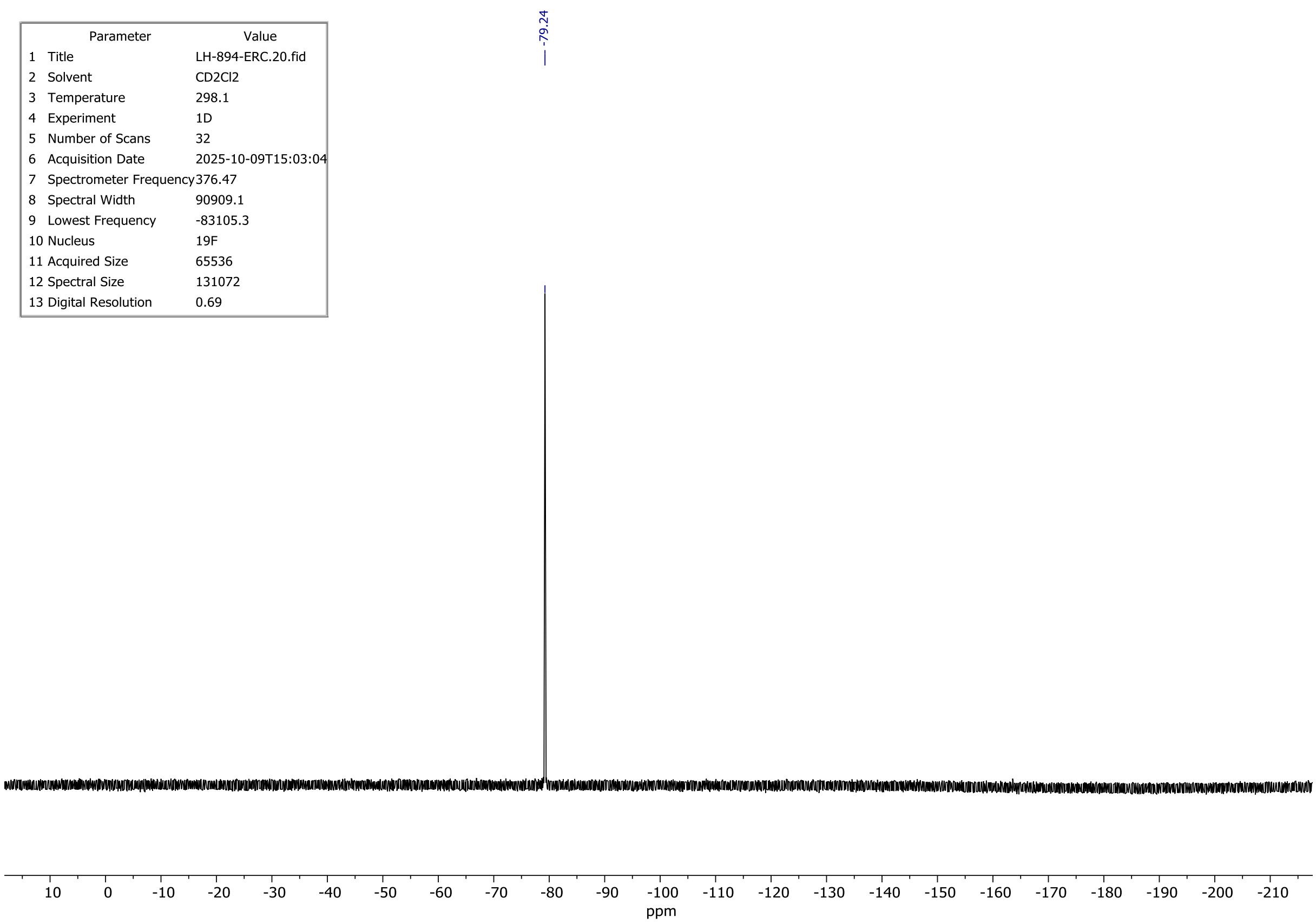

Figure S67: $^{19}F\{^1H\}$ NMR spectrum of $\mathbf{5_{Ph}OTf}$ in CD_2Cl_2.

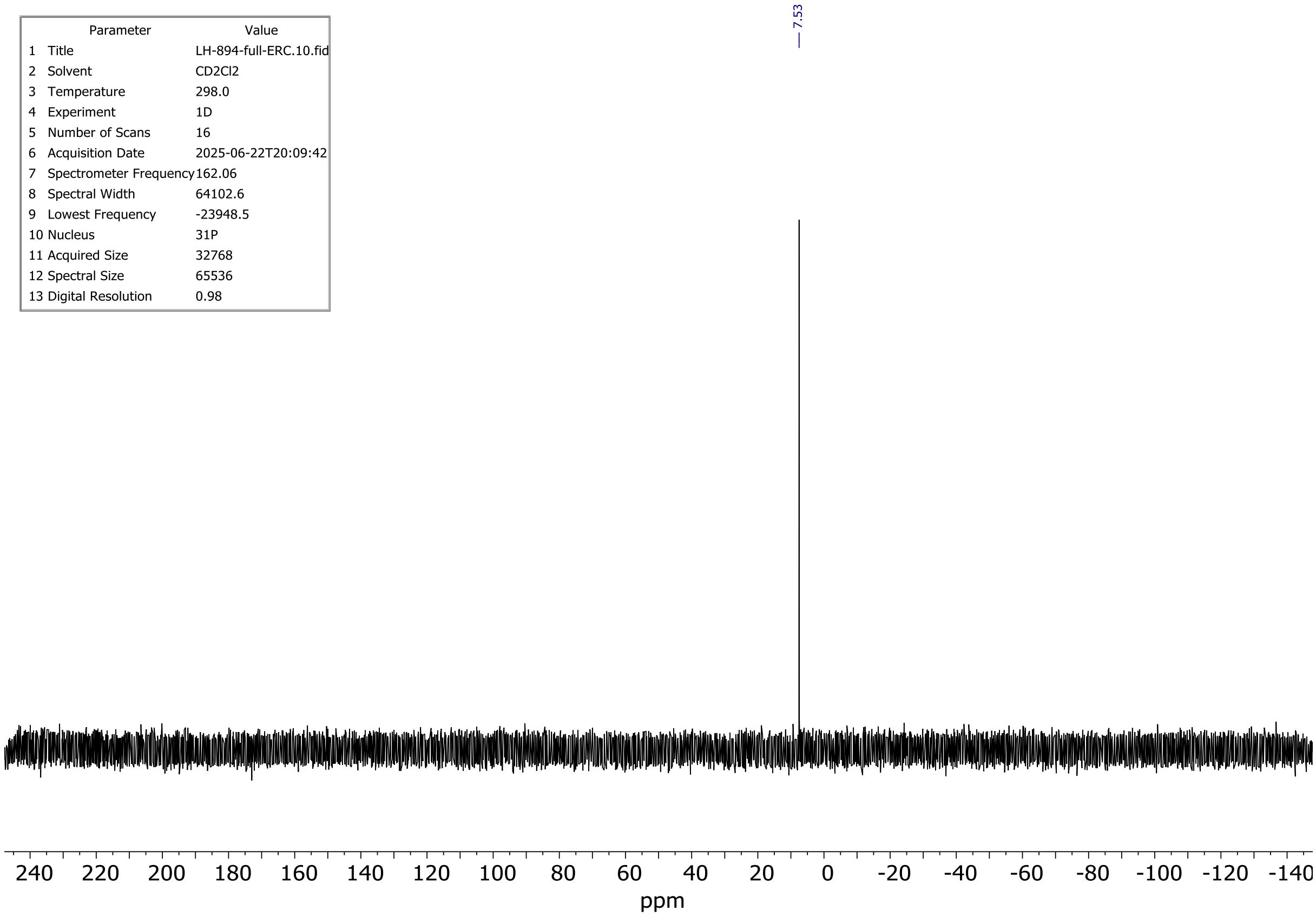

Figure S68: $^{31}P\{^{1}H\}$ NMR spectrum of **5_{Ph}OTf** in CD_2Cl_2.

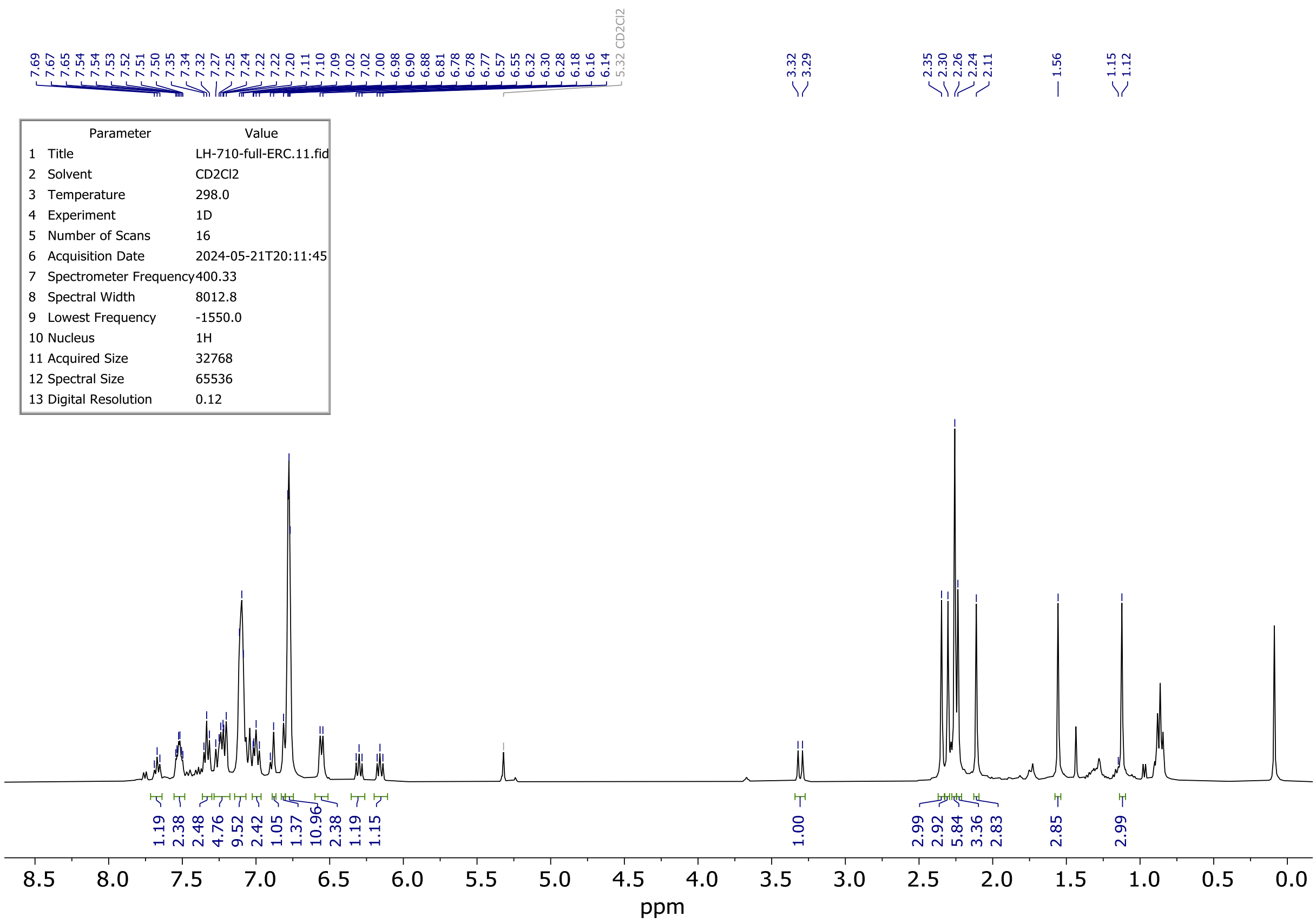

Figure S69: ^{1}H NMR spectrum of **$5_{Ph}BPh_4$** in CD_2Cl_2.

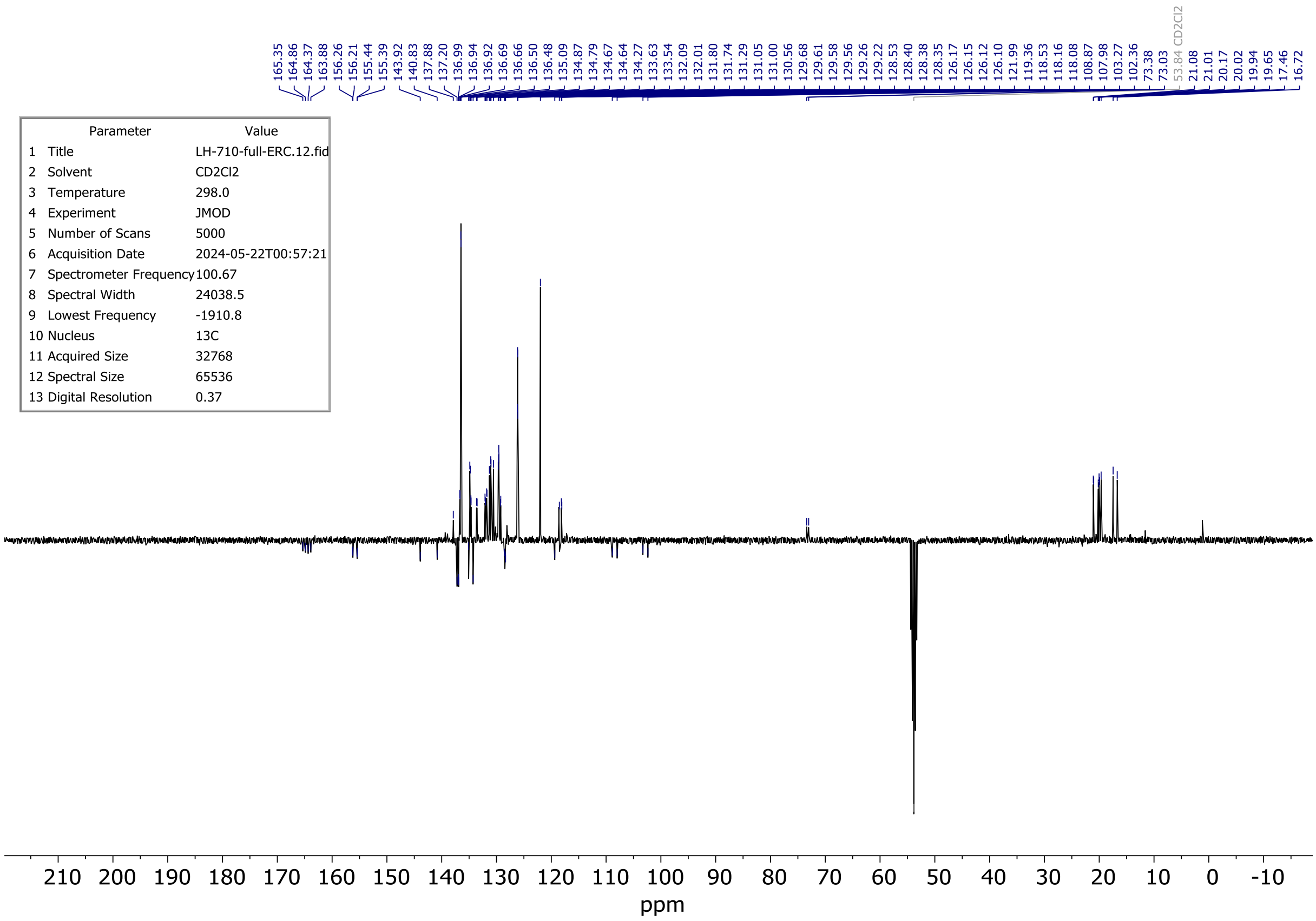

Figure S70: $^{13}C\{^{1}H\}$ NMR spectrum of $\mathbf{5_{Ph}BPh_4}$ in CD_2Cl_2.

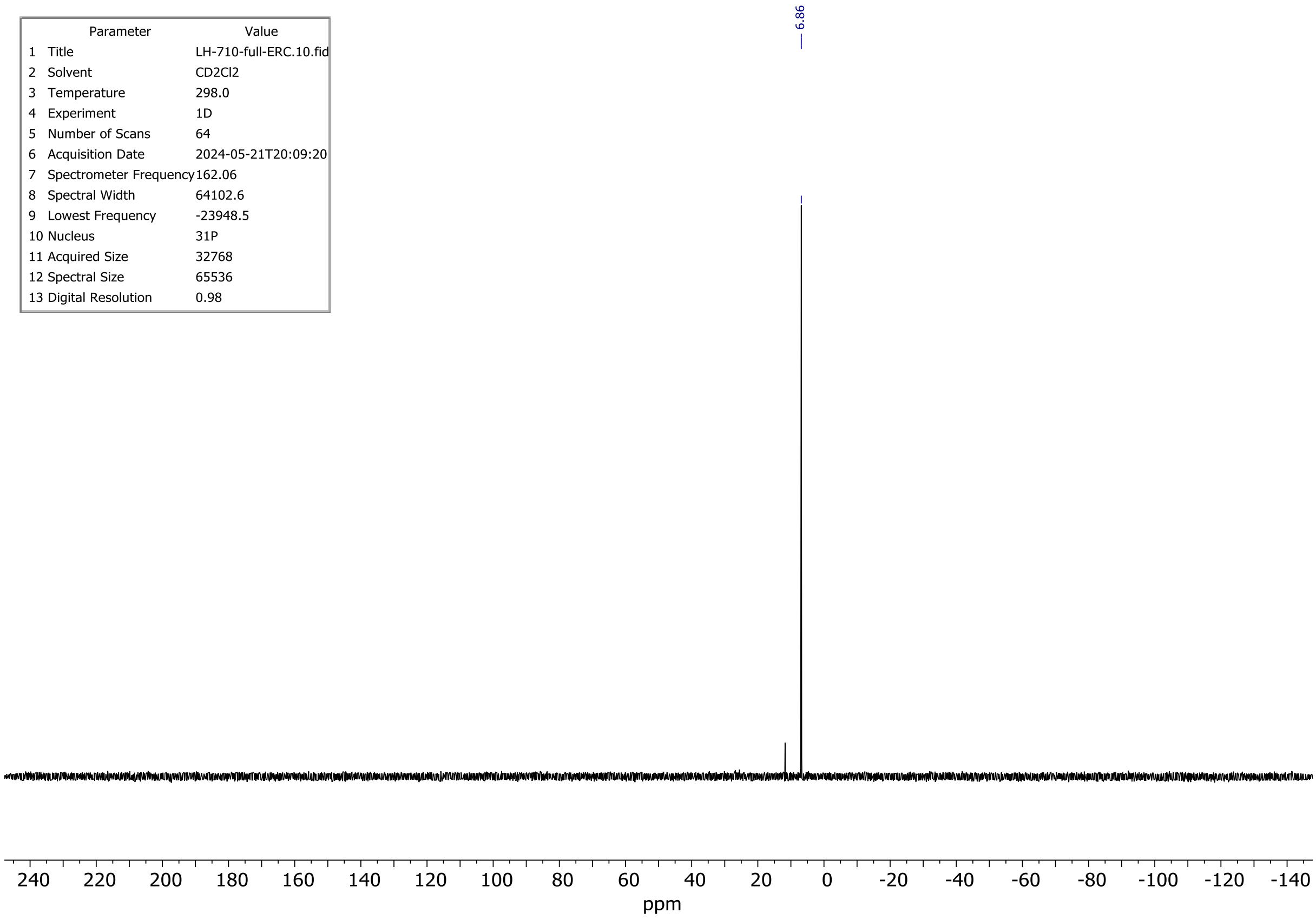

Figure S71: $^{31}P\{^{1}H\}$ NMR spectrum of $\mathbf{5_{Ph}BPh_4}$ in CD_2Cl_2.

C

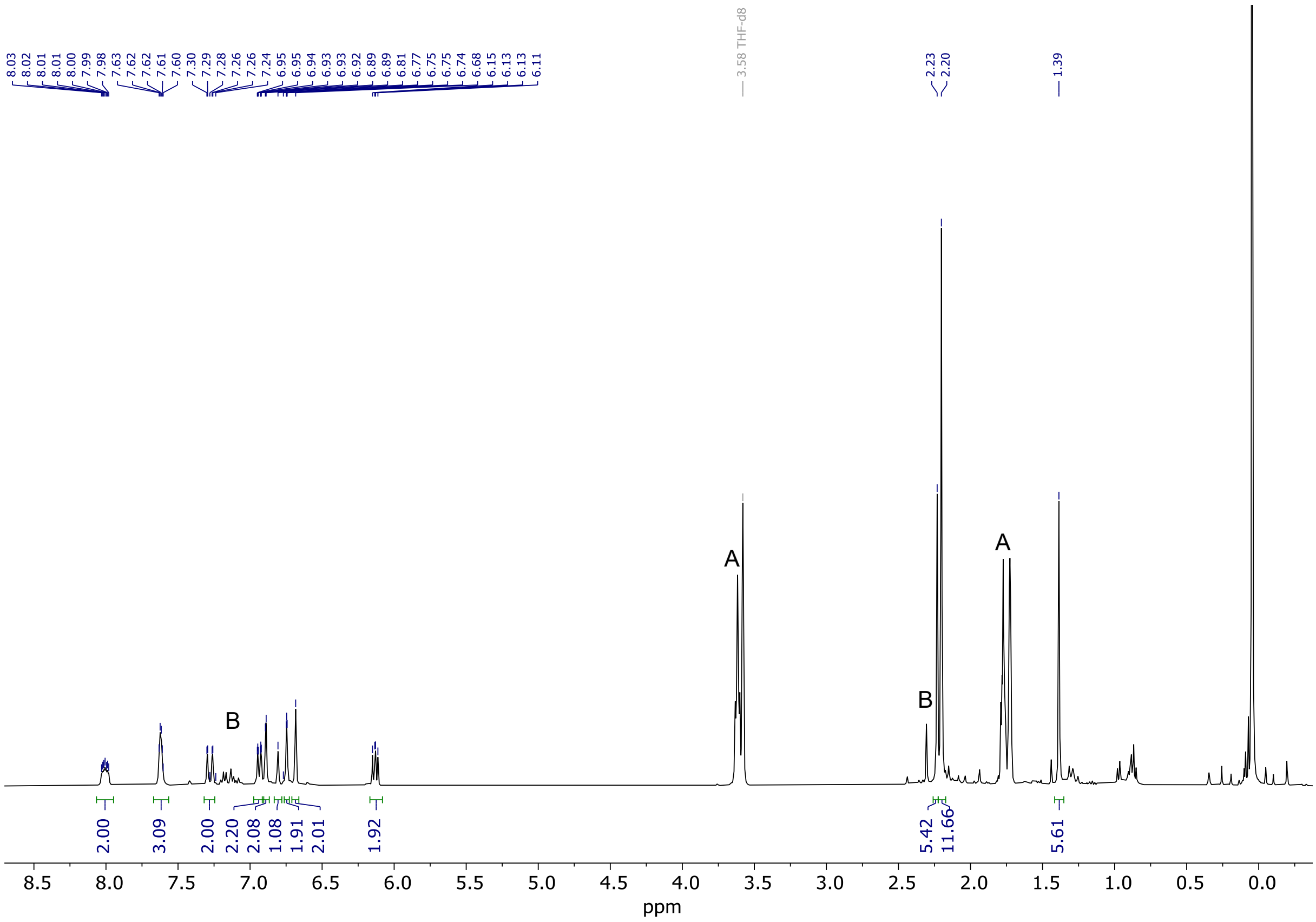

Figure S72: ^{1}H NMR spectrum of **6** in THF-d_8. Symbols for residues: A: THF, B: Toluene, C: HMDS

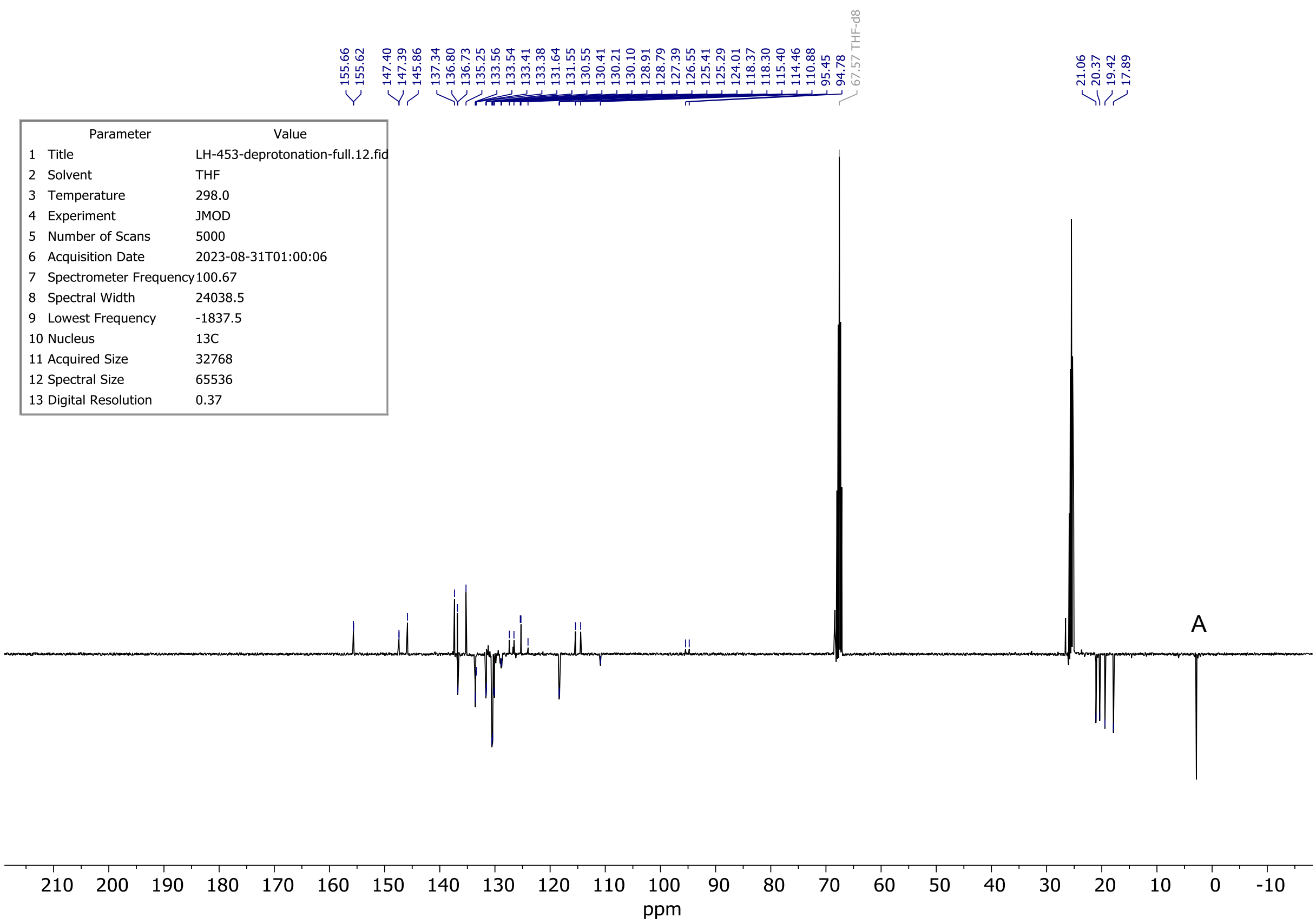

Figure S73: $^{13}C\{^{1}H\}$-APT NMR spectrum of **6** in THF-d_8. Symbols for residues: A: HMDS

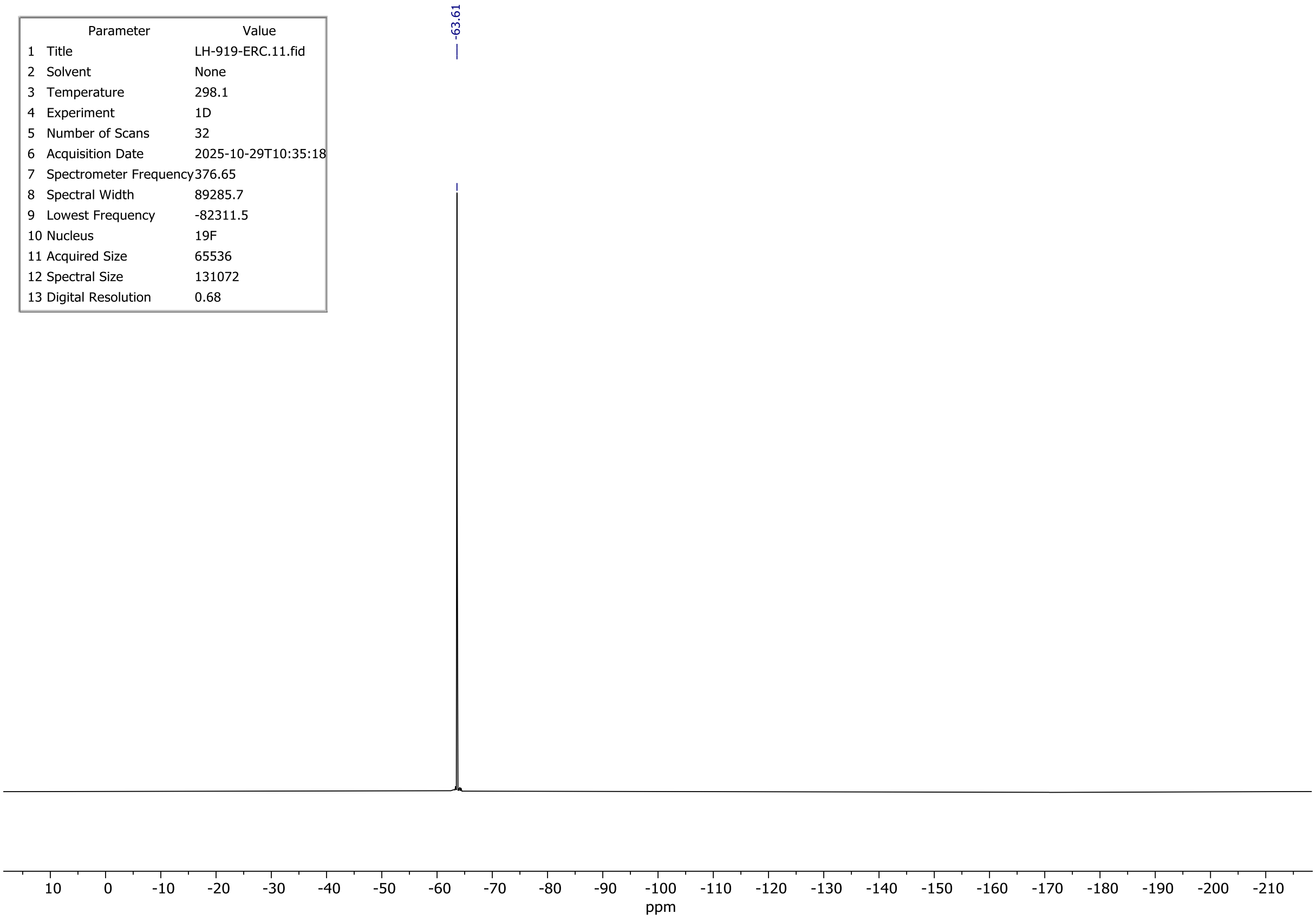

Figure S74: $^{19}F\{^1H\}$ NMR spectrum of **6** in THF.

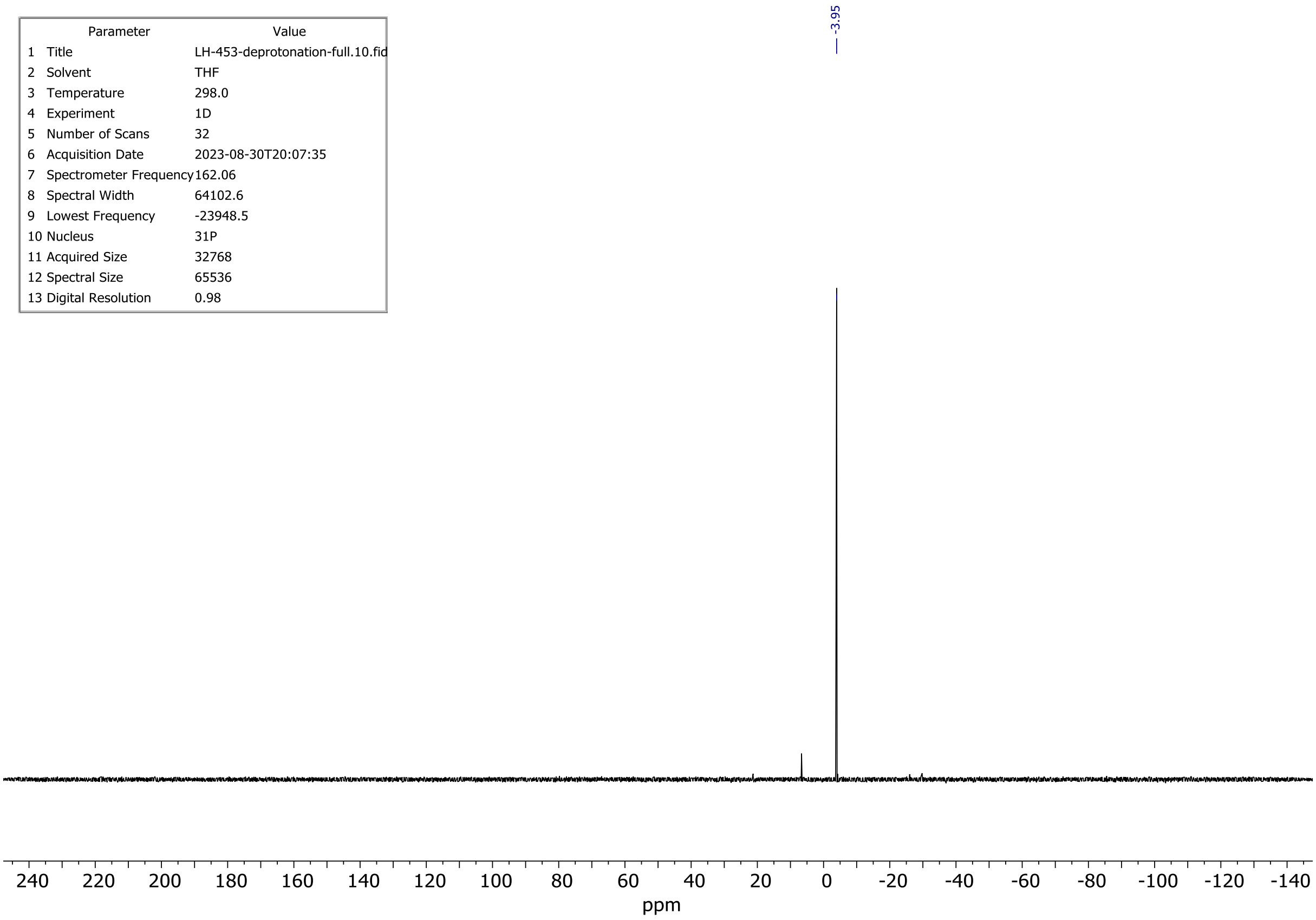

Figure S75: $^{31}P\{^1H\}$ NMR spectrum of **6** in THF-d_8.

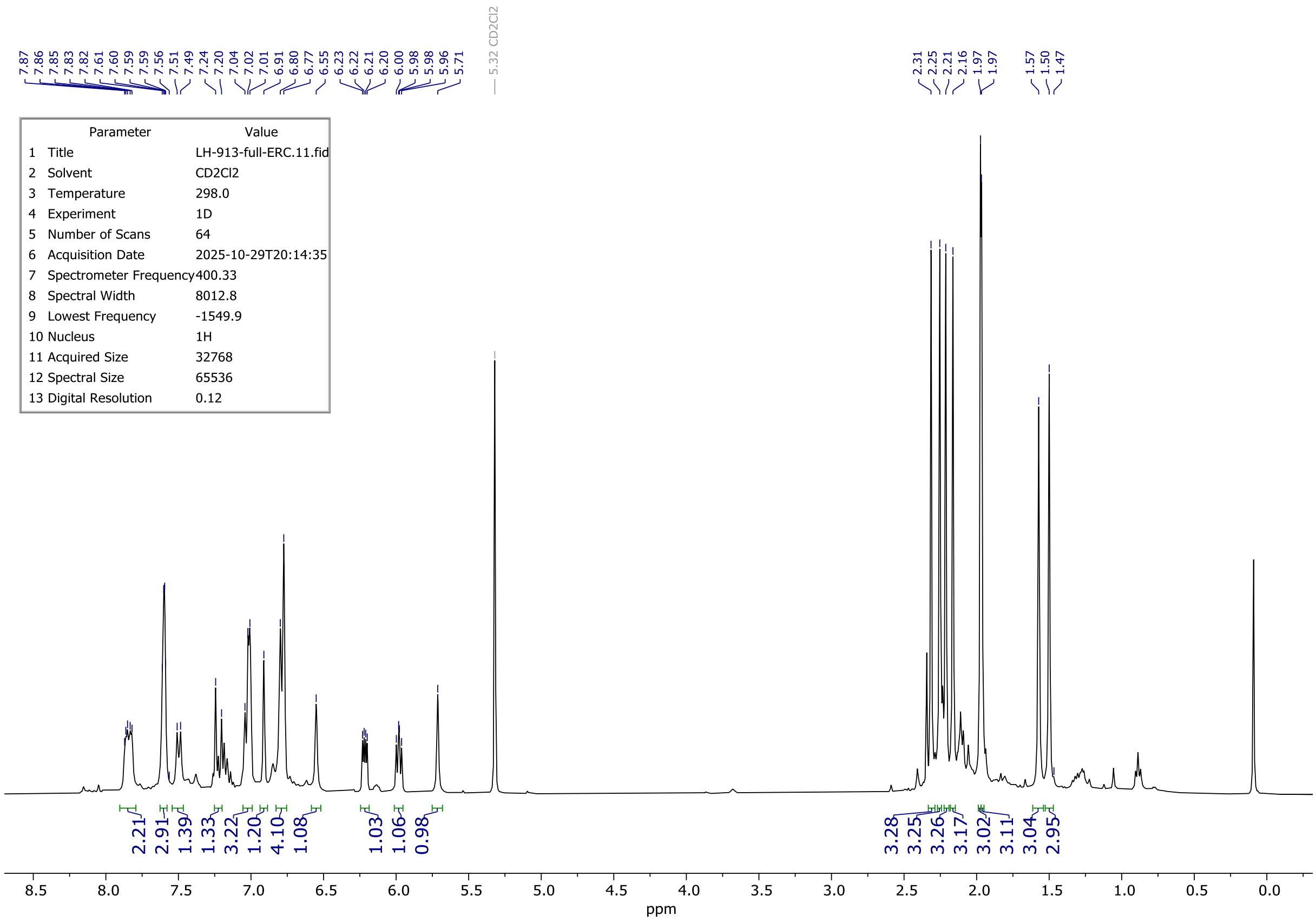

Figure S76: ^{1}H NMR spectrum of **7** in CD_2Cl_2.

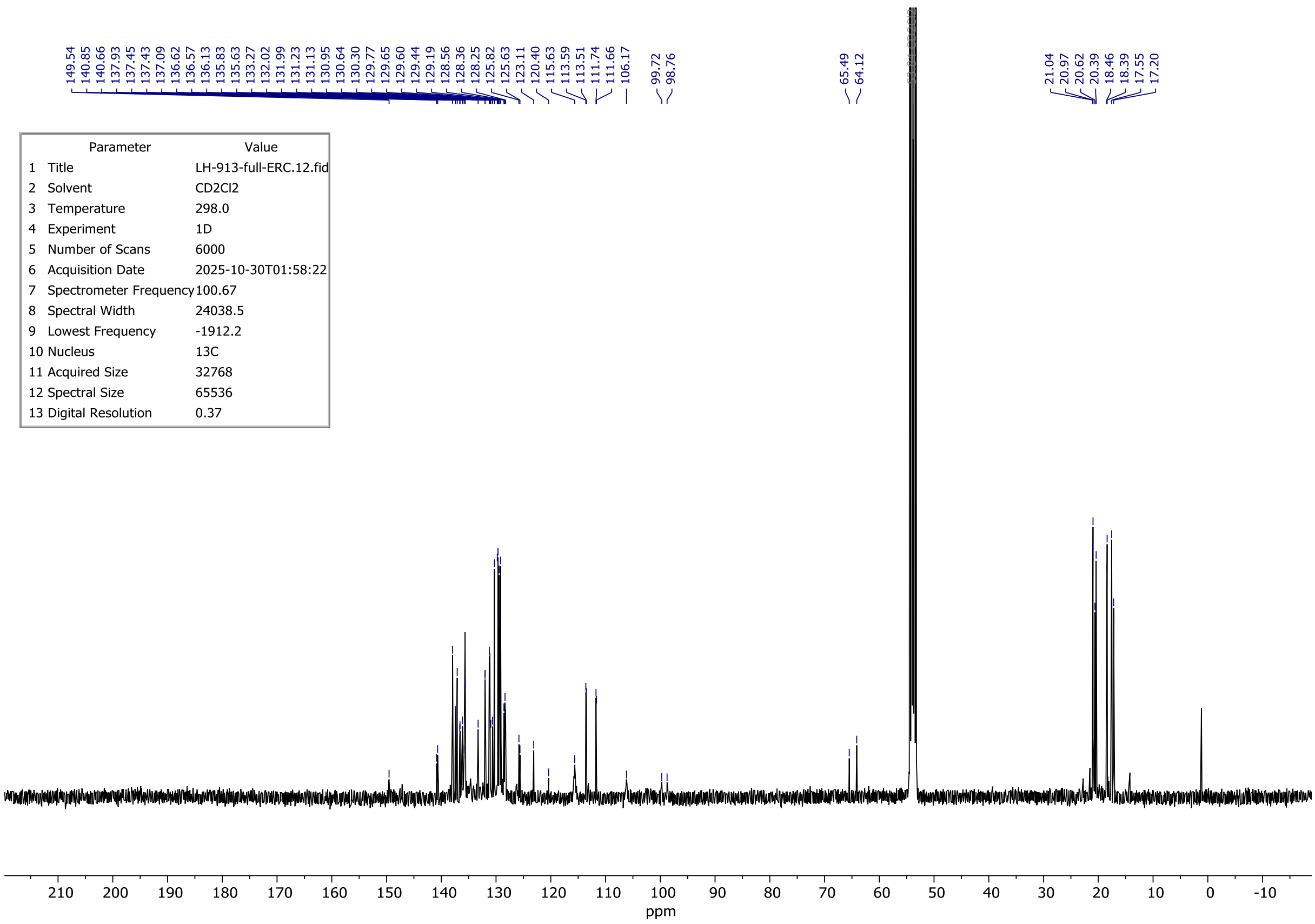

Figure S77: $^{13}C\{^{1}H\}$ NMR spectrum of **7** in CD_2Cl_2.

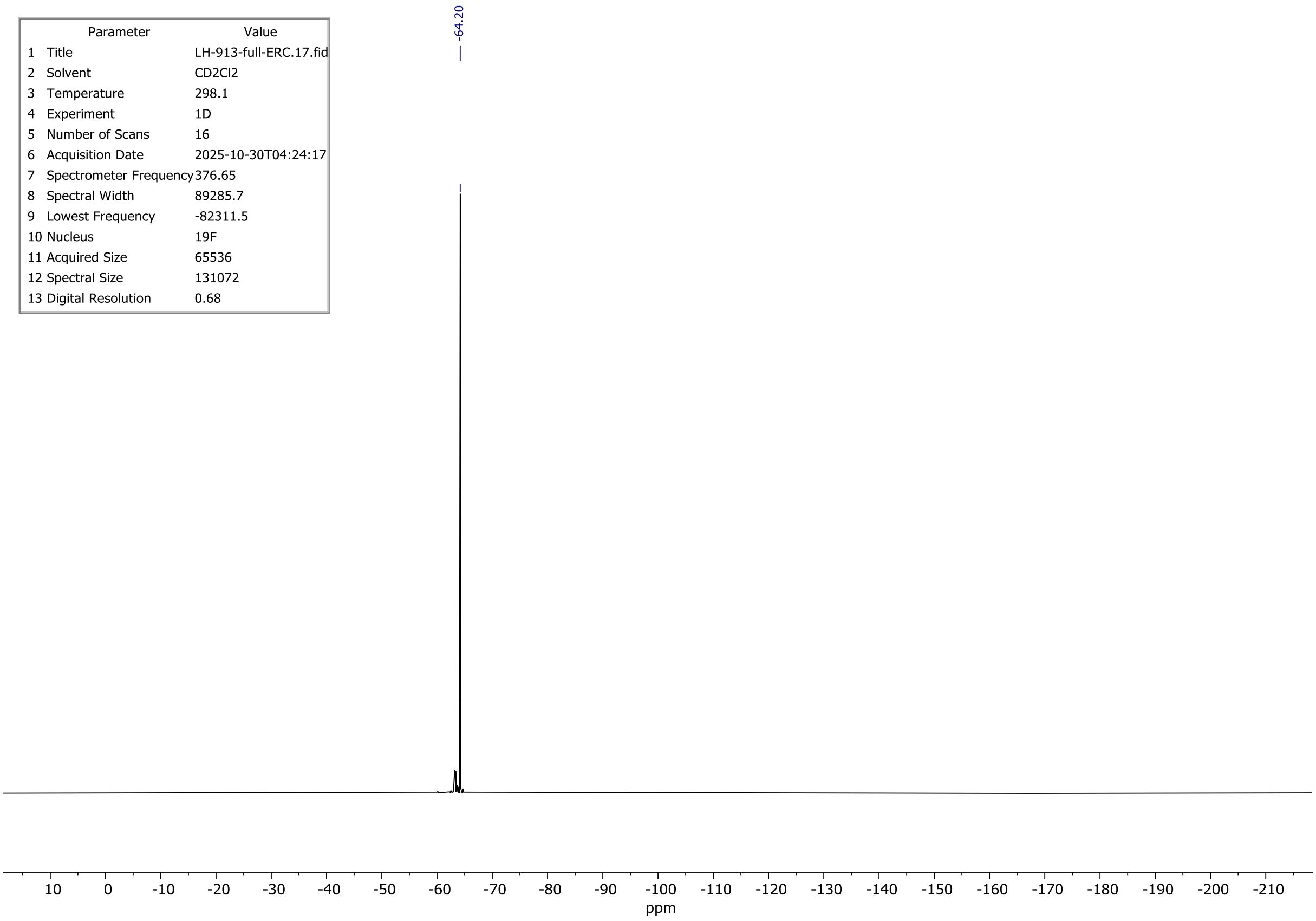

Figure S78: $^{19}F\{^{1}H\}$ NMR spectrum of **7** in CD_2Cl_2.

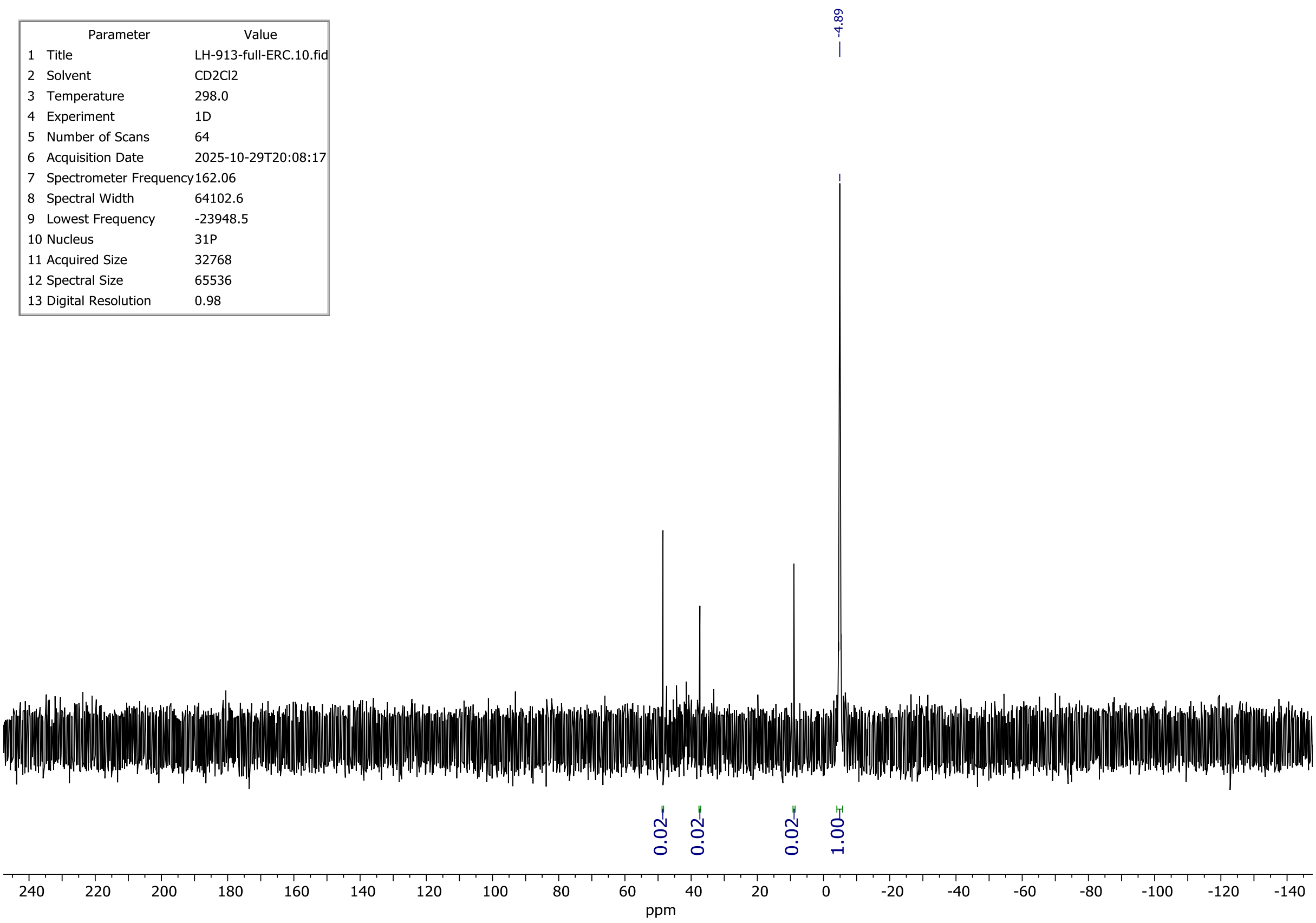

Figure S79: $^{31}P\{^{1}H\}$ NMR spectrum of **7** in CD_2Cl_2.

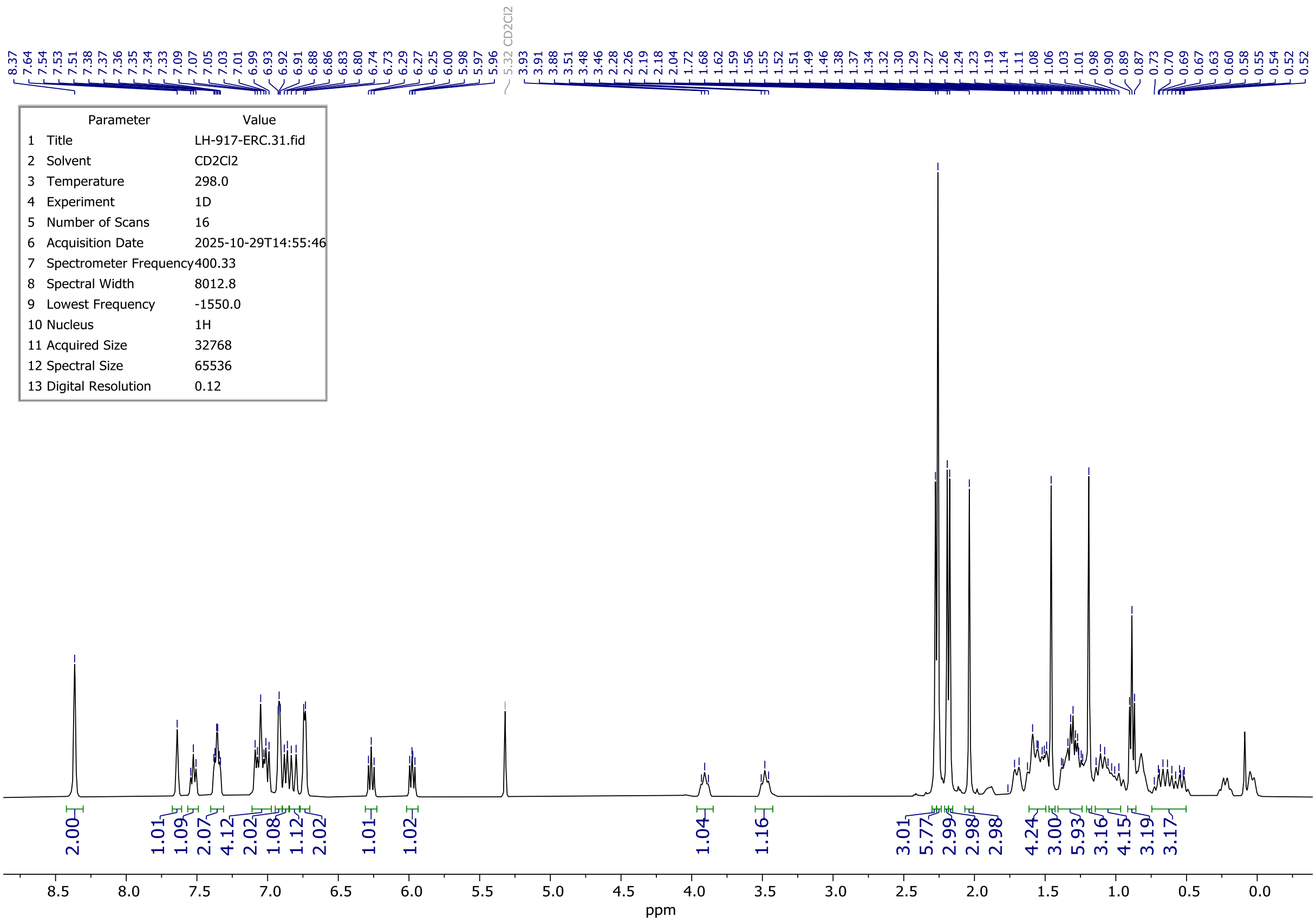

Figure S80: ^{1}H NMR spectrum of **8** in CD_2Cl_2.

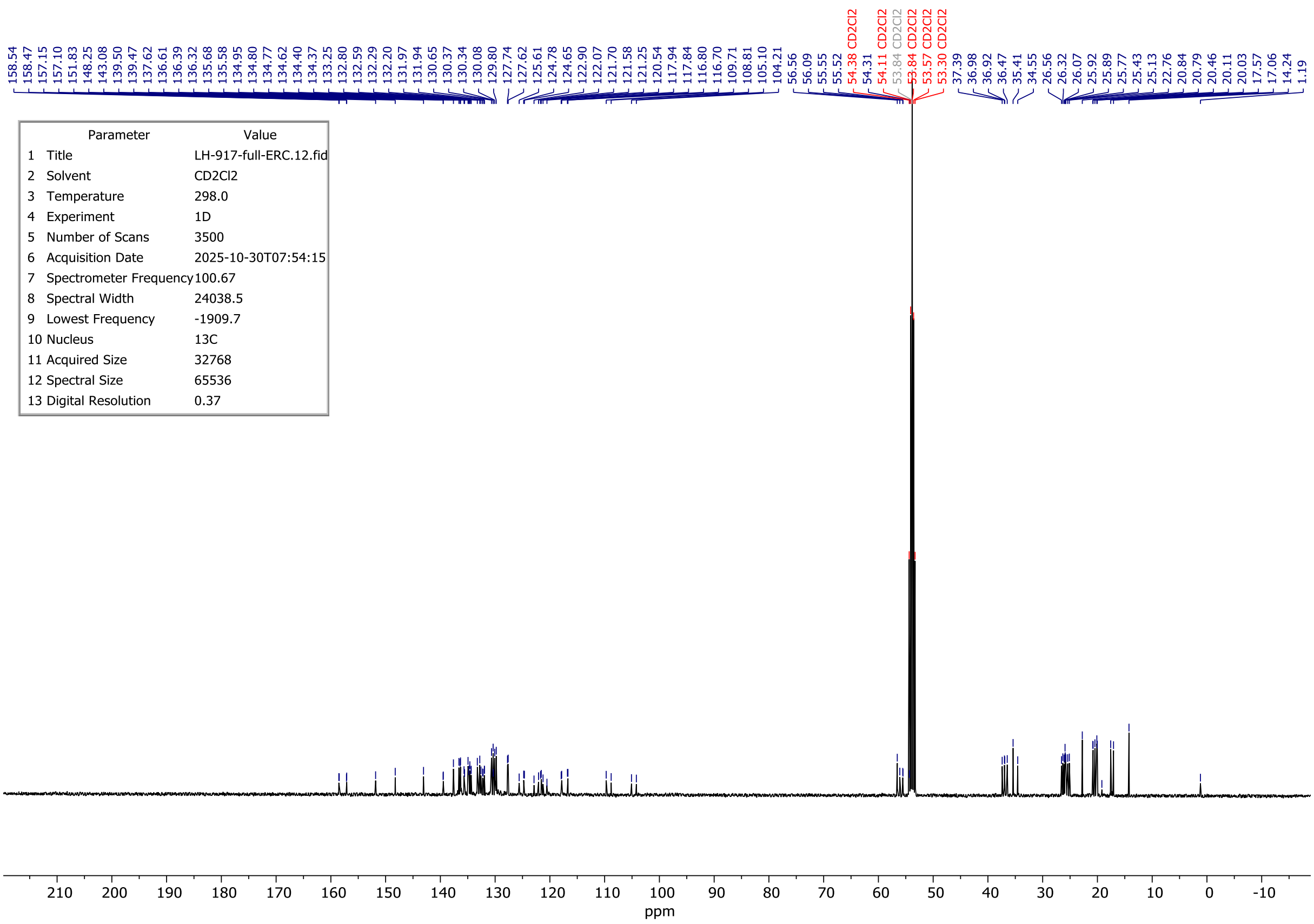

Figure S81: $^{13}C\{^{1}H\}$ NMR spectrum of **8** in CD_2Cl_2.

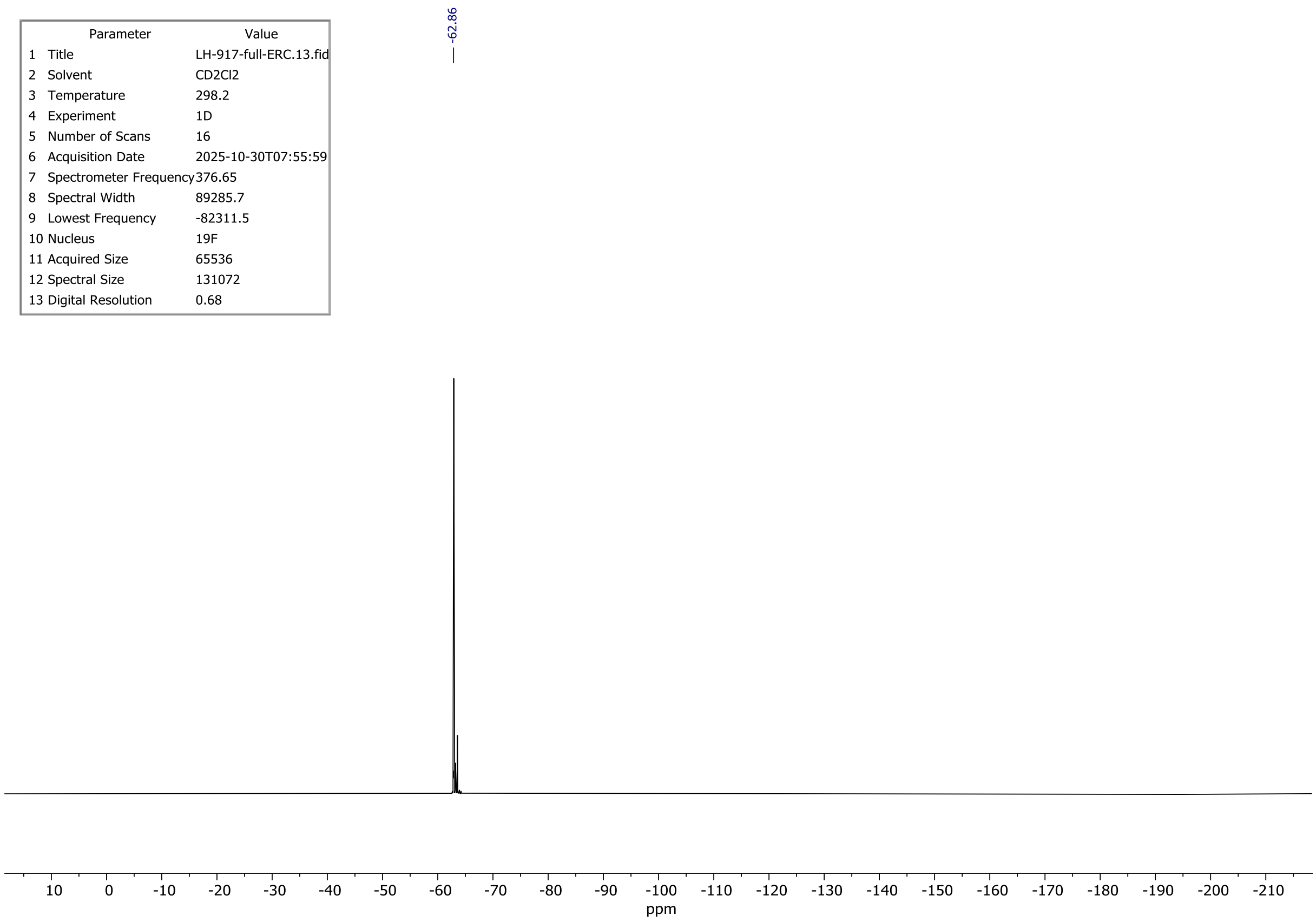

Figure S82: $^{19}F\{^1H\}$ NMR spectrum of **8** in CD_2Cl_2.

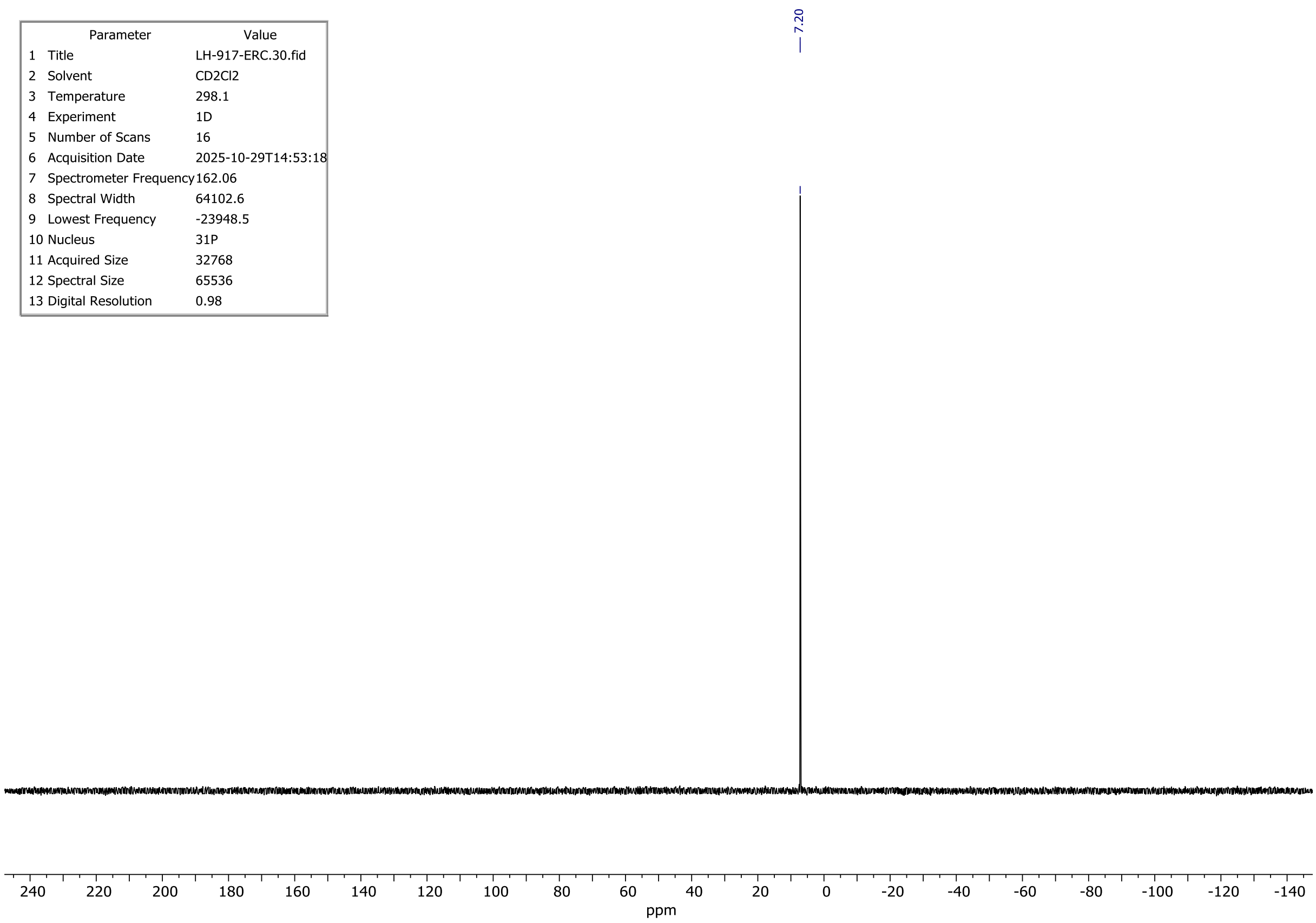

Figure S83: $^{31}P\{^1H\}$ NMR spectrum of **8** in CD_2Cl_2.

6 Single-crystal structure analyses

6.1 Data collection and structure refinement details

Table S1 Data collection and structure refinement details for compounds **2_{Ph}I**, **3_H**, **3_{Ph}** and **3_{ArF}**.

**Compound**	**2_{Ph}I**	**3_H**	**3_{Ph}**	**3_{ArF}**
Formula	$C_{45}H_{48}BiN_2PI$	$C_{39}H_{43}N_2P$	$C_{45}H_{47}N_2P$	$C_{52}H_{57}F_6N_2P$
CCDC	2484196	2484198	2484199	2484197
Formula weight	774.72	570.72	646.81	854.96
Temperature [K]	100(2) K	100(2) K	100(2)	100(2)
Wave length [Å]	1.54184 Å	1.54184 Å	1.54184 Å	1.54184 Å
Crystal system	Monoclinic	Monoclinic	Orthorhombic	Monoclinic
Space group	$P2_1/n$	$P2_1/c$	$Pna2_1$	$P2_1/c$
a [Å]	14.2719(2)	11.8303(2)	14.6291(1)	13.2955(5)
b [Å]	22.8068(2)	16.6691(2)	12.9557(1)	22.0693(7)
c [Å]	15.6246(2)	17.0663(2)	19.3514(2)	15.8266(4)
α[°]	90	90	90	90
β [°]	111.820(1)	105.384(1)	90	90.597(3)
γ [°]	90	90	90	90
Volume [$Å^3$]	4721.39(10)	3244.89(8)	3667.68(5)	4643.6(3)
Z	4	4	4	4
Calc. density [$Mg\cdot m^{-3}$]	1.090	1.168	1.171	1.223
μ (MoKα) [mm^{-1}]	5.846	0.957	0.905	1.027
F(000)	1600	1224	1384	1808
Crystal dimensions [mm]	0.300 x 0.060 x 0.050	0.880 x 0.630 x 0.390	0.103 x 0.091 x 0.048	0.259 x 0.091 x 0.080
Theta range θ [°]	3.586 to 77.273	3.775 to 76.934	4.106 to 76.839	3.324 to 67.980
Index ranges	-16<=h<=18	-14<=h<=14	-17<=h<=18	-15<=h<=15
	-24<=k<=28	-17<=k<=20	-16<=k<=15	-26<=k<=22
	-19<=l<=18	-21<=l<=20	-24<=l<=21	-19<=l<=17
Reflections collected	63347	23827	36610	32305
Independent reflections	9811 [R(int) = 0.0487]	6631 [R(int) = 0.0366]	7169 [R(int) = 0.049]	8392 [R(int) = 0.114]
Data/Restraints/ Parameters	9811 / 0 / 456	6631 / 0 / 391	7169 / 1 / 444	8392 / 0 / 571
Goodness-of-fit on F^2	A 1.014	1.069	1.037	1.068
Final R indices [I>2sigma(I)]	R1 = 0.0333, wR2 = 0.0772	R1 = 0.0410, wR2 = 0.1103	R1 = 0.0377, wR2 = 0.1011	R1 = 0.0682, wR2 = 0.1915
Largest diff. peak and hole [$e\cdot Å^{-3}$]	1.213 and -0.640	0.349 and -0.361	0.336 and -0.286	0.513 and -0.485

Table S2 Data collection and structure refinement details for compounds **4_{Ph}Cl**, **4_{Ph}Br**, **4_{Ph}I** and **4_{H}Cl**.

**Compound**	**4_{Ph}Cl**	**4_{Ph}Br**	**4_{Ph}I**	**4_{H}Cl**
Formula	$C_{48}H_{52}BiClN_2P$	$C_{52}H_{53}BiBrN_2P$	$C_{51}H_{51}BiIN_2P$	$C_{78}H_{82}Bi_2Cl_2N_4P_2$
CCDC	2484203	2484202	2484204	2484201
Formula weight	932.31	1025.82	1058.79	1626.27
Temperature [K]	110(2)	100(2)	100(2)	100(2)
Wave length [Å]	1.54184	1.54184	1.54184	1.54184
Crystal system	Triclinic	triclinic	monoclinic	Orthorhombic
Space group	P-1	P -1	I 2/a	Pbca
a [Å]	12.8268(3)	12.7509(3)	27.6469(4)	15.5611(2)
b [Å]	12.8714(2)	12.8286(3)	12.9984(2)	13.3542(2)
c [Å]	15.6323(2)	14.5704(3)	24.6924(4)	33.0480(4)
α[°]	85.511(1)	91.049(2)	90	90
β [°]	69.480(2)	109.576(2)	92.939(1)	90
γ [°]	88.620(2)	91.307(2)	90	90
Volume [$Å^3$]	2409.69(8)	2244.17(9)	8861.9(2)	6867.57(16)
Z	2	2	8	4
Calc. density [$Mg \cdot m^{-3}$]	1.285	1.518	1.587	1.573
μ (MoKα) [mm^{-1}]	8.226	9.361	13.888	11.451
F(000)	938	1024	4176	3232
Crystal dimensions [mm]	0.525 x 0.095 x 0.074	0.458 x 0.095 x 0.037	0.256 x 0.151 x 0.072	0.150 x 0.120 x 0.050
Theta range θ [°]	3.027 to 67.079	3.220 to 67.078	3.201 to 67.080	2.674 to 67.997
Index ranges	-15<=h<=15	-13 <= h <= 15	-31 <= h <= 33	-13<=h<=18
	-15<=k<=15	-15 <= k< = 15	-15 <= k< = 15	-13<=k<=16
	-18<=l<=18	-17 <= l< = 17	-25 <= l< = 29	-39<=l<=39
Reflections collected	80565	27784	28738	26788
Independent reflections	8604 [R(int) = 0.1038]	8007 [Rint = 0.0689]	7869 [Rint = 0.0553]	6229 [R(int) = 0.042]
Data/Restraints/ Parameters	8604 / 0 / 487	8007 / 129 / 564	7869 / 48 / 544	6229 / 0 / 405
Goodness-of-fit on F^2	1.046	1.019	1.077	1.035
Final R indices [I>2sigma(I)]	R1 = 0.0334, wR2 = 0.0883	R1 = 0.0392, wR2 = 0.0982	R1 = 0.0356, wR2 = 0.0968	R1 = 0.0272, wR2 = 0.0651
Largest diff. peak and hole [$e \cdot Å^{-3}$]	1.804 and -1.558	2.145 and -1.706	0.902 and -1.375	1.156 and -1.182

Table S3 Data collection and structure refinement details for compounds **4_{ArF}Cl**, **4_{ArF}Br**, **5_{Ph}OTf** and **5_{Ph}BPh$_4$**.

Compound	4_{ArF}Cl	4_{ArF}Br	5_{Ph}OTf	5_{Ph}BPh$_4$
Formula	$C_{106}H_{98}4Bi_2Cl_2F_{12}N_4P_2$	$C_{94}H_{86}Bi_2Br_2F_{12}N_4P_2$	$C_{46}H_{45}BiF_3N_2O_3PS$	$C_{71}H_{69}BBiCl_4N_2P$
CCDC	2484209	2484200	2484206	2484205
Formula weight	2206.68	2139.38	1002.85	1342.84
Temperature [K]	100(2)	100(2)	100(2)	100(2)
Wave length [Å]	1.54184	1.54184	1.54184	1.54184
Crystal system	Triclinic	Monoclinic	Monoclinic	Monoclinic
Space group	P-1	$P2_1/n$	$P2_1/c$	$P2_1/n$
a [Å]	12.3033(3)	13.3330(2)	20.4266(4)	12.1323(1)
b [Å]	13.7343(4)	12.3537(2)	12.2189(2)	20.5536(1)
c [Å]	15.7499(4)	25.1415(4)	19.3312(4)	26.2709(2)
α[°]	77.511(2)	90	90	90
β [°]	69.787(2)	93.048(2)	113.341(2)	100.724(1)
γ [°]	69.812(2)	90	90	90
Volume [Å^3]	2329.78(11)	4135.25(11)	4430.03(16)	6436.56(8)
Z	1	2	4	4
Calc. density [Mg·m^{-3}]	1.573	1.718	1.504	1.386
μ (MoKα) [mm^{-1}]	8.803	10.405	9.035	7.455
F(000)	1100	2104	2000	2720
Crystal dimensions [mm]	0.265 x 0.135 x 0.105	0.110 x 0.050 x 0.040	0.123 x 0.092 x 0.055	0.110 x 0.100 x 0.050
Theta range θ [°]	3.008 to 67.074	3.521 to 67.993	4.319 to 67.994	2.748 to 67.993
Index ranges	-14<=h<=14	-16<=h<=14	-21<=h<=24	-14<=h<=14
	-16<=k<=15	-14<=k<=11	-14<=k<=14	-24<=k<=9
	-18<=l<=18	-29<=l<=30	-23<=l<=23	-31<=l<=31
Reflections collected	67393	27949	30795	44007
Independent reflections	8258 [R(int) = 0.1136]	7508 [R(int) = 0.0467]	8070 [R(int) = 0.048]	11681 [R(int) = 0.042]
Data/Restraints/ Parameters	8258 / 72 / 616	7508 / 0 / 531	8070 / 0 / 522	11681 / 0 / 729
Goodness-of-fit on F^2	1.124	1.035	1.026	1.046
Final R indices [I>2sigma(I)]	R1 = 0.0354, wR2 = 0.0940	R1 = 0.0281, wR2 = 0.0664	R1 = 0.0327, wR2 = 0.0796	R1 = 0.0336, wR2 = 0.0898
Largest diff. peak and hole [e·Å^{-3}]	1.029 and -1.648	1.027 and -0.922	1.899 and -1.271	1.336 and -1.447

Table S4 Data collection and structure refinement details for compounds **7** and **8**.

**Compound**	**7**	**8**
Formula	$C_{52.26}H_{51.69}F_6N_{3.13}P$	$C_{60}H_{64}BiF_6N_4P$
CCDC	2484207	2484208
Formula weight	868.52	1195.10
Temperature [K]	100(2)	110(2)
Wave length [Å]	1.54184	1.54184
Crystal system	Triclinic	Monoclinic
Space group	P-1	C2/c
a [Å]	9.2327(2)	25.4693(4)
b [Å]	13.7689(3)	14.2636(2)
c [Å]	19.4175(4)	32.2809(5)
α[°]	80.241(2)	90
β [°]	88.179(2)	104.482(2)
γ [°]	87.929(2)	90
Volume [Å^3]	2430.30(9)	11354.5(3)
Z	2	8
Calc. density [Mg·m^{-3}]	1.187	1.398
μ (MoKα) [mm^{-1}]	0.999	6.855
F(000)	912	4832
Crystal dimensions [mm]	0.674 x 0.116 x 0.065	0.240 x 0.140 x 0.050
Theta range θ [°]	2.310 to 67.079	2.828 to 67.997
Index ranges	-11<=h<=10	-30<=h<=30
	-16<=k<=16	-17<=k<=16
	-17<=l<=23	-36<=l<=38
Reflections collected	28364	38322
Independent reflections	8624 [R(int) = 0.0524]	10257 [R(int) = 0.0298]
Data/Restraints/ Parameters	8624 / 148 / 626	10257 / 42 / 685
Goodness-of-fit on F^2	1.059	1.068
Final R indices [I>2sigma(I)]	R1 = 0.0819, wR2 = 0.2421	R1 = 0.0272, wR2 = 0.0666
Largest diff. peak and hole [e·Å^{-3}]	0.939 and -0.927	0.905 and -1.155

6.2 ORTEP plots of all crystal structures

6.2.1 Molecular structure of $2_{Ph}I$

All hydrogen atoms were placed on ideal positions.

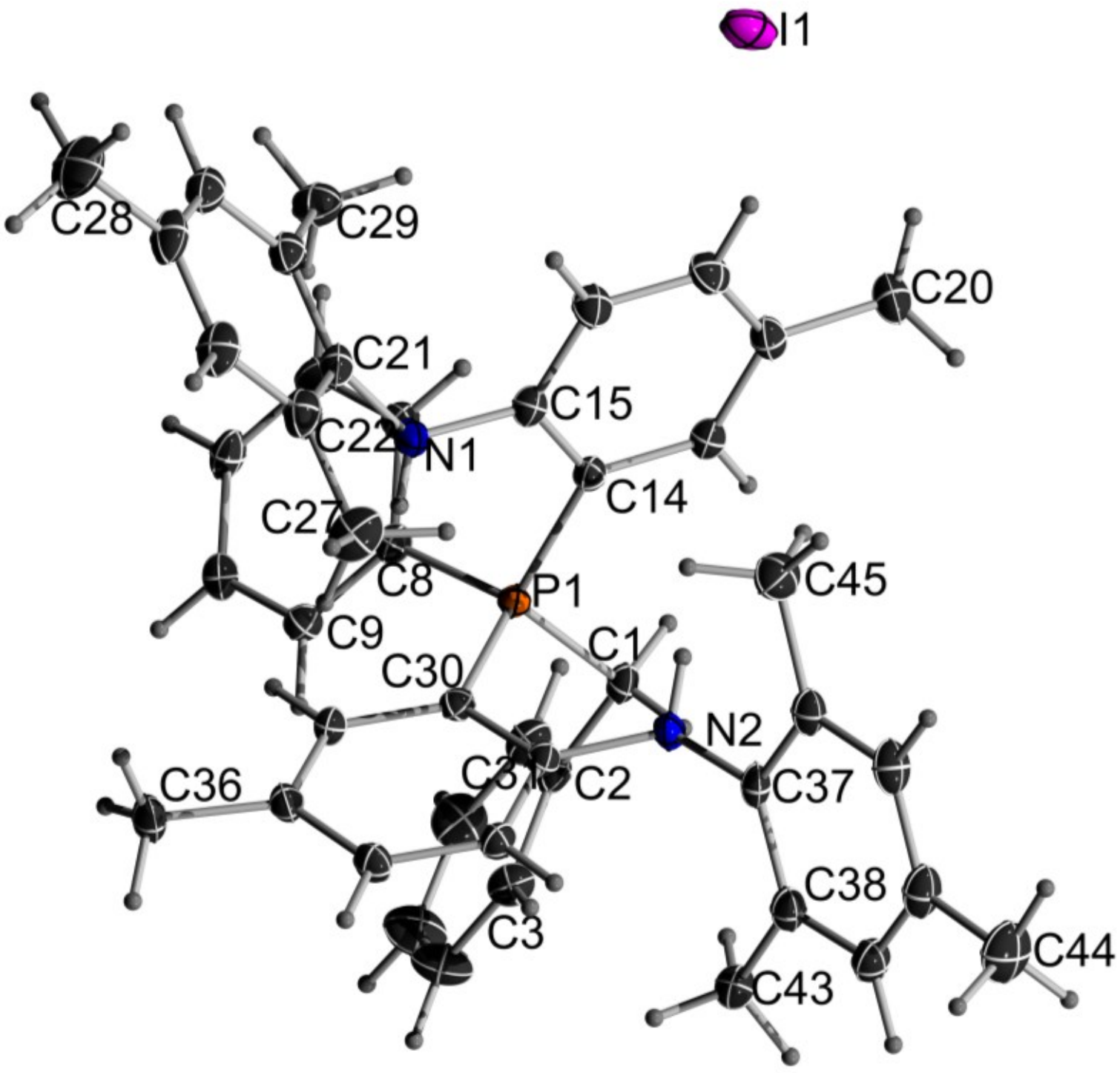

Figure S84: ORTEP of **2_{Ph}I**. Ellipsoids drawn at 50 % probability. Selected bond lengths [Å] and angles [°]: P1-C1 1.825(2), C1-C2 1.511(3), C2-C1-P1 110.1(1).

6.2.2 Molecular Structure of 3_H

All hydrogen atoms except the one attached to N1 were placed on ideal positions.

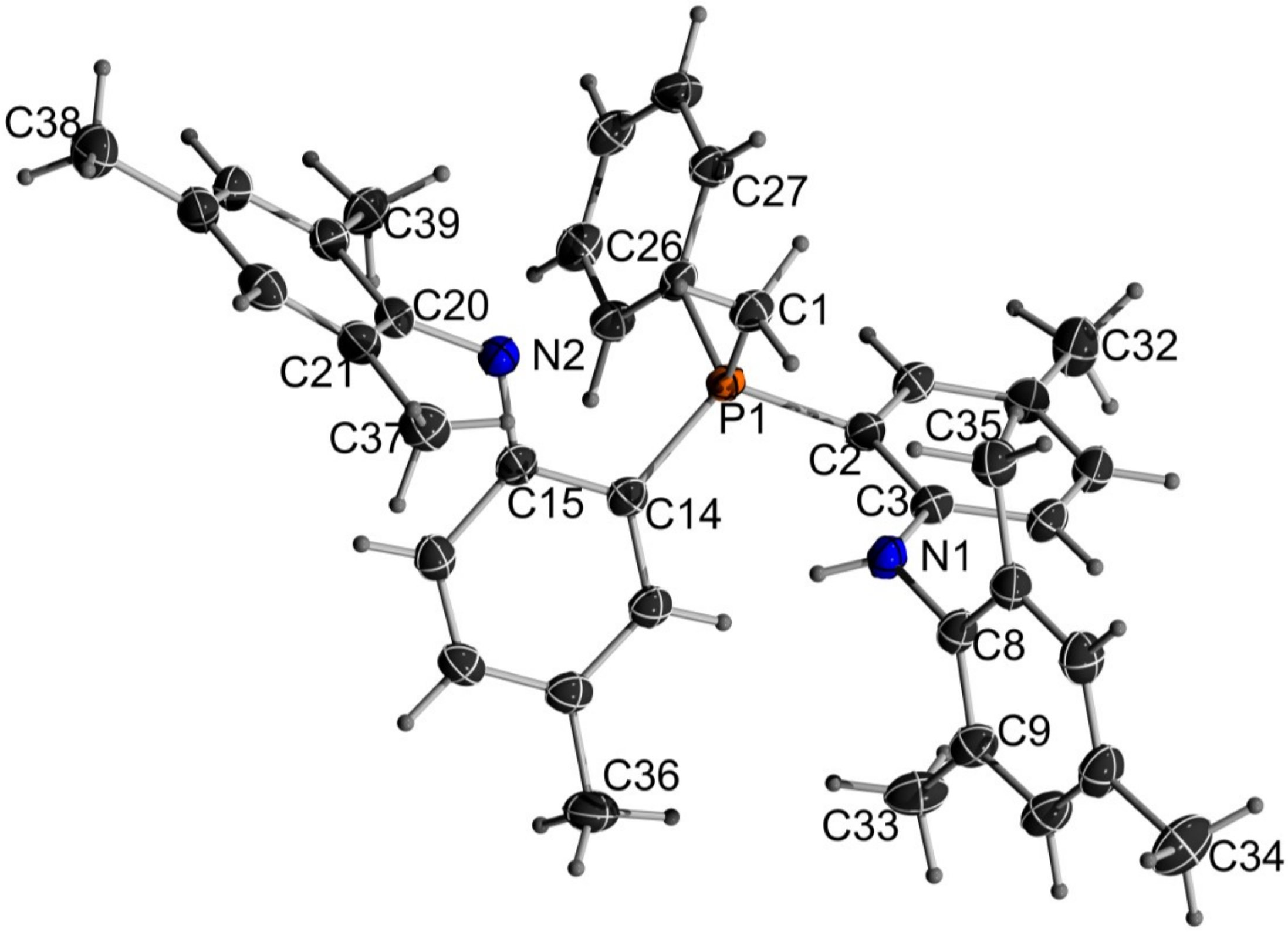

Figure S85: ORTEP of $\mathbf{3_H}$. Ellipsoids drawn at 50 % probability. Selected bond lengths [Å] and angles [°]: P1-C1 1.801(1).

6.2.3 Molecular Structure of 3_{Ph}

All hydrogen atoms except the one attached to N1 were placed on ideal positions.

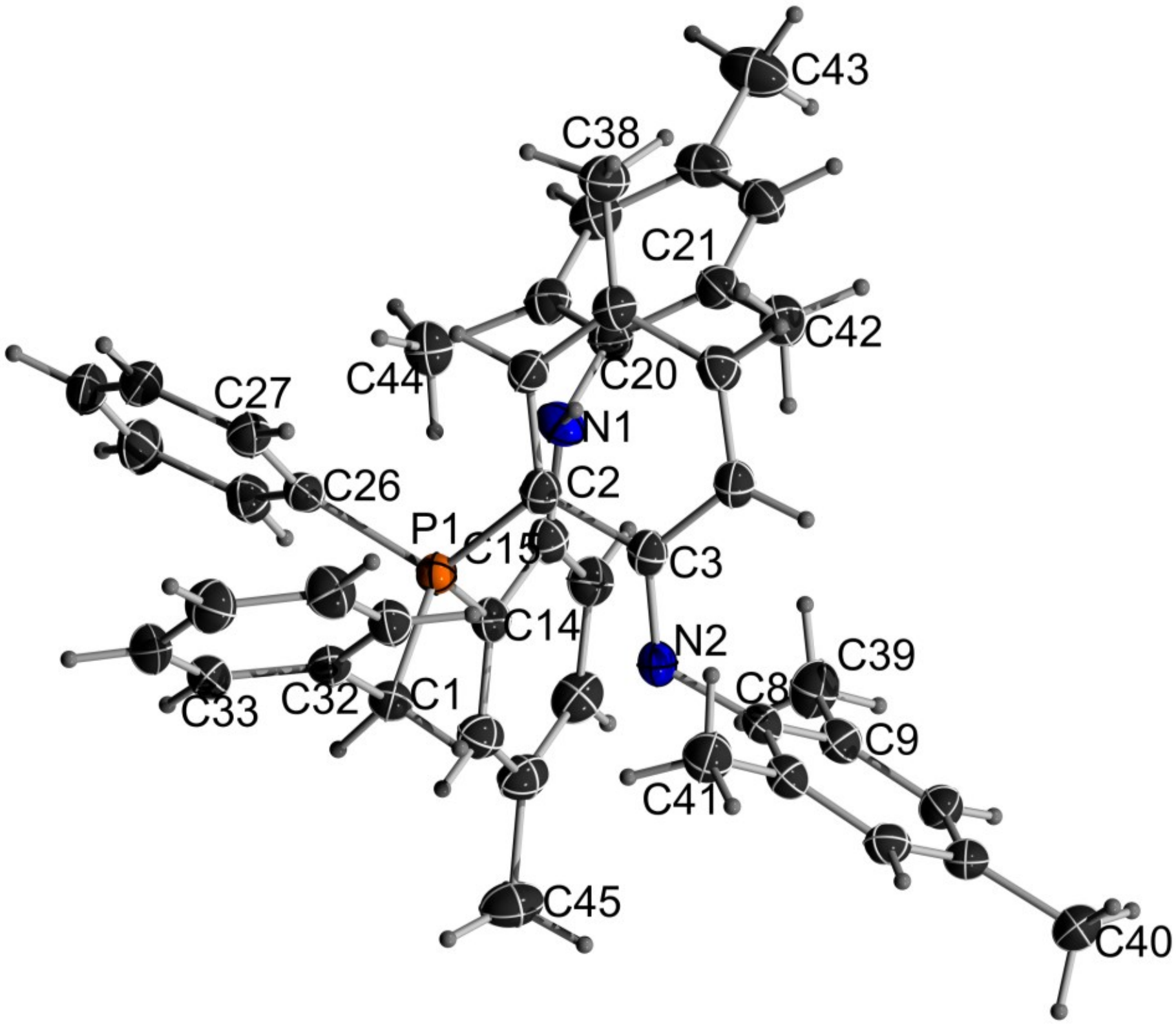

Figure S86: ORTEP of $\mathbf{3}_{\mathbf{Ph}}$. Ellipsoids drawn at 50 % probability. Selected bond lengths [Å] and angles [°]: P1-C1 1.834(2),C1-C32 1.506(4), C32-C1-P1 111.4(2).

6.2.4 Molecular Structure of 3_{ArF}

All hydrogen atoms except the ones attached to N1, N2 and C1, which were found on the Fourier electron difference map, were placed on ideal positions.

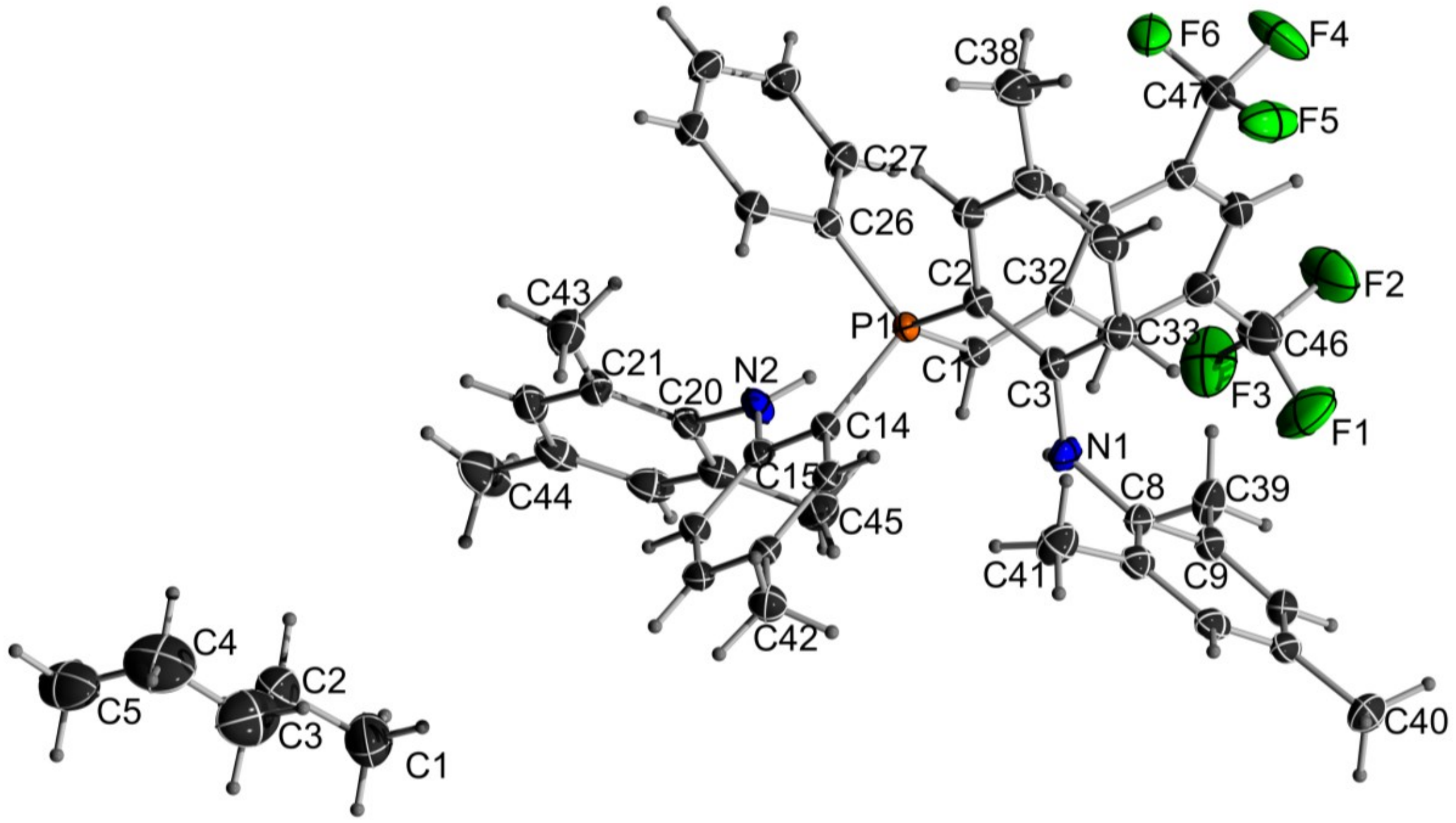

Figure S87: ORTEP of $\mathbf{3_{ArF}}$. Ellipsoids drawn at 50 % probability. Selected bond lengths [Å] and angles [°]: P1-C1 1.710(2), C1-C32 1.435(3), C32-C1-P1 126.0(2).

6.2.5 Molecular Structure of $4_{Ph}Cl$

All hydrogen atoms were placed on ideal positions.

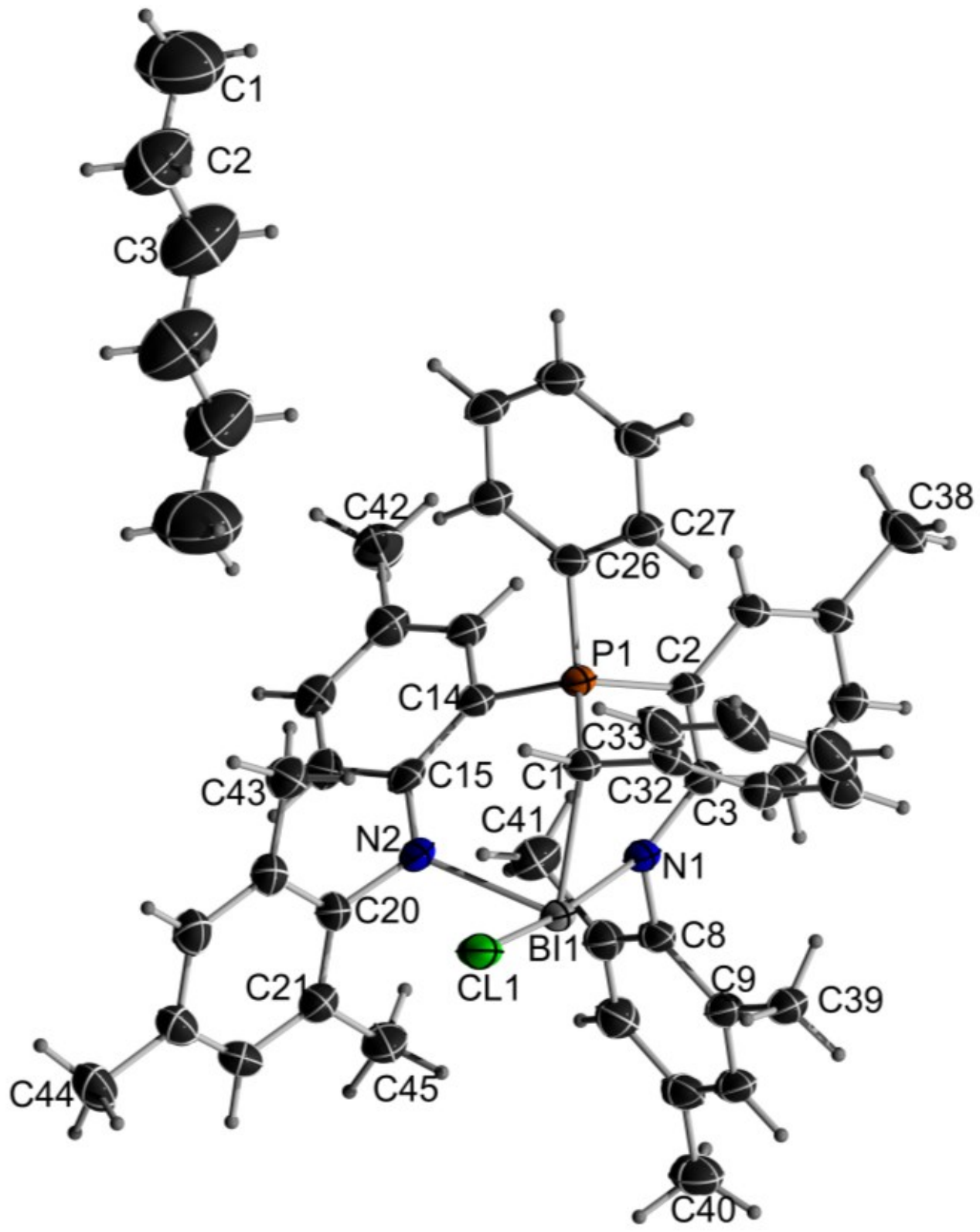

Figure S88: ORTEP of **4_{Ph}Cl**. Ellipsoids drawn at 50 % probability. Selected bond lengths [Å] and angles [°]: Bi1−C1 2.291(3), Bi1−N1 2.395(3), Bi1−N2 2.200(3), Bi−Cl1 2.719(1), P1−C1 1.788(4), N1-Bi1-N2 94.4(1), N1-Bi1-C1 86.2(1), N2-Bi1-C1 83.8(1), N1-Bi1-Cl1 168.2(1), N2-Bi1-Cl1 88.0 (1), C1-Bi1-Cl1 82.6(1).

6.2.6 Molecular Structure of $4_{Ph}Br$

All hydrogen atoms were placed on ideal positions. The crystal structure contained a disordered toluene solvent molecule. The disorder was modelled using SAME, SIMU and DELU restraints and refined using the PART instructions and free variables, which optimized to occupancies of 57 % and 43 %.

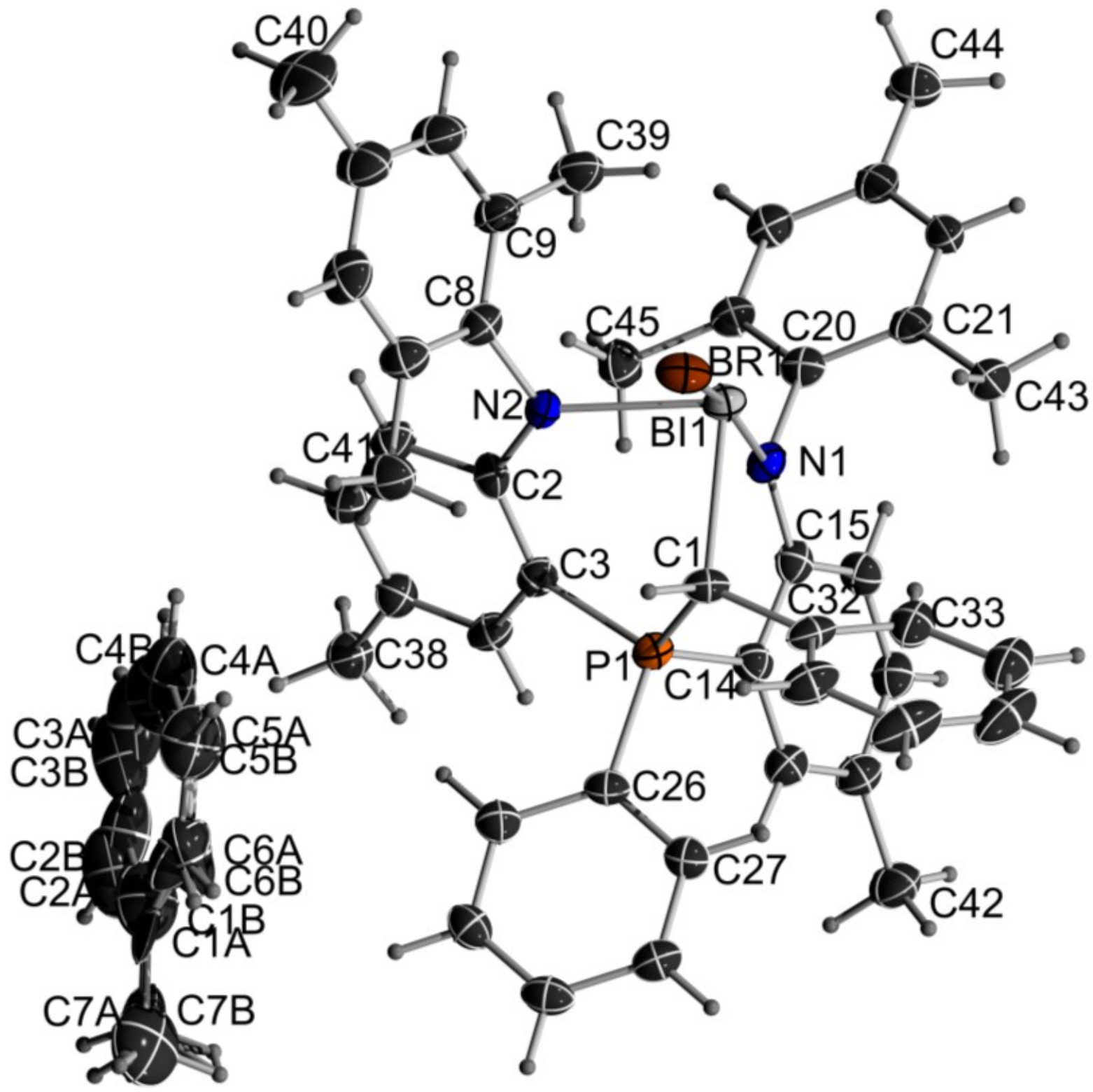

Figure S89: ORTEP of **4_{Ph}Br**. Ellipsoids drawn at 50 % probability. Selected bond lengths [Å] and angles [°]: Bi1−C1 2.290(4), Bi1−N1 2.396(3), Bi1−N2 2.214(3), Bi−Br1 2.8729(5), P1−C1 1.784(4), N1-Bi1-N2 94.6(1), N1-Bi1-C1 86.1(1), N2-Bi1-C1 84.4(1), N1-Bi1-Br1 169.0(1), N2-Bi1-Br1 87.7(1), C1-Bi1-Br1 83.4(1).

6.2.7 Molecular Structure of $4_{Ph}I$

All hydrogen atoms were placed on ideal positions. The crystal structure contained a disordered benzene solvent molecule. The disorder was modelled using the AFIX 66, SIMU and DELU restraints and refined using the PART instructions and free variables, which optimized to occupancies of 87 % and 13 %.

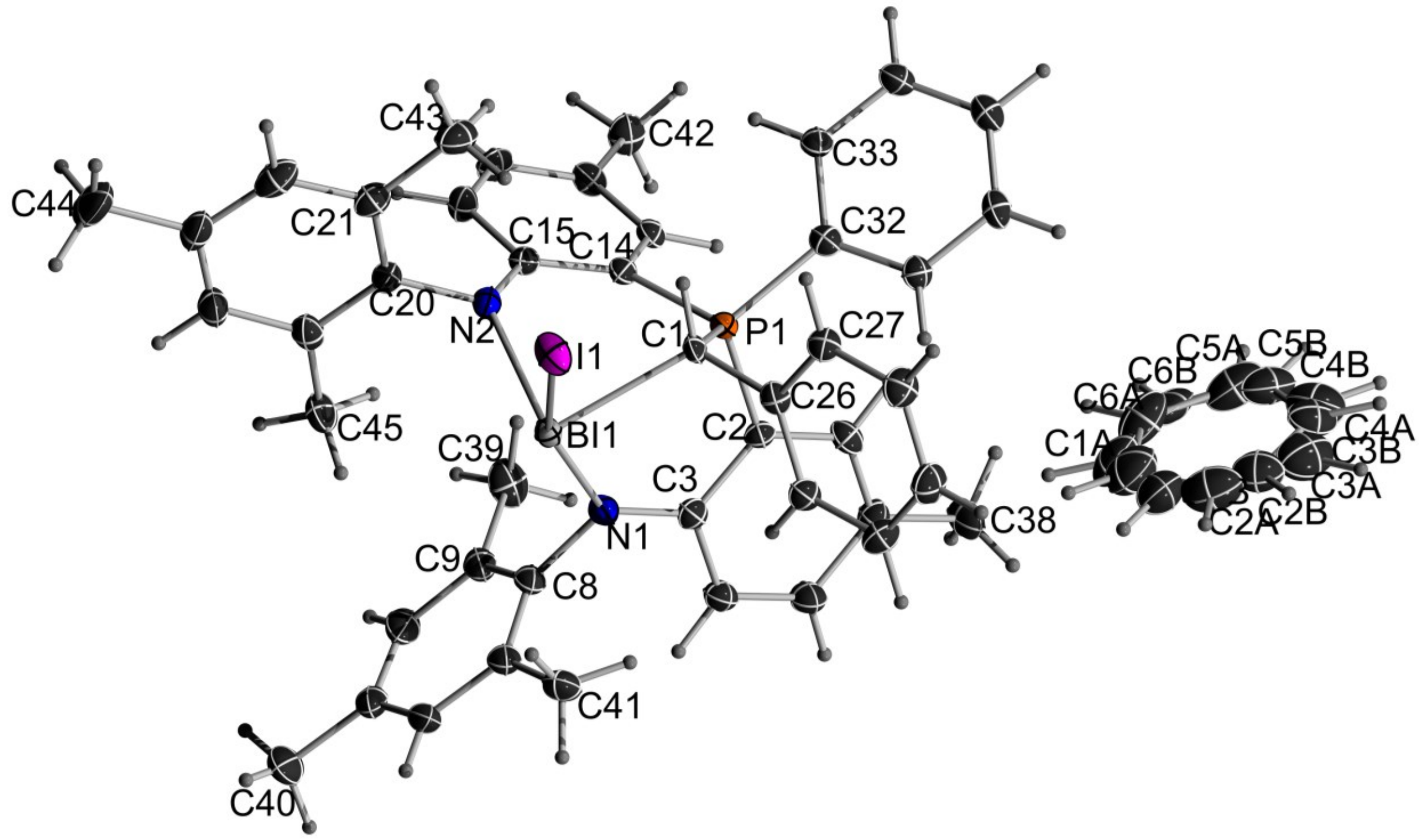

Figure S90: ORTEP of **$4_{Ph}I$**. Ellipsoids drawn at 50 % probability. Selected bond lengths [Å] and angles [°]: Bi1−C1 2.300(3), Bi1−N1 2.369(3), Bi1−N2 2.207(3), Bi−I1 3.1491(3), P1−C1 1.776(4), N1-Bi1-N2 95.2(1), N1-Bi1-C1 85.9(1), N2-Bi1-C1 85.6(1), N1-Bi1-I1 158.7(1), N2-Bi1-I1 99.9(1), C1-Bi1-I1 80.3(1).

6.2.8 Molecular Structure of $\mathbf{4_{ArF}Cl}$

All hydrogen atoms were placed on ideal positions. The crystal structure contained a disordered benzene solvent molecule. The disorder was modelled using the AFIX 66, SIMU and DELU restraints and refined using the PART instructions and free variables, which optimized to occupancies of 53 % and 47 %.

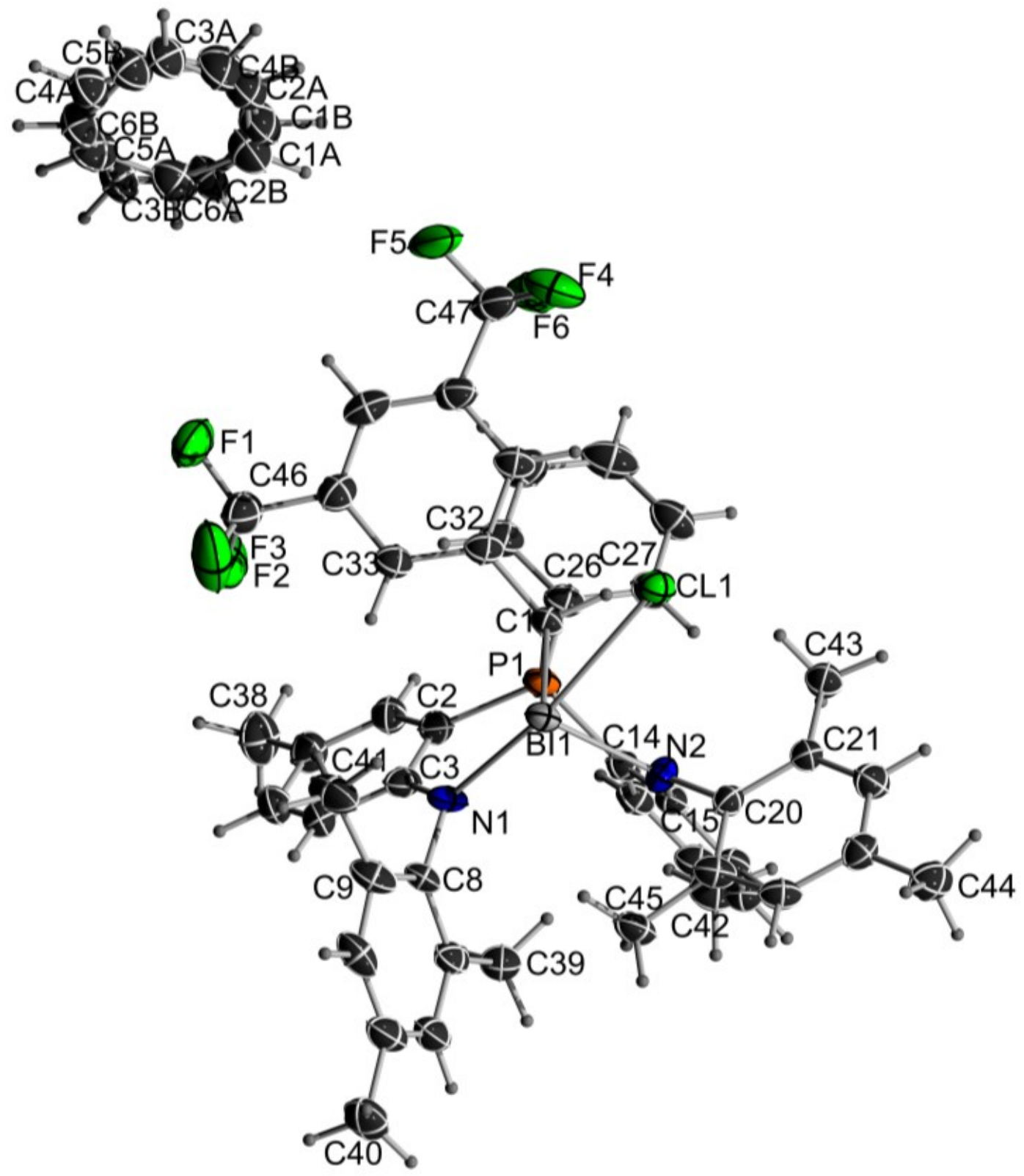

Figure S91: ORTEP of $\mathbf{4_{ArF}Cl}$. Ellipsoids drawn at 50 % probability. Selected bond lengths [Å] and angles [°]: Bi1−C1 2.280(4), Bi1−N1 2.348(4), Bi1−N2 2.256(4), Bi−Cl1 2.8492(11), P1−C1 1.804(5), N1-Bi1-N2 92.8(1), N1-Bi1-C1 85.7(2), N2-Bi1-C1 84.1(2), N1-Bi1-Cl1 164.4(1), N2-Bi1-Cl1 86.2(1), C1-Bi1-Cl1 78.7(1).

6.2.9 Molecular Structure of $4_{ArF}Br$

All hydrogen atoms were placed on ideal positions.

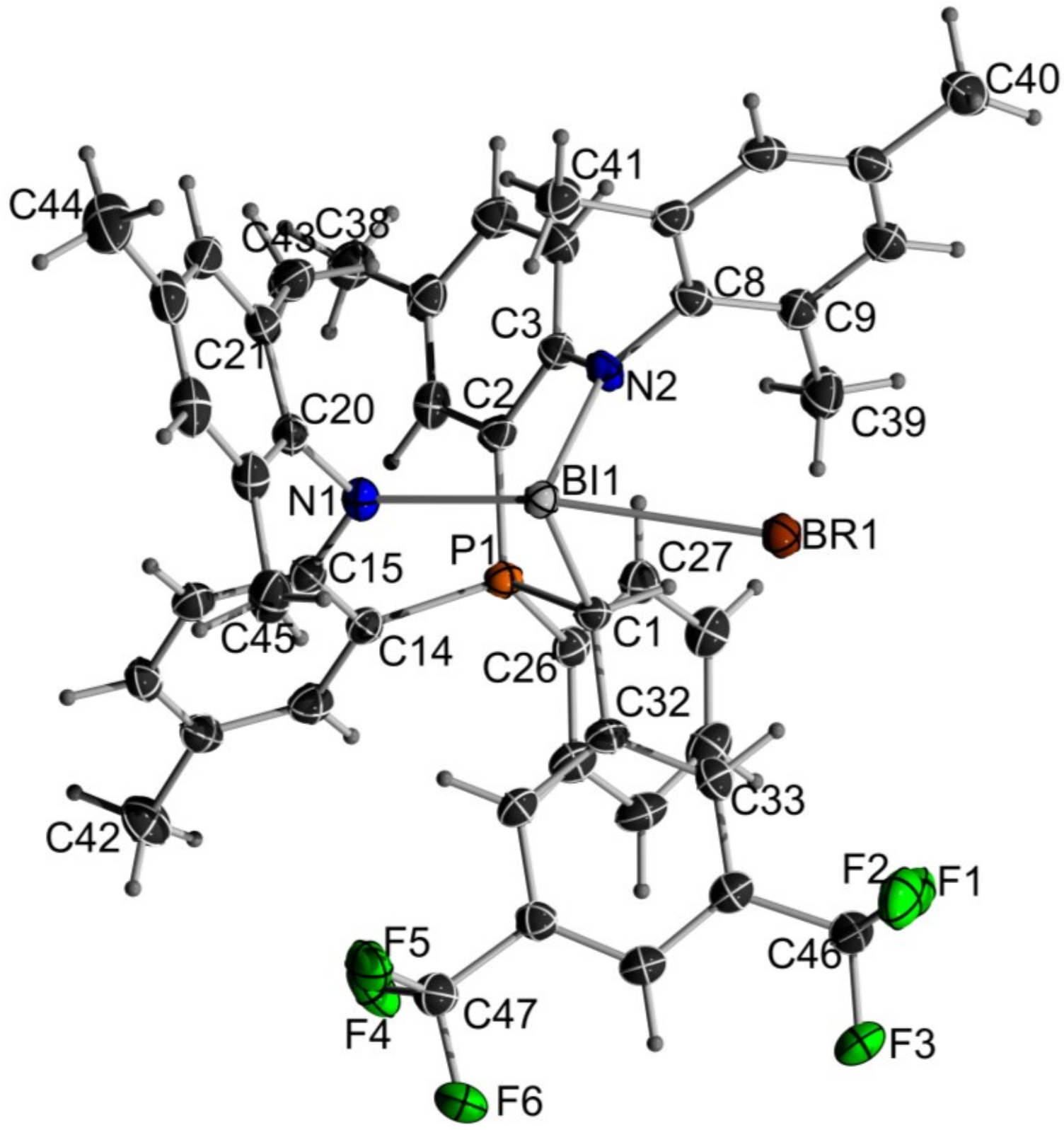

Figure S92: ORTEP of **4_{ArF}Br**. Ellipsoids drawn at 50 % probability. Selected bond lengths [Å] and angles [°]: Bi1−C1 2.290(4), Bi1−N1 2.345(3), Bi1−N2 2.284(3), Bi−Br1 3.0010(4), P1−C1 1.795(4), N1-Bi1-N2 93.2(1), N1-Bi1-C1 87.7(1), N2-Bi1-C1 79.6(1), N1-Bi1-Br1 168.2(1), N2-Bi1-Br1 86.2(1), C1-Bi1-Br1 80.6(1).

6.2.10 Molecular Structure of 4_HCl

All hydrogen atoms were placed on ideal positions.

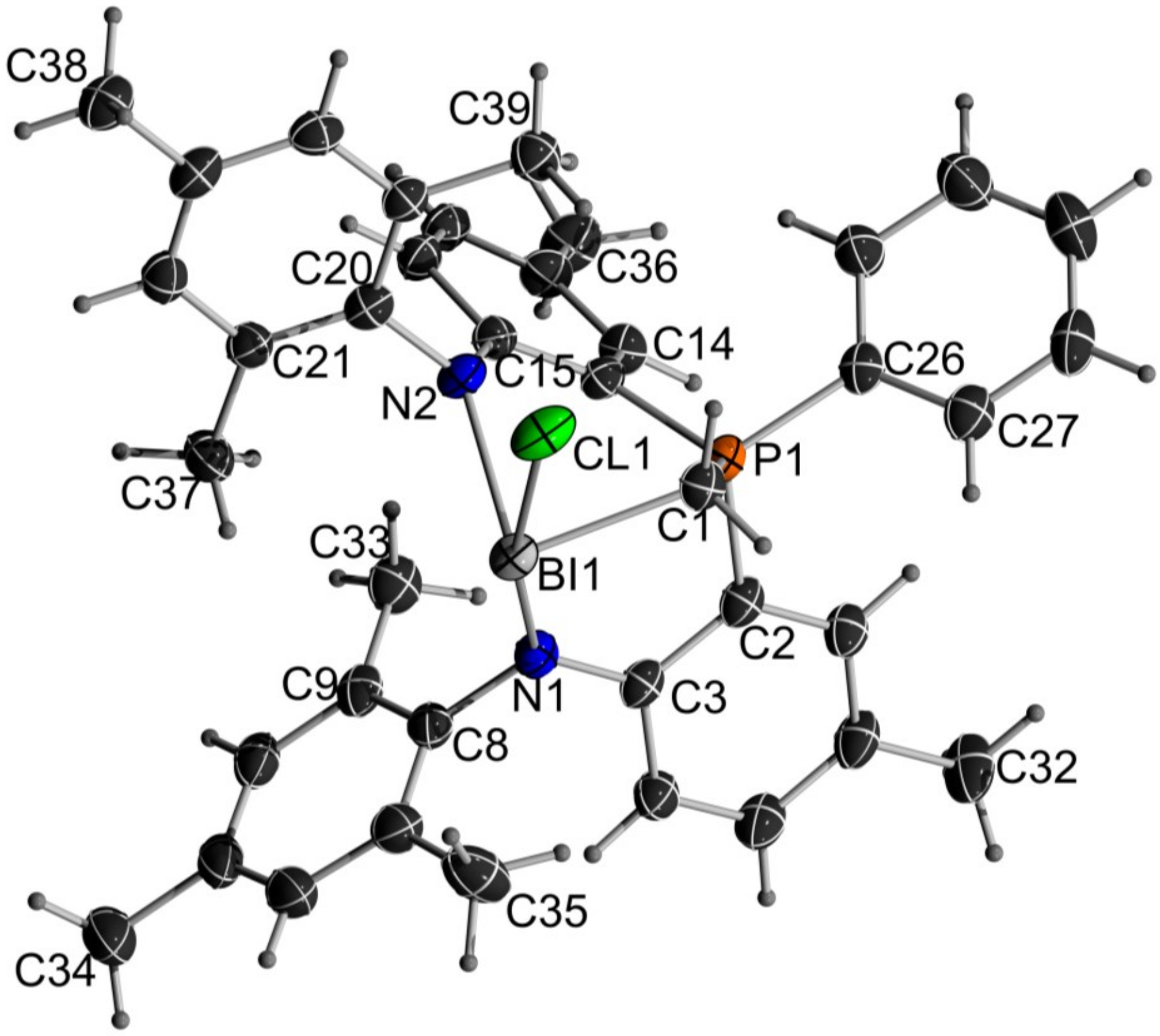

Figure S93: ORTEP of **4_HCl**. Ellipsoids drawn at 50 % probability. Selected bond lengths [Å] and angles [°]: Bi1−C1 2.231(4), Bi1−N1 2.338(3), Bi1−N2 2.252(3), Bi−Cl1 2.854(1), P1−C1 1.768(3), N1-Bi1-N2 94.4(1), N1-Bi1-C1 86.5(1), N2-Bi1-C1 82.6(1), N1-Bi1-Cl1 167.4(1), N2-Bi1-Cl1 87.8(1), C1-Bi1-Cl1 81.5(1).

6.2.11 Molecular Structure of $5_{Ph}OTf$

All hydrogen atoms were placed on ideal positions.

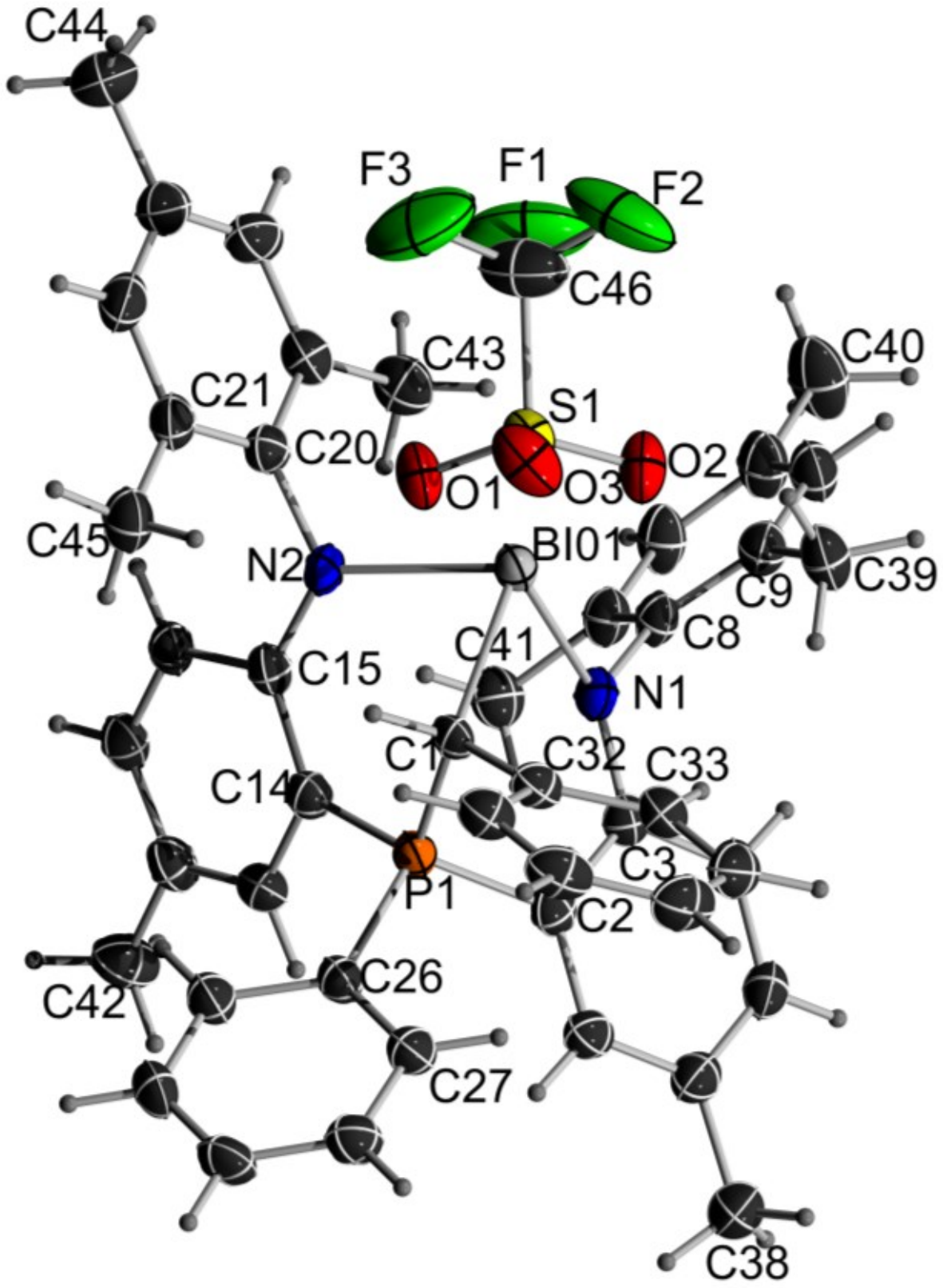

Figure S94: ORTEP of **5_{Ph}OTf**. Ellipsoids drawn at 50 % probability. Selected bond lengths [Å] and angles [°]: Bi1−C1 2.271(4), Bi1−N1 2.242(3), Bi1−N2 2.188(3), P1−C1 1.800(4), N1-Bi1-N2 96.4(1), N1-Bi1-C1 89.6(1), N2-Bi1-C1 84.0(1).

6.2.12 Molecular Structure of $5_{Ph}BPh_4$

All hydrogen atoms were placed on ideal positions.

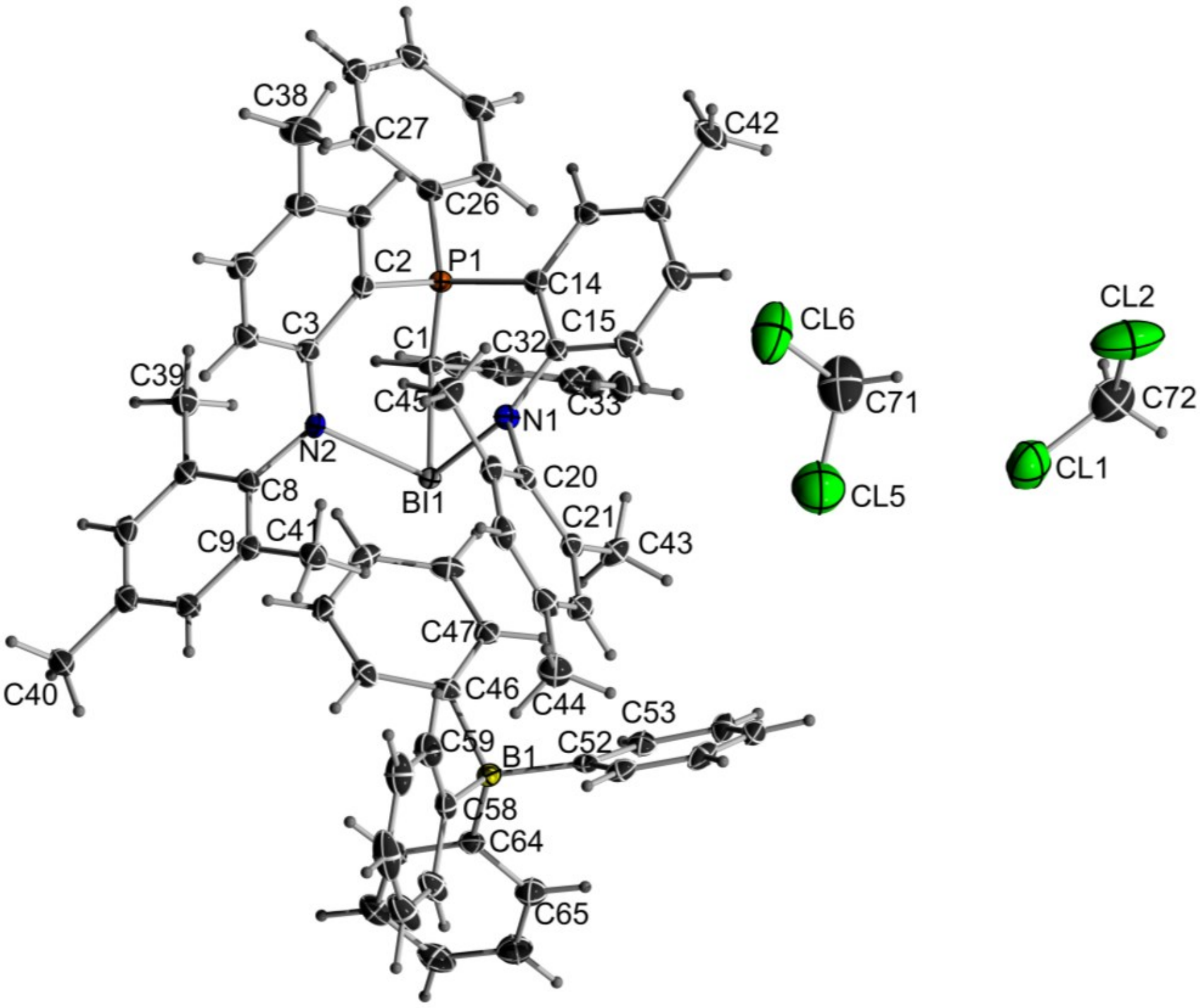

Figure S95: ORTEP of $\mathbf{5_{Ph}BPh_4}$. Ellipsoids drawn at 50 % probability. Selected bond lengths [Å] and angles [°]: Bi1−C1 2.282(3), Bi1−N1 2.210(3), Bi1−N2 2.193(3), P1−C1 1.795(4), N1-Bi1-N2 96.0(1), N1-Bi1-C1 90.8(1), N2-Bi1-C1 82.6(1).

6.2.13 Molecular Structure of 7

All hydrogen atoms were placed on ideal positions. The crystal structure contained a disordered CF_3 group. The disorder was modelled using the SADI and EADP restraints and refined using the PART instructions and free variables, which optimized to occupancies of 57 % and 43 %. The crystal structure also contained a DMAP and a hexane molecule with shared electron occupancy. The disorder was modelled using the AFIX 66, SIMU, DELU and EADP restraints and refined using the PART instructions and free variables, which optimized to occupancies of 52 % and 48 %.

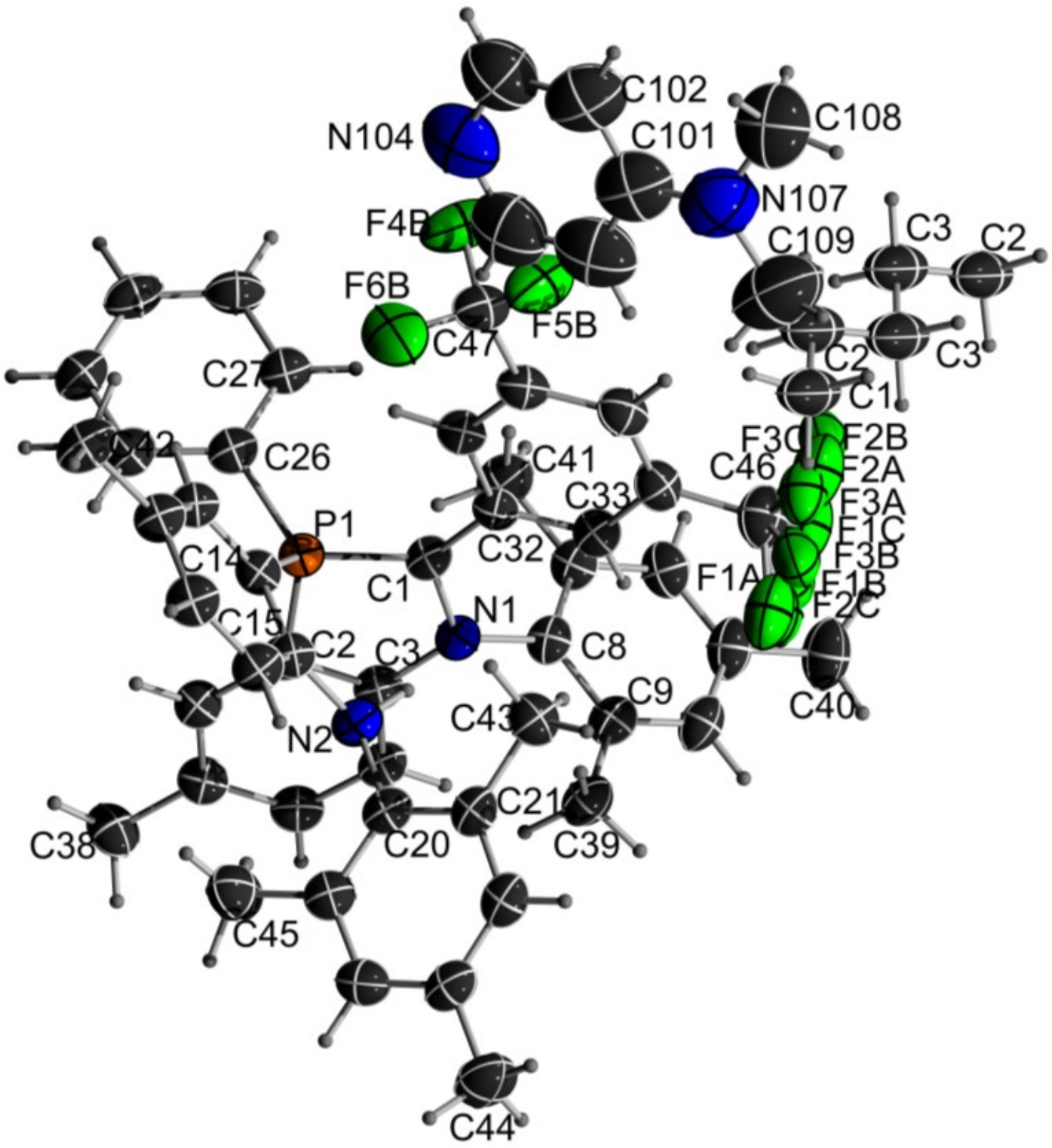

Figure S96: ORTEP of **7**. Ellipsoids drawn at 50 % probability. Selected bond lengths [Å] and angles [°]: P1-C1 1.733(3), C1-N1 1.449(3), P1-C1-N1 109.8(2).

6.2.14 Molecular Structure of 8

All hydrogen atoms were placed on ideal positions. The crystal structure contained a disordered CF_3 group. The disorder was modelled using the SIMU and DELU restraints and refined using the PART instructions and free variables, which optimized to occupancies of 91 % and 9 %.

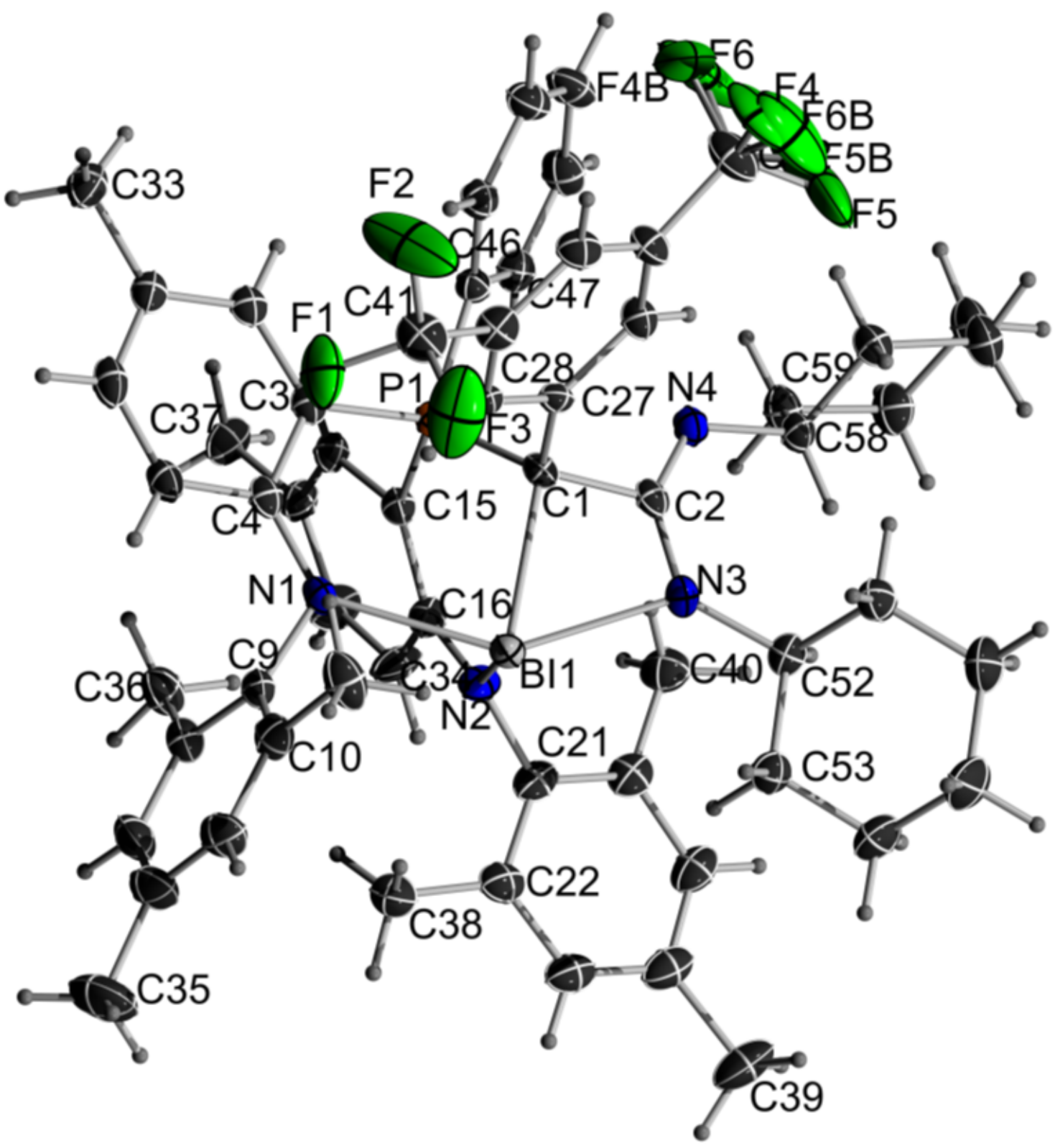

Figure S97: ORTEP of **8**. Ellipsoids drawn at 50 % probability. Selected bond lengths [Å] and angles [°]: Bi1−C1 2.284(3), Bi1−N1 2.400(3), Bi1−N2 2.211(2), Bi−N3 2.301(3), P1−C1 1.803(3), N1-Bi1-N2 90.5(1), N1-Bi1-C1 85.3(1), N2-Bi1-C1 90.8(1), N1-Bi1-N3 145.8(1), N2-Bi1-N3 87.7(1), C1-Bi1-N3 60.6(1).

7 References

[1] E. A. MacLachlan, M. D. Fryzuk, *Organometallics*, **2005**, *24*, 6, 1112
[2] a) G. M. Sheldrick, *Acta Cryst.* **2008**, *A64*, 112; b) G. M. Sheldrick, *Acta Cryst.* **2015**, *C71*, 3; c) A. Thorn, B. Dittrich, G. M. Sheldrick, *Acta Cryst.* **2012**, *A68*, 448; d) G. M. Sheldrick, *Acta Cryst.* **2015**, *A71*, 3
[3] J. Heine, B. Peerless, S. Dehnen, C. Lichtenberg, *Angew. Chem. Int. Ed.* **2023**, 62, e202218771
